# Quinol–Enedione Rearrangement

**DOI:** 10.1021/acs.orglett.5c01266

**Published:** 2025-04-30

**Authors:** Tomás Vieira de Castro, François Richard, Steven H. Bennett, Caspar S. Lamborelle, Gary S. Nichol, Rafał Szabla, Andrew L. Lawrence

**Affiliations:** † EaStCHEM School of Chemistry, University of Edinburgh, Joseph Black Building, David Brewster Road, Edinburgh EH9 3FJ, U.K.; ‡ Institute of Advanced Materials, Faculty of Chemistry, Wrocław University of Science and Technology, 50-370 Wrocław, Poland; § Department of Physics, Faculty of Science, University of Ostrava, 30. dubna 22, 701 03 Ostrava, Czech Republic

## Abstract

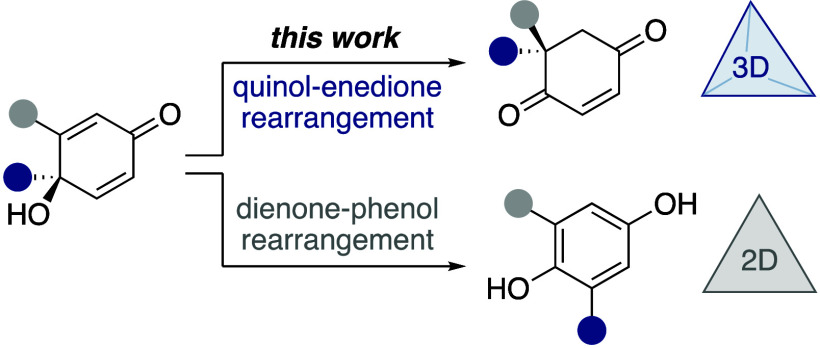

The quinol–enedione rearrangement enables the synthesis of 2-cyclohexene-1,4-diones from readily available *para*-quinol substrates. Building on sporadic early reports of this transformation, we have optimized the reaction conditions and systematically investigated its substrate scope. The utility of Brønsted acid-mediated reaction conditions for a variety of quinol derivatives, including those with substituted and unsubstituted migrating termini, is highlighted. Notably, kinetic selectivity between quinol–enedione and dienone–phenol rearrangements is demonstrated. The synthetic potential of the enedione products is showcased through a range of transformations, leading to the formation of complex polycyclic structures. These findings provide a valuable framework for recognizing and applying the quinol–enedione rearrangement in synthesis, while computational studies offer valuable insights into its mechanistic underpinnings.

First reported over a century ago, the dienone–phenol rearrangement (also known as the Auwers–Inhoffen rearrangement) is a historically important reaction (Scheme 1a).^1^ Despite inherently reducing structural complexity—converting a three-dimensional precursor into a two-dimensional product—it has found widespread application in target-oriented synthesis.^2^ This enduring popularity can be partly attributed to the reliable and robust nature of the reaction, which is driven by the aromatic stability of the phenol product. A common variant of the dienone–phenol rearrangement involves a quinol substrate, which undergoes rearrangement to give a hydroquinone product (Scheme 1b).^3^ The final aromatization step in these rearrangements requires the migrating termini to be unsubstituted. For quinol substrates possessing substituted migrating termini a final deprotonation cannot occur, which results in the formation of a 2-cyclohexene-1,4-dione, or “enedione”, product (Scheme 1c).^4^ Unlike the dienone–phenol rearrangement, the quinol–enedione rearrangement preserves three-dimensional structural complexity, making it a potentially useful reaction for the synthesis of targets featuring challenging quaternary centers.^5^ In 1968, Davis and co-workers reported the first example of a quinol–enedione rearrangement, treating *para*-quinol **1a** to excess boron trifluoride etherate (Scheme 2a).^4a,4b^ The resulting enedione **2a** was described as an unstable oil and was not purified but instead immediately reduced to the diketone **3**, with no yield given (Scheme 2a). The reported instability of the enedione product **2a** may have discouraged further investigation into the development of this reaction. Since Davis’ seminal work, only four additional examples of quinol–enedione rearrangements have been reported (see Supporting Information for details).^4c,6^ However, no detailed reaction development has been undertaken to advance a more broadly useful synthetic methodology. Our interest in the quinol–enedione rearrangement was sparked by the prevalence of the enedione motif in numerous natural products (Scheme 2b).^6,7^ Indeed, this largely overlooked rearrangement likely plays an important role in several biosynthetic pathways.^8^

**Scheme 1:**
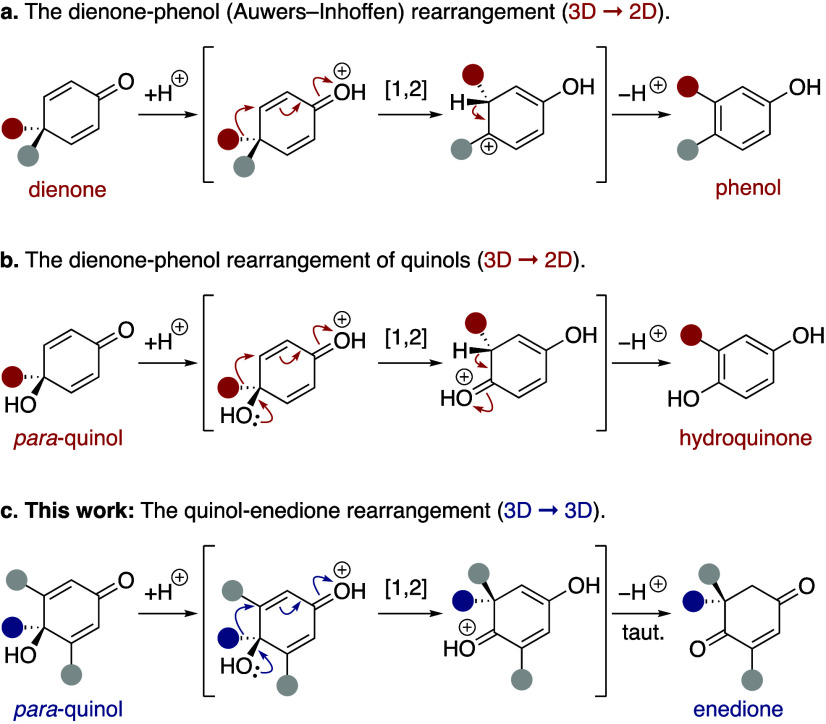
Dienone–Phenol and Quinol–Enedione Rearrangements, Shown Proceeding via Brønsted Acid Catalyzed Mechanisms

**Scheme 2:**
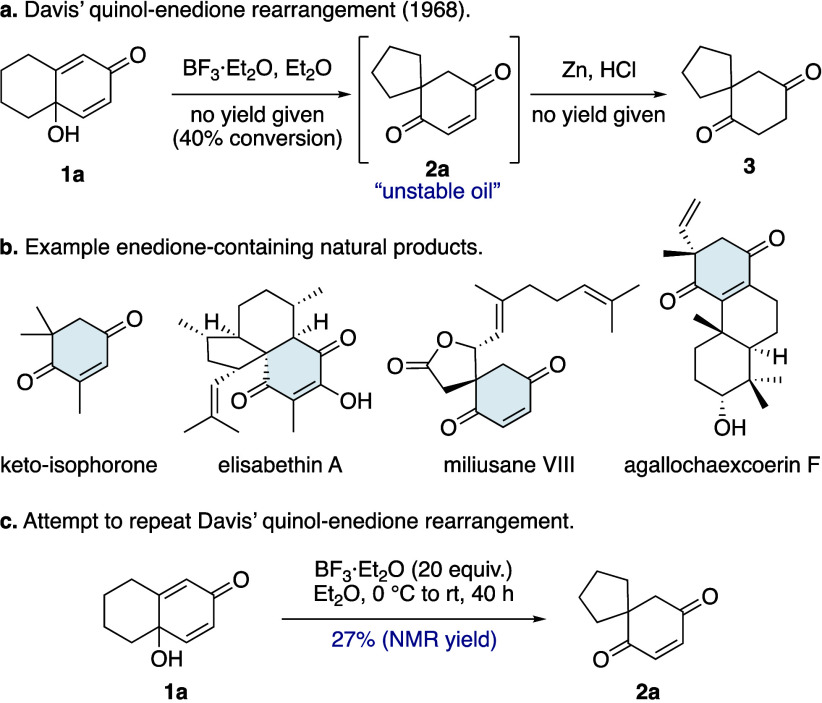
Davis’ Seminal Quinol–Enedione Rearrangement and Representative Natural Products That Contain the Enedione Framework

The required *para*-quinol starting materials are bench-stable, easily handled compounds that can be accessed in a single step from their corresponding phenols (see Supporting Information for details).^9^ We began by screening reaction conditions for quinol **1a**, the substrate originally studied by Davis and co-workers (Table 1a). Our attempt to use boron trifluoride etherate, as reported by Davis and co-workers,^4a,4b^ resulted in a complex mixture, with enedione **2a** present in just 27% NMR yield (Scheme 2c). Our attempts to use basic reaction conditions led to significant decomposition,^4c,4e,4f^ whereas treatment with stoichiometric Brønsted acids in CH_2_Cl_2_ at ambient temperature led to relatively clean formation of enedione **2a** (Table 1a).^4d^ PPTS, TFA, and aqueous HCl gave only modest yields (Table 1a, Entries 1–3), while CSA and *p*-TsOH afforded improved yields of 66% and 74%, respectively (Table 1a, Entries 4 and 5). Further solvent screening (Table 1a, Entries 6–10) identified 1,2-dichloroethane as an optimal solvent for the rearrangement with *p*-TsOH (Table 1a, Entry 10). Notably, substoichiometric amounts of *p*-TsOH were found to be sufficient, with just 5 mol % providing an 85% yield (Table 1a, Entry 11). Increasing the reaction concentration from 35 mM to a more practical 0.1 M also improved the yield (Table 1a, Entry 12). Moreover, the reaction proved scalable, with a two-gram batch of quinol **1a** undergoing the rearrangement to give enedione **2a** in 95% isolated yield (Table 1a, Entry 13). In contrast to previous reports of instability,^4a,4b^ spirocycle **2a** was found to be a bench-stable, crystalline solid, readily purified by standard column chromatography or recrystallization. Indeed, the structure of enedione **2a** was unequivocally confirmed through single-crystal X-ray structure analysis (Table 1a). Re-exposing enedione **2a** to the reaction conditions led to partial reformation of a small quantity of quinol **1a** (∼3%), revealing that this quinol–enedione rearrangement is reversible under the reaction conditions.

**Table 1:**
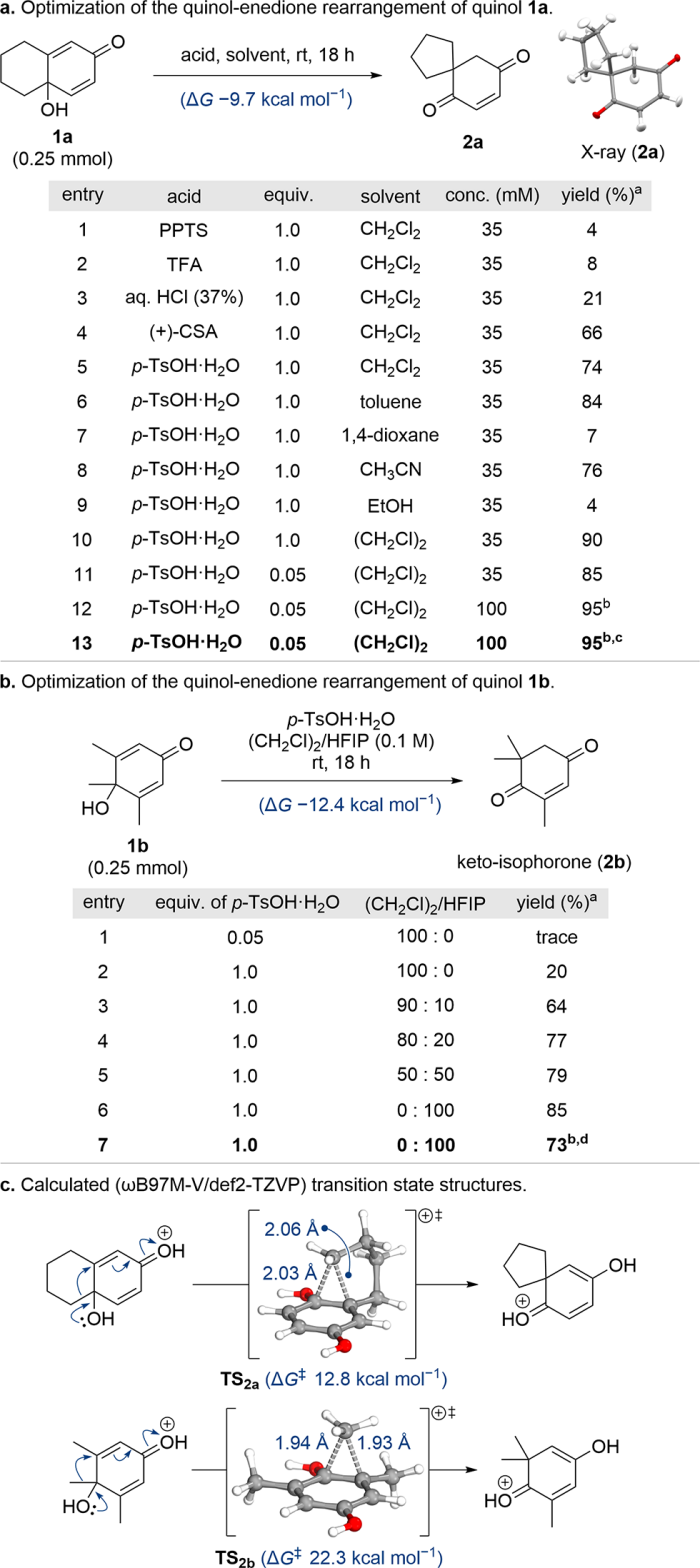
Optimization of the Quinol–Enedione Rearrangement for Quinols **1a** and **1b**, with Calculated (ωB97M-V/def2-TZVP) Energy Values and Transition State Structures

a Determined by ^1^H NMR spectroscopy using 1,4-dinitrobenzene as internal standard.

b Isolated yield.

c Reaction performed on 2.00 g (12.2 mmol) of quinol **1a**.

d Reaction performed on 390 mg (2.5 mmol) of quinol **1b**.

Although many quinol substrates rearranged efficiently under these conditions (*vide infra*), those with untethered migrating substituents of poor migratory aptitude yielded little or no product. A particularly challenging case was trimethyl quinol **1b** (Table 1b), which was expected to rearrange into keto-isophorone (**2b**),^7b^ a natural product found in various plants and widely used in the industrial synthesis of carotenoids and vitamin E.^10^ However, under our optimized conditions, quinol **1b** failed to undergo appreciable rearrangement (Table 1b, Entry 1). A modest 20% yield of keto-isophorone (**2b**) could be achieved if a stoichiometric amount of *p*-TsOH was used (Table 1b, Entry 2). Several studies have detailed the use of hexafluoroisopropanol (HFIP) to enhance the efficiency of Brønsted acid-mediated reactions.^11^ Introducing HFIP as a cosolvent had a dramatic impact on the yield of keto-isophorone (**2b**) formed (Table 1b, Entries 3–5). The best result was obtained when HFIP was employed as the sole solvent (Table 1b, Entry 6), with keto-isophorone (**2b**) isolated in 73% yield on a 2.5 mmol scale (Table 1b, Entry 7). Unlike enedione **2a**, when keto-isophorone (**2b**) was re-exposed to the reaction conditions no reverse quinol–enedione rearrangement was observed.

Density functional theory (DFT) calculations, at the ωB97M-V/def2-TZVP level of theory, were undertaken to investigate the difference in reactivity of quinols **1a** and **1b** (Table 1c).^12^ The relative change in free energy for the quinol–enedione rearrangement of quinol **1b** is calculated to be more favorable (Δ*G* −12.4 kcal mol^–1^) than for quinol **1a** (Δ*G* −9.7 kcal mol^–1^) (Table 1a and 1b). However, the barrier for the rearrangement of quinol **1a** (Δ*G*^‡^ 12.8 kcal mol^–1^) is significantly lower than for quinol **1b** (Δ*G*^‡^ 22.3 kcal mol^–1^) (Table 1c), which aligns with our experimental observations. Despite the two transition state structures (**TS**_**2a**_ and **TS**_**2b**_) being very different in relative energy they both reveal highly synchronous, concerted migration steps (Δ*r***_2a_** 0.03 Å; Δ*r***_2b_** 0.01 Å) (Table 1c). In summary, two sets of reaction conditions have been developed for quinol–enedione rearrangements (Table 1). For substrates with good migrating groups, use of substoichiometric *p*-TsOH in 1,2-dichloroethane is sufficient (Table 1a; conditions **A**), whereas for more recalcitrant substrates use of stoichiometric *p*-TsOH in HFIP can be employed (Table 1b; conditions **B**).

The substrate scope of the quinol–enedione rearrangement was examined using 16 carefully selected quinol substrates (**1a**–**p**). Initially, a series of relatively simple quinols (**1a**–**g**) was investigated (Scheme 3). Migration of the *n*-butyl group in quinol **1c**, to give enedione **2c**, proceeded in higher yield under reaction conditions **B**. Rearrangement of quinol **1d**, under reaction conditions **A**, gave enedione **2d** in 73% yield and a d.r. of 7:1, thanks to a diastereoselective final tautomerization. Aryl group migration in quinols **1e**–**g** was achieved under reaction conditions **B**, with the reaction time correlating with the electronic properties of the migrating groups. The phenyl group in quinol **1e** migrated in 80% yield within 2 h, while quinol **1f** underwent rearrangement in just 30 min, affording enedione **2f** in 72% yield. In contrast, quinol **1g** required 16 h to give enedione **2g** in a slightly lower yield of 56% (Scheme 3). The stereospecific nature of the quinol–enedione rearrangement mechanism was evident in the rearrangement of estrone-derived quinol **1h** to give enedione **2h** in 73% yield (Scheme 3).^4d^

**Scheme 3:**
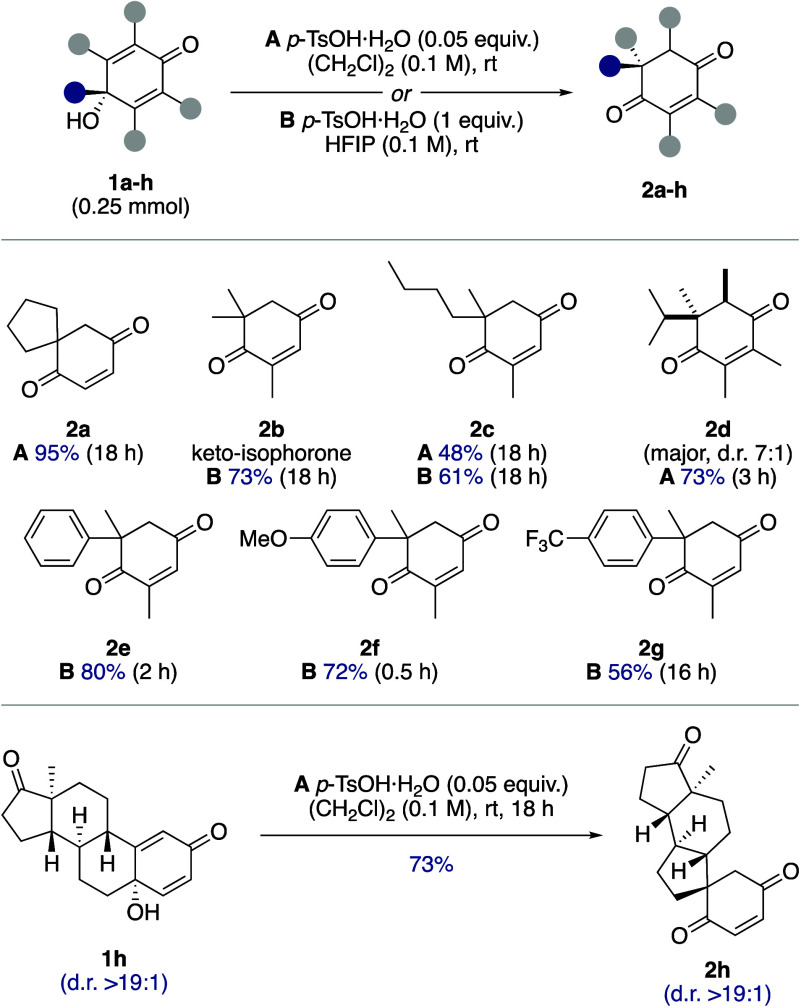
Substrate Scope for the Quinol−Enedione Rearrangement

A further set of substrates (**1i**–**m**) was designed where the quinol has both a substituted and unsubstituted migrating terminus (Scheme 4). This enables the selectivity between quinol–enedione and dienone–phenol rearrangements to be assessed, which could be very important when applying this reaction in complex settings.

**Scheme 4:**
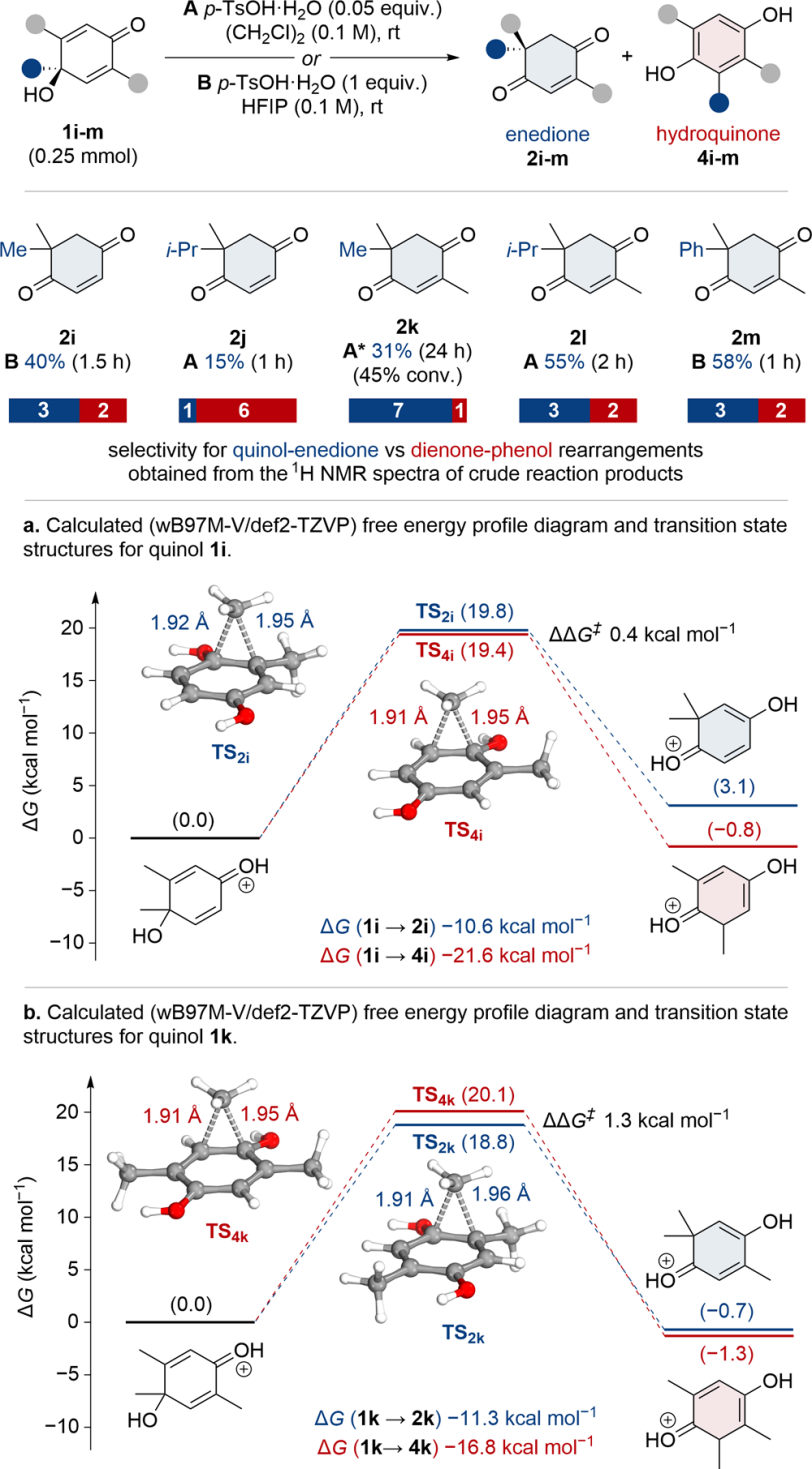
Investigating Selectivity between Quinol–Enedione and Dienone–Phenol Rearrangements 1.0 equiv of *p*-TsOH used.

Quinol **1i**, under reaction conditions **B**, exhibited a preference for the quinol–enedione rearrangement (∼3:2), with enedione **2i** isolated in 40% yield (Scheme 4). Thus, despite the potential risk of a thermodynamically favorable formation of hydroquinone **4i**, a kinetic preference for the enedione product **2i** is observed. With the larger *iso*-propyl migrating group in quinol **1j**, however, there is a switch in selectivity toward the dienone–phenol rearrangement (∼1:6), with enedione **2j** isolated in just 15% yield. It was envisaged that inclusion of a distal methyl substituent on the quinol (**1k**–**m**) would reduce the electrophilicity of the unsubstituted migrating terminus and thus increase the kinetic preference for quinol–enedione rearrangements.^13^ Indeed, the rearrangement of quinol **1k** exhibited marked selectivity for the quinol–enedione rearrangement (∼7:1), with enedione **2k** isolated in 31% yield (69% BRSM) after 24 h (Scheme 4).^14^ Quinols **1l** and **1m** also rearranged with modest quinol–enedione selectivity (∼3:2), giving enediones **2l** and **2m** in 55% and 58% yield, respectively (Scheme 4). The influence of the distal methyl substituent on the enedione/hydroquinone selectivity is also reflected by further DFT calculations.^12^ For quinol **1i** the barriers for the quinol–enedione (Δ*G*^‡^ 19.8 kcal mol^–1^) and dienone–phenol (Δ*G*^‡^ 19.4 kcal mol^–1^) rearrangements were calculated to be very close (ΔΔ*G*^‡^ 0.4 kcal mol^–1^) (Scheme 4a). This is consistent with the observed enedione (**2i**) to hydroquinone (**4i**) distribution of ∼3:2, which suggests that the associated Gibbs free energy barriers should be virtually identical for a kinetically controlled reaction. Even though the DFT calculations suggest the opposite trend in the product distribution, the computed results are well within the expected accuracy of the ωB97M-V density functional approximation for barrier heights.^12^ For quinol **1k** the calculated barrier for the quinol–enedione rearrangement (Δ*G*^‡^ 18.8 kcal mol^–1^) was appreciably lower (ΔΔ*G*^‡^ 1.3 kcal mol^–1^) than the barrier for the dienone–phenol rearrangement (Δ*G*^‡^ 20.1 kcal mol^–1^) (Scheme 4b). Even though the ΔΔ*G*^‡^ remains small, it clearly demonstrates the preference for formation of enedione **2k** from quinol **1k**.

Several quinol substrates we tested failed to undergo the quinol–enedione rearrangement (Scheme 5). For example, quinols **1n** and **1o** did not rearrange under conditions **A** and degraded when subjected to conditions **B**. Rearrangement of allyl-substituted quinol **1p** under reaction conditions **A** gave just 15% of the expected enedione **2p**, with the major product, hydroquinone **5**, being the result of an oxy-Cope rearrangement (Scheme 5).

**Scheme 5:**
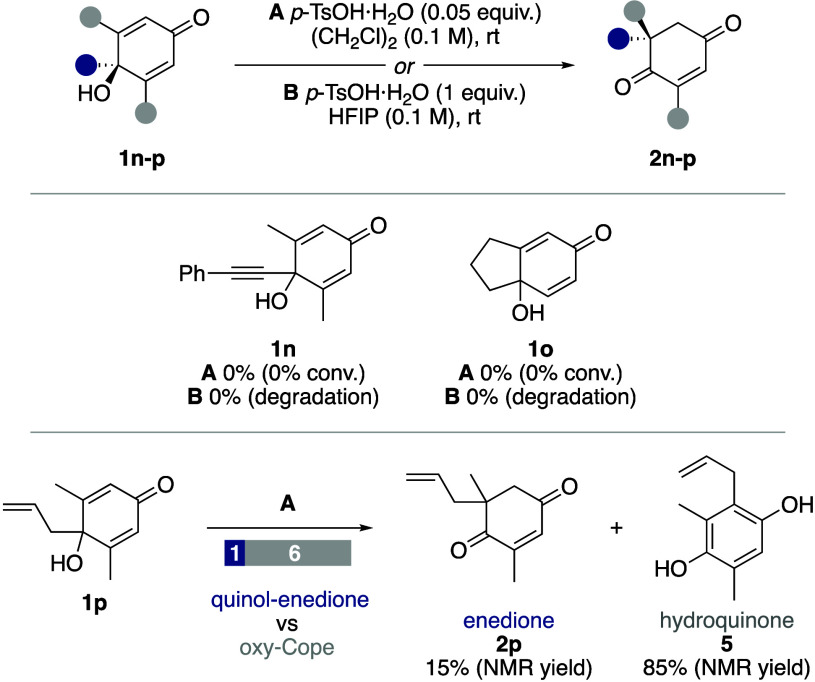
Failed Substrates for the Quinol–Enedione Rearrangement

The enedione products are simple, yet three-dimensionally rich building blocks with multiple synthetic handles, and so with a generalized methodology established we were curious to explore their synthetic potential. Davis and co-workers reported selective alkene reduction of enedione **2a** under dissolving metal conditions to give diketone **3** (Scheme 2a). Under Luche conditions the ketones can be selectively reduced to give diol **6** in 74% yield as a mixture of diastereomers (Scheme 6). Epoxidation using hydrogen peroxide under basic conditions gives epoxide **7** in 60% yield. The quaternary center in enedione **2a** can impart useful regiocontrol in the Michael addition of diethyl malonate to give product **8** as the major regioisomer (r.r. 6:1) in 98% combined yield. This substrate-controlled regioselectivity is also evident in the selective formation of tosyl hydrazone **9** in 64% yield, which gives access to a range of other useful reactions.^15^ Perhaps the greatest synthetic potential for enediones is their ability to engage in cycloadditions. For example, the Diels–Alder reaction of enedione **2a** with cyclopentadiene at ambient temperature gives *endo*-adduct **10** in 95% yield. Regioselective Diels–Alder reactions with unsymmetrical dienes can also be achieved. For example, the Diels–Alder reaction between enedione **2a** and isoprene using Sc(OTf)_3_ gives the tricyclic product **11** in a 5:1 r.r. and 80% combined yield. While the election-deficient enedione is a good dienophile, we hypothesized that enolization of enedione **2a** could generate a diene capable of reacting with various dienophiles to give access to complex fused-bridged-spiro polycyclic ring systems. Thus, acetylation of enedione **2a** under standard conditions gives enol acetate **12**, which was then used directly in Diels–Alder reactions with *N*-phenylmaleimide (NPM), dimethyl acetylenedicarboxylate (DMAD) or 4-phenyl-1,2,4-triazole-3,5-dione (PTAD), to give polycycles **13**–**15** (Scheme 6).

**Scheme 6:**
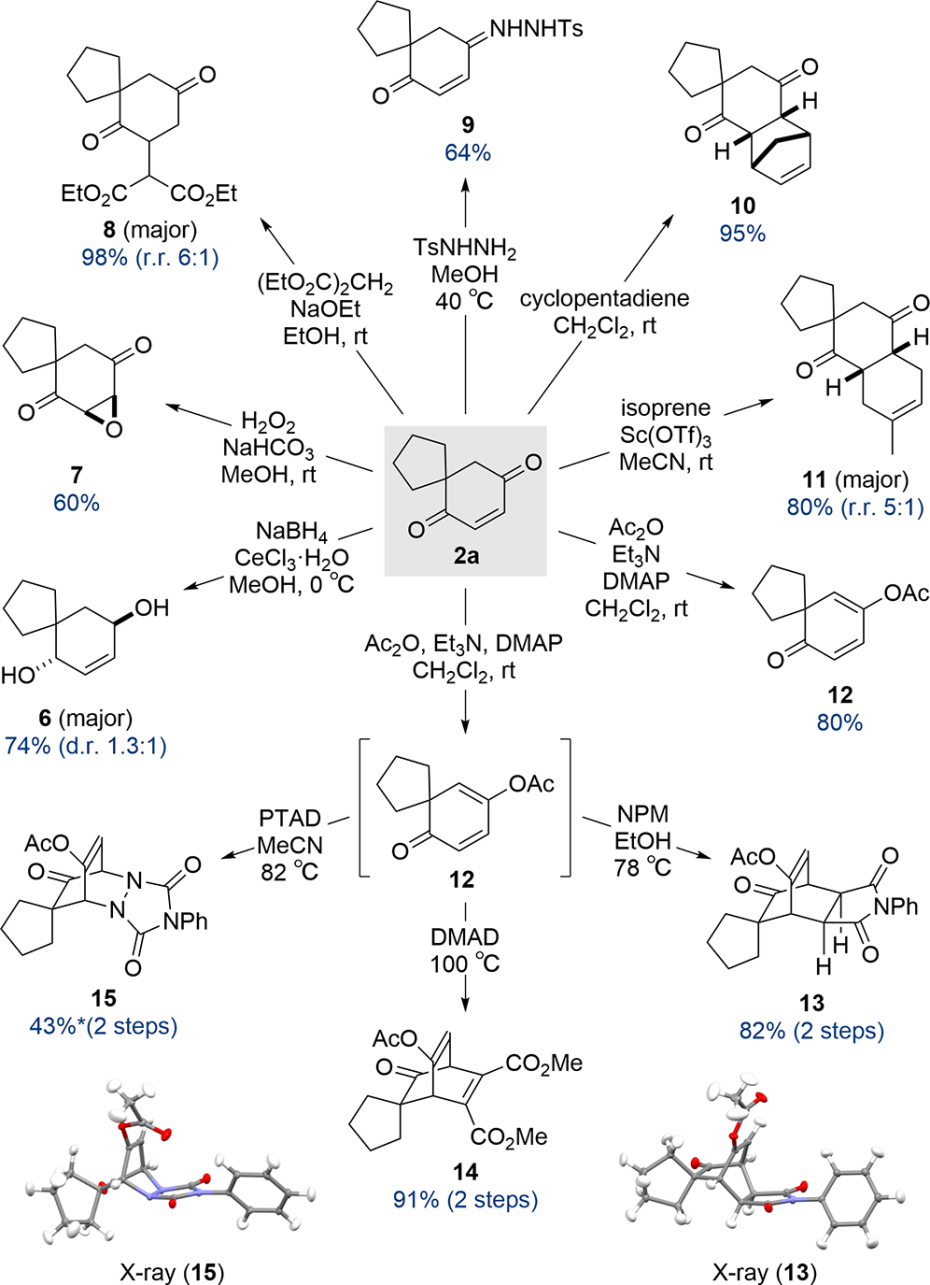
Derivatization Studies on Model Enedione **2a** 23% yield of the diketone formed from hydrolysis of enol acetate **15** was also isolated.

In summary, we have established the quinol–enedione rearrangement as a powerful synthetic tool for accessing valuable enedione products. The utility of this transformation is exemplified by our strategically novel two-step synthesis of the commercially important natural product keto-isophorone (**2b**) from 3,4,5-trimethylphenol, via quinol **1b** (Table 1b).^10^ By expanding the synthetic utility of the quinol–enedione rearrangement and offering insights into its selectivity relative to the dienone–phenol rearrangement, this work establishes a foundation for its broader application in organic synthesis.^16^

## Data Availability

The data underlying this study are available in the published article and its Supporting Information.

## Supporting Information Available

Experimental procedures, including the synthesis of starting materials and characterization data, NMR spectral data for all new compounds, description of computational methods, and an overview of previous reports of quinol–enedione rearrangements (PDF)
Optimized geometries of the stationary points and converged minimum-energy paths in *xyz* format with the corresponding potential energies (ZIP)
