# Supplementary material for: Quinol–Enedione Rearrangement
Source: Org Lett. 2025 Apr 30;27(18):4782–7. doi: 10.1021/acs.orglett.5c01266 (PMC12070464; doi:10.1021/acs.orglett.5c01266)
Supplement: Supplementary file 1 — ol5c01266_si_001.pdf [file ol5c01266_si_001.pdf]

Quinol–Enedione Rearrangement

Tomás Vieira de Castro,[a] François Richard,[a,‡] Steven H. Bennett,[a,‡] Caspar S. Lamborelle,[a] Gary S. Nichol,[a] Rafał Szabla,[b,*] Andrew L. Lawrence [a,*]

[a] EaStCHEM School of Chemistry, University of Edinburgh, Joseph Black Building, David Brewster Road, Edinburgh, EH9 3FJ, UK. [b] Department of Physical and Quantum Chemistry, Faculty of Chemistry, Wrocław University of Science and Technology, Wrocław, Poland & Department of Physics, Faculty of Science, University of Ostrava, 30. dubna 22, 701 03 Ostrava, Czech Republic.

*e-mail: a.lawrence@ed.ac.uk, rafal.szabla@pwr.edu.pl

Table of Contents

1. Previously reported quinol–enedione rearrangements 9

2. General Experimental 10

2.1. Thin-Layer Chromatography (TLC) 10

2.2. Chromatography 10

2.3. Solvents 10

2.4. Analysis 11

3. Optimisation and Control Experiments 12

3.1 Optimisation of the quinol–enedione rearrangement 12

3.1.1 Optimisation of tethered *p*-quinol **1a** 12

3.1.2 Optimisation of trimethyl *p*-quinol **1b** 13

3.2. Investigation of previously reported conditions by Davis and co-workers[1,2] 14

3.2.1. ^{1}H NMR Spectrum of crude reaction mixture using Davis' conditions (500 MHz, $CDCl_3$) 15

3.2.2. ^{1}H NMR Spectrum of crude reaction mixture using Conditions **A** (500 MHz, $CDCl_3$) 15

3.3. Reversibility Experiments 16

3.3.1. ^{1}H NMR Spectra of Tethered Enedione **2a** Reversibility 17

3.3.2. ^{1}H NMR Spectra of Keto-Isophorone (**2b**) Reversibility 17

4. Synthesis of Biphenyl Starting Materials 18

4.1. General Procedure 1: Synthesis of Biphenyls 18

4.2. Biphenyl **S3** 19

4.3. Biphenyl **S5** 20

4.4. Biphenyl **S7** 21

5. Synthesis of *p*-Quinol Substrates 22

5.1. General Procedure 2: Synthesis of *p*-quinols using Oxone® 22

5.2. General Procedure 3: Synthesis of *p*-quinols using PIDA 23

5.3. General Procedure 4: Synthesis of *p*-quinols using organolithium addition 24

5.4. General Procedure 5: Synthesis of *p*-quinols using Grignard addition 25

5.5. Tethered *p*-quinol (**1a**) 26

5.6. 3,4,5-trimethyl *p*-quinol (**1b**) 27

5.7. 4-Butyl 3,5-dimethyl *p*-quinol (**1c**) 28

5.8. 4-Isopropyl 2,3,5,6-tetramethyl *p*-quinol (**1d**) 29
5.9. 3,5-Dimethyl 4-phenyl *p*-quinol (**1e**) 30
5.10. 3,5-Dimethyl 4-(4-methoxyphenyl) *p*-quinol (**1f**) 31
5.11. 3,5-Dimethyl 4-(4-trifluoromethylphenyl) *p*-quinol (**1g**) 32
5.12. Estrone *p*-quinol (**1h**) 33
5.15. 3,4-Dimethyl *p*-quinol (**1i**) 34
5.16. 4-Isopropyl 3-methyl *p*-quinol (**1j**) 35
5.17. 2,4,5-Trimethyl *p*-quinol (**1k**) 36
5.18. 2,5-Dimethyl 4-isopropyl *p*-quinol (**1l**) 37
5.19. 2,5-Dimethyl 4-phenyl *p*-quinol (**1m**) 38
5.20. 4-Phenyl-3-methyl *p*-quinol (**S24**) 39
5.21. Alkyne *p*-quinol (**1n**) 40
5.22. 5-indanol *p*-quinol (**1o**) 41
5.23. Allyl phenol **S19** 42
5.24. Dimethyl allyl quinol (**1p**) 43

6. Synthesis of Enediones 44
6.1. Conditions **A**: Substoichiometric *p*-TsOH·H_2O in $(CH_2Cl)_2$ 44
6.2. Conditions **B**: Stoichiometric *p*-TsOH·H_2O in HFIP 44
6.3. Tethered enedione (**2a**) 45
6.4. Keto-isophorone (**2b**) 46
6.5. 5-Butyl-3,5-dimethyl enedione (**2c**) 47
6.6. 5-Isopropyl 2,4,5,6-tetramethyl enedione (**2d**) 48
6.7. 3,5-Dimethyl 4-phenyl enedione (**2e**) 49
6.8. 3,5-Dimethyl 4-(4-methoxy)phenyl enedione (**2f**) 50
6.9. 3,5-Dimethyl 4-(4-trifluoromethyl)phenyl enedione (**2g**) 51
6.10. Estrone enedione (**2h**) 52
6.11. 5,5-Dimethyl enedione (**2i**) 53
6.12. 5-Isopropyl-5-methylenedione (**2j**) 54
6.13. 2,5,5-Trimethyl enedione (**2k**) 56
6.14. 2,5-Dimethyl 5-isopropyl enedione (**2l**) 57
6.15. 2,5-Dimethyl 5-phenyl enedione (**2m**) 59
6.16. Allyl enedione (**2p**) 61

7. Synthesis of Enedione Derivatives 62

7.1. Diol **6** 62
7.2. Epoxide **7** 63
7.3. Michael adduct **8** 64
7.4. Hydrazone **9** 65
7.5. Cyclopentadiene adduct **10** 66
7.6. Isoprene adduct **11** 67
7.7. Enol acetate **12** 69
7.8. NPM Adduct **13** 70
7.10. DMAD Adduct **14** 71
7.10. PTAD Adduct **15** 72

8. References 74

9. NMR Spectra 76
9.1. Biaryl compounds 76
9.1.1. ^{1}H NMR Spectrum of Compound **S3** (500 MHz, $CDCl_3$): 76
9.1.2. ^{13}C NMR Spectrum of Compound **S3** (126 MHz, $CDCl_3$): 76
9.1.3. ^{1}H NMR Spectrum of Compound **S5** (500 MHz, $CDCl_3$): 77
9.1.4. ^{13}C NMR Spectrum of Compound **S5** (126 MHz, $CDCl_3$): 77
9.1.5. ^{1}H NMR Spectrum of Compound **S7** (500 MHz, $CDCl_3$): 78
9.1.6. ^{13}C NMR Spectrum of Compound **S7** (126 MHz, $CDCl_3$): 78
9.1.7. ^{19}F NMR Spectrum of Compound **S7** (471 MHz, $CDCl_3$): 79
9.2. *p*-Quinols 80
9.2.1. ^{1}H NMR Spectrum of Compound **1a** (500 MHz, $CDCl_3$): 80
9.2.2. ^{13}C NMR Spectrum of Compound **1a** (126 MHz, $CDCl_3$): 80
9.2.3. ^{1}H NMR Spectrum of Compound **1b** (500 MHz, $CDCl_3$): 81
9.2.4. ^{13}C NMR Spectrum of Compound **1b** (126 MHz, $CDCl_3$): 81
9.2.5. ^{1}H NMR Spectrum of Compound **1c** (500 MHz, $CDCl_3$): 82
9.2.6. ^{13}C NMR Spectrum of Compound **1c** (126 MHz, $CDCl_3$): 82
9.2.7. ^{1}H NMR Spectrum of Compound **1d** (500 MHz, $CDCl_3$): 83
9.2.8. ^{13}C NMR Spectrum of Compound **1d** (126 MHz, $CDCl_3$): 83
9.2.9. ^{1}H NMR Spectrum of Compound **1e** (500 MHz, $CDCl_3$): 84
9.2.10. ^{13}C NMR Spectrum of Compound **1e** (126 MHz, $CDCl_3$): 84
9.2.11. ^{1}H NMR Spectrum of Compound **1f** (500 MHz, $CDCl_3$): 85
9.2.12. ^{13}C NMR Spectrum of Compound **1f** (126 MHz, $CDCl_3$): 85

9.2.13. ^{1}H NMR Spectrum of Compound **1g** (500 MHz, $CDCl_3$): 86
9.2.14. ^{13}C NMR Spectrum of Compound **1g** (126 MHz, $CDCl_3$): 86
9.2.15. ^{19}F NMR Spectrum of Compound **1g** (471 MHz, $CDCl_3$): 87
9.2.16. ^{1}H NMR Spectrum of Compound **1h** (500 MHz, CD_3OD): 88
9.2.17. ^{13}C NMR Spectrum of Compound **1h** (126 MHz, CD_3OD): 88
9.2.18. ^{1}H NMR Spectrum of Compound **1i** (500 MHz, $CDCl_3$): 89
9.2.19. ^{13}C NMR Spectrum of Compound **1i** (126 MHz, $CDCl_3$): 89
9.2.20. ^{1}H NMR Spectrum of Compound **1j** (500 MHz, $CDCl_3$): 90
9.2.21. ^{13}C NMR Spectrum of Compound **1j** (126 MHz, $CDCl_3$): 90
9.2.22. ^{1}H NMR Spectrum of Compound **1k** (500 MHz, $CDCl_3$): 91
9.2.23. ^{13}C NMR Spectrum of Compound **1k** (126 MHz, $CDCl_3$): 91
9.2.24. ^{1}H NMR Spectrum of Compound **1l** (500 MHz, $CDCl_3$): 92
9.2.25. ^{13}C NMR Spectrum of Compound **1l** (126 MHz, $CDCl_3$): 92
9.2.26. ^{1}H NMR Spectrum of Compound **1m** (500 MHz, $CDCl_3$): 93
9.2.27. ^{13}C NMR Spectrum of Compound **1m** (126 MHz, $CDCl_3$): 93
9.2.28. ^{1}H NMR Spectrum of Compound **S24** (500 MHz, $CDCl_3$): 94
9.2.29. ^{13}C NMR Spectrum of Compound **S24** (126 MHz, $CDCl_3$): 94
9.2.30. ^{1}H NMR Spectrum of Compound **1n** (500 MHz, $CDCl_3$): 95
9.2.31. ^{13}C NMR Spectrum of Compound **1n** (126 MHz, $CDCl_3$): 95
9.2.32. ^{1}H NMR Spectrum of Compound **1o** (601 MHz, $CDCl_3$) 96
9.2.33. ^{13}C NMR Spectrum of Compound **1o** (151 MHz, $CDCl_3$) 96
9.2.34. ^{1}H NMR Spectrum of Compound **S19** (500 MHz, $CDCl_3$) 97
9.2.35. ^{13}C NMR Spectrum of Compound **S19** (126 MHz, $CDCl_3$) 97
9.2.36. ^{1}H NMR Spectrum of Compound **1p** (601 MHz, $CDCl_3$) 98
9.2.37. ^{13}C NMR Spectrum of Compound **1p** (151 MHz, $CDCl_3$) 98
9.3. Enediones 99
9.3.1. ^{1}H NMR Spectrum of Compound **2a** (500 MHz, $CDCl_3$): 99
9.3.2. ^{13}C NMR Spectrum of Compound **2a** (126 MHz, $CDCl_3$): 100
9.3.3. ^{1}H NMR Spectrum of Keto-Isophorone **2b (**500 MHz, $CDCl_3$): 100
9.3.4. ^{13}C NMR Spectrum of Keto-Isophorone **2b** (126 MHz, $CDCl_3$): 101
9.3.5. ^{1}H NMR Spectrum of Compound **2c** (500 MHz, $CDCl_3$): 101
9.3.6. ^{13}C NMR Spectrum of Compound **2c** (126 MHz, $CDCl_3$): 102
9.3.7. ^{1}H NMR Spectrum of Compound **2d** (500 MHz, $CDCl_3$): 102
9.3.8. ^{13}C NMR Spectrum of Compound **2d** (126 MHz, $CDCl_3$): 103

9.3.9. NOESY Spectrum of Compound **2d** (500 MHz, MeOD, diagonal suppressed): 103
9.3.10. ^{1}H NMR Spectrum of Compound **2e** (601 MHz, $CDCl_3$): 104
9.3.11. ^{13}C NMR Spectrum of Compound **2e** (126 MHz, $CDCl_3$): 104
9.3.12. ^{1}H NMR Spectrum of Compound **2f** (500 MHz, $CDCl_3$): 105
9.3.13. ^{13}C NMR Spectrum of Compound **2f** (126 MHz, $CDCl_3$): 105
9.3.14. ^{1}H NMR Spectrum of Compound **2g** (500 MHz, $CDCl_3$): 106
9.3.15. ^{13}C NMR Spectrum of Compound **2g** (126 MHz, $CDCl_3$): 106
9.3.16. ^{19}F NMR Spectrum of Compound **2g** (471 MHz, $CDCl_3$): 107
9.3.17. ^{1}H NMR Spectrum of Compound **2h** (500 MHz, $CDCl_3$): 108
9.3.18. ^{13}C NMR Spectrum of Compound **2h** (126 MHz, $CDCl_3$): 108
9.3.19. ^{1}H NMR Spectrum of Compound **2i** (500 MHz, $CDCl_3$): 109
9.3.20. ^{13}C NMR Spectrum of Compound **2i** (126 MHz, $CDCl_3$): 109
9.3.21. ^{1}H NMR Spectrum of Compound **2j** (500 MHz, $CDCl_3$): 110
9.3.22. ^{13}C NMR Spectrum of Compound **2j** (126 MHz, $CDCl_3$): 110
9.3.23. ^{1}H NMR Spectrum of Compound **4j** (500 MHz, $CDCl_3$): 111
9.3.24. ^{13}C NMR Spectrum of Compound **4j** (126 MHz, $CDCl_3$): 111
9.3.25. ^{1}H NMR Spectrum of Compound **2k** (500 MHz, $CDCl_3$): 112
9.3.26. ^{13}C NMR Spectrum of Compound **2k** (126 MHz, $CDCl_3$): 112
9.3.27. ^{1}H NMR Spectrum of Compound **2l** (500 MHz, $CDCl_3$): 113
9.3.28. ^{13}C NMR Spectrum of Compound **2l** (126 MHz, $CDCl_3$): 113
9.3.29. ^{1}H NMR Spectrum of Compound **4l** (500 MHz, $CDCl_3$): 114
9.3.30. ^{13}C NMR Spectrum of Compound **4l** (126 MHz, $CDCl_3$): 114
9.3.31. ^{1}H NMR Spectrum of Compound **2m** (500 MHz, $CDCl_3$): 115
9.3.32. ^{13}C NMR Spectrum of Compound **2m** (126 MHz, $CDCl_3$): 115
9.3.33. ^{1}H NMR Spectrum of Compound **4m** (500 MHz, $CDCl_3$): 116
9.3.34. ^{13}C NMR Spectrum of Compound **4m** (126 MHz, $CDCl_3$): 116
9.3.35. ^{1}H NMR Spectrum of Compounds **2p** and **5** (500 MHz, $CDCl_3$): 117
9.3.36. Stacked ^{1}H NMR Spectrum of Compound **1p** and Compounds **2p** and **5** (500 MHz, $CDCl_3$) 117
9.4. Enedione derivatives 118
9.4.1. ^{1}H NMR Spectrum of Compound **6** (500 MHz, $CDCl_3$) 118
9.4.2. ^{13}C NMR Spectrum of Compound **6** (126 MHz, $CDCl_3$) 118
9.4.3. ^{1}H NMR Spectrum of Compound **7** (500 MHz, $CDCl_3$) 119

9.4.4. ^{13}C NMR Spectrum of Compound **7** (126 MHz, CDCl3) 119
9.4.5. ^{1}H NMR Spectrum of Compounds **8** and **S21** (500 MHz, $CDCl_3$).......... 120
9.4.6. ^{13}C NMR Spectrum of Compounds **8** and **S21** (126 MHz, $CDCl_3$) 120
9.4.7. COSY Spectrum of Compounds **8** and **S21** (500 MHz, $CDCl_3$)............ 121
9.4.8. HSQC Spectrum of Compounds **8** and **S21** (500 MHz, $CDCl_3$) 121
9.4.9. HMBC Spectrum of Compounds **8** and **S21** (500 MHz, $CDCl_3$) 122
9.4.10. ^{1}H NMR Spectrum of Compound **9** (500 MHz, $CDCl_3$)........................ 123
9.4.11. ^{13}C NMR Spectrum of Compound **9** (126 MHz, $CDCl_3$) 123
9.4.12. COSY Spectrum of Compound **9** (500 MHz, $CDCl_3$)........................... 124
9.4.13. HSQC Spectrum of Compound **9** (500 MHz, $CDCl_3$)........................... 124
9.4.14. HMBC Spectrum of Compound **9** (500 MHz, $CDCl_3$) 125
9.4.15. NOESY Spectrum of Compound **9** (500 MHz, $CDCl_3$, diagonal suppressed) 125
9.4.16. ^{1}H NMR Spectrum of Compound **10** (500 MHz, $CDCl_3$)...................... 126
9.4.17. CP DA ^{13}C NMR Spectrum of Compound **10** (126 MHz, $CDCl_3$)......... 126
9.4.18. COSY Spectrum of Compound **10** (500 MHz, $CDCl_3$)......................... 127
9.4.19. HSQC Spectrum of Compound **10** (500 MHz, $CDCl_3$)......................... 127
9.4.20. HMBC Spectrum of Compound **10** (500 MHz, $CDCl_3$) 128
9.4.21. NOESY Spectrum of Compound **10** (500 MHz, $CDCl_3$, diagonal suppressed) 128
9.4.22. ^{1}H NMR Spectrum of Compounds **11** and **S22** (500 MHz, $CDCl_3$)...... 129
9.4.23. ^{13}C NMR Spectrum of Compounds **11** and **S22** (126 MHz, $CDCl_3$) 129
9.4.24. COSY Spectrum of Compounds **11** and **S22** (500 MHz, $CDCl_3$)......... 130
9.4.25. HSQC Spectrum of Compounds **11** and **S22** (500 MHz, $CDCl_3$) 130
9.4.26. HMBC Spectrum of Compounds **11** and **S22** (500 MHz, $CDCl_3$) 131
9.4.27. ^{1}H NMR Spectrum of Compound **12** (601 MHz, $CDCl_3$)...................... 132
9.4.28. ^{13}C NMR Spectrum of Compound **12** (151 MHz, $CDCl_3$) 132
9.4.29. ^{1}H NMR Spectrum of Compound **13** (500 MHz, $CDCl_3$)...................... 133
9.4.30. ^{13}C NMR Spectrum of Compound **13** (126 MHz, $CDCl_3$) 133
9.4.31. ^{1}H NMR Spectrum of Compound **14** (500 MHz, $CDCl_3$)...................... 134
9.4.32. ^{13}C NMR Spectrum of Compound **14** (126 MHz, $CDCl_3$) 134
9.4.33. ^{1}H NMR Spectrum of Compound **15** (601 MHz, $CDCl_3$)...................... 135
9.4.34. ^{13}C NMR Spectrum of Compound **15** (126 MHz, $CDCl_3$) 135
9.4.35. ^{1}H NMR Spectrum of Compound **S23** (500 MHz, $CDCl_3$) 136

9.4.36. ^{13}C NMR Spectrum of Compound **S23** (126 MHz, $CDCl_3$) 136

10. X-Ray Data 137

10.1. X-Ray Crystal Structure of **2a**: CCDC Number 2425361: 137

10.2. X-Ray Crystal Structure of **6**: CCDC Number 2425362: 146

10.3. X-Ray Crystal Structure of **13**: CCDC Number 2425364: 160

10.4. X-Ray Crystal Structure of **15**: CCDC Number 2425363: 172

11. Computational Methods ....- 188 -

1. Previously reported quinol–enedione rearrangements

1968 – Davis' Lewis acid-mediated quinol-enedione rearrangement.

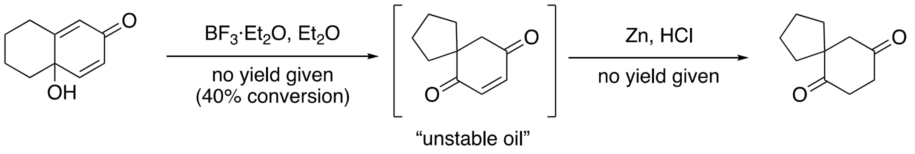

References:
1. Burkinshaw, G. F.; Davis, B. R.; Woodgate P. D.; Hodges R. The Isolation of a Spiran in the Acid-catalysed Rearrangement of a Bicyclic Cyclohexadienone. *Chem. Commun.* **1968**, 528.
2. Burkinshaw, G. F.; Davis, B. R.; Hutchinson, E. G.; Woodgate P. D.; Hodges R. The synthesis and acid-catalysed rearrangements of 4-hydroxycyclohexa-2,5-dienones. *J. Chem. Soc. C* **1971**, 3002–3006.

1976 – Nishinaga & Rieker's Brønsted base-mediated quinol-enedione rearrangement.

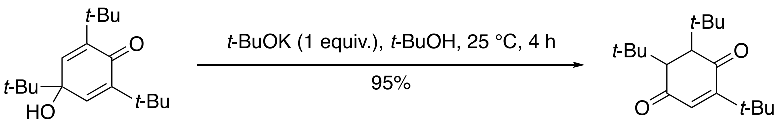

A very specific example of a quinol-enedione rearrangement, where the final aromatization step of the dienone-phenol rearrangement does not occur due to steric repulsion.

Reference:
Berger, S.; Henes, G.; Rieker, A. Baseninduzierte Acyloin-Umlagerung sterisch gehinderter *p*-Chinole. *Chem. Ber.* **1976**, *109*, 1530–1548.

1987 – Bonet's Brønsted acid-mediated quinol-enedione rearrangement.

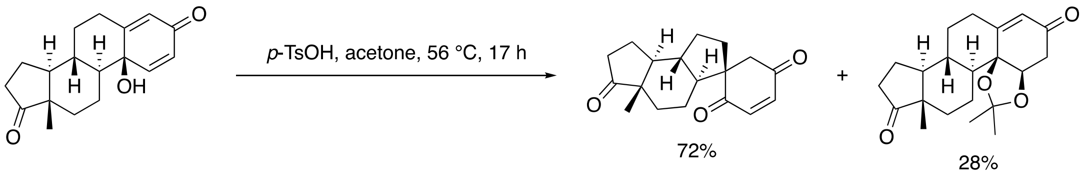

Reference:
Planas, A.; Tomás, J.; Bonet, J.-J. SPIRAN ISOLATION IN THE DIENONE-PHENOL REARRANGEMENT OF STEROIDAL p-QUINOLS. *Tetrahedron Lett.* **1987**, *28*, 471–474.

1991 – Suzuki's Brønsted base-mediated quinol-enedione rearrangement.

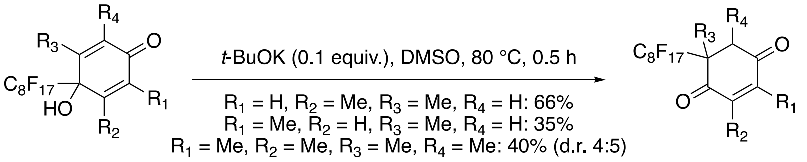

References:
1. Uno, H.; Yayama, A.; Suzuki, H. 1,2-Migration of Perfluoroalkyl Groups in Anionotropic Rearrangement. The Acyloin Rearrangement of 4-Perfluoroalkyl-4-quinols. *Chem. Lett.* **1991**, 1165–1168.
2. Uno, H.; Yayama, A.; Suzuki, H. Perfluoroalkyl Migration in the Rearrangement of 4- Perfluoroalkyl-4-quinols. *Tetrahedron* **1992**, *48*, 8353–8368.

2. General Experimental

Unless otherwise stated, all reactions were carried out using oven-dried glassware under a positive pressure of dry nitrogen using standard Schlenk manifold techniques. All starting materials were bought from commercial suppliers (*e.g.,* Sigma-Aldrich, Fisher Scientific, Alfa Aesar and Acros Organics) and used without further purification unless indicated otherwise. Room temperature (rt) means 18–25 °C and was measured by a temperature probe. Reactions were stirred magnetically using a temperature-regulated hotplate/stirrer and monitored by thin-layer chromatography (TLC) or nuclear magnetic resonance spectroscopy (NMR) where appropriate.

2.1. Thin-Layer Chromatography (TLC)

Thin-layer chromatography (TLC) was performed using aluminum-backed silica plates (Merck Keiselgel 60 F_{254}) and visualized by UV light (λ = 254–312 nm) and/or by staining with *p*-anisaldehyde, potassium permanganate or vanillin solutions, which were subsequently heat-treated.

2.2. Chromatography

Flash column chromatography was carried out using Merck silica gel 60 (40–63 μm, 230–400 mesh).

2.3. Solvents

The following anhydrous solvents were obtained from the University of Edinburgh School of Chemistry's communal solvent purification system, which were purified by filtration through activated alumina columns: acetonitrile (MeCN), dichloromethane (CH_2Cl_2), diethyl ether (Et_2O), tetrahydrofuran (THF) and toluene. These solvents were transferred, stored, and used under a positive pressure of nitrogen in Young's valve-sealed, oven-dried and nitrogen-purged Strauss flasks containing activated 3 Å molecular sieves (~20% w/v). The following anhydrous solvents were purchased and used as received from Acros Organics: 1,2-dichloroethane (DCE) [CAS: 107-06-2], 1,4-dioxane [CAS: 123-91-1], *N*,*N*-dimethylformamide (DMF) [CAS: 68-12-2], 1,1,1,3,3,3-hexafluoro-2-propanol (HFIP) [CAS: 920-66-1], methanol (MeOH) [CAS: 67-56-1] and ethanol [CAS: 64-17-5]. Solvents for non-anhydrous reactions or purifications were used as received.

2.4. Analysis

Nuclear Magnetic Resonance (NMR) data (^{1}H, ^{13}C, ^{19}F, HSQC, HMBC, COSY, NOESY) were recorded using a Bruker Avance 600 MHz, Bruker Avance 500 MHz or Bruker Pro 500 MHz NMR spectrometer. Data were recorded at 298 K unless specified and all ^{13}C NMR spectra were broadband ^{1}H decoupled. Deuterated solvents ($CDCl_3$, CD_3OD) were used as obtained from Sigma-Aldrich. Chemical shifts (δ / ppm) are reported relative to the solvent's reference peaks ($CDCl_3$: ^{1}H - 7.26 ppm; ^{13}C - 77.16 ppm; CD_3OD: ^{1}H - 3.31 ppm, ^{13}C - 49.00). Structural assignments were aided with additional information from ^{1}H-^{1}H COSY, ^{1}H-^{13}C HSQC, ^{1}H-^{13}C HMBC and ^{1}H-^{1}H NOESY experiments.^{19}F NMR spectra were referenced to an external standard (neat $CFCl_3$, δ = 0 ppm). Data for ^{1}H NMR spectra are reported as follows: chemical shift (δ / ppm), peak multiplicity (s, singlet; d, doublet; t, triplet; q, quartet; p, pentet; hept, heptet; m, multiplet or unresolved, br s, broad singlet), integration, coupling constants (Hz) and peak assignment. Coupling constants (*J*) are quoted to the nearest 0.1 Hz.

High Resolution Mass Spectrometry (HRMS) data were recorded by the University of Edinburgh Mass Spectrometry Services Laboratory: EI mass spectra were recorded on a MAT 900 XP double focussing high resolution sector, run at 70 eV. ESI spectra were recorded on a Bruker microTOF, calibrated with sodium formate clusters, with data analysis using Data Analysis 4.1 (Bruker Daltonics).

Fourier Transformed Infra-Red (FTIR) spectra were obtained using a Shimadzu IR Affinity-1 FTIR spectrometer loading compounds as thin films or solids. Select peak values were quoted in wave numbers (cm^{-1}); only characteristic functional group absorption maxima (ν_{max}) are reported.

Optical Rotation ($[\alpha]_D$ values) were recorded using a Bellingham and Stanley Ltd. ADP 450 polarimeter with a Bellingham and Stanley Ltd. 0.5 mL cell (*l* = 0.25 dm). Concentrations (*c*) are reported in g/100 mL.

Melting Points: Melting points were measured using a Bibby Scientific SMP10 melting point apparatus.

3. Optimisation and Control Experiments

3.1 Optimisation of the quinol–enedione rearrangement

3.1.1 Optimisation of tethered *p*-quinol 1a

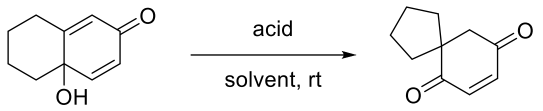

1a
(1.0 equiv.)

2a

Entry	Acid	Equiv.	Solvent	Conv. (%)[A]	Yield (%)[A]
1[B]	-	-	CH_2Cl_2	1	*trace*
2	PPTS	1.00	CH_2Cl_2	10	4
3	TFA	1.00	CH_2Cl_2	14	8
4	aq. HCl (37%)	1.00	CH_2Cl_2	44	21
5	(+)-CSA	1.00	CH_2Cl_2	82	66
6	*p*-TsOH·H_2O	1.00	CH_2Cl_2	97	74
7	*p*-TsOH·H_2O	1.00	Toluene	97	84
8	*p*-TsOH·H_2O	1.00	1,4-Dioxane	18	7
9	*p*-TsOH·H_2O	1.00	CH_3CN	90	76
10	*p*-TsOH·H_2O	1.00	EtOH	98	4
11	*p*-TsOH·H_2O	1.00	$(CH_2Cl)_2$	97	90
12	*p*-TsOH·H_2O	0.20	$(CH_2Cl)_2$	97	93
13	*p*-TsOH·H_2O	0.05	$(CH_2Cl)_2$	96	85
**14**[C]	***p*-TsOH·H_2O**	**0.05**	**$(CH_2Cl)_2$**	**97**	**95**[D,E]
15	*p*-TsOH·H_2O	0.05	HFIP	95	31
16	*p*-TsOH·H_2O	1.00	HFIP	100	0

Reactions conducted on 0.25 mmol scale in solvent (0.035 M). **(A)** Determined by ^1H NMR spectroscopy using 1,4-dinitrobenzene as internal standard; **(B)** Reaction ran at 40 °C; **(C)** Reaction ran at 0.1 M; **(D)** Isolated yield. **(E)** Reaction performed on a 12.2 mmol scale.

3.1.2 Optimisation of trimethyl *p*-quinol 1b

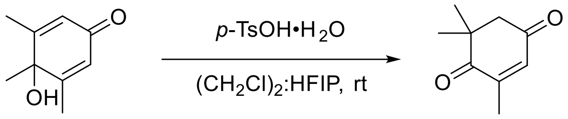

1b
(1.0 equiv.)

2b

Entry	Solvent	HFIP (vol. %)	*p*-TsOH equiv.	Yield (%)[A]
1	$(CH_2Cl)_2$	-	0.05	*trace*
2[B]	$(CH_2Cl)_2$	-	0.05	20
3	$(CH_2Cl)_2$	10	0.05	5
4	-	100	0.05	13
5	$(CH_2Cl)_2$	-	1.00	20
6	$(CH_2Cl)_2$	10	1.00	64
7	$(CH_2Cl)_2$	20	1.00	77
8	$(CH_2Cl)_2$	50	1.00	79
9	-	100	1.00	85
10	-	100	1.00	**73[C,D]**

Reactions conducted on 0.25 mmol scale in solvent (0.1 M). **(A)** Determined by ^1H NMR spectroscopy using 1,4-dinitrobenzene as internal standard; **(B)** Reaction ran at 40 ˚C; **(C)** Isolated yield. **(D)** Reaction performed on a 2.5 mmol scale (for details see Section 6.4).

3.2. Investigation of previously reported conditions by Davis and co-workers[1,2]

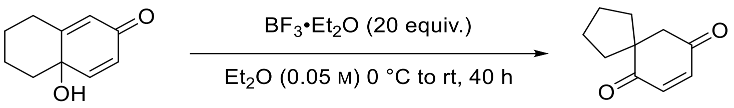

1a **2a** (27% NMR yield)

To an oven-dried round-bottom flask containing a magnetic stirrer bar under an atmosphere of N_2 was charged *p*-quinol **1a** (41 mg, 0.25 mmol, 1.0 equiv.) followed by anhydrous Et_2O (5 mL). The mixture was cooled to 0 °C in an ice-bath after which, BF_3•Et_2O (0.62 mL, 5.0 mmol, 20 equiv.)[A] was added. The reaction was stirred at 0 °C for 5 min before warming to room temperature. Reaction progress was monitored by thin-layer chromatography and ^1H NMR spectroscopy. After 40 h the reaction was diluted with H_2O (10 mL). The layers were separated and the aqueous phase was extracted with Et_2O (2 × 10 mL). The combined organic phases were dried over anhydrous Na_2SO_4, filtered, and concentrated under reduced pressure to afford a crude residue.[B] The yield of **2a** was determined by ^1H NMR spectroscopy using 1,4-dinitrobenzene as internal standard.

Notes: (**A**) BF_3•Et_2O was added in portions (starting with 1.0 equiv.) over 24 h until a total of 20 equiv. had been added. The reaction was stirred for a further 16 h (40 h total) and subsequently worked up. (**B**) Despite several attempts, enedione **2a** could not be separated from several unknown impurities.

3.2.1. ^{1}H NMR Spectrum of crude reaction mixture using Davis' conditions (500 MHz, $CDCl_3$)

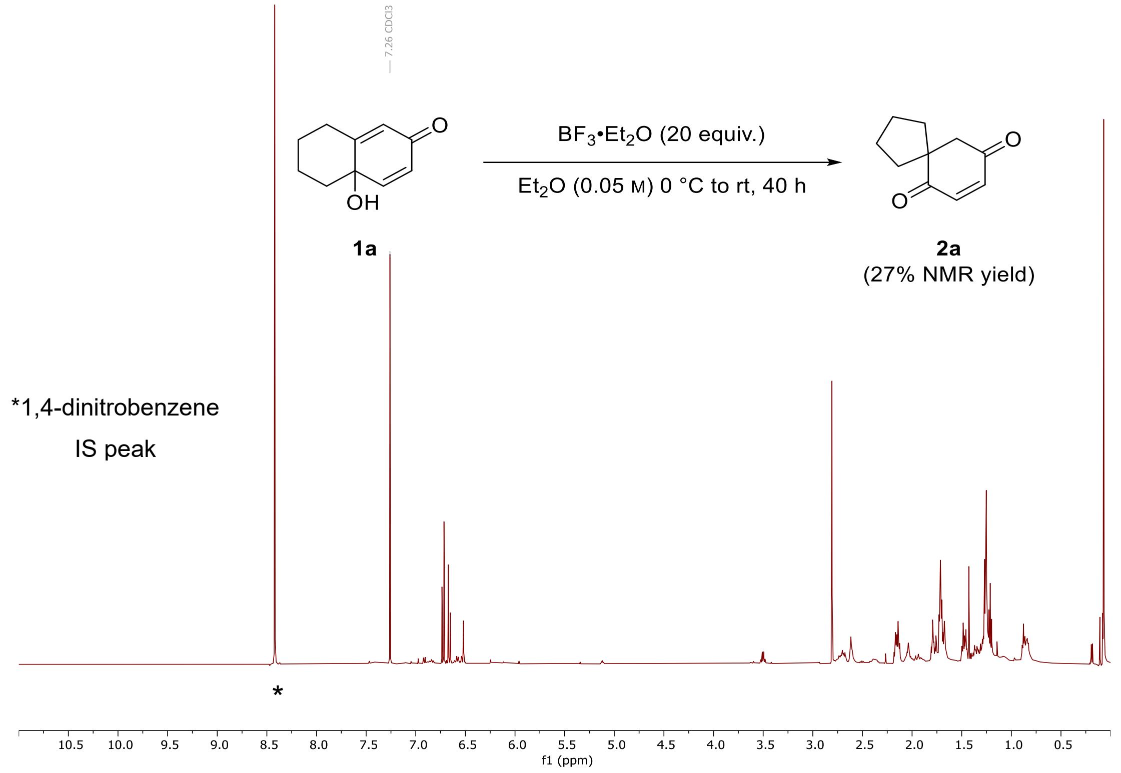

3.2.2. ^{1}H NMR Spectrum of crude reaction mixture using Conditions A (500 MHz, $CDCl_3$)

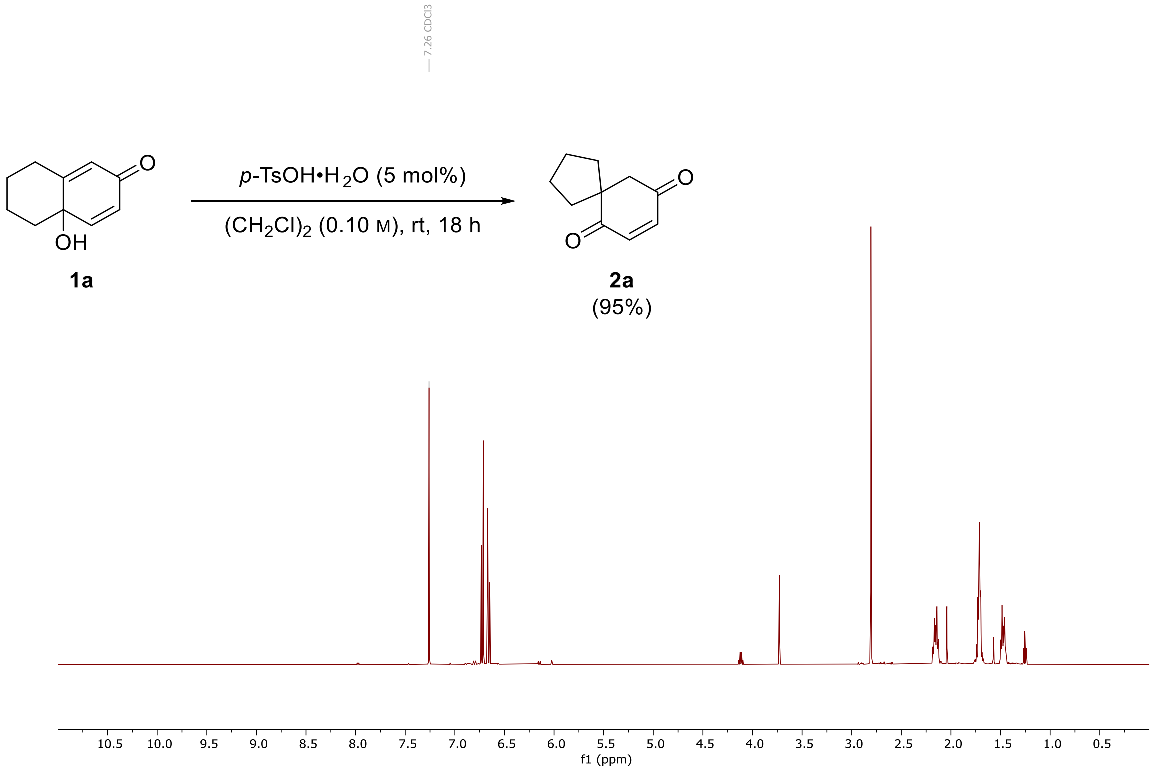

3.3. Reversibility Experiments

To assess the potential reversibility of the quinol–enedione rearrangement, pure samples of enediones **2a** and **2b** were re-subjected to their rearrangement conditions (Conditions **A** and **B** respectively) and the formation of the corresponding *p*-quinols was monitored by ^{1}H NMR spectroscopy using 1,4-dinitrobenzene as internal standard.

Tethered enedione 2a

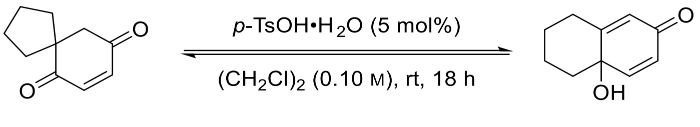

2a **1a**

To an oven-dried 7 mL screw neck sample vial containing a magnetic stirrer bar under an atmosphere of N_2 was charged enedione **2a** (41 mg, 0.25 mmol, 1.0 equiv.) followed by anhydrous 1,2-dichloroethane ($(CH_2Cl)_2$, 2.50 mL, 0.10 M). To this was added *p*-TsOH·H_2O (2 mg, 12.5 μmol, 5 mol%) and the reaction was stirred at room temperature for 18 h. Following this, the reaction was filtered through a short-pad of silica (5 × 5 cm) washing the transferring flask and silica with EtOAc. The filtrate was concentrated under reduced pressure to afford a crude residue.

Keto-isophorone (2b)

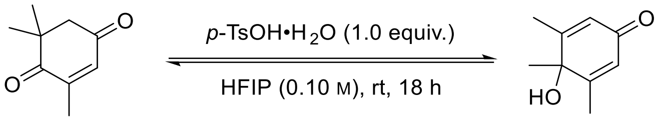

2b **1b**

To an oven-dried 7 mL screw neck sample vial containing a magnetic stirrer bar under an atmosphere of nitrogen was charged keto-isophorone (**2b**) (38 mg, 0.25 mmol, 1.00 equiv.) followed by hexafluoroisopropanol (HFIP, 2.50 mL, 0.10 M). To this was added *p*-TsOH·H_2O (48 mg 0.25 mmol, 1.0 equiv.) and the reaction was stirred at room temperature for 18 h. Following this, the reaction was filtered through a short-pad of silica (5 × 5 cm) washing the transferring flask and silica with EtOAc. The filtrate was concentrated under reduced pressure to afford a crude residue.

3.3.1. ^{1}H NMR Spectra of Tethered Enedione 2a Reversibility

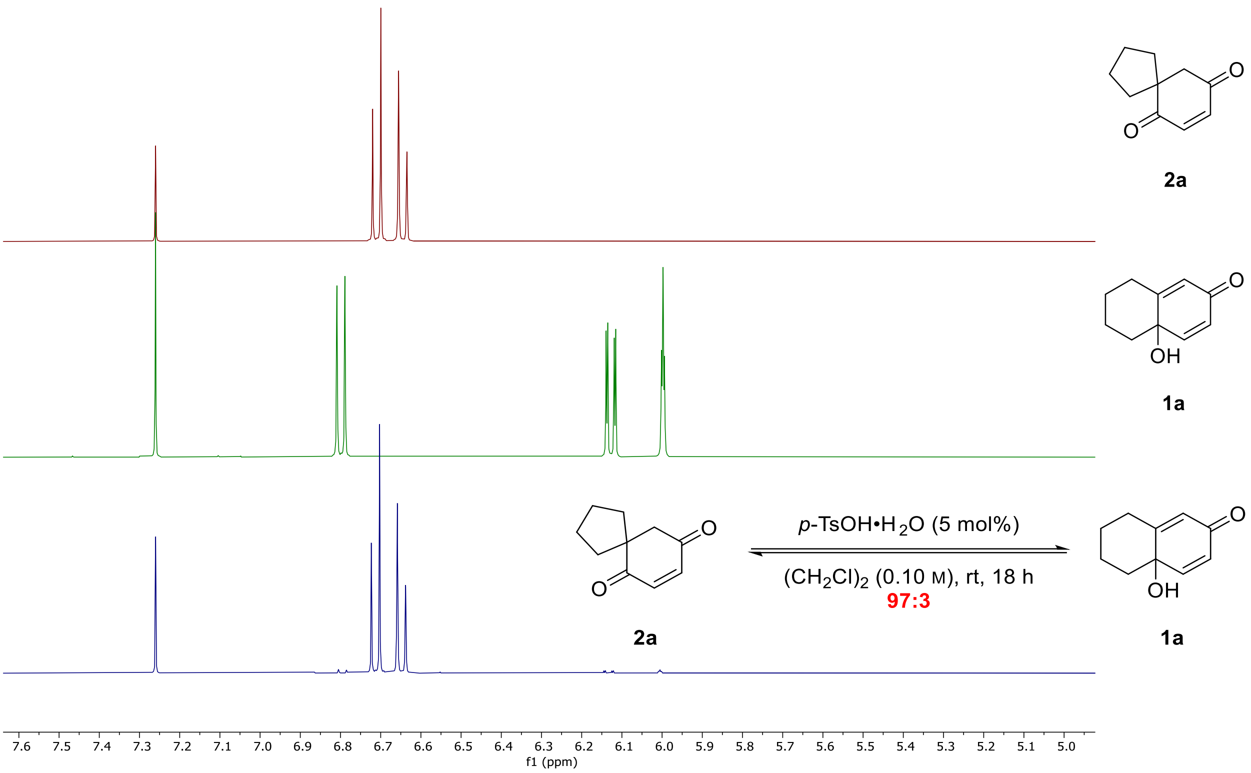

3.3.2. ^{1}H NMR Spectra of Keto-Isophorone (2b) Reversibility

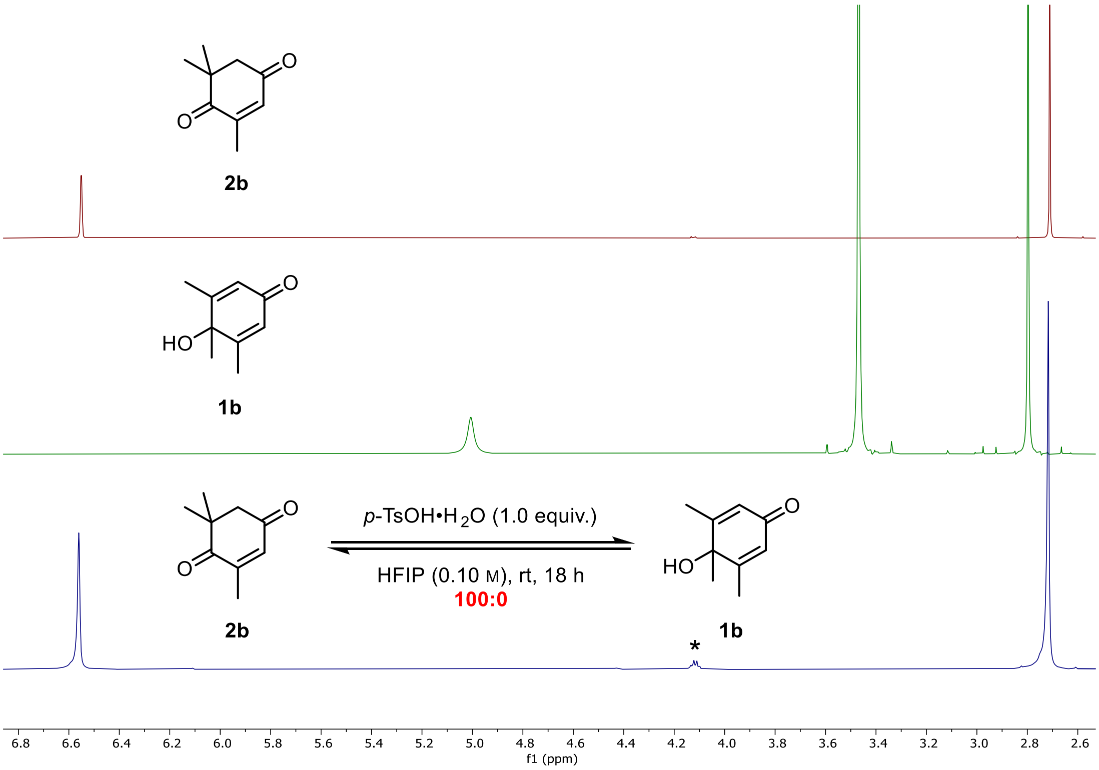

* = Residual EtOAc peak (4.12 ppm)

4. Synthesis of Biphenyl Starting Materials

4.1. General Procedure 1: Synthesis of Biphenyls

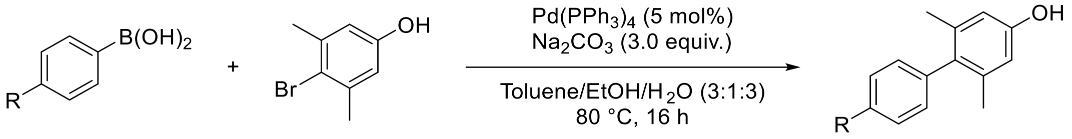

2.0 equiv.   **S1** 1.0 equiv.

Adapted from a procedure outlined by Liu co-workers,[3] To a solution of bromophenol **S1** (500 mg, 2.5 mmol, 1.0 equiv.) in a mixture of toluene/EtOH/H_2O (3:1:3, 14 mL) was added the corresponding boronic acid (5.0 mmol, 2.0 equiv.) and Na_2CO_3 (791 mg, 7.5 mmol, 3.0 equiv.). The suspension was degassed for 15 minutes by sparging using a flow of nitrogen gas. Following this, $Pd(PPh_3)_4$ (144 mg, 0.12 mmol, 5 mol%) was added and the mixture was stirred at 80 ˚C for 16 h. Upon completion, the black reaction mixture was cooled to room temperature and diluted with EtOAc (50 mL) and H_2O (50 mL). The phases were separated and the aqueous phase was extracted with EtOAc (2 × 50 mL). The combined organic phases were washed with brine (100 mL), dried over anhydrous Na_2SO_4, filtered, and concentrated under reduced pressure. The resulting crude residue was purified by flash column chromatography using the conditions stated below to afford the desired phenol.

4.2. Biphenyl S3

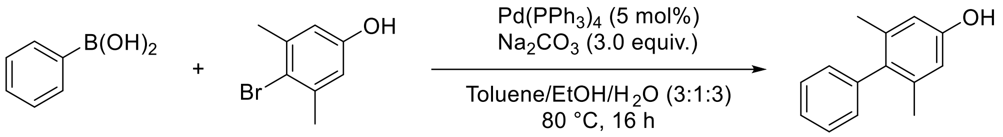

S2 **S1** **S3**
(42%)

Prepared from boronic acid **S2** (606 mg, 5.0 mmol, 2.0 equiv.) according to General Procedure **1**. Flash column chromatography (0–10% EtOAc in *n*-hexane) afforded biphenyl **S3** (207 mg, 1.1 mmol, 42%)[A] as an off-white solid.

Notes: (**A**) If the product is contaminated with the starting material (3,5-dimethyl 4-bromophenol **S1**) it can be carried forward without affecting the yield or purity of the product formed in subsequent steps.

^{1}H NMR (500 MHz, $CDCl_3$): δ = 7.44 – 7.38 (m, 2H), 7.35 – 7.30 (m, 1H), 7.16 – 7.10 (m, 2H), 6.61 – 6.58 (m, 2H), 4.59 (s, 1H), 1.99 (s, 6H);

^{13}C NMR (126 MHz, $CDCl_3$): δ = 154.3, 140.9, 137.9, 134.9, 129.8, 128.5, 126.7, 114.1, 21.1.

Analytical data are consistent with those reported previously in the literature.[4]

4.3. Biphenyl S5

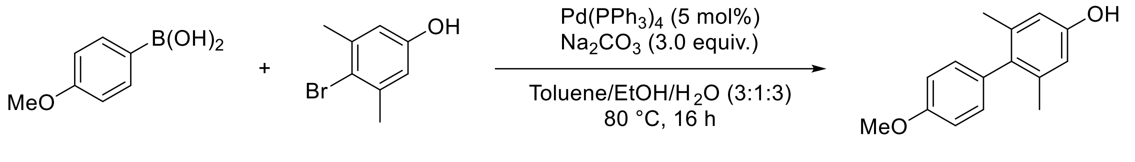

S4 **S1** **S5** (57%)

Prepared from boronic acid **S4** (757 mg, 5.0 mmol, 2.0 equiv.) according to General Procedure **1**. Flash column chromatography (10–30% EtOAc in *n*-hexane) afforded biphenyl **S5** (322 mg, 1.4 mmol, 57%) as an off-white solid.

***R*f** = 0.51 (30% EtOAc in *n*-hexane), stained with $KMnO_4$;

MP: 106–108 °C (*n*-hexane/EtOAc);

^{1}H NMR (500 MHz, $CDCl_3$): δ = 7.06 – 7.00 (m, 2H), 6.99 – 6.91 (m, 2H), 6.59 (s, 2H), 4.56 (app. d, *J* = 1.3 Hz, 1H), 3.85 (s, 3H), 1.99 (s, 6H);

^{13}C NMR (126 MHz, $CDCl_3$): δ = 158.4, 154.2, 138.3, 134.5, 133.1, 130.8, 114.0, 113.9, 55.4, 21.1;

HRMS (ESI$^+$): Calc. for $C_{15}H_{17}O_2$ $[M+H]^+$: 229.1223, found 229.1221;

IR (thin film, cm^{-1}): 3375, 2956, 1607, 1516, 1459, 1307, 1285, 1240, 1150, 1039.

4.4. Biphenyl S7

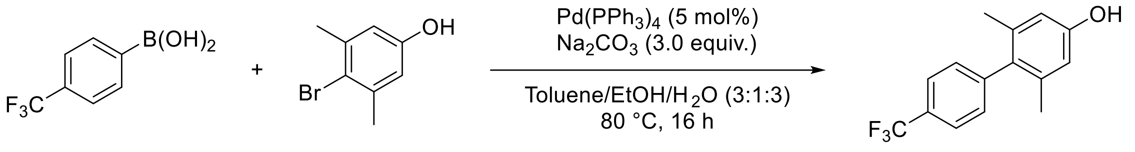

S6 **S1** **S7** (38%)

Prepared from boronic acid **S6** (946 mg, 5.0 mmol, 2.0 equiv.) according to General Procedure **1**. Flash column chromatography (10–30% EtOAc in *n*-hexane) biphenyl **S7** (249 mg, 307 mg total mass recovery, 0.94 mmol, 38%)[A] as a white solid.

Notes: (**A**) The final product was contaminated with the starting material (3,5-dimethyl 4-bromophenol **S1**) in a ratio of 81:19 (**S7**:**S1**) after flash column chromatography. The yield of the **S7** was calculated from the mass of material recovered from flash chromatography (307 mg). It was engaged in the next reaction with PIDA regardless (see 5.11, page 31).

***R*f** = 0.54 (30% EtOAc in *n*-hexane), stained with $KMnO_4$;

^{1}H NMR (500 MHz, $CDCl_3$): δ = 7.68 (d, *J* = 8.0 Hz, 2H), 7.27–7.24 (m, 2H), 6.63 (s, 2H), 4.83 (s, 1H), 1.98 (s, 6H);

^{13}C NMR (126 MHz, $CDCl_3$): δ = 154.7, 139.7, 137.7, 133.4, 130.3, 129.1(q, *J* = 32.2 Hz), 125.5 (q, *J* = 4.1 Hz), 124.5 (q, *J* = 272.4 Hz), 114.3, 21.0;

^{19}F NMR (471 MHz, $CDCl_3$) δ –62.34;

HRMS (ESI$^-$): Calc. for $C_{15}H_{12}F_3O$ [M−H]$^-$: 265.0846, found 265.0841;

IR (thin film, cm^{-1}): 3357, 2928, 1617, 1323, 1153, 1125, 1068.

5. Synthesis of *p*-Quinol Substrates

5.1. General Procedure 2: Synthesis of *p*-quinols using Oxone®

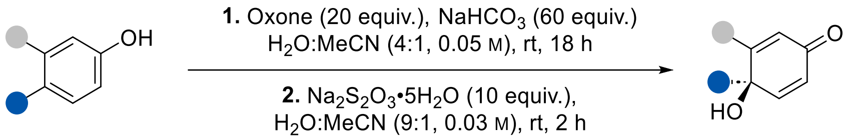

Adapted from a procedure outlined by Carreño co-workers,[5] to a three-neck round-bottom flask with two empty balloons attached to outer necks was charged the corresponding phenol (3.0 mmol, 1.0 equiv.) followed by H_2O:MeCN (4:1, 60 mL, 0.05 M). The resulting solution was cooled to 0 °C and to this was added a pre-ground mixture of Oxone® (18.5 g, 60 mmol, 20 equiv.) and $NaHCO_3$ (15.1 g, 180 mmol, 60 equiv.) quickly before the flask was sealed. The reaction was allowed to stir at this temperature for 15 min before warming to room temperature and stirring for 18 h. Following this, the flask was opened and H_2O (40 mL) was added followed by $Na_2S_2O_3 \cdot 5H_2O$ (7.45 g, 30 mmol, 10 equiv.) and the reaction was left to stir for a further 2 h. The reaction was then transferred to a separating funnel containing water (100 mL) and the solution was extracted with EtOAc (3 × 100 mL). The organic phases were combined, washed with brine (100 mL), dried over anhydrous Na_2SO_4, filtered, and concentrated under reduced pressure. The resulting crude residue was purified by flash column chromatography using the conditions stated below to afford the desired *p*-quinol.

5.2. General Procedure 3: Synthesis of *p*-quinols using PIDA

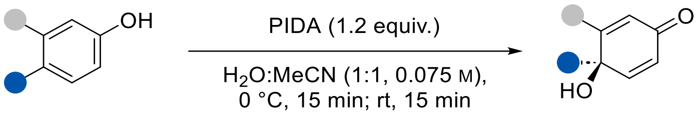

According to a procedure outlined by Baran and co-workers,[6] to a solution of the corresponding phenol (3.0 mmol, 1.0 equiv.) in H_2O:MeCN (1:1, 40 mL, 0.075 M) at 0 °C was added (diacetoxyiodo)benzene (PIDA, 1.16 g, 3.6 mmol, 1.2 equiv.). The reaction was allowed to stir at this temperature for 15 min before warming to room temperature and stirring for a further 15 min. The reaction was then transferred to a separating funnel containing water (100 mL) and the solution was extracted with EtOAc (3 × 100 mL). The organic phases were combined, washed with brine (100 mL), dried over anhydrous Na_2SO_4, filtered, and concentrated under reduced pressure. The resulting crude residue was purified by flash column chromatography using the conditions stated below to afford the desired *p*-quinol.

5.3. General Procedure 4: Synthesis of *p*-quinols using organolithium addition

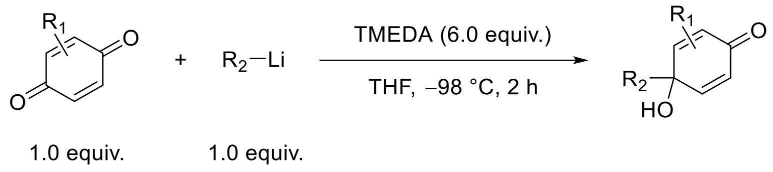

According to a procedure outlined by Liotta and co-workers,[7] to a solution of TMEDA (6.0 equiv.) in THF (0.3 M) was added the corresponding organolithium[A] (1.0 equiv.) at −40 ºC. The resulting solution was taken out of the cold bath for 5 min before being cooled to −40 ºC again. The organolithium/TMEDA solution was then added to a solution of the desired quinone (2.0 or 3.0 mmol,1.0 equiv.) in THF (0.14 M) at –98 ˚C over 10 min. The reaction mixture turned dark green upon addition. The reaction was stirred for 1 h at this temperature and was then warmed to room temperature. Following this, EtOH (5 mL) was added, followed by a saturated solution of aqueous NH_4Cl (20 mL). The reaction mixture was diluted with water (15 mL) and extracted with EtOAc (3 × 50 mL). The combined organic phases were washed with brine (50 mL), dried over anhydrous Na_2SO_4, filtered, and concentrated under reduced pressure. The resulting crude residue was purified by flash column chromatography using the conditions stated below to afford the desired *p*-quinol.

Notes: (**A**) The following organolithium reagents were purchased from commercial suppliers and used as received: *n*-BuLi (1.6 or 2.5 M solution in hexanes), MeLi (1.6 M solution in diethyl ether) and PhLi (1.9 M solution in *n*-butyl ether).

5.4. General Procedure 5: Synthesis of *p*-quinols using Grignard addition

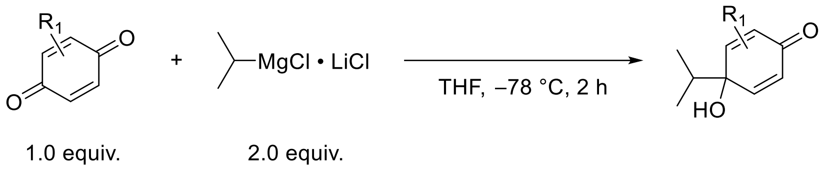

According to a procedure outlined by Liotta and co-workers,[7] *iso*-propylmagnesium chloride lithium chloride complex (1.3 M in THF, 2.0 equiv.) was added to a solution of the desired quinone (2.0 mmol, 1.0 equiv.) in THF (20 mL, 0.1 M) at −78 ºC over 10 min. The reaction mixture turned dark green upon addition. The reaction was stirred at this temperature for 1 h before being warmed to room temperature. Following this, EtOH (5 mL) was added, followed by a saturated aqueous NH_4Cl (20 mL). The reaction mixture was diluted with water (15 mL) and extracted with ethyl acetate (3 × 50 mL). The combined organic phases were washed with brine (50 mL), dried over anhydrous Na_2SO_4, filtered, and concentrated under reduced pressure. The resulting crude residue was purified by flash column chromatography using the conditions stated below to afford the desired *p*-quinol.

5.5. Tethered *p*-quinol (1a)

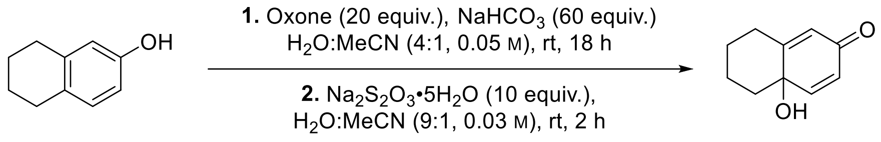

S8

1a
(52% yield)

Prepared from phenol **S8** (2.00 g, 13.6 mmol, 1.0 equiv.) according to General Procedure **2**. Flash column chromatography (50% EtOAc in *n*-hexane) afforded *p*-quinol **1a** (1.16 g, 7.1 mmol, 52%) as a white solid.

$\boldsymbol{R_f}$ = 0.27 (50% EtOAc in *n*-hexane), stained with $KMnO_4$;

^{1}H NMR (500 MHz, $CDCl_3$): δ = 6.80 (d, *J* = 9.9 Hz, 1H), 6.13 (dd, *J* = 9.9, 2.0 Hz, 1H), 6.00 (t, *J* = 1.9 Hz, 1H), 2.70 (tdd, *J* = 13.2, 5.1, 1.8 Hz, 1H), 2.33 (ddt, *J* = 12.9, 4.1, 2.1 Hz, 1H), 2.11 (ddt, *J* = 13.7, 4.2, 2.3 Hz, 1H), 2.06 – 1.97 (m, 2H), 1.93 (tt, *J* = 13.6, 3.9 Hz, 1H), 1.66 (dtt, *J* = 13.5, 4.1, 2.0 Hz, 1H), 1.45 – 1.29 (m, 2H);

^{13}C NMR (126 MHz, $CDCl_3$): δ = 186.5, 164.2, 152.1, 127.8, 123.4, 68.5, 39.2, 32.4, 28.1, 20.4;

Analytical data are consistent with those reported previously in the literature.[5]

5.6. 3,4,5-trimethyl *p*-quinol (1b)

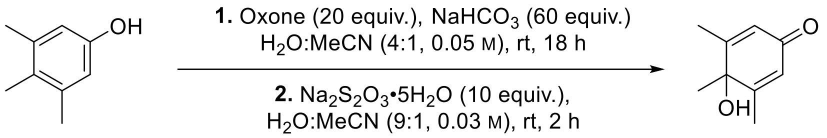

S9

1b
(60% yield)

Prepared from phenol **S9** (1.00 g, 7.3 mmol, 1.0 equiv.) according to General Procedure **2**. Flash column chromatography (50% EtOAc in *n*-hexane) afforded *p*-quinol **1b** (669 mg, 4.4 mmol, 60%) as a white solid.

***R*f** = 0.18 (50% EtOAc in *n*-hexane), stained with $KMnO_4$;

^{1}H NMR (500 MHz, $CDCl_3$): δ = 5.87 – 5.82 (m, 2H), 3.59 (s, 1H), 2.05 (d, *J* = 1.7 Hz, 6H), 1.38 (s, 3H);

^{13}C NMR (126 MHz, $CDCl_3$): δ = 186.2, 164.5, 125.4, 71.5, 26.1, 18.3;

Analytical data are consistent with those reported previously in the literature.[5]

5.7. 4-Butyl 3,5-dimethyl *p*-quinol (1c)

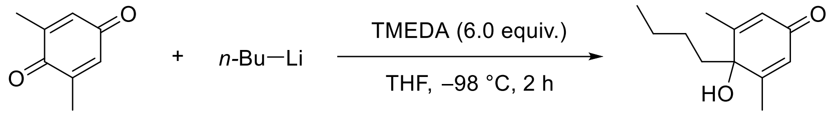

S10 **1c** (15%)

Prepared from quinone **S10** (408 mg, 3.0 mmol, 1.0 equiv.) according to General Procedure **4**. Flash column chromatography (10–30% EtOAc in *n*-hexane) afforded *p*-quinol **1c** (88 mg, 0.45 mmol, 15%) as a brown oil.

***R*f** = 0.09 (20% EtOAc in *n*-hexane), stained with *p*-anisaldehyde;

^{1}H NMR (500 MHz, $CDCl_3$): δ = 6.05 (q, *J* = 1.1 Hz, 2H), 2.09 (s, 1H), 2.04 (d, *J* = 1.1 Hz, 6H), 1.84 – 1.76 (m, 2H), 1.34 – 1.19 (m, 2H), 0.88 – 0.77 (m, 5H);

^{13}C NMR (126 MHz, $CDCl_3$): δ = 186.0, 161.0, 127.8, 74.8, 36.4, 25.5, 22.6, 18.2, 13.9;

HRMS (ESI$^+$): Calc. for $C_{12}H_{19}O_2$ [M+H]$^+$: 195.1380, found: 195.1374;

IR (thin film, cm^{-1}): 3353, 2956, 1660, 1616, 1381, 1317, 1012, 890.

5.8. 4-Isopropyl 2,3,5,6-tetramethyl *p*-quinol (1d)

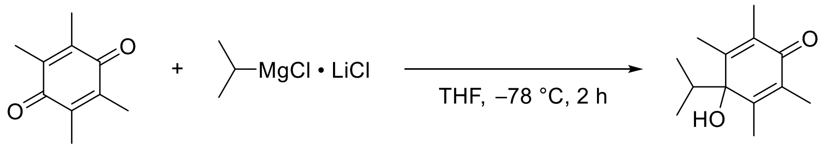

S11 **1d** (72%)

Prepared from duroquinone (**S11**, 328 mg, 2.0 mmol, 1.0 equiv.) according to General Procedure **5**. Flash column chromatography (5–20% EtOAc in *n*-hexane) afforded *p*-quinol **1d** (300 mg, 1.4 mmol, 72%) as a beige oil.

$\boldsymbol{R_f}$ = 0.23 (20% EtOAc in *n*-hexane), stained with $KMnO_4$;

^{1}H NMR (500 MHz, $CDCl_3$): δ = 2.07 (hept, *J* = 6.9 Hz, 1H), 1.98 (q, *J* = 1.1 Hz, 6H), 1.84 (q, *J* = 1.1 Hz, 6H), 0.82 (d, *J* = 6.9 Hz, 6H);

^{13}C NMR (126 MHz, $CDCl_3$): δ = 185.3, 153.7, 132.2, 77.9, 36.6, 17.8, 16.3, 11.5;

HRMS (ESI$^+$): Calc. for $C_{13}H_{20}O_2Na$ $[M+Na]^+$: 231.1356, found: 231.1353;

IR (thin film, cm^{-1}): 3409, 2966, 1659, 1612, 1455, 1373, 1259, 1027.

5.9. 3,5-Dimethyl 4-phenyl *p*-quinol (1e)

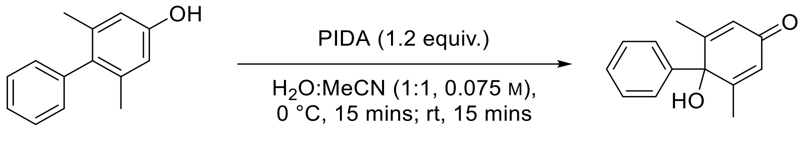

Prepared from phenol **S3** (198 mg, 1.0 mmol, 1.0 equiv.) according to General Procedure **3**. Flash column chromatography (10–30% EtOAc in *n*-hexane) afforded *p*-quinol **1e** (126 mg, 0.63 mmol, 63%) as a yellow-orange oil.

$\boldsymbol{R_f}$ = 0.29 (30% EtOAc in *n*-hexane), stained with vanillin;

^{1}H NMR (500 MHz, $CDCl_3$): δ 7.41–7.32 (m, 4H), 7.31–7.27 (m, 1H), 6.09 – 6.07 (m, 2H), 2.41 (s, 1H), 1.78 (d, *J* = 1.3 Hz, 6H);

^{13}C NMR (126 MHz, $CDCl_3$): δ 186.5, 161.7, 138.9, 128.8, 128.0, 126.1, 125.1, 75.7, 18.4;

HRMS (ESI$^+$): Calc. for $C_{14}H_{15}O_2$ $[M+H]^+$: 215.1067, found: 215.1065;

IR (thin film, cm^{-1}): 3356, 2960, 1668, 1620, 1378, 1167, 931, 761, 700.

5.10. 3,5-Dimethyl 4-(4-methoxyphenyl) *p*-quinol (1f)

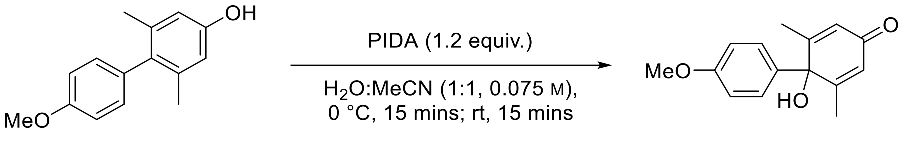

S5 **1f** (42%)

Prepared from phenol **S5** (228 mg, 1.0 mmol, 1.0 equiv.) according to General Procedure **3**. Flash column chromatography (30–40% EtOAc in *n*-hexane) afforded *p*-quinol **1f** (104 mg, 0.42 mmol, 42%) as a yellow-orange solid.

$\boldsymbol{R}_\mathbf{f}$ = 0.16 (30% EtOAc in *n*-hexane), stained with *p*-anisaldehyde;

MP: 143–147 °C (*n*-hexane/EtOAc);

^{1}H NMR (500 MHz, $CDCl_3$): δ = 7.31–7.26 (m, 2H), 6.89–6.85 (m, 2H), 6.04 (d, *J* = 1.6 Hz, 2H), 3.80 (s, 3H), 2.54 (s, 1H), 1.78 (d, *J* = 1.1 Hz, 6H);

^{13}C NMR (126 MHz, $CDCl_3$): δ = 186.6, 162.2, 159.3, 130.8, 126.4, 125.8, 114.1, 75.4, 55.4, 18.4;

HRMS (ESI$^+$): Calc. for $C_{15}H_{17}O_3$ $[M+H]^+$: 245.1172, found 245.1167;

IR (thin film, cm^{-1}): 3366, 2910, 1667, 1620, 1608, 1507, 1379, 1299, 1249, 1166.

5.11. 3,5-Dimethyl 4-(4-trifluoromethylphenyl) *p*-quinol (1g)

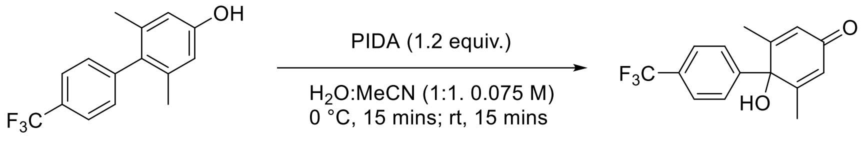

Prepared from phenol **S7**[A] (215 mg of **S7**, 266 mg total mass, 0.81 mmol, 1.0 equiv.) according to General Procedure **3**. Flash column chromatography (40% EtOAc in *n*-hexane) afforded *p*-quinol **1g** (73 mg, 0.26 mmol, 33%) as an orange solid.

Notes (**A**): Starting material **S7** was contaminated with bromophenol **S1** (see 4.4, page 20). The mass of the **S7** was calculated from total mass of impure starting material based on ^{1}H NMR yields.

***R*f** = 0.19 (30% EtOAc in *n*-hexane), stained with *p*-anisaldehyde;

^{1}H NMR (500 MHz, $CDCl_3$): δ = 7.65 – 7.59 (app. m, 2H), 7.52 (d, *J* = 8.1 Hz, 2H), 6.14 (app. p, *J* = 0.8 Hz, 2H), 2.21 (d, *J* = 0.7 Hz, 1H), 1.77 (d, *J* = 1.1 Hz, 6H);

^{13}C NMR (126 MHz, $CDCl_3$): δ = 185.9, 160.4, 143.2, 130.4 (q, *J* = 32.7 Hz), 128.7, 126.7, 125.9–125.7 (m), 124.1 (q, *J* = 271.8 Hz), 75.5, 18.3;

^{19}F NMR (471 MHz, $CDCl_3$) δ –62.60;

HRMS (ESI$^-$): Calc. for $C_{15}H_{12}F_3O_2$ [M–H]$^-$: 281.0795, found 281.0797;

IR (thin film, cm^{-1}): 3353, 2962, 2359, 1674, 1630, 1326, 1162, 1128, 1068, 1018.

5.12. Estrone *p*-quinol (1h)

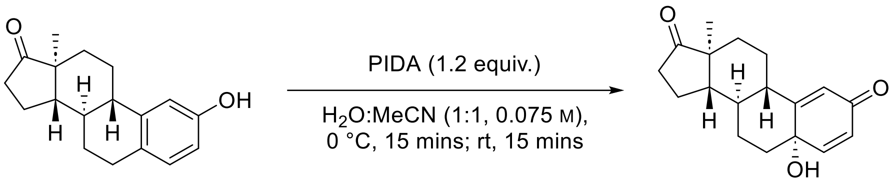

Prepared from estrone (**S12**) (811 mg, 3.0 mmol, 1.0 equiv.) according to a slightly modified General Procedure **3**. CH_2Cl_2 (50 mL) was added to the crude residue and the resulting insoluble precipitate produced was filtered off. The filtrate was concentrated under reduced pressure to give a crude oil that was purified by flash column chromatography (0–30% acetone in *n*-hexane)[A] to afford the *p*-quinol **1h** (70 mg, >95:5 d.r., 0.24 mmol, 8%)[B] as a white solid.

Notes: (**A**) Two rounds of flash column chromatography (0–30% acetone in *n*-hexane) were often required to fully remove impurities from the desired *p*-quinol. (**B**) On 15 mmol scale (4.06 g of estrone) the reaction afforded *p*-quinol **1h** (184 mg, >95:5 d.r., 0.64 mmol, 4%) as a white solid.

***R*f** = 0.28 (30% acetone in *n*-hexane), stained with *p*-anisaldehyde;

^{1}H NMR (500 MHz, CD_3OD) δ = 7.21 (d, *J* = 10.2 Hz, 1H), 6.13 (dd, *J* = 10.2, 2.0 Hz, 1H), 5.98 (dd, *J* = 1.8, 1.8 Hz, 1H), 2.82 (dddd, *J* = 13.6, 12.5, 5.3, 1.7 Hz, 1H), 2.47 (dd, *J* = 9.0, 1.0 Hz, 1H), 2.39 (ddd, *J* = 12.5, 4.2, 2.3 Hz, 1H), 2.20–1.94 (m, 6H), 1.84–1.75 (m, 2H), 1.64 (app. tt, *J* = 12.4, 9.0 Hz, 1H), 1.38–1.21 (m, 2H), 1.20–1.09 (m, 2H), 0.97 (s, 3H);

^{13}C NMR (126 MHz, CD_3OD) δ = 223.1, 188.1, 169.8, 154.6, 127.9, 123.0, 70.9, 56.4, 51.3, 49.1, 36.5, 35.8, 33.7, 33.0, 32.3, 23.2, 23.0, 14.1;

HRMS (ESI$^+$): Calculated for $C_{18}H_{22}O_3Na$ $[M+Na]^+$: 309.1461, found: 309.1461;

IR (thin film, cm^{-1}): 3408, 2940, 2857, 1736, 1667, 1624, 1609, 1403, 1328, 1243, 1172, 1091, 1068, 1051, 985, 889, 825, 808 and 718.

Analytical data are consistent with those reported previously in the literature.[8–10]

5.15. 3,4-Dimethyl *p*-quinol (1i)

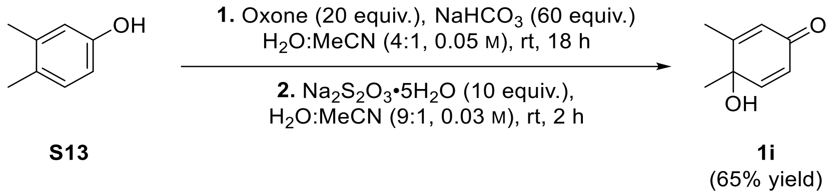

Prepared from phenol **S13** (500 mg, 4.0 mmol, 1.0 equiv.) according to General Procedure **2**. Flash column chromatography (30–40% EtOAc in *n*-hexane) afforded *p*-quinol **1i** (365 mg, 2.7 mmol, 65%) as an off-white solid.

^{1}H NMR (500 MHz, $CDCl_3$): δ = 6.87 (d, *J* = 9.9 Hz, 1H), 6.08 (dd, *J* = 9.9, 1.9 Hz, 1H), 5.97 (dt, *J* = 2.8, 1.5 Hz, 1H), 2.37 (m, 1H), 2.08 (d, *J* = 1.4 Hz, 3H), 1.44 (s, 3H);

^{13}C NMR (126 MHz, $CDCl_3$): δ = 186.1, 162.3, 153.0, 126.9, 125.9, 69.3, 26.2, 18.2.

Analytical data are consistent with those reported previously in the literature.[5]

5.16. 4-Isopropyl 3-methyl *p*-quinol (1j)

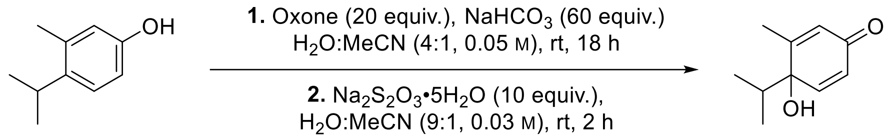

S14

1j
(16% yield)

Prepared from phenol **S14** (600 mg, 4.0 mmol, 1.0 equiv.) according to General Procedure **2**. Flash column chromatography (30–40% EtOAc in *n*-hexane) afforded *p*-quinol **1j** (102 mg, 0.62 mmol, 16%) as a white solid.[A]

Notes: (**A**) Isolated with minor impurities.

^{1}H NMR (500 MHz, $CDCl_3$): δ = 6.86 (d, *J* = 10.2 Hz, 1H), 6.23 (dd, *J* = 10.2, 2.0 Hz, 1H), 6.06 (dq, *J* = 2.8, 1.3 Hz, 1H), 2.14 (app. p, *J* = 6.9 Hz, 2H), 2.02 (d, *J* = 1.4 Hz, 3H), 1.14 (d, *J* = 6.9 Hz, 3H), 0.67 (d, *J* = 6.9 Hz, 3H);

^{13}C NMR (126 MHz, $CDCl_3$): δ = 185.9, 162.0, 148.6, 129.8, 127.7, 74.8, 35.2, 18.0, 16.9, 16.8.

Analytical data are consistent with those reported previously in the literature.[11]

5.17. 2,4,5-Trimethyl *p*-quinol (1k)

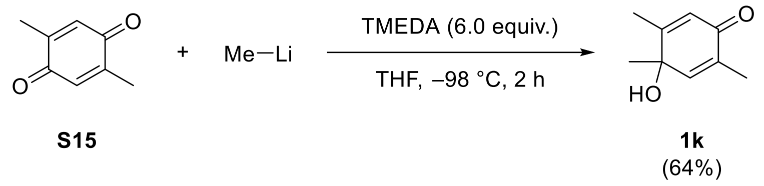

Prepared from quinone **S15** (275 mg, 2.0 mmol, 1.0 equiv.) according to General Procedure **4**. Flash column chromatography (10–20% EtOAc in *n*-hexane) afforded *p*-quinol **1k** (194 mg, 1.3 mmol, 64%) as a beige solid.

R_f = 0.22 (30% EtOAc in *n*-hexane), stained with vanillin;

MP: 110–114 °C (*n*-hexane/EtOAc);

^{1}H NMR (500 MHz, $CDCl_3$): δ 6.63 (q, *J* = 1.5 Hz, 1H), 5.96 (q, *J* = 1.4 Hz, 1H), 2.12 (s, 1H), 2.05 (d, *J* = 1.4 Hz, 3H), 1.84 (d, *J* = 1.4 Hz, 3H), 1.41 (s, 3H);

^{13}C NMR (126 MHz, $CDCl_3$): δ 186.7, 161.8, 148.4, 133.4, 125.7, 69.5, 26.3, 17.9, 15.3;

HRMS (ESI$^+$): Calc. for $C_9H_{12}O_2Na$ $[M+Na]^+$: 175.0730, found: 175.0729;

IR (thin film, cm^{-1}): 3409, 2980, 1671, 1623, 1389, 1373, 1264, 1049, 1027, 895;

5.18. 2,5-Dimethyl 4-isopropyl *p*-quinol (1l)

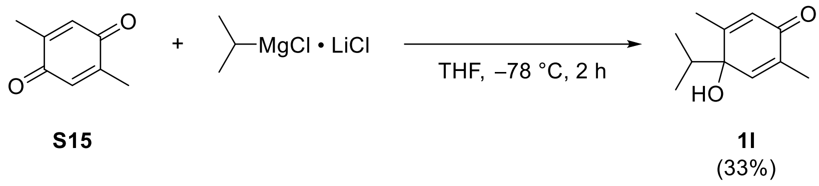

Prepared from quinone **S15** (275 mg, 2.0 mmol, 1.0 equiv.) according to General Procedure **5**. Flash column chromatography (5–20% EtOAc in *n*-hexane) afforded *p*-quinol **1l** (119 mg, 0.66 mmol, 33%) as a beige solid.

R_f = 0.32 (30% EtOAc in *n*-hexane), stained with $KMnO_4$;

MP: 99–102 °C (*n*-hexane/EtOAc);

^{1}H NMR (500 MHz, $CDCl_3$): δ = 6.63 (q, *J* = 1.5 Hz, 1H), 6.06 (q, *J* = 1.4 Hz, 1H), 2.10 (app. hept, *J* = 6.9 Hz, 1H), 1.99 (d, *J* = 1.4 Hz, 3H), 1.90 (d, *J* = 1.4 Hz, 3H), 1.13 (d, *J* = 6.9 Hz, 3H), 0.65 (d, *J* = 6.9 Hz, 3H);

^{13}C NMR (126 MHz, $CDCl_3$): δ = 186.4, 161.3, 143.3, 136.5, 127.6, 74.9, 35.2, 17.7, 17.1, 16.8, 15.7;

HRMS (EI$^+$): Calculated for $C_{11}H_{16}O_2$ $[M]^+$: 180.1145, found: 180.1138;

IR (thin film, cm^{-1}): 3392, 2963, 2877, 1672, 1657, 1375, 1033, 993.

5.19. 2,5-Dimethyl 4-phenyl *p*-quinol (1m)

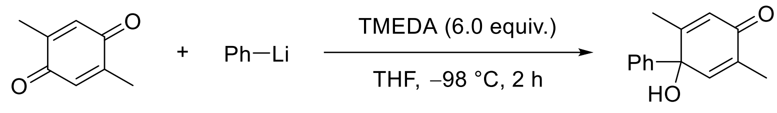

S15

1m
(20%)

Prepared from quinone **S15** (275 mg, 2.0 mmol, 1.0 equiv.) according to General Procedure **4**. Flash column chromatography (5–20% EtOAc in *n*-hexane) afforded *p*-quinol **1m** (86 mg, 0.40 mmol, 20%) as a beige solid.

R_f = 0.41 (30% EtOAc in *n*-hexane), stained with $KMnO_4$;

MP: 103–104 °C (*n*-hexane/EtOAc);

^{1}H NMR (500 MHz, $CDCl_3$): δ 7.41–7.33 (m, 4H), 7.32–7.28 (m, 1H), 6.59 (q, *J* = 1.5 Hz, 1H), 6.14 (q, *J* = 1.5 Hz, 1H), 2.23 (m, 1H), 1.88 (d, *J* = 1.6 Hz, 3H), 1.81 (d, *J* = 1.4 Hz, 3H);

^{13}C NMR (126 MHz, $CDCl_3$): δ 187.1, 160.6, 147.0, 139.5, 132.8, 128.9, 128.0, 126.6, 125.3, 73.8, 18.3, 15.3;

HRMS (ESI$^+$): Calc. for $C_{14}H_{15}O_2$ [M+H]$^+$: 215.1067, found: 215.1068;

IR (thin film, cm^{-1}): 3417, 2924, 2925, 1671, 1647, 1488, 1448, 1376, 1152, 993, 929.

5.20. 4-Phenyl-3-methyl *p*-quinol (S24)

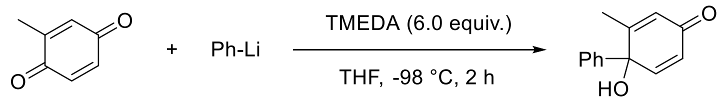

S24
(7%)

Prepared from methyl-*p*-benzoquinone (366 mg, 3.0 mmol, 1.0 equiv.) according to General Procedure **4**. Flash column chromatography (5–20% EtOAc in *n*-hexane) afforded *p*-quinol **S24** (42 mg, 0.21 mmol, 7%) as a beige semi-solid.[A]

Notes: (**A**) Attempts to rearrange quinol **S24** using Conditions **B** resulted in complete degradation.

R_f = 0.32 (30% EtOAc in *n*-hexane), stained with $KMnO_4$;

^{1}H NMR (500 MHz, $CDCl_3$): δ 7.50 – 7.45 (m, 2H), 7.40 – 7.35 (m, 2H), 7.34 – 7.29 (m, 1H), 6.87 (dd, *J* = 9.9, 3.1 Hz, 1H), 6.68 (dq, *J* = 3.1, 1.5 Hz, 1H), 6.21 (d, *J* = 9.9 Hz, 1H), 2.61 (s, 1H), 1.91 (d, *J* = 1.5 Hz, 3H);

^{13}C NMR (126 MHz, $CDCl_3$): δ 186.6, 150.8, 146.5, 139.6, 133.7, 129.0, 128.3, 127.0, 125.4, 71.5, 15.7.

Analytical data are consistent with those reported previously in the literature.[12]

5.21. Alkyne *p*-quinol (1n)

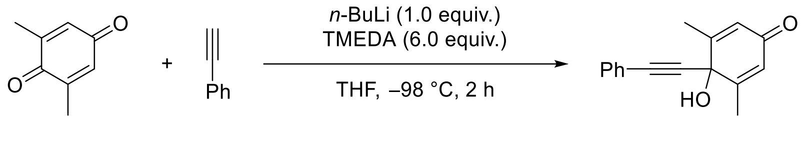

S10 **S16** **1n** (63% yield)

Prepared according to a modified General Procedure **4**. To a solution of phenylacetylene (**S16**) (0.33 mL, 3.0 mmol, 1.0 equiv.) in anhydrous THF (10 mL, 0.3 M) at –40 ˚C was added *n*-BuLi (1.6 M in hexanes, 3.0 mmol, 1.0 equiv.). The resulting solution was stirred for 5 min. The mixture was taken out of the cold bath and allowed to warm back up to room temperature before being cooled to −40 ºC again after which, TMEDA (2.7 mL, 18 mmol, 6.0 equiv.) was added. The resulting organolithium/TMEDA solution was then added to a solution of quinone **S10** (408 mg, 3.0 mmol, 1.0 equiv.) in THF (20 mL, 0.15 M) at –98 ˚C over 10 min. The resulting dark green mixture was stirred for 2 h at this temperature and was then warmed to room temperature. Following this, EtOH (10 mL) was added, followed by a saturated solution of NH_4Cl (30 mL). The reaction mixture was diluted with water (20 mL) and extracted with EtOAc (3 × 30 mL). The combined organic phases were washed with brine (50 mL), dried over anhydrous Na_2SO_4, filtered, and concentrated under reduced pressure. The resulting crude residue was purified by flash column chromatography (10–30% EtOAc in *n*-hexane) to afford *p*-quinol **1n** (454 mg, 1.9 mmol, 63% yield).[A,B]

Notes: (**A**) Attempts to rearrange quinol **1n** under Conditions **A** resulted in no reaction. (**B**) Attempts to rearrange quinol **1n** under Conditions **B** resulted in complete degradation.

$\boldsymbol{R_f}$ = 0.31 (50% EtOAc in *n*-hexane), stained with $KMnO_4$;

^{1}H NMR (601 MHz, $CDCl_3$) δ = 7.46 – 7.41 (m, 2H), 7.39 – 7.30 (m, 3H), 6.06 (m, 2H), 2.68 (s, 1H), 2.28 (d, *J* = 1.2 Hz, 6H);

^{13}C NMR (151 MHz, $CDCl_3$) δ = 185.5, 157.1, 132.1, 129.4, 128.6, 125.8, 121.5, 86.8, 85.9, 68.1, 18.9;

HRMS (ESI$^+$): Calculated for $C_{16}H_{15}O_2$ [M+H]$^+$: 239.1067, found: 239.1065

IR (thin film, cm^{-1}): 3377, 2227, 1671, 1618, 1019, 758 and 691.

5.22. 5-indanol *p*-quinol (1o)

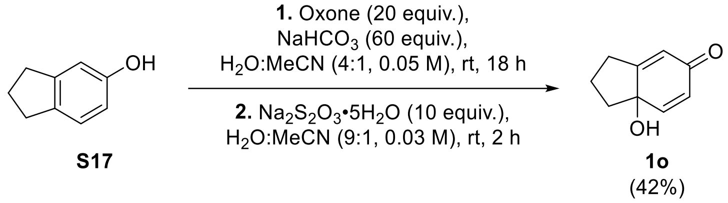

Prepared from 5-indanol (**S17**, 500 mg, 3.8 mmol, 1.0 equiv.) according to General Procedure **2**. Flash column chromatography (0–30% acetone in *n*-hexane) afforded *p*-quinol **1o** (235 mg, 1.6 mmol, 42%) as a light-yellow solid.[A,B]

Notes: (**A**) Attempts to rearrange quinol **1o** under Conditions **A** resulted in no reaction. (**B**) Attempts to rearrange quinol **1o** under Conditions **B** resulted in complete degradation.

R_f = 0.34 (30% acetone in *n*-hexane), stained with *p*-anisaldehyde;

^{1}H NMR (601 MHz, $CDCl_3$) δ = 7.01 (d, *J* = 9.9, Hz, 1H), 6.11 (dd, *J* = 9.9, 1.7 Hz, 1H), 6.02–5.98 (m, 1H), 2.95 – 2.86 (m, 1H), 2.54 – 2.45 (m, 1H), 2.30 – 2.19 (m, 1H), 2.08 (ddt, *J* = 13.5, 7.8, 1.3 Hz, 1H), 1.96 (dtdd, *J* = 12.9, 9.4, 4.6, 1.5 Hz, 1H), 1.81 (s, 1H), 1.67 – 1.59 (m, 1H);

^{13}C NMR (151 MHz, $CDCl_3$) δ = 186.5, 169.0, 147.1, 128.9, 122.3, 73.7, 35.6, 28.6, 21.6;

HRMS (ESI$^+$): Calculated for $C_9H_{10}O_2Na$ $[M+Na]^+$: 173.0573, found: 173.0567;

IR (thin film, cm^{-1}): 3355, 2959, 2923, 2851, 1669, 1632, 1607, 1426, 1388, 1295, 1166, 1127, 1087, 1048, 966, 893, 867, 814, 782, 749, 703, 699 and 600.

5.23. Allyl phenol S19

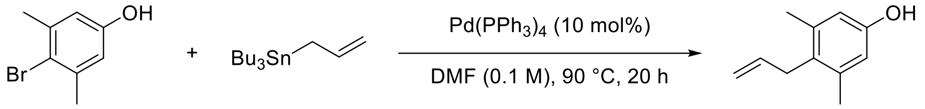

S1 **S18** **S19** (71%)

To an oven-dried round-bottom flask under an atmosphere of N_2 containing a magnetic stirrer bar was added bromo phenol **S1** (800 mg, 4.0 mmol, 1.0 equiv.). Anhydrous DMF (40 mL) was added and the resulting mixture was degassed by sparging with a flow of N_2 gas for 10 min. Allyl tributyltin (**S18**, 1.96 mL, 6.0 mmol, 1.50 equiv.) was added followed by $Pd(PPh_3)_4$ (460 mg, 0.4 mmol, 10 mol%). The reaction mixture was warmed to 90 °C and stirred at this temperature for 20 h. Upon completion, the mixture was cooled to room temperature and a saturated solution of NH_4Cl (40 mL) was added followed by EtOAc (80 mL). The layers were allowed to separate and the mixture was extracted with EtOAc (2 × 80 mL). The combined organic phases were washed with brine (3 × 100 mL), dried over anhydrous Na_2SO_4, filtered and concentrated under reduced pressure to afford a crude residue. Flash column chromatography (5–10% EtOAc in *n*-hexane) afforded phenol **S19** (456 mg, 2.8 mmol, 71%) as a colourless oil.[A]

Notes: (**A**) If the product was contaminated with organotin reagent **S18**, additional extractions using MeCN/*n*-hexane were carried out to afford pure product.

***R*f** = 0.32 (10% EtOAc in *n*-hexane), stained with $KMnO_4$;

^{1}H NMR (500 MHz, $CDCl_3$) δ = 6.52 (s, 2H), 5.88 (ddt, *J* = 17.1, 10.1, 5.6 Hz, 1H), 4.97 (dq, *J* = 10.1, 1.8 Hz, 1H), 4.84 (dq, *J* = 17.1, 1.9 Hz, 1H), 4.47 (s, 1H), 3.32 (dt, *J* = 5.6, 1.9 Hz, 2H), 2.24 (s, 6H);

^{13}C NMR (126 MHz, $CDCl_3$) δ = 153.4, 138.4, 135.8, 128.6, 114.8, 114.7, 33.0, 20.1.

Analytical data are consistent with those reported previously in the literature.[13]

5.24. Dimethyl allyl quinol (1p)

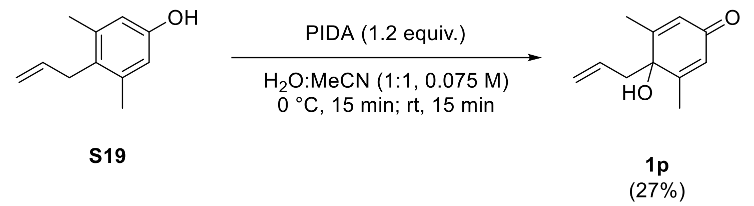

Prepared from phenol **S19** (456 mg, 2.8 mmol, 1.0 equiv.) according to General Procedure **3**. Flash column chromatography (20–50% EtOAc in *n*-hexane) afforded *p*-quinol **1p** (134 mg, 0.75 mmol, 27%) as a beige solid.

***R*$_f$** = 0.23 (50% EtOAc in *n*-hexane), stained with $KMnO_4$;

^{1}H NMR (601 MHz, $CDCl_3$) δ = 6.01 (app. t, *J* = 1.0 Hz, 2H), 5.23 (ddt, *J* = 17.3, 10.1, 7.3 Hz, 1H), 5.02 (dq, *J* = 17.1, 1.5 Hz, 1H), 4.99 (app. ddt, *J* = 10.1, 1.9, 1.0 Hz, 1H), 2.57 (dt, *J* = 7.3, 1.3 Hz, 2H), 2.49 – 2.46 (br s, 1H), 2.06 (d, *J* = 1.2 Hz, 6H);

^{13}C NMR (151 MHz, $CDCl_3$) δ = 185.9, 160.7, 130.4, 127.8, 119.2, 74.6, 41.4, 18.2;

HRMS (ESI$^+$): Calculated for $C_{11}H_{15}O_2$ [M+H]$^+$: 179.1067, found 179.1065;

IR (thin film, cm^{-1}): 3374, 2956, 1664, 1607, 1383, 1315 and 900.

6. Synthesis of Enediones

6.1. Conditions A: Substoichiometric *p*-TsOH·H_2O in $(CH_2Cl)_2$

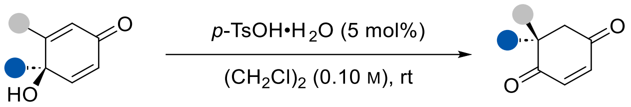

To an oven-dried 7 mL screw neck sample vial containing a magnetic stirrer bar under an atmosphere of nitrogen was charged the corresponding *p*-quinol (0.25 mmol, 1.0 equiv.) followed by anhydrous 1,2-dichloroethane ($(CH_2Cl)_2$, 2.5 mL, 0.10 M). To this was added *p*-TsOH·H_2O (2 mg, 12.5 µmol, 5 mol%) and the reaction was stirred at room temperature for the times stated below. Following this, the reaction was filtered through a short-pad of silica (5 × 5 cm) washing the transferring flask and silica with EtOAc (25 mL). The filtrate was concentrated under reduced pressure to afford a crude residue that was purified by flash column chromatography using the conditions stated below to afford the desired enedione.

6.2. Conditions B: Stoichiometric *p*-TsOH·H_2O in HFIP

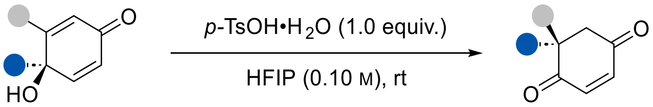

To an oven-dried 7 mL screw neck sample vial containing a magnetic stirrer bar under an atmosphere of nitrogen was charged the corresponding *p*-quinol (0.25 mmol, 1.0 equiv.) followed by hexafluoroisopropanol (HFIP, 2.5 mL, 0.10 M). To this was added *p*-TsOH·H_2O (48 mg, 0.25 mmol, 1.0 equiv.) and the reaction was stirred at room temperature for the time stated below. Following this, the reaction was filtered through a short-pad of silica (10 × 5 cm) washing the transferring flask and silica with EtOAc (25 mL). The filtrate was concentrated under reduced pressure to afford a crude residue that was purified by flash column chromatography using the conditions stated below to afford the desired enedione.

6.3. Tethered enedione (2a)

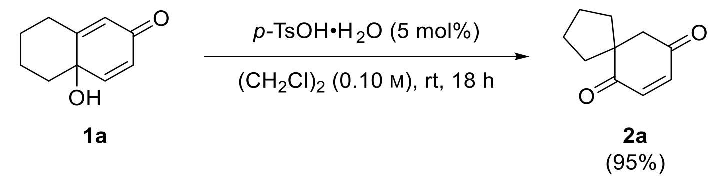

Prepared from *p*-quinol **1a** (41 mg, 0.25 mmol, 1.0 equiv.) using Conditions **A**.[A] Flash column chromatography (15% EtOAc in *n*-hexane) afforded enedione **2a** (39 mg, 0.24 mmol, 95%)[B,C] as yellow crystals.

Notes: (**A**) 18 h reaction time. (**B**) On a 12.2 mmol scale (2.00 g of *p*-quinol **1a**) the reaction afforded enedione **2a** (1.90 g, 11.6 mmol, 95%) as a yellow crystals. (**C**) This reaction can be carried out open to air using ACS reagent grade $(CH_2Cl)_2$ without affecting the yield or purity of the final compound.

R_f = 0.30 (20% EtOAc in *n*-hexane), stained with $KMnO_4$;

MP: 51–54 °C (TBME);

^{1}H NMR (500 MHz, $CDCl_3$) δ = δ = 6.70 (d, *J* = 10.3 Hz, 1H), 6.64 (d, *J* = 10.3 Hz, 1H), 2.79 (app. d, *J* = 0.7 Hz, 2H), 2.18 – 2.07 (m, 2H), 1.76 – 1.63 (m, 4H), 1.50 – 1.41 (m, 2H);

^{13}C NMR (126 MHz, $CDCl_3$) δ = 201.9, 198.2, 140.9, 140.2, 55.5, 50.2, 36.6, 25.5;

HRMS (EI$^+$): Calculated for $C_{10}H_{12}O_2$ $[M]^+$: 164.0832, found: 164.0832;

IR (thin film, cm^{-1}): 2961, 2884, 1682, 1600, 1422, 1280, 1249, 1116, 1082, 895, 834.

6.4. Keto-isophorone (2b)

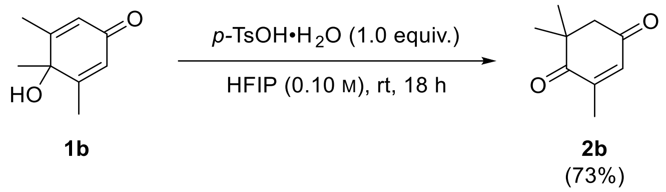

Prepared from *p*-quinol **1b** (390 mg, 2.5 mmol, 1.0 equiv.) according to Conditions **B**.[A] Flash column chromatography (10% acetone in *n*-hexane) afforded keto-isophorone (**2b**, 278 mg, 1.85 mmol, 73% yield)[B] as a yellow oil.

***R*f** = 0.35 (20% EtOAc in *n*-hexane), stained with $KMnO_4$;

Notes: (**A**) 18 h reaction time. (**B**) On a 0.25 mmol scale (38 mg of *p*-quinol **1b**) the reaction afforded keto-isophorone (**2b**) in 64% yield.

^{1}H NMR (500 MHz, $CDCl_3$) δ = 6.55 (tq, *J* = 1.4, 0.7 Hz, 1H), 2.71 (s, 2H), 2.00 (d, *J* = 1.5 Hz, 3H), 1.24 (s, 6H);

^{13}C NMR (126 MHz, $CDCl_3$) δ = 203.6, 197.8, 149.1, 137.2, 52.0, 45.3, 26.2, 16.9;

Analytical data are consistent with those reported previously in the literature.[14]

6.5. 5-Butyl-3,5-dimethyl enedione (2c)

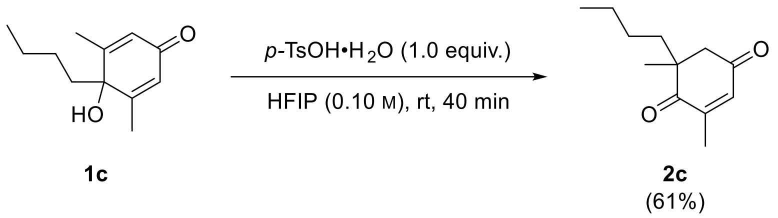

Prepared from *p*-quinol **1c** (23 mg, 0.12 mmol, 1.0 equiv.) using Conditions **B**. Flash column chromatography (5–10% EtOAc in *n*-hexane) afforded enedione **2c** (14 mg, 70 µmol, 61%)[A] as a brown oil.

Notes: (**A**) 40 min reaction time. (**B**) The desired enedione **2c** was obtained in 48% yield when using Conditions **A**.

$\boldsymbol{R_f}$ = 0.47 (20% EtOAc in *n*-hexane), stained with $KMnO_4$;

^{1}**H NMR** (500 MHz, $CDCl_3$): δ = 6.54 (app. p, *J* = 1.5 Hz, 1H), 2.78 (dd, *J* = 16.2, 0.9 Hz, 1H), 2.65 (d, *J* = 16.2 Hz, 1H), 1.99 (d, *J* = 1.6 Hz, 3H), 1.67–1.58 (m, 1H), 1.51 (ddd, *J* = 13.7, 11.7, 4.7 Hz, 1H), 1.30–1.20 (m, 4H), 1.19 (s, 3H), 1.05 (m, 1H), 0.86 (t, *J* = 7.2 Hz, 3H);

^{13}**C NMR** (126 MHz, $CDCl_3$): δ = 203.4, 198.2, 149.4, 137.1, 50.2, 48.6, 39.5, 26.9, 23.9, 23.2, 16.9, 14.0;

HRMS (ESI$^+$): Calc. for $C_{12}H_{18}O_2Na$ $[M+Na]^+$: 217.1199, found: 217.1198;

IR (thin film, cm^{-1}): 2958, 1675, 1623, 1460, 1378, 1277, 1185.

6.6. 5-Isopropyl 2,4,5,6-tetramethyl enedione (2d)

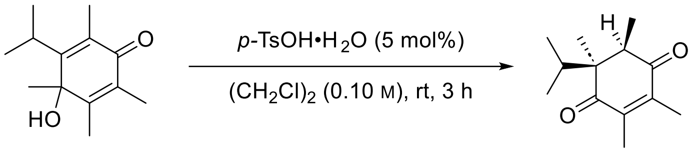

1d **(*cis*)-2d** (73%)

Prepared from *p*-quinol **1d** (52 mg, 0.25 mmol, 1.0 equiv.) using Conditions **A**.[A,B] Flash column chromatography (5% EtOAc in *n*-hexane) afforded enedione ***(cis)*-2d** (38 mg, 0.18 mmol, 73%) as a colourless oil.

Notes: (**A**) 3 h reaction time. (**B**) ^{1}H NMR analysis of the crude reaction mixture indicated a diastereomeric ratio of 7:1 *cis:trans.*

$\boldsymbol{R_f}$ = 0.47 (10% EtOAc in *n*-hexane), stained with $KMnO_4$;

^{1}H NMR (500 MHz, $CDCl_3$): δ = 2.75 (q, *J* = 7.0 Hz, 1H), 2.03 – 1.98 (m, 1H), 1.97 (app. dq, *J* = 3.1, 1.1 Hz, 6H), 1.23 – 1.17 (app. m, 6H), 0.84 (d, *J* = 6.9 Hz, 3H), 0.75 (d, *J* = 6.8 Hz, 3H);

^{13}C NMR (126 MHz, $CDCl_3$): δ = 203.2, 201.2, 145.2, 143.8, 55.3, 52.8, 32.2, 20.1, 20.0, 18.2, 13.4, 12.8, 11.2;

HRMS (ESI$^+$): Calc. for $C_{13}H_{21}O_2$ [M+H]$^+$: 209.1536, found: 209.1535;

IR (thin film, cm^{-1}): 2967, 1681, 1679, 1454, 1375, 1240, 1058.

6.7. 3,5-Dimethyl 4-phenyl enedione (2e)

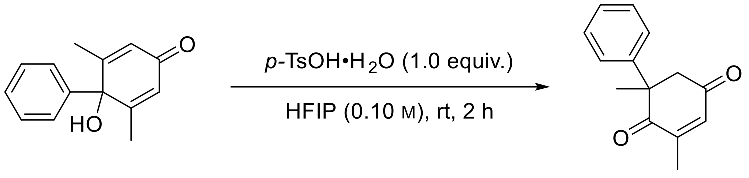

1e **2e** (80%)

Prepared from *p*-quinol **1e** (40 mg, 0.19 mmol, 1.0 equiv.) using Conditions **B**.[A] Flash column chromatography (20% EtOAc in *n*-hexane) afforded enedione **2e** (32 mg, 0.15 mmol, 80%) as a beige solid.

Notes: (**A**) 2 h reaction time.

R R_f = 0.29 (10% EtOAc in *n*-hexane), stained with *p*-anisaldehyde;

MP: 139–143 °C (*n*-hexane/EtOAc);

^{1}H NMR (601 MHz, $CDCl_3$): δ = 7.34–7.28 (m, 2H), 7.24 (m, 1H), 7.19–7.16 (m, 2H), 6.37 (p, *J* = 1.5 Hz, 1H), 3.45 (dd, *J* = 16.6, 1.6 Hz, 1H), 2.98 (d, *J* = 16.7 Hz, 1H), 1.95 (d, *J* = 1.6 Hz, 3H), 1.52 (s, 3H);

^{13}C NMR (126 MHz, $CDCl_3$): δ = 200.8, 197.0, 150.6, 141.5, 136.8, 129.3, 127.7, 125.6, 53.4, 49.2, 26.9, 17.4;

HRMS (ESI+): Calc. for $C_{14}H_{15}O_2$ $[M+H]^+$: 215.1067, found 215.1065;

IR (thin film, cm^{-1}): 2929, 1685, 1675, 1445, 1274, 917, 773.

6.8. 3,5-Dimethyl 4-(4-methoxy)phenyl enedione (2f)

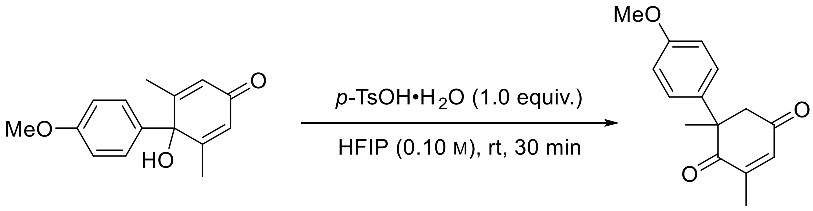

1f **2f** (72%)

Prepared from *p*-quinol **1f** (61 mg, 0.25 mmol, 1.0 equiv.) using Conditions **B**.[A] Flash column chromatography (10–20% EtOAc in *n*-hexane) afforded enedione **2f** (44 mg, 0.18 mmol, 72%) as a yellow solid.

Notes: (**A**) 30 min reaction time.

$\boldsymbol{R_f}$ = 0.49 (30% EtOAc in *n*-hexane), stained with *p*-anisaldehyde;

MP: 51–55 °C (*n*-hexane/EtOAc);

^{1}H NMR (500 MHz, $CDCl_3$): δ = 7.13 – 7.04 (m, 2H), 6.86 – 6.79 (m, 2H), 6.37 (p, *J* = 1.5 Hz, 1H), 3.76 (s, 3H), 3.40 (dd, *J* = 16.6, 1.6 Hz, 1H), 2.97 (d, *J* = 16.6 Hz, 1H), 1.94 (d, *J* = 1.5 Hz, 3H), 1.50 (s, 3H);

^{13}C NMR (126 MHz, $CDCl_3$): δ = 200.8, 197.2, 159.0, 150.5, 136.6, 133.5, 126.8, 114.6, 55.4, 52.6, 49.4, 26.9, 17.4;

HRMS (ESI$^+$): Calc. for $C_{15}H_{16}O_3Na$ $[M+Na]^+$: 267.0991; found 267.0992;

IR (thin film, cm^{-1}): 2965, 2929, 1678, 1513, 1255, 1186, 1031.

6.9. 3,5-Dimethyl 4-(4-trifluoromethyl)phenyl enedione (2g)

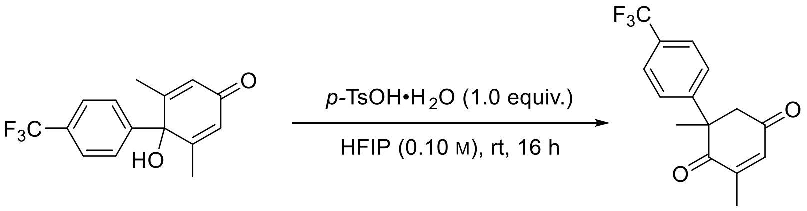

1g **2g** (56%)

Prepared from *p*-quinol **1g** (70 mg, 0.25 mmol, 1.0 equiv.) using Conditions **B**. Flash column chromatography (10% EtOAc in *n*-hexane) afforded enedione **2g** (39 mg, 0.14 mmol, 56%) as a light-yellow solid.

Notes: (**A**) 16 h reaction time.

R_f = 0.53 (30% EtOAc in *n*-hexane);

MP: 77–79 °C (*n*-hexane/EtOAc);

^{1}H NMR (500 MHz, $CDCl_3$): δ = 7.61–7.54 (m, 2H), 7.33–7.30 (m, 2H), 6.42 (p, *J* = 1.6 Hz, 1H), 3.45 (dd, *J* = 16.6, 1.6 Hz, 1H), 3.03 (d, *J* = 16.6 Hz, 1H), 1.97 (d, *J* = 1.6 Hz, 3H), 1.55 (s, 3H);

^{13}C NMR (126 MHz, $CDCl_3$): δ = 200.1, 196.4, 150.1, 145.5, 137.2, 130.1 (q, *J* = 32.8 Hz), 126.3 (q, *J* = 3.8 Hz), 126.1, 124.0 (q, *J* = 272.2 Hz), 53.4, 49.1, 26.8, 17.4;

^{19}F NMR (471 MHz, $CDCl_3$) δ = –62.77;

HRMS (ESI+): Calc. for $C_{15}H_{14}F_3O_2$ $[M+H]^+$: 283.0940; found 283.0937;

IR (thin film, cm^{-1}): 2975, 2932, 1681, 1618, 1326, 1166, 1115, 1079.

6.10. Estrone enedione (2h)

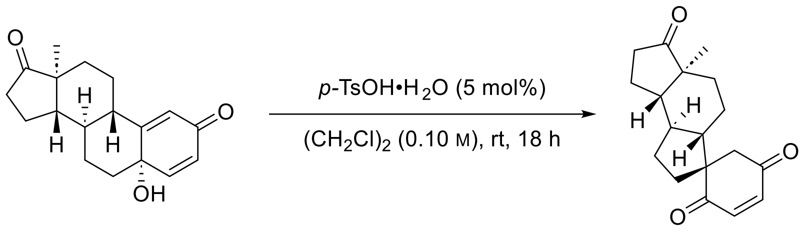

1h

2h (73%)

Prepared from *p*-quinol **1h** (143 mg, 0.50 mmol, 1.0 equiv.) using Conditions **A**. Flash column chromatography (0–30% acetone in *n*-hexane) afforded enedione **2h** (105 mg, >95:5 d.r., 100% d.s., 0.37 mmol, 73% [88% brsm])[A] as a light-yellow solid.

Notes: (**A**) On 0.20 mmol scale (57 mg of *p*-quinol **1h**) the reaction afforded enedione **2h** (40 mg, >95:5 d.r., 100% d.s., 0.14 mmol, 70% [quant. brsm]) as a light-yellow solid.

***R*f** = 0.46 (30% acetone in *n*-hexane), stained with *p*-anisaldehyde;

^{1}H NMR (500 MHz, $CDCl_3$) δ = 6.72 (dd, *J* = 10.3, 1.1 Hz, 1H), 6.66 (d, *J* = 10.3 Hz, 1H), 2.95 (d, *J* = 16.3 Hz, 1H), 2.76 (dd, *J* = 16.3, 1.1 Hz, 1H), 2.49 – 2.36 (m, 2H), 2.06 (dt, *J* = 19.3, 9.1 Hz, 1H), 1.96 – 1.83 (m, 3H), 1.77 (dt, *J* = 13.3, 3.0 Hz, 1H), 1.62 (tt, *J* = 12.5, 9.1 Hz, 1H), 1.54 – 1.41 (m, 2H), 1.34 – 1.08 (m, 5H), 0.78 (s, 3H);

^{13}C NMR (126 MHz, $CDCl_3$) δ = 219.8, 202.4, 198.2, 141.4, 140.8, 59.4, 56.5, 51.3, 50.9, 48.6, 40.8, 35.8, 35.2, 31.7, 27.5, 23.8, 22.5, 14.1;

HRMS (ESI$^+$): Calculated for $C_{18}H_{23}O_3$ $[M+H]^+$: 287.1642, found: 287.1641;

IR (thin film, cm^{-1}): 2932, 2858, 1736, 1674, 1606, 1453, 1403, 1375, 1339, 1263, 1200, 1085, 1075, 1005, 985, 959, 877, 768 and 702;

$[\alpha]_{\mathrm{D}}^{23}$**:** +126.0 (*c* = 1.00, $CHCl_3$);

Analytical data are consistent with those reported previously in the literature.[15]

6.11. 5,5-Dimethyl enedione (2i)

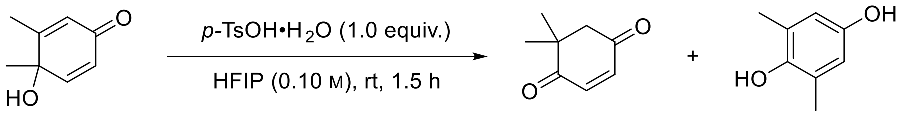

1i **2i** (40%) **4i** *not isolated*

Prepared from *p*-quinol **1i** (294 mg, 2.2 mmol, 1.0 equiv.) using Conditions **B**.[A,B] Flash column chromatography (5–10% EtOAc in *n*-hexane) afforded enedione **2i** (120 mg, 0.87 mmol, 40%) as a light yellow solid.

Notes: (**A**) 1.5 h reaction time. (**B**) ^{1}H NMR analysis of the crude reaction product showed an enedione **2i**:hydroquinone **4i** ratio of 59:41.

$\boldsymbol{R_f}$ = 0.53 (20% EtOAc in *n*-hexane), stained with $KMnO_4$;

MP: 45–50 °C (*n*-hexane/EtOAc);

^{1}H NMR (500 MHz, $CDCl_3$): δ = 6.68 (d, *J* = 10.3 Hz, 1H), 6.63 (d, *J* = 10.3 Hz, 1H), 2.74 (s, 2H), 1.24 (s, 6H);

^{13}C NMR (126 MHz, $CDCl_3$): δ = 203.2, 198.1, 140.2, 139.5, 51.8, 45.5, 26.0;

HRMS (EI^{+}): Calc. for $C_8H_{10}O_2$ $[M]^{+}$: 138.0675, found: 138.0679;

IR (thin film, cm^{-1}): 2968, 1682, 1370, 1284, 1079, 882, 821.

Analytical data are consistent with those reported previously in the literature.[16]

6.12. 5-Isopropyl-5-methylenedione (2j)

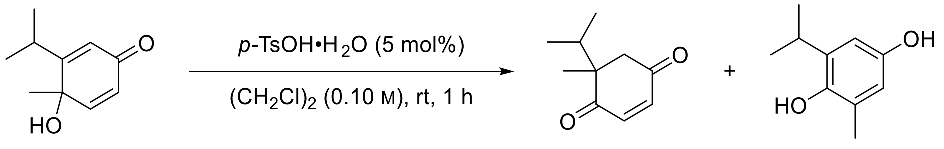

1j **2j** (15%) **4j** (69%)

Prepared from *p*-quinol **1j** (42 mg, 0.25 mmol, 1.0 equiv.) using Conditions **A**.[A,B] Flash column chromatography (5–20% EtOAc in *n*-hexane) afforded enedione **2j** (6.2 mg, 40 μmol, 15%) as a colourless oil and hydroquinone **4j** (28 mg, 0.17 mmol, 69%) as a white solid.

Notes: (**A**) 1 h reaction time. (**B**) ^{1}H NMR analysis of the crude reaction mixture showed an enedione **2j**:hydroquinone **4j** ratio of 14:86.

Data for **2j**

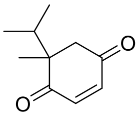

2j

***R*f** = 0.6 (20% EtOAc in *n*-hexane), stained with *p*-anisaldehyde;

^{1}H NMR (500 MHz, CDCl3): δ =6.67 (dd, *J* = 10.2, 0.9 Hz, 1H), 6.62 (d, *J* = 10.3 Hz, 1H), 2.89 (dd, *J* = 16.3, 0.9 Hz, 1H), 2.59 (d, *J* = 16.3 Hz, 1H), 2.15 – 2.03 (app. m, 1H), 1.12 (s, 3H), 0.91 (d, *J* = 6.8 Hz, 3H), 0.82 (d, *J* = 6.9 Hz, 3H);

^{13}C NMR (126 MHz, CDCl3): δ = 203.5, 198.7, 140.1, 140.0, 52.2, 47.0, 33.9, 20.4, 17.9, 17.4;

HRMS (EI^{+}): Calc. for $C_{10}H_{14}O_2$ [M]$^{+}$: 166.0988, found: 166.0993;

IR (thin film, cm^{-1}): 2967, 1679, 1653, 1378, 1279, 1073.

Data for **4j**

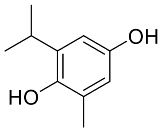

4j

***R*f** = 0.26 (20% EtOAc in *n*-hexane), stained with *p*-anisaldehyde;

MP: 127–133 °C (*n*-hexane/EtOAc);

^{1}H NMR (500 MHz, $CDCl_3$): δ = 6.55 (d, *J* = 3.0 Hz, 1H), 6.50 – 6.45 (app. m, 1H), 4.24 (br s, 2H), 3.16 (hept, *J* = 6.9 Hz, 1H), 2.21 (s, 3H), 1.23 (d, *J* = 6.9 Hz, 6H);

^{13}C NMR (126 MHz, $CDCl_3$): δ = 149.1, 145.1, 135.5, 124.5, 114.8, 110.8, 27.4, 22.8, 16.4;

HRMS (EI$^+$): Calc. for $C_{10}H_{14}O_2$ $[M]^+$: 166.0988, found: 166.0989;

IR (thin film, cm^{-1}): 3291, 2961, 1603, 1461, 1181, 977, 857.

6.13. 2,5,5-Trimethyl enedione (2k)

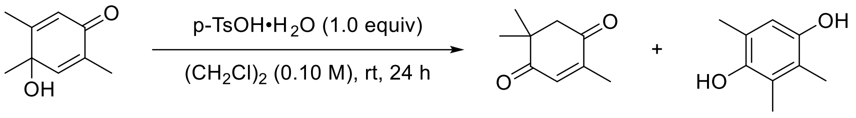

1k 2k (31%) 4k *not isolated*

Prepared from *p*-quinol **1k** (38 mg, 0.25 mmol, 1.0 equiv.) using Conditions **A**.[A] Flash column chromatography (10–20% EtOAc in *n*-hexane) afforded enedione **2k** (12 mg, 80 μmol, 31%)[B–E] as a light yellow oil.

Notes: (**A**) 24 h reaction time, 1.0 equiv. of *p*-TsOH·H_2O used. (**B**) ^{1}H NMR analysis of the crude residue showed 45% conversion to **2k,** with an enedione **2k**:hydroquinone **4k** ratio of 88:12 (**C**) The product appears to be volatile under high vacuum (0.1 mbar). The desired enedione **2k** was isolated with some impurities (hydroquinone **4k** and its corresponding benzoquinone). (**D**) Attempting the rearrangement using 1.0 equiv. of *p*-TsOH·H_2O and 5.0 equiv. of HFIP in $(CH_2Cl)_2$ gave the desired enedione **2k** in 15% NMR yield with significant decomposition after 1 h at room temperature. (**E**) Attempting the rearrangement using Conditions **B** resulted in complete decomposition.

***R*f** = 0.33 (20% EtOAc in *n*-hexane), stained with $KMnO_4$;

^{1}H NMR (500 MHz, $CDCl_3$): δ = 6.50 (q, *J* = 1.6 Hz, 1H), 2.73 (s, 2H), 1.99 (d, *J* = 1.6 Hz, 3H), 1.22 (s, 6H);

^{13}C NMR (126 MHz, $CDCl_3$): δ = 203.4, 198.7, 149.9, 136.4, 51.9, 45.9, 29.8, 26.1, 16.0;

HRMS (EI$^+$): Calc. for $C_9H_{12}O_2$ $[M]^+$: 152.0832, found: 152.0827;

IR (thin film, cm^{-1}): 2924, 1640, 1276, 750.

6.14. 2,5-Dimethyl 5-isopropyl enedione (2l)

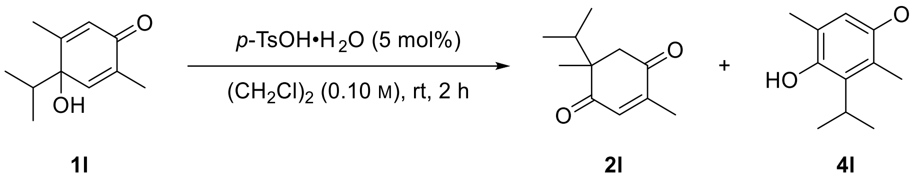

Prepared from *p*-quinol **1l** (45 mg, 0.25 mmol, 1.0 equiv.) using Conditions **A**. [A,B] Flash column chromatography (2–20% EtOAc in *n*-hexane) afforded enedione **2l** (25 mg, 0.14 mmol, 55%) as a yellow solid and hydroquinone **4l** (3 mg, 20 μmol, 7%) as a colourless oil.

Notes: (**A**) 2 h reaction time. (**B**) ^{1}H NMR analysis of the crude reaction mixture showed an enedione **2l**:hydroquinone **4l** ratio of 59:41.

Data for **2l**

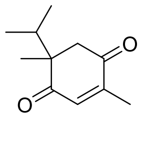

2l

***R*f** = 0.44 (20% EtOAc in *n*-hexane), stained with *p*-anisaldehyde;

MP: 53–58 °C (*n*-hexane/EtOAc);

^{1}H NMR (500 MHz, $CDCl_3$): δ 6.49 (q, *J* = 1.5 Hz, 1H), 2.88 (d, *J* = 16.0 Hz, 1H), 2.57 (d, *J* = 16.0 Hz, 1H), 2.10 – 2.00 (m, 1H), 1.98 (d, *J* = 1.5 Hz, 3H), 1.10 (s, 3H), 0.89 (d, *J* = 6.8 Hz, 3H), 0.81 (d, *J* = 6.9 Hz, 3H);

^{13}C NMR (126 MHz, $CDCl_3$): δ 203.6, 199.3, 149.6, 137.1, 52.5, 47.0, 34.0, 20.6, 18.0, 17.4, 15.9;

HRMS (ESI$^+$): Calc. for $C_{11}H_{16}O_2Na$ $[M+Na]^+$: 203.1043, found: 203.1046;

IR (thin film, cm^{-1}): 2966, 1681, 1625, 1448, 1378, 1259, 1205, 1144.

Data for **4l**

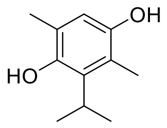

4l

***R*$_f$** = 0.26 (20% EtOAc in *n*-hexane), stained with *p*-anisaldehyde;

^{1}H NMR (500 MHz, $CDCl_3$): δ 6.45 (s, 1H), 4.22 (d, *J* = 1.8 Hz, 2H), 3.35 (app. p, *J* = 6.7 Hz, 1H), 2.20 (s, 3H), 2.16 (s, 3H), 1.36 (d, *J* = 7.1 Hz, 6H);

^{13}C NMR (126 MHz, $CDCl_3$): δ 147.2, 133.5, 121.5, 120.5, 114.7, 20.8 (2C), 15.9, 12.1;

HRMS (ESI+): Calc. for $C_{11}H_{17}O_2$ $[M+H]^+$: 181.1223, found: 181.1227;

IR (thin film, cm^{-1}): 3431, 2957, 1645, 1468, 1313, 1267, 1234, 1069, 864.

6.15. 2,5-Dimethyl 5-phenyl enedione (2m)

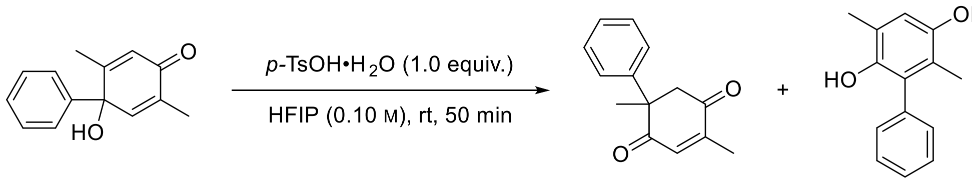

1m **2m** (58%) **4m** (23%)

Prepared from *p*-quinol **1m** (40 mg, 0.19 mmol, 1.0 equiv.) using Conditions **B**.[A,B] Flash column chromatography (1–5% EtOAc in *n*-hexane) afforded enedione **2m** (23 mg, 0.11 mmol, 58%) as a yellow oil and hydroquinone **4m** (9 mg, 40 μmol, 23%) as a yellow oil.

Notes: (**A**) 50 min reaction time. (**B**) ^{1}H NMR analysis of the crude reaction product showed a enedione **2m**:hydroquinone **4m** ratio of 57:43.

Data for **2m**

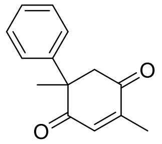

2m

***R*f** = 0.38 (20% EtOAc in *n*-hexane), stained with *p*-anisaldehyde;

^{1}H NMR (500 MHz, $CDCl_3$): δ = 7.33–7.28 (m, 2H), 7.26–7.20 (m, 3H), 6.50 (q, *J* = 1.6 Hz, 1H), 3.48 (d, *J* = 16.4 Hz, 1H), 2.99 (d, *J* = 16.4 Hz, 1H), 1.88 (d, *J* = 1.6 Hz, 3H), 1.52 (s, 3H);

^{13}C NMR (126 MHz, $CDCl_3$): δ = 200.2, 197.8, 149.5, 141.6, 137.3, 129.2, 127.6, 125.8, 53.7, 49.4, 26.8, 15.9;

HRMS (ESI+): Calc. for $C_{14}H_{15}O_2$ $[M+H]^+$: 215.1067, found: 215.1070;

IR (thin film, cm^{-1}): 2920, 1682, 1445, 1259, 913, 743.

Data for **4m**

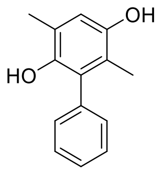

4m

***R*$_f$** = 0.36 (20% EtOAc in *n*-hexane), stained with *p*-anisaldehyde;

^{1}H NMR (500 MHz, $CDCl_3$): δ = 7.45 – 7.37 (m, 3H), 7.18 – 7.11 (m, 2H), 6.69 (q, *J* = 1.6 Hz, 1H), 2.09 (d, *J* = 1.6 Hz, 3H), 1.94 (s, 3H);

^{13}C NMR (126 MHz, $CDCl_3$): δ = 188.3, 187.3, 145.7, 144.1, 141.7, 133.4, 133.4, 129.5, 128.6, 128.3, 16.2, 13.9;

HRMS (ESI$^+$): Calc. for $C_{14}H_{14}O_2Na$ $[M+Na]^+$: 237.0886, found: 237.0882;

IR (thin film, cm^{-1}): 3403, 2923, 2852, 1488, 1260, 1224, 1121, 799.

6.16. Allyl enedione (2p)

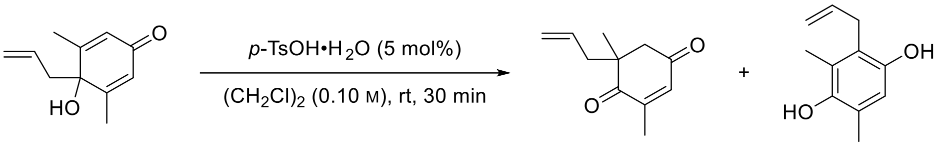

Prepared from *p*-quinol **1p** (22 mg, 0.12 mmol, 1.0 equiv.) using Conditions **A**.[A,B] Product yield was determined by ^{1}H NMR spectroscopy using 1,2-dimethoxyethane as internal standard. (See NMR spectrum, section 9.3.36, page 114)

Notes: (**A**) 30 min reaction. (**B**) ^{1}H NMR analysis of the crude reaction mixture showed an enedione **2p**:hydroquinone **5** ratio of 15:85.

Data for hydroquinone **5** is consistent with those reported previously in the literature.[17]

Data for **2p**

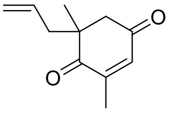

2p

^{1}H NMR (500 MHz, $CDCl_3$): δ = 6.59 (td, *J* = 1.6, 0.9 Hz, 1H), 5.69 (dddd, *J* = 17.0, 10.2, 8.0, 6.9 Hz, 1H), 5.13 (ddt, *J* = 10.2, 1.9, 0.9 Hz, 1H), 5.04 (overlapped, 1H), 2.86 (d, *J* = 16.3 Hz, 1H), 2.64 (dd, *J* = 16.4, 0.6 Hz, 1H), 2.49 (ddt, *J* = 13.7, 6.8, 1.2 Hz, 1H), 2.27 – 2.23 (overlapped, 1H), 2.02 (d, *J* = 1.5 Hz, 3H), 1.45 (s, 3H).

Data for **5**

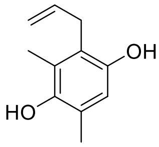

4p

^{1}H NMR (500 MHz, $CDCl_3$): δ = 6.52 (s, 1H), 5.97 (ddt, *J* = 17.1, 10.1, 5.8 Hz, 1H), 5.07 (dq, *J* = 10.2, 1.7 Hz, 1H), 5.01 (dq, *J* = 17.2, 1.8 Hz, 1H), 3.44 – 3.42 (m, 3H), 2.22 (d, *J* = 0.7 Hz, 3H), 2.20 (d, *J* = 0.6 Hz, 3H).

7. Synthesis of Enedione Derivatives

7.1. Diol 6

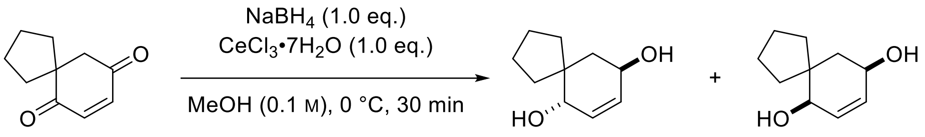

2a **6** *major* **S20** *minor*

(74% combined yield, 1.3:1 d.r.)

To a round-bottom flask equipped with a magnetic stirrer bar was added enedione **2a** (41 mg, 0.25 mmol, 1.0 equiv.), $CeCl_3 \cdot 7H_2O$ (93 mg, 0.25 mmol, 1.0 equiv.) and MeOH (2.5 mL). The mixture was cooled to 0 °C after which, $NaBH_4$ (10 mg, 0.26 mmol, 1.0 equiv.) was added portion-wise. The reaction was stirred at 0 °C for 30 min. Upon completion, the mixture was diluted with ice/brine (5 mL) and extracted with CH_2Cl_2 (3 × 5 mL). The combined organic phases were dried over anhydrous Na_2SO_4, filtered, and concentrated under reduced pressure. The resulting crude residue was purified by flash column chromatography (50% EtOAc in hexane) to afford a mixture of diols **6** and **S20** (31 mg, 0.18 mmol, 74% combined yield). A small sample of this product mixture was subjected to further flash column chromatography (15%–50% EtOAc) yielding an analytically pure sample of diol **6** as a colourless solid.

***R*f** = 0.12 (50% EtAOc in *n*-hexane), stained with $KMnO_4$;

MP = 133–137 °C (*n*-hexane/EtOAc);

^{1}H NMR (500 MHz, $CDCl_3$): δ = 5.76 (app. ddt, *J* = 10.1, 2.6, 1.4 Hz, 1H), 5.73 (app. dt, *J* = 10.0, 1.6 Hz, 1H), 4.30 (br s, 1H), 4.09 (br s, 1H), 2.07 (ddd, *J* = 13.2, 5.6, 1.3 Hz, 1H), 1.81 – 1.57 (m, 5H), 1.45 (ddd, *J* = 13.1, 7.7, 2.9 Hz, 3H), 1.34 – 1.26 (m, 1H);

^{13}C NMR (126 MHz, $CDCl_3$): δ = 132.9, 131.8, 73.1, 66.4, 48.1, 43.0, 37.3, 30.4, 26.3, 25.1;

HRMS (EI$^+$): Calculated for $C_{10}H_{16}O_2$ $[M]^+$: 168.1145, found: 168.1137;

IR (thin film, cm^{-1}): 3276, 2912, 2850, 1463, 1366 and 719.

7.2. Epoxide 7

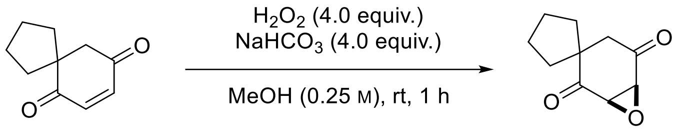

2a **7** (60%)

To an oven-dried 7 mL screw neck sample vial equipped with a magnetic stirrer was added enedione **2a** (41 mg, 0.25 mmol, 1.0 equiv.), $NaHCO_3$ (84 mg, 1.0 mmol, 4.0 equiv.) and MeOH (1.0 mL). To this mixture was added H_2O_2 (30% w/w in H_2O, 102 µL, 1.0 mmol, 4.0 equiv.) at room temperature. The reaction was stirred at room temperature for 1 h. Upon completion, the mixture was diluted with water (5 mL) and extracted with Et_2O (3 × 5 mL). The combined organic phases were washed with H_2O (10 mL), brine (10 mL), dried over anhydrous Na_2SO_4, filtered, and concentrated under reduced pressure. The resulting crude residue was purified by flash column chromatography (20% EtOAc in hexane) to afford epoxide **7** (27 mg, 0.15 mmol, 60% yield) as a yellow oil.

***R*$_f$** = 0.44 (25% EtOAc in *n*-hexane), stained with vanillin;

^{1}H NMR (500 MHz, $CDCl_3$): δ = 3.66 (d, *J* = 3.8 Hz, 1H), 3.60 (dd, *J* = 3.9, 1.0j Hz, 1H), 3.11 (d, *J* = 14.0 Hz, 1H), 2.25 – 2.18 (overlapped, 1H), 2.22 (d, *J* = 14.1 Hz, 1H), 1.83 – 1.75 (m, 2H), 1.72 – 1.63 (m, 3H), 1.53 (app. ddd, *J* = 13.4, 7.7, 5.3 Hz, 1H), 1.49 – 1.43 (m, 1H);

^{13}C NMR (126 MHz, $CDCl_3$): δ = 205.2, 203.5, 59.1, 56.9, 55.5, 45.0, 38.6, 38.0, 25.7, 25.0;

HRMS (EI$^+$): Calculated for $C_{10}H_{12}O_3$ $[M]^+$: 180.0781, found: 180.0778;

IR (thin film, cm^{-1}): 2957, 2872, 1706, 1449, 1278, 1001 and 867.

7.3. Michael adduct 8

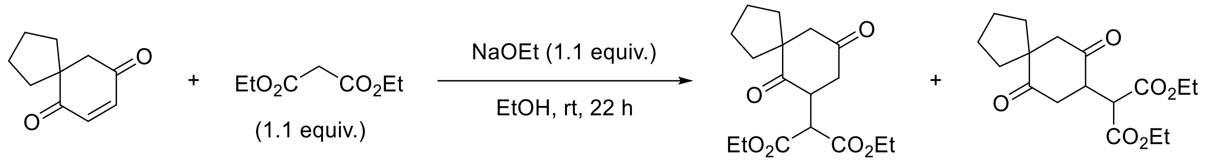

2a **8** *major* **S21** *minor*

(98% combined yield, 6:1 r.r.)

To a solution of enedione **2a** (41 mg, 0.25 mmol, 1.0 equiv.) and diethyl malonate (42 µL, 0.28 mmol, 1.1 equiv.) in EtOH (2 mL) was added NaOEt (19 mg, 0.27 mmol, 1.1 equiv.). The reaction was stirred at room temperature for 22 h. Upon completion, the reaction was quenched by the addition of a saturated solution of aqueous NH_4Cl (5 mL) and extracted with EtOAc (3 × 5 mL). The combined organic phases were dried over anhydrous Na_2SO_4, filtered, and concentrated under reduced pressure. The resulting crude residue was purified by flash column chromatography (20% EtOAc in hexane) to afford a mixture of isomers **8** and **S1** (79 mg, 0.24 mmol, 98% combined yield) as a pale-yellow oil which solidified to an off-white, amorphous solid after storing at 4 °C.

R_f = 0.31 (20% EtOAc in *n*-hexane), stained with vanillin;

^{1}H NMR (601 MHz, $CDCl_3$): δ = 4.27 – 4.17 (m, 4H$^{\dagger+\ddagger}$), 3.94 (d, *J* = 5.9 Hz, 1H‡), 3.90 (d, *J* = 6.8 Hz, 1H†), 3.58 (dt, *J* = 13.4, 6.6 Hz, 1H†), 3.31 (dt, *J* = 13.7, 6.0 Hz, 1H‡), 3.10 (dd, *J* = 16.2, 13.6 Hz, 1H‡), 2.84 (dd, *J* = 17.3, 13.5 Hz, 1H†), 2.77 (d, *J* = 15.7 Hz, 1H†), 2.74 – 2.67 (m, 1H† + 3H‡), 2.62 (d, *J* = 15.6 Hz, 1H†), 2.15 – 2.06 (m, 2H$^{\dagger+\ddagger}$), 1.80 – 1.63 (m, 4H$^{\dagger+\ddagger}$), 1.41 – 1.33 (m, 2H$^{\dagger+\ddagger}$), 1.28 (app. tdd, *J* = 7.1, 2.9, 1.3 Hz, 6H$^{\dagger+\ddagger}$);

^{13}C NMR (126 MHz, $CDCl_3$): δ = 210.9†, 210.5‡, 206.7‡, 206.6†, 168.1†, 168.0†, 168.0‡, 167.9‡, 62.1‡, 62.1†, 62.0‡, 61.9†, 55.4‡, 54.4†, 51.5‡, 51.2†, 50.6†, 49.7‡, 46.0‡, 45.1†, 40.0†, 39.6‡, 37.6†, 36.9‡, 36.5†, 36.4‡, 25.8†, 25.7‡, 25.7‡, 25.6†, 14.2† (2C), 14.1‡ (2C);

HRMS (ESI$^+$): Calculated for $C_{17}H_{25}O_6$ $[M+H]^+$: 325.1646, found: 325.1648;

IR (thin film, cm^{-1}): 2869, 1750, 1724, 1702 and 1193.

† Major regioisomer, ‡ Minor regioisomer

7.4. Hydrazone 9

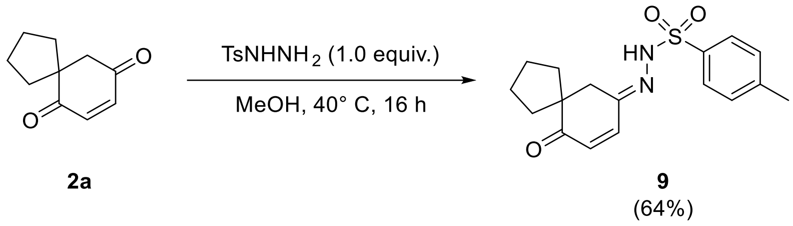

To an oven-dried round-bottom was added enedione **2a** (41 mg, 0.25 mmol, 1.0 equiv.), *p*-toluenesulfonyl hydrazide (42 mg, 0.25 mmol, 1.0 equiv.) and MeOH (2.5 mL). The reaction was stirred at 40 °C overnight. Upon completion, the resulting cream coloured suspension was concentrated under reduced pressure to give a crude residue which was purified by flash column chromatography (20% EtOAc in *n*-hexane) to afford hydrazone **9** (54 mg, 0.16 mmol, 64%) as a yellow solid.

***R*f** = 0.22 (20% EtOAc in *n*-hexane), stained with $KMnO_4$;

^{1}H NMR (500 MHz, $CDCl_3$): δ = 8.55 (br. s, 1H), 7.85 (d, *J* = 8.1 Hz, 2H), 7.34 (d, *J* = 8.0 Hz, 2H), 6.91 (d, *J* = 10.1 Hz, 1H), 6.18 (d, *J* = 10.2 Hz, 1H), 2.59 (s, 2H), 2.44 (s, 3H), 2.02 (app. dt, *J* = 11.2, 5.7 Hz, 2H), 1.63 (app. tt, *J* = 10.1, 5.1 Hz, 4H), 1.35 – 1.28 (m, 2H);

^{13}C NMR (126 MHz, $CDCl_3$): δ = 201.8, 150.4, 144.9, 142.2, 135.0, 131.4, 130.0, 128.1, 52.8, 36.9, 36.0, 25.5, 21.8;

HRMS (ESI$^+$): Calculated for $C_{17}H_{20}N_2O_3SNa$ $[M+Na]^+$: 355.1087, found: 355.1084;

IR (thin film, cm^{-1}): 3223, 2960, 1669, 1345, 1168, 1068, 911 and 663.

7.5. Cyclopentadiene adduct 10

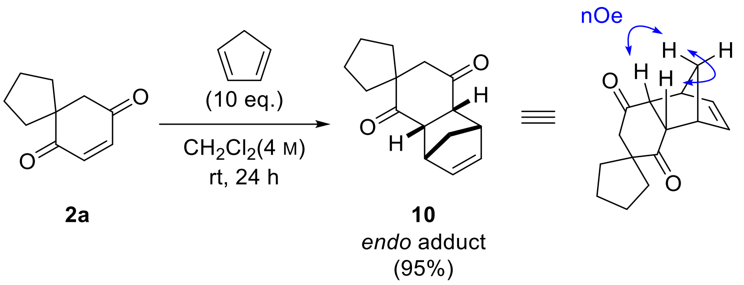

To an oven-dried 7 mL screw neck sample vial equipped with a magnetic stirrer was added enedione **2a** (100 mg, 0.61 mmol), CH_2Cl_2 (0.15 mL) and freshly cracked cyclopentadiene[A] (0.5 mL, 6.1 mmol). The reaction was stirred at room temperature for 24 h. Upon completion, the mixture was concentrated under reduced pressure to afford a crude residue which was purified by flash column chromatography (10% EtOAc in hexane) to afford *endo*-adduct **10** (134 mg, 0.58 mmol, 95% yield) as a white solid.

Notes: (**A**) Cyclopentadiene was obtained by cracking dicyclopentadiene according to a known procedure.[18]

$\boldsymbol{R_f}$ = 0.31 (20% EtOAc in *n*-hexane), stained with $KMnO_4$;

^{1}H NMR (500 MHz, $CDCl_3$): δ = 6.22 (dd, *J* = 5.7, 2.9 Hz, 1H), 6.06 (dd, *J* = 5.7, 2.9 Hz, 1H), 3.52 (app. dhept, *J* = 4.0, 1.7 Hz, 1H), 3.36 (app ddq, *J* = 4.1, 2.7, 1.5 Hz, 1H), 3.27 (dd, *J* = 9.9, 3.7 Hz, 1H), 3.22 (ddd, *J* = 9.9, 3.8, 1.4 Hz, 1H), 2.43 (dd, *J* = 15.9, 1.5 Hz, 1H), 2.25 – 2.15 (m, 2H), 1.85 (app. ddd, *J* = 12.5, 6.38, 5.3 Hz, 1H), 1.72 – 1.45 (m, 6H), 1.37 (app. dt, *J* = 8.5, 1.6 Hz, 1H), 1.05 (app. ddd, *J* = 13.4, 8.0, 5.9 Hz, 1H);

^{13}C NMR (126 MHz, $CDCl_3$): δ = 211.9, 210.4, 138.0, 135.1, 56.0, 52.1, 50.8, 50.2, 49.1, 48.4, 46.5, 36.8, 33.1, 25.3, 24.8;

HRMS (ESI$^+$): Calculated for $C_{15}H_{18}NaO_2$ $[M+Na]^+$: 253.1199, found: 253.1204;

IR (thin film, cm^{-1}): 2991, 2944, 2867, 1697, 1242, 1157, 1053, 911 and 729.

7.6. Isoprene adduct 11

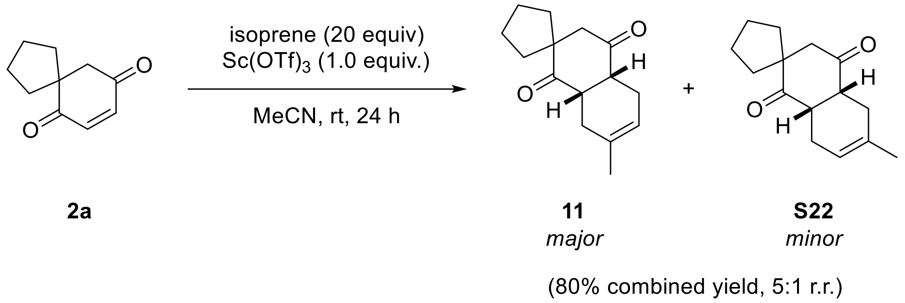

To an oven-dried round-bottom flask equipped with a magnetic stirrer was added enedione **2a** (164 mg, 1.0 mmol, 1.0 equiv.), $Sc(OTf)_3$ (492 mg, 1.0 mmol, 1.0 equiv.) and MeCN (2.0 mL). To the resulting mixture was added isoprene (2.0 mL, 20 mmol, 20 equiv.) and the flask was sealed. The reaction was stirred at room temperature for 24 h. Upon completion, the reaction was quenched by the addition of a solution of saturated aqueous $NaHCO_3$ (10 mL) and extracted with EtOAc (3 × 15 mL). The organic phases were combined, washed with brine (10 mL), dried with anhydrous Na_2SO_4, filtered, and concentrated under reduced pressure. The resulting crude residue was purified by flash column chromatography (15% EtOAc in *n*-hexane) to give an inseparable mixture of adducts **11** and **S22** (186 mg, 0.80 mmol, 80% yield)[A] as a colourless oil which solidified to a colourless, amorphous solid after storing at 4 °C.

Notes: (**A**) This reaction can be performed under thermal conditions without $Sc(OTf)_3$ (sealed tube, 120 °C, 1% BHT, 24 h). NMR analysis of the crude reaction mixture shows a 1.3:1 (**11**:**S22**) ratio of regioisomers.

***R*f** = 0.44 (20% EtOAc in *n*-hexane), stained with $KMnO_4$;

^{1}H NMR (500 MHz, $CDCl_3$) both isomers: δ 5.34 (app. tt, *J* = 3.6, 1.7 Hz, 1H‡), 5.31 (app. tt, *J* = 3.2, 1.6 Hz, 1H†), 3.22 (app. dt, *J* = 6.7, 5.5 Hz, 1H†), 3.14 – 3.09 (m, 1H‡), 3.00 – 2.95 (m, 1H‡), 2.89 (app. ddd, *J* = 8.0, 6.7, 5.6 Hz, 1H†), 2.74 (app. dd, *J* = 16.7, 3.6 Hz, 1H$^{†+‡}$), 2.60 (dd, *J* = 16.7, 4.4 Hz, 1H$^{†+‡}$), 2.37 (tp, *J* = 3.9, 1.9 Hz, 1H‡), 2.29 (ddd, *J* = 17.9, 5.1, 2.5 Hz, 1H†), 2.24 – 2.08 (m, 2H† + 3H‡), 2.08 – 1.99 (m, 1H†), 1.96 – 1.86 (m, 1H† + 1H‡), 1.81 – 1.56 (m, 9H $^{†+‡}$), 1.32 (ddd, *J* = 12.0, 7.3, 4.7 Hz, 1H† + 1H‡).

^{13}**C NMR** (126 MHz, $CDCl_3$): δ =213.9‡, 213.7†, 210.4†, 210.2‡, 132.0†, 131.5‡, 118.7‡, 118.2†, 53.9†, 53.9‡, 49.7†, 49.6‡, 45.6‡, 45.0†, 44.6†, 43.8‡, 40.0†, 39.9‡, 37.2†+‡, 28.7‡, 28.1†, 26.1‡, 26.0†, 26.0‡, 25.9†, 24.7†, 24.0‡, 23.6‡, 23.5†;

† Major regioisomer

‡ Minor regioisomer

HRMS (ESI$^+$): Calculated for $C_{15}H_{20}O_2Na$ $[M+Na]^+$: 255.1356, found: 255.1354;

IR (thin film, cm^{-1}): 2960, 2968, 1697 and 1148.

7.7. Enol acetate 12

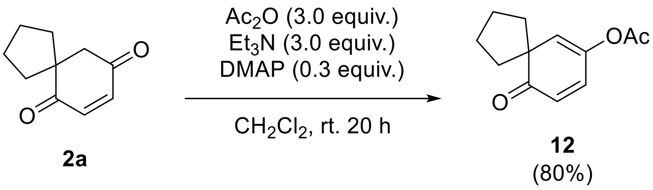

2a **12** (80%)

To a solution of enedione **2a** (41 mg, 0.25 mmol, 1.0 equiv.) in CH_2Cl_2 (5 mL) was added acetic anhydride (71 μL, 0.75 mmol, 3.0 equiv.), DMAP (9 mg, 75 μmol, 0.3 equiv.) and triethylamine (105 μL, 0.75 mmol, 3.0 equiv.). This mixture was stirred at room temperature for 20 h. Upon completion, the reaction was quenched by the addition of a solution of saturated aqueous NH_4Cl (5 mL). The mixture was transferred to a separating funnel containing H_2O (5 mL) and extracted with CH_2Cl_2 (3 × 10 mL). The organic phases were combined, washed with brine (10 mL), dried with anhydrous Na_2SO_4, filtered, and concentrated under reduced pressure. The resulting crude residue was purified by flash column chromatography (20% EtOAc in *n*-hexane to afford enol acetate **12** (41 mg, 0.20 mmol, 80% yield) as a yellow oil.

R_f = 0.25 (20% EtOAc in *n*-hexane), stained with vanillin;

^{1}H NMR (601 MHz, $CDCl_3$): δ = 6.82 (dd, *J* = 10.1, 3.0 Hz, 1H), 6.06 (dd, *J* = 10.1, 0.7 Hz, 1H), 6.02 (dd, *J* = 3.0, 0.7 Hz, 1H), 2.21 (s, 3H), 2.09 – 2.02 (m, 2H), 2.01 – 1.89 (m, 2H), 1.85 – 1.72 (m, 4H);

^{13}C NMR (151 MHz, $CDCl_3$): δ = 204.7, 169.4, 141.3, 140.1, 131.8, 126.8, 57.6, 39.9, 26.0, 21.0;

HRMS (EI$^+$): Calculated for $C_{12}H_{14}O_3$ $[M]^+$: 206.0938, found: 206.0937;

IR (thin film, cm^{-1}): 2953, 1755, 1636, 1200 and 1150.

7.8. NPM Adduct 13

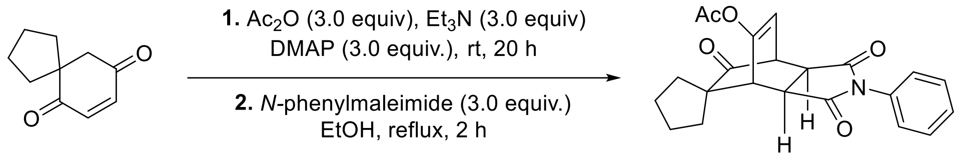

2a **13** (82%)

To a solution of enedione **2a** (233 mg, 1.4 mmol, 1.0 equiv.) in CH_2Cl_2 (10 mL) was added acetic anhydride (0.40 mL, 4.2 mmol, 3.0 equiv.), DMAP (52 mg, 0.43 mmol, 0.30 equiv.) and triethylamine (0.59 mL, 4.2 mmol, 3.0 equiv.). This mixture was stirred at room temperature for 20 h. Upon completion, the reaction was quenched by the addition of a solution of saturated aqueous NH_4Cl (15 mL). The mixture was transferred to a separating funnel containing H_2O (20 mL) and extracted with CH_2Cl_2 (3 × 20 mL). The organic phases were combined, washed with brine (10 mL), dried with anhydrous Na_2SO_4, filtered, and concentrated under reduced pressure to afford a crude residue. This crude residue was dissolved in EtOH (5 mL) after which, *N*-phenyl maleimide (738 mg, 4.3 mmol, 3.0 equiv.) was added in a single portion. The reaction mixture was stirred under reflux for 2 h. Upon completion, the reaction was cooled to room temperature and concentrated under reduced pressure. The resulting crude residue was purified by flash column chromatography (30% EtOAc in *n*-hexane) to give adduct **13** (440 mg, 1.2 mmol, 82% yield) as white crystals.

$\boldsymbol{R_f}$ = 0.34 (50% EtOAc in *n*-hexane), stained with $KMnO_4$;

MP = 144–147 °C (Et_2O);

^{1}H NMR (500 MHz, $CDCl_3$): δ = 7.48 – 7.44 (m, 2H), 7.42 – 7.37 (m, 1H), 7.25 – 7.22 (m, 2H), 5.93 (dd, *J* = 7.0, 2.7 Hz, 1H), 3.81 (dd, *J* = 7.0, 3.0 Hz, 1H), 3.44 (dd, *J* = 8.4, 3.3 Hz, 1H), 3.33 (dd, *J* = 8.5, 3.0 Hz, 1H), 3.16 (dd, *J* = 3.3, 2.7 Hz, 1H), 2.15 (s, 3H), 1.96 – 1.76 (m, 5H), 1.75 – 1.57 (m, 3H);

^{13}C NMR (126 MHz, $CDCl_3$): δ = 212.7, 176.1, 175.0, 167.3, 152.8, 131.9, 129.4, 129.1, 126.8, 107.3, 53.9, 49.4, 48.3, 42.2, 41.9, 38.7, 35.9, 26.8, 26.2, 21.3;

HRMS (ESI$^+$): Calculated for $C_{22}H_{21}NO_5$ $[M+H]^+$: 380.1493, found: 380.1485;

IR (thin film, cm^{-1}): 2957, 1708, 1349, 1179, 738 and 688.

7.10. DMAD Adduct 14

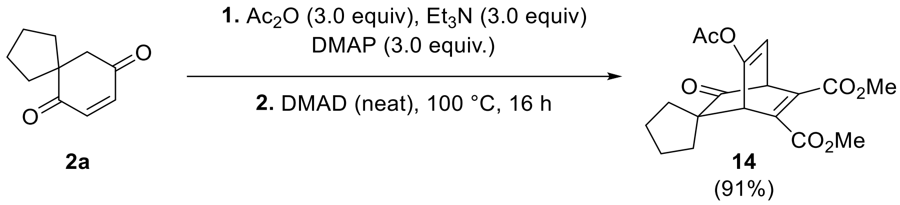

To a solution of enedione **2a** (164 mg, 1.0 mmol, 1.0 equiv.) in CH_2Cl_2 (5 mL) was added acetic anhydride (0.28 mL, 3.0 mmol, 3.0 equiv.), DMAP (37 mg, 0.30 mmol, 0.30 equiv.) and triethylamine (0.42 mL, 3.0 mmol, 3.0 equiv.). This mixture was stirred at room temperature for 20 h. Upon completion, the reaction was quenched by the addition of a solution of saturated aqueous NH_4Cl (5 mL). The mixture was transferred to a separating funnel containing H_2O (10 mL) and extracted with CH_2Cl_2 (3 × 10 mL). The organic phases were combined, washed with brine (10 mL), dried with anhydrous Na_2SO_4, filtered, and concentrated under reduced pressure to afford a crude residue. To this crude residue was added dimethyl acetylenedicarboxylate (2.5 mL, approx. 20 equiv.) and the resulting mixture has heated with stirring at 100 °C for 16 h. Upon completion, the reaction mixture was cooled to room temperature and loaded directly onto a flash chromatography column (20% EtOAc in *n*-hexane) to afford cycloadduct **14** (318 mg, 0.91 mmol, 91% yield) as a white solid.

R_f = 0.22 (20% EtOAc in *n*-hexane), stained with $KMnO_4$;

^{1}H NMR (500 MHz, $CDCl_3$): δ = 6.19 (dd, *J* = 6.7, 2.7 Hz, 1H), 4.33 (d, *J* = 6.7 Hz, 1H), 3.83 (s, 3H), 3.79 (s, 3H), 3.79 (d, *J* = 2.8 Hz, 1H), 2.19 (s, 3H), 1.84 – 1.70 (m, 5H), 1.70 – 1.63 (m, 1H), 1.56 – 1.47 (m, 2H);

^{13}C NMR (126 MHz, $CDCl_3$): δ = 207.6, 167.9, 165.6, 164.7, 156.9, 142.2, 139.2, 109.1, 55.9, 53.0, 52.9, 52.7, 49.7, 38.7, 38.6, 26.7, 26.4, 21.3;

HRMS (ESI$^+$): Calculated for $C_{18}H_{20}O_7Na$ $[M+Na]^+$: 371.1101, found: 371.1101;

IR (thin film, cm^{-1}): 2950, 1708, 1627, 1280, and 903.

7.10. PTAD Adduct 15

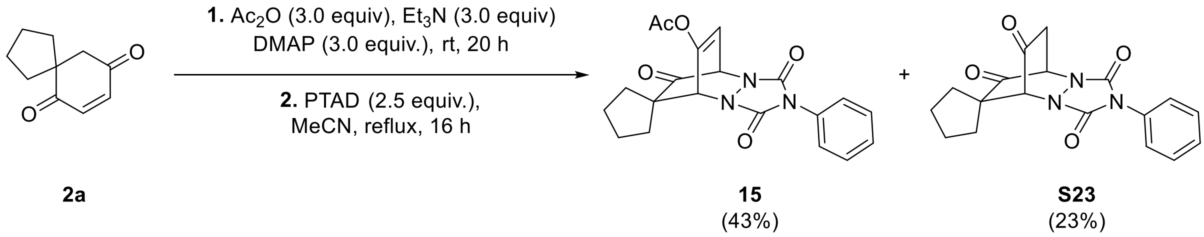

To a solution of enedione **2a** (0.164 g, 1.0 mmol, 1.0 equiv.) in CH_2Cl_2 (5 mL) was added acetic anhydride (0.28 mL, 3.0 mmol, 3.0 equiv.), DMAP (37 mg, 0.30 mmol, 0.30 equiv.) and triethylamine (0.42 mL, 3.0 mmol, 3.0 equiv.). This mixture was stirred at room temperature for 20 h. Upon completion, the reaction was quenched by the addition of a solution of saturated aqueous NH_4Cl (5 mL). The mixture was transferred to a separating funnel containing H_2O (10 mL) and extracted with CH_2Cl_2 (3 × 10 mL). The organic phases were combined, washed with brine (10 mL), dried with anhydrous Na_2SO_4, filtered, and concentrated under reduced pressure to afford a crude residue. This crude residue was dissolved in MeCN (5 mL) after which, 4-phenyl-1,2,4-triazole-3,5-dione (PTAD, 438 mg, 2.5 mmol, 2.5 equiv.) was added in a single portion. The reaction mixture was stirred under reflux for 16 h. Upon completion, the reaction was cooled to room temperature and concentrated under reduced pressure. The resulting crude residue was purified by flash column chromatography (50% EtOAc in *n*-hexane) to give adducts **15** (163 mg, 0.43 mmol, 43% yield) and **S23** (78 mg, 0.23 mmol, 23% yield) as colourless solids.

Data for **15**

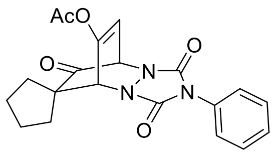

15

MP = 191–194 °C (acetone);

***R*f** = 0.45 (50% EtOAc in *n*-hexane), stained with $KMnO_4$;

^{1}H NMR (601 MHz, $CDCl_3$): δ = 7.50 – 7.45 (m, 4H), 7.42 – 7.37 (m, 1H), 6.17 (dd, *J* = 6.5, 2.8 Hz, 1H), 5.17 (d, *J* = 6.5 Hz, 1H), 4.64 (d, *J* = 2.8 Hz, 1H), 2.23 (s, 3H), 2.09 – 2.02 (m, 1H), 1.99 – 1.74 (m, 6H), 1.64 – 1.58 (m, 1H);

^{13}C NMR (126 MHz, $CDCl_3$): δ = 203.2, 167.1, 156.2, 155.8, 152.9, 131.2, 129.4, 128.8, 126.0, 107.6, 62.4, 61.6, 54.9, 37.0, 35.4, 26.6, 26.5, 21.1;

HRMS (ESI$^+$): Calculated for $C_{20}H_{19}N_3O_5Na$ [M+Na]$^+$: 404.1217, found: 404.1213;

IR (thin film, cm^{-1}): 1712, 1400, 1193 and 761.

Data for **S23**

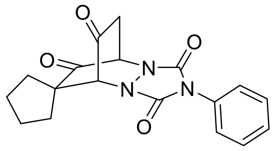

S23

$\boldsymbol{R_f}$ = 0.24 (50% EtOAc in *n*-hexane), stained with $KMnO_4$;

^{1}H NMR (500 MHz, $CDCl_3$): δ = 7.53 – 7.47 (m, 4H), 7.45 – 7.39 (m, 1H), 4.95 (dd, *J* = 3.4, 2.4 Hz, 1H), 4.55 (s, 1H), 3.04 (ddd, *J* = 19.6, 3.4, 0.7 Hz, 1H), 2.80 (dd, *J* = 19.5, 2.4 Hz, 1H), 2.11 – 2.02 (m, 1H), 2.00 – 1.87 (m, 4fH), 1.87 – 1.77 (m, 2H), 1.69 – 1.61 (m, 1H);

^{13}C NMR (126 MHz, $CDCl_3$): δ = 203.9, 195.7, 152.2, 151.6, 131.0, 129.5, 128.9, 125.6, 66.4, 58.6, 57.9, 39.3, 35.1, 34.5, 26.3, 26.0;

HRMS (ESI$^+$): Calculated for $C_{18}H_{18}N_3O_4$ [M+H]$^+$: 340.1292, found: 340.1293;

IR (thin film, cm^{-1}): 2866, 1706, 1414, 1034 and 761.

8. References

(1) Burkinshaw, G. F.; Davis, B. R.; Woodgate, P. D.; Hodges, R. The Isolation of a Spiran in the Acid-catalysed Rearrangement of a Bicyclic Cyclohexadienone. *Chem. Commun.* **1968**, 528. DOI: 10.1039/C19680000528.

(2) Burkinshaw, G. F.; Davis, B. R.; Hutchinson, E. G.; Woodgate P. D.; Hodges R. The synthesis and acid-catalysed rearrangements of 4-hydroxycyclohexa-2,5-dienones. *J. Chem. Soc. C* **1971**, 3002–3006. DOI: 10.1039/J39710003002.

(3) Liu, X.; Zhang, Y.; Li, B.; Zakharov, L. N.; Vasiliu, M.; Dixon, D. A.; Liu, S.-Y. A Modular Synthetic Approach to Monocyclic 1,4-Azaborines. *Angew. Chem. Int. Ed.* **2016**, *55*, 8333–8337. DOI: 10.1002/anie.201602840

(4) Ortin, G. G. D.; Salles Jr, A. G. Persulfate-promoted synthesis of biphenyl compounds in water from biomass-derived triacetic acid lactone**.** *Org. Biomol. Chem.* **2022**, *20*, 9292–9297. DOI: 10.1039/D2OB01900K.

(5) Carreño, M. C.; González-López, M.; Urbano, A. Oxidative De-aromatization of *para*-Alkyl Phenols into *para*-Peroxyquinols and *para*-Quinols Mediated by Oxone as a Source of Singlet Oxygen. *Angew. Chem. Int. Ed.* **2006**, *45*, 2737–2741. DOI: 10.1002/anie.200504605.

(6) Gu, J.; Rodriguez, K. X.; Kanda, Y.; Yang, S.; Ociepa, M.; Wilke, H.; Abrishami, A. V.; Jørgensen, L.; Skak-Nielsen, T.; Chen, J. S.; Baran, P. S. Convergent total synthesis of (+)-calcipotriol: A scalable, modular approach to vitamin D analogs. *Proc Natl Acad Sci USA* **2022**, e2200814119. DOI: 10.1073/pnas.2200814119.

(7) Liotta, D.; Saindane, M.; Barnum, C. Selective reactions of carbanions with *p*-quinones. The aggregate model. *J. Org. Chem.* **1981**, *46*, 3369–3370. DOI: 10.1021/jo00329a055.

(8) Carson, M. C.; Orzolek, B. J.; Kozlowski, M. C. Photocatalytic Synthesis of *para*-Peroxyquinols: Total Synthesis of (±)-Stemenone B and (±)-Parvistilbine B. *Org. Lett.* **2022**, *24*, 7250–7254. DOI: 10.1021/acs.orglett.2c02640.

(9) Liu, B.; Xu, Y.; Luo, Z.; Xie, J. Reductive Aromatization of Quinols with B_2pin_2 as Deoxidizing Agent. *Chem. Asian J.* **2020**, *15*, 1022–1024. DOI: 10.1002/asia.202000064.

(10) Milić, D.; Kop, T.; Csanadi, J.; Juranić , Z.; Zizak , Z.; Gašić, M.; Šolaja, B. Estrone derived steroidal diepoxide: Biologically active compound and precursor of a stable steroidal A,B-spiro system. *Steroids*, **2009**, *74*, 890–895. DOI: 10.1016/j.steroids.2009.06.002.

(11) Wu, J.; Zhang, J.; Soto-Acosta, R.; Mao, L.; Lian, J.; Chen, K.; Pillon, G.; Zhang, G.; Geraghty, R. J.; Zheng, S. One-Pot Synthesis of 1-Hydroxyacridones from *para*-Quinols and *ortho*-Methoxycarbonylaryl Isocyanates. *J. Org. Chem.* **2020**, *85*, 4515–4524. DOI: 10.1021/acs.joc.9b03307.

(12) Koszelewski, D.; Paprocki, D.; Brodzka, A.; Kęciek, A.; Wilk, M.; Ostaszewski, R.; The sustainable copper-catalyzed direct formation of highly functionalized p-quinols in water. *Sustain. Chem. Pharm.* **2022**, *25*, 100576. DOI: 10.1016/j.scp.2021.100576.

(13) Rao, J.; Zhao, J.; Zhu, X.; Wang, C.; Zhou, C.-Y. Rhodium-catalyzed reaction of diazoquinones with allylboronates to synthesize allylphenols. *Org. Chem. Front.* **2022**,*9*, 3677–3683. DOI: 10.1039/D2QO00626J.

(14) Tavanti, M.; Parmeggiani, F.; Castellanos, R. G.; Mattevi, A.; Turner, N. J. One-Pot Biocatalytic Double Oxidation of α-Isophorone for the Synthesis of Ketoisophorone. *ChemCatChem* **2017**, *9*, 3338–3348. DOI: 10.1002/cctc.201700620.

(15) Planas, A.; Tomás, J.; Bonet, J.-J. Spiran isolation in the dienone-phenol rearrangement of steroidal *p*-quinols. *Tetrahedron Lett.* **1987**, *28*, 471–474. DOI: 10.1016/S0040-4039(00)95759-9.

(16) Yates, P.; Burnell, D. J.; Freer, V. J.; Sawyer, J. F**.** Synthesis of cedranoid sesquiterpenes. III. Functionalization at carbon 4. *Can. J. Chem.* **1987**, *65*, 69–77. DOI: 10.1139/v87-012.

(17) Xie, W.-B.; Li, Z. Bis(μ-oxo)–Dititanium(IV)–Chiral Binaphthyldisulfonate Complexes for Highly Enantioselective Intramolecular Hydroalkoxylation of Nonactivated Alkenes**.** *ACS Catal.* **2021**, *11*, 6270–6275. 10.1021/acscatal.1c01146

(18) Moffett, R. B. Cyclopentadiene and 3-Chlorocyclopentadiene. *Org. Synth.* **1952**, *32*, 41. DOI: 10.15227/orgsyn.032.0041

9. NMR Spectra

9.1. Biaryl compounds

9.1.1. ^{1}H NMR Spectrum of Compound S3 (500 MHz, $CDCl_3$):

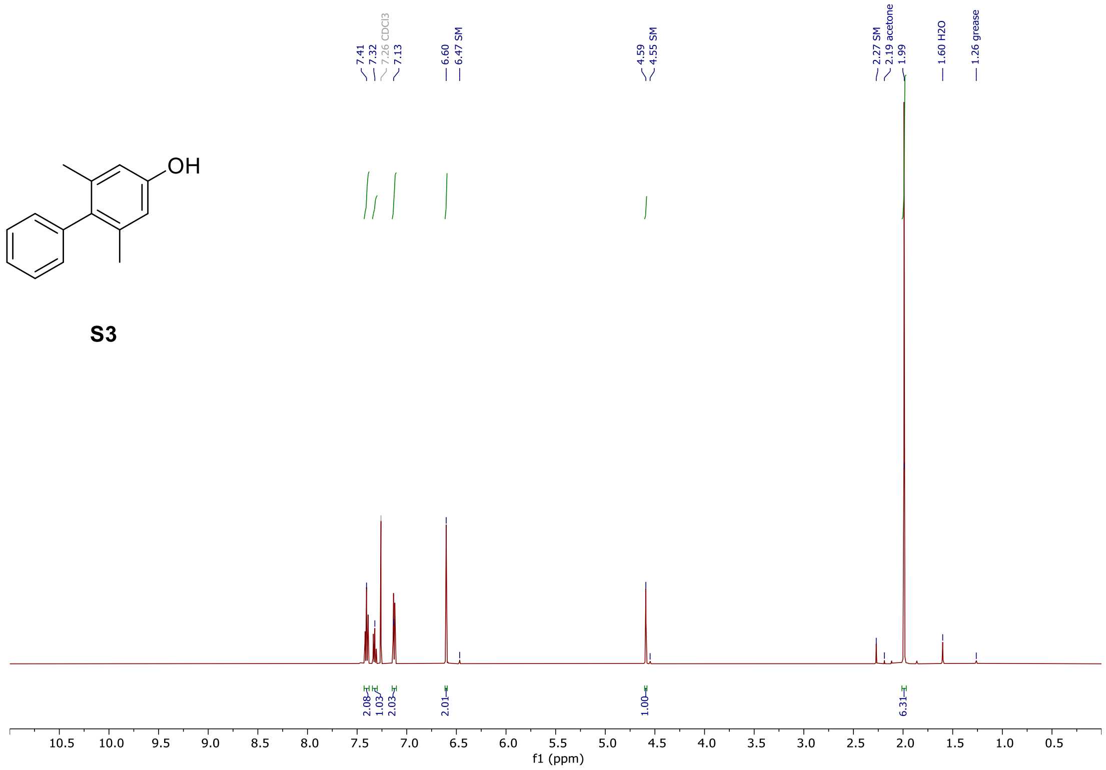

S3

9.1.2. ^{13}C NMR Spectrum of Compound S3 (126 MHz, $CDCl_3$):

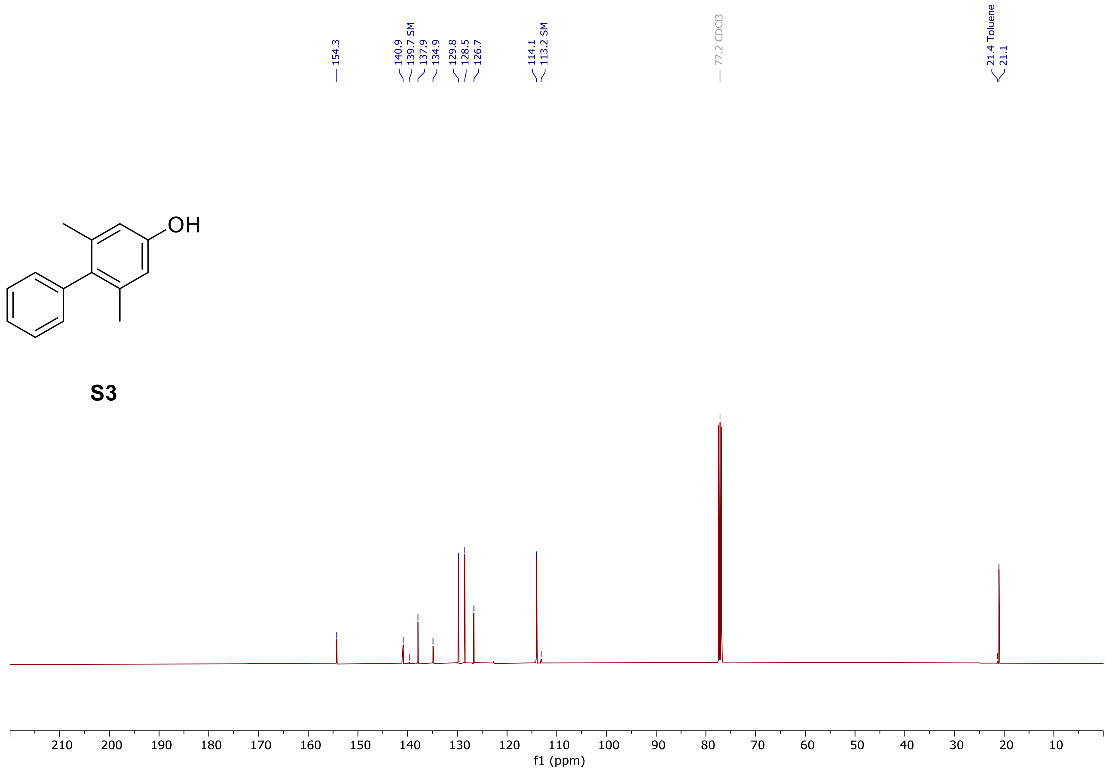

S3

9.1.3. ^{1}H NMR Spectrum of Compound S5 (500 MHz, $CDCl_3$):

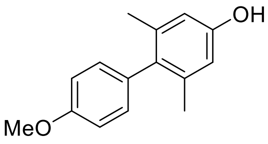

S5

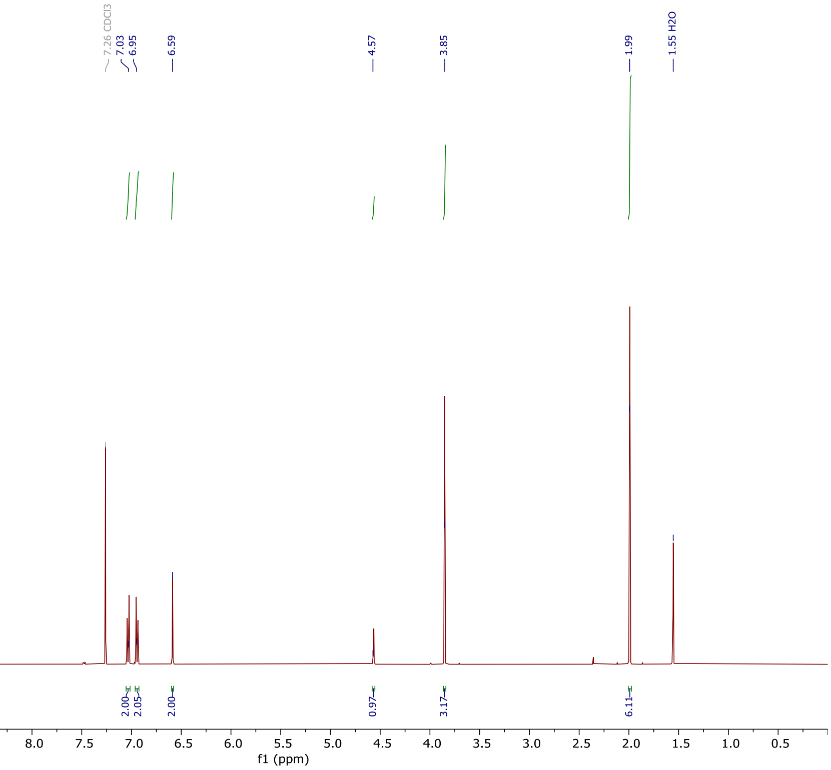

9.1.4. ^{13}C NMR Spectrum of Compound S5 (126 MHz, $CDCl_3$):

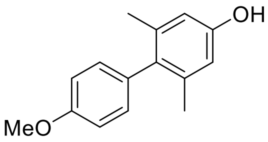

S5

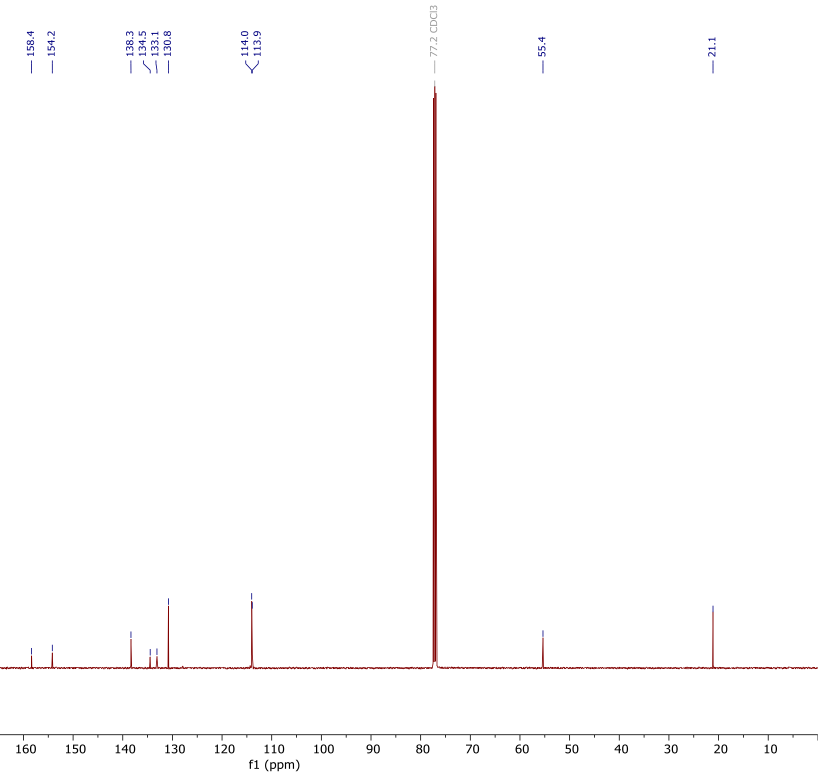

9.1.5. ^{1}H NMR Spectrum of Compound S7 (500 MHz, $CDCl_3$):

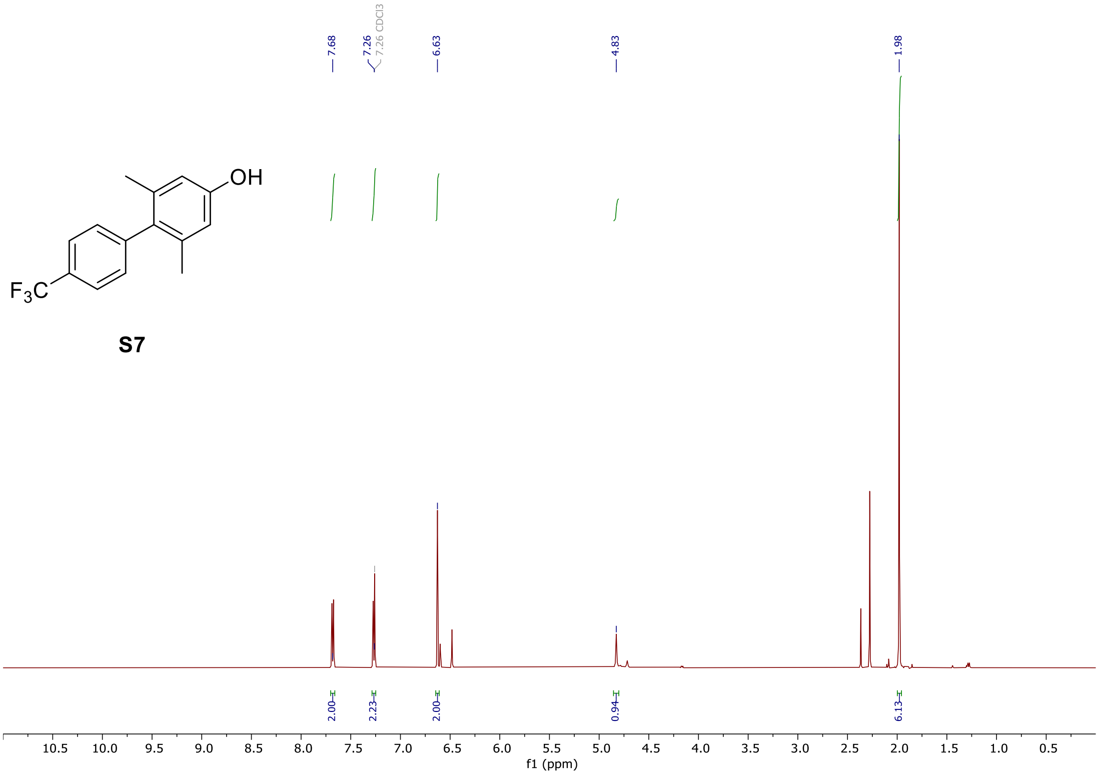

9.1.6. ^{13}C NMR Spectrum of Compound S7 (126 MHz, $CDCl_3$):

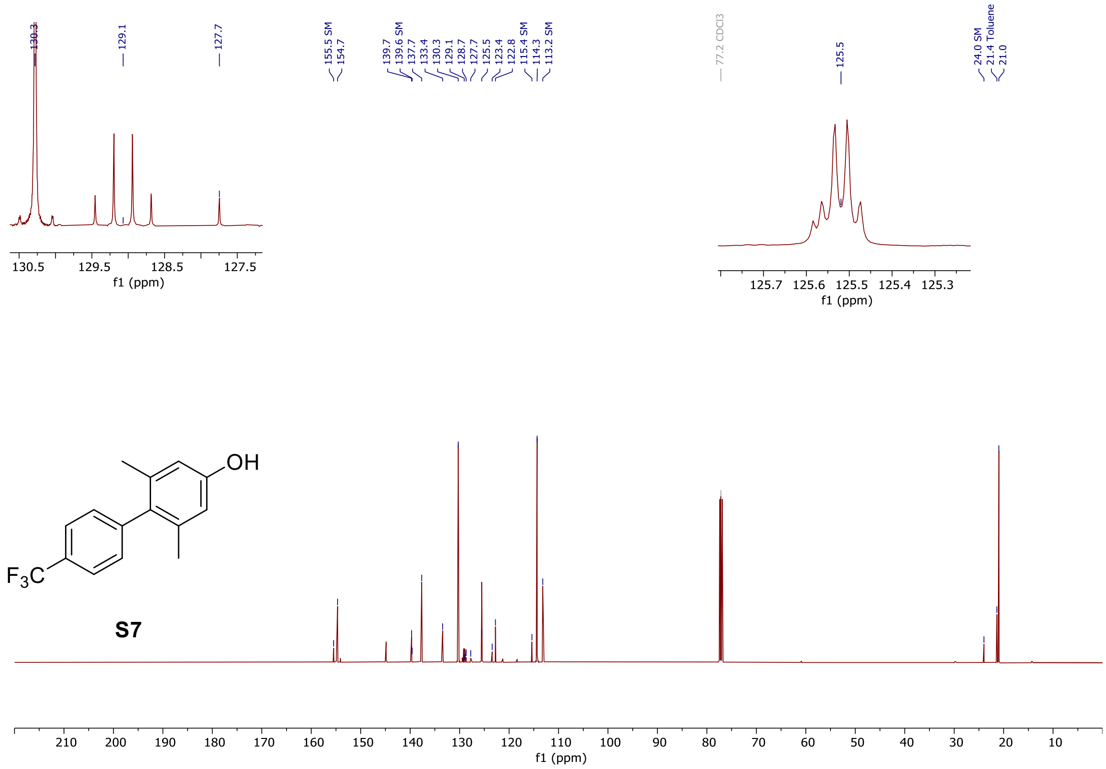

9.1.7. ^{19}F NMR Spectrum of Compound S7 (471 MHz, $CDCl_3$):

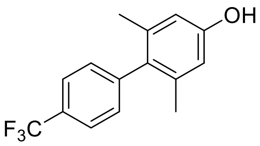

S7

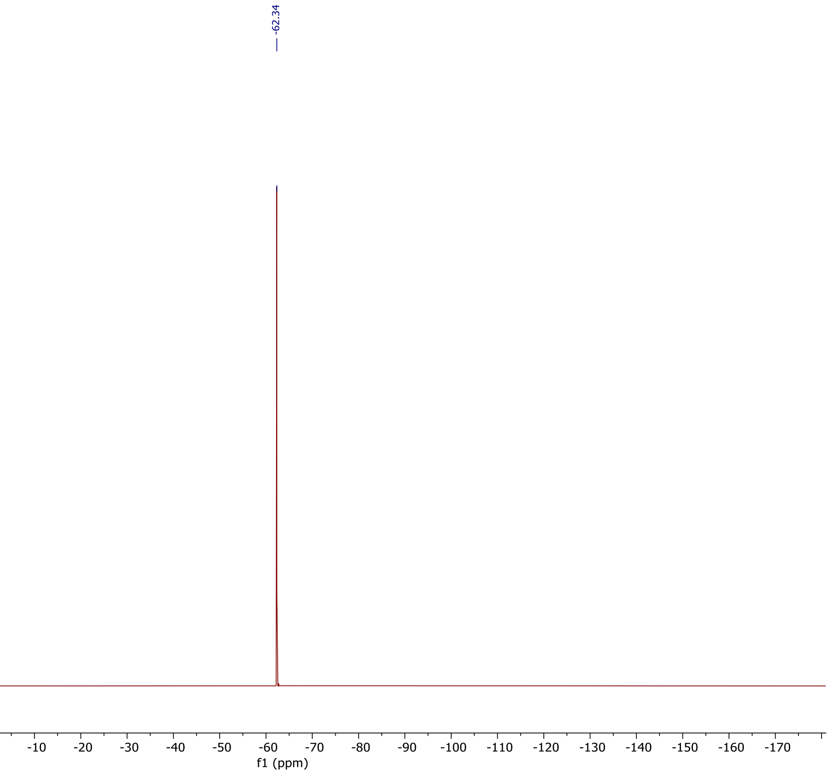

9.2. *p*-Quinols

9.2.1. ^{1}H NMR Spectrum of Compound 1a (500 MHz, $CDCl_3$):

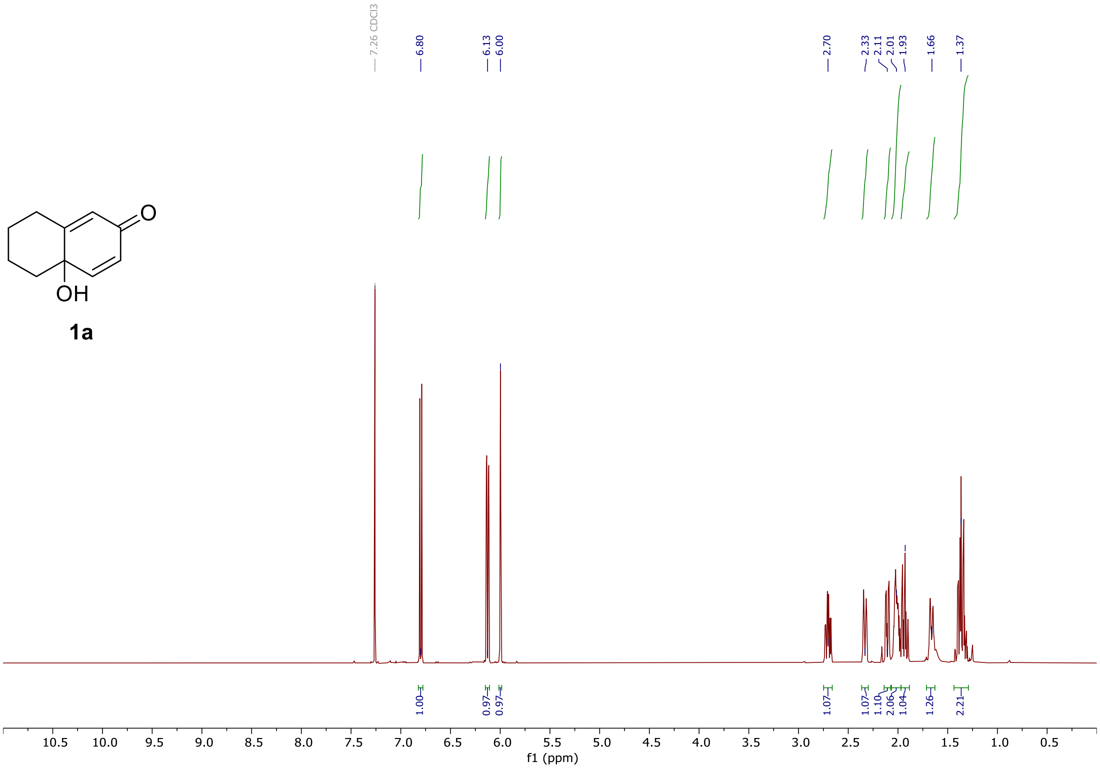

9.2.2. ^{13}C NMR Spectrum of Compound 1a (126 MHz, $CDCl_3$):

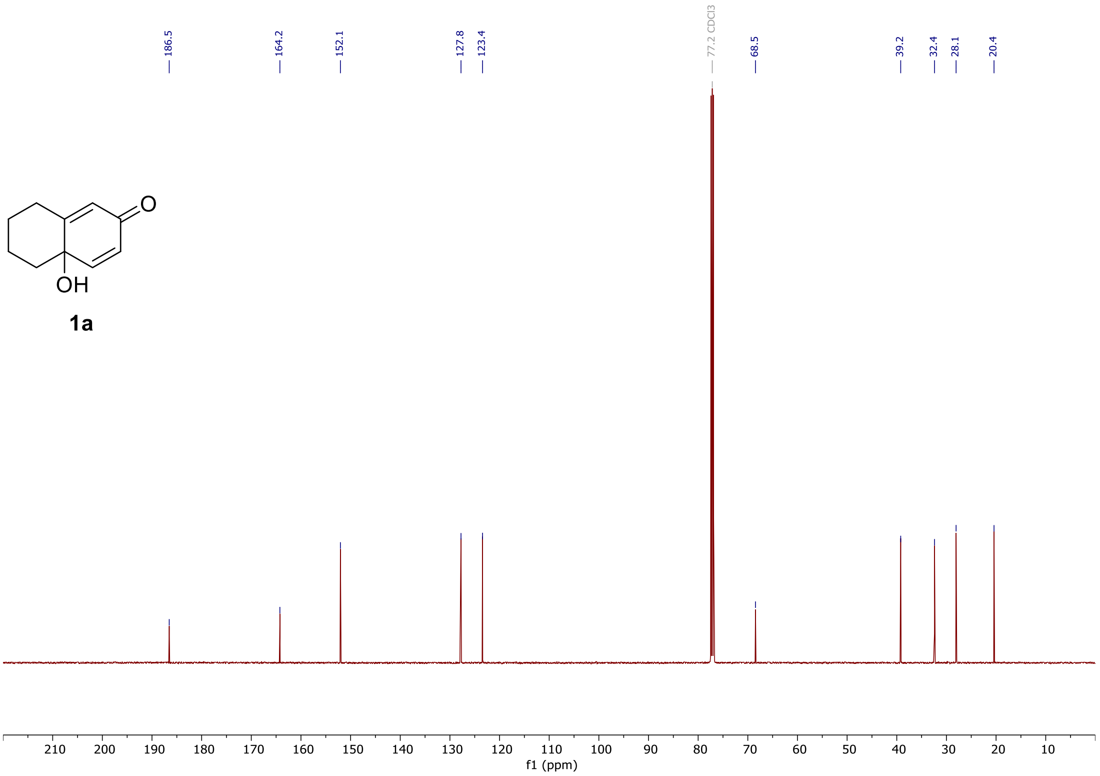

9.2.3. ^{1}H NMR Spectrum of Compound 1b (500 MHz, $CDCl_3$):

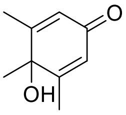

1b

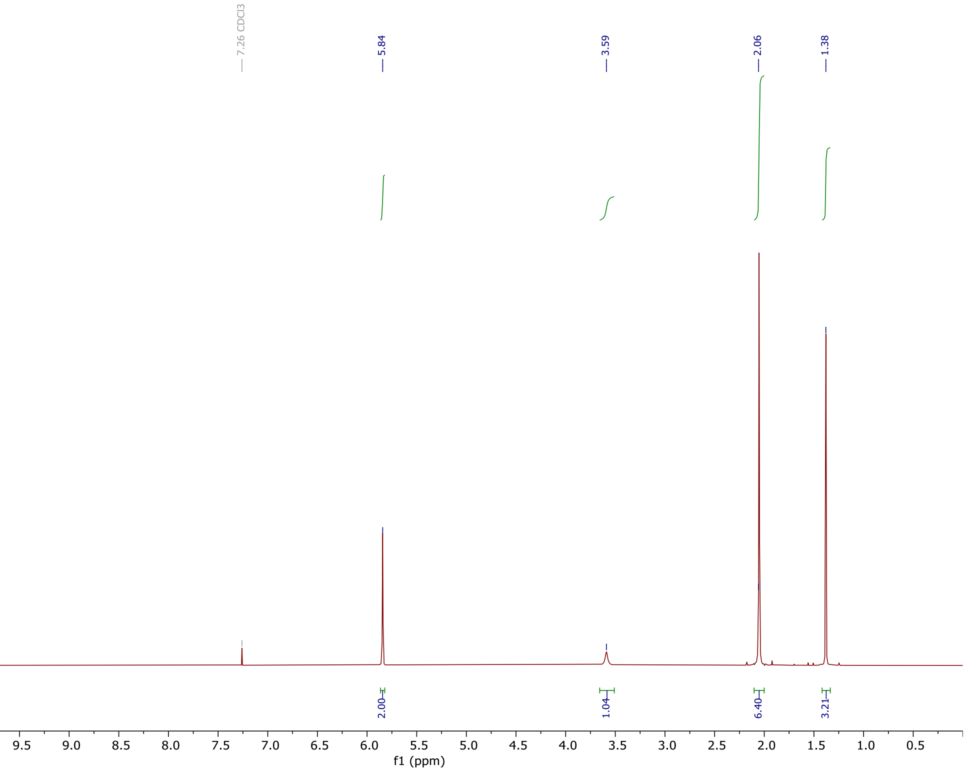

9.2.4. ^{13}C NMR Spectrum of Compound 1b (126 MHz, $CDCl_3$):

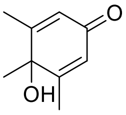

1b

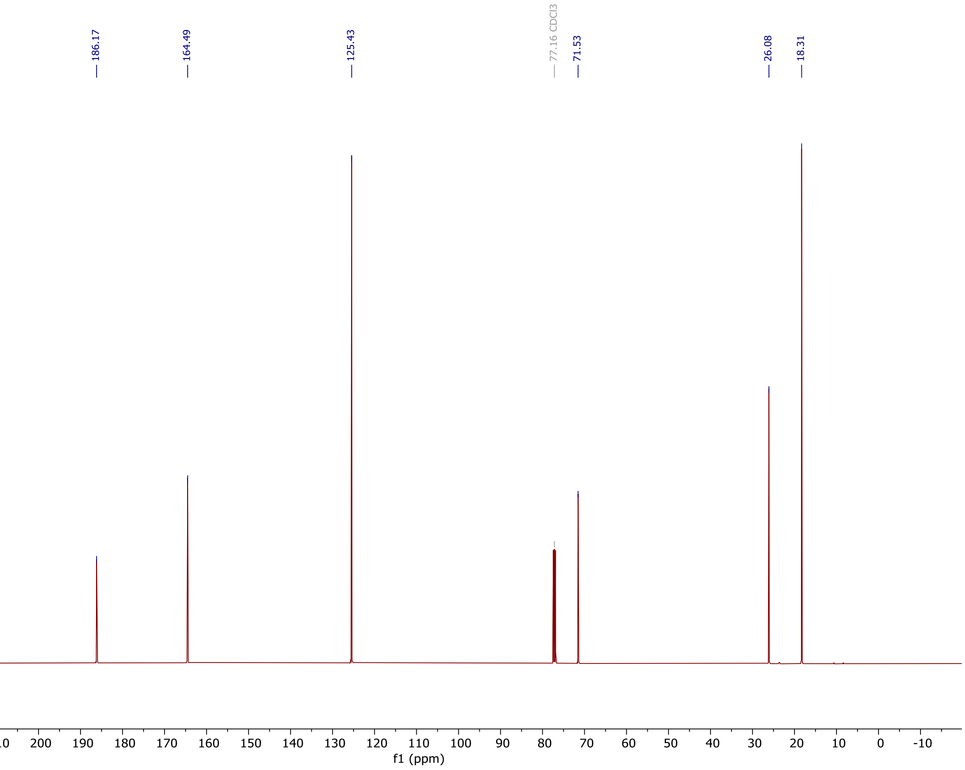

9.2.5. ^{1}H NMR Spectrum of Compound 1c (500 MHz, $CDCl_3$):

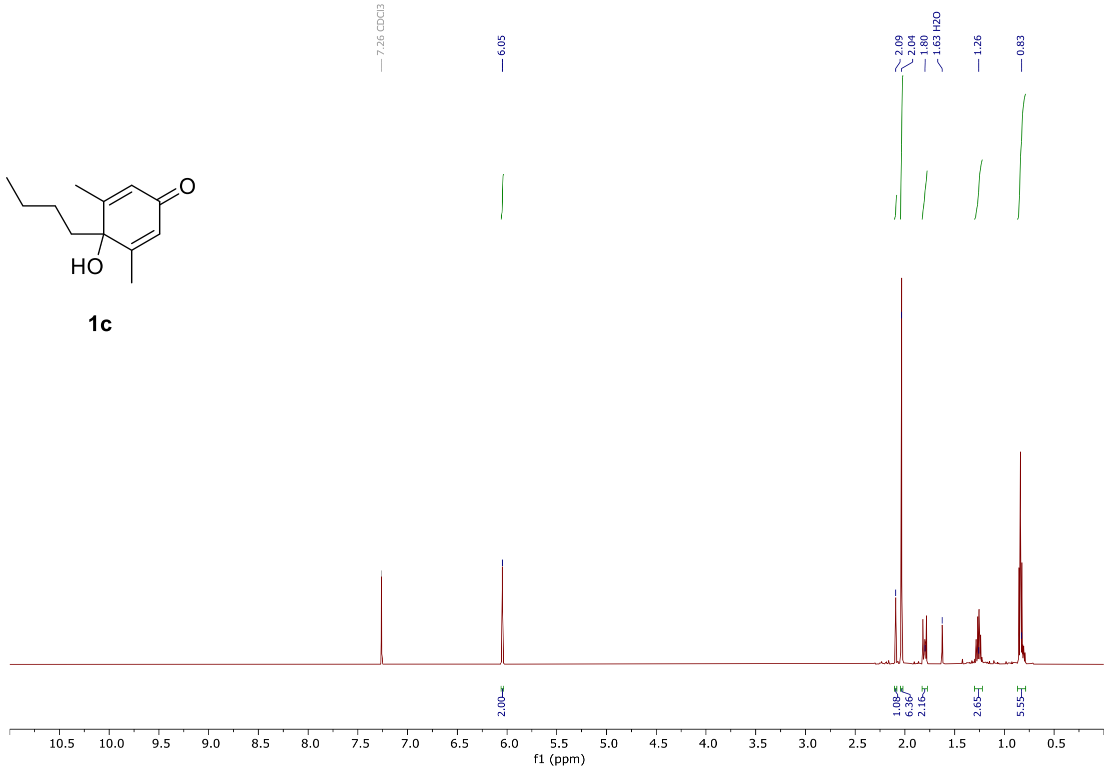

9.2.6. ^{13}C NMR Spectrum of Compound 1c (126 MHz, $CDCl_3$):

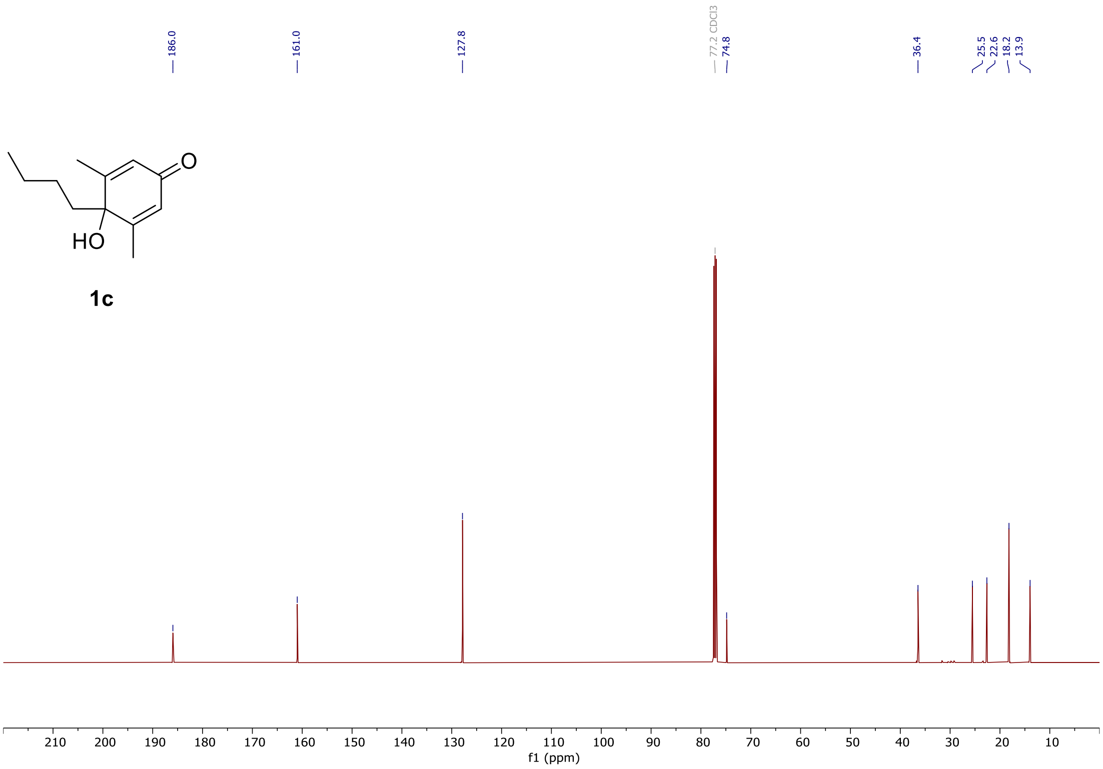

9.2.7. ^{1}H NMR Spectrum of Compound 1d (500 MHz, $CDCl_3$):

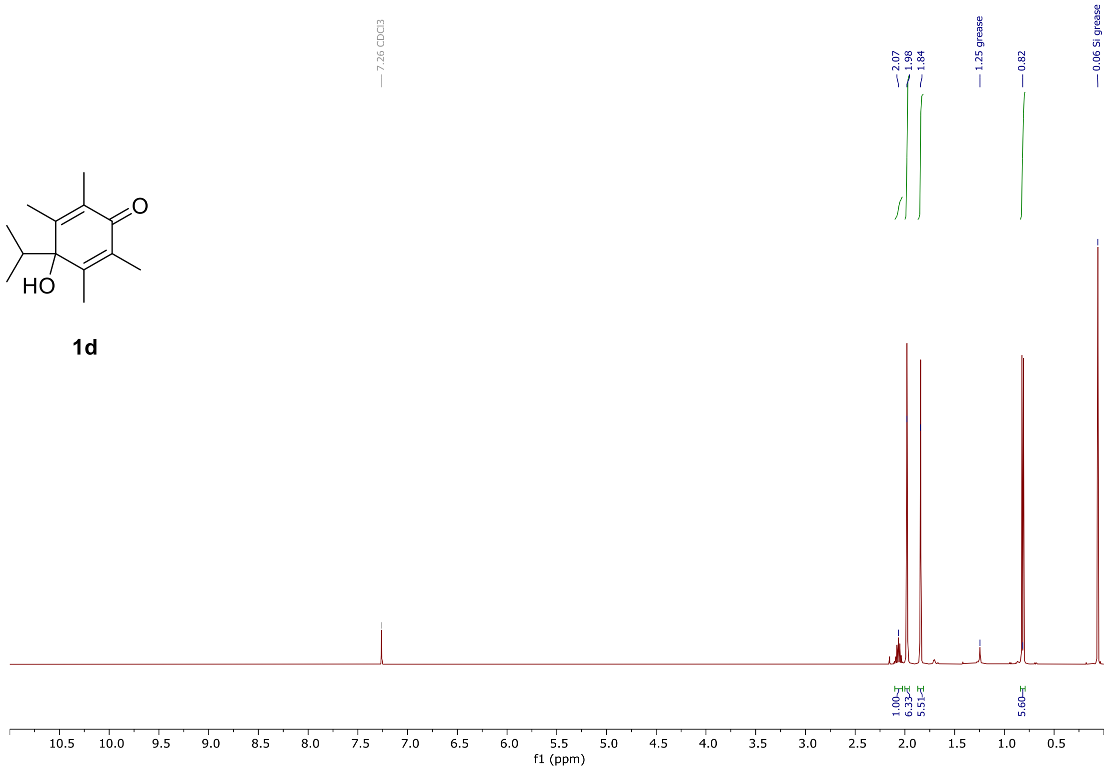

9.2.8. ^{13}C NMR Spectrum of Compound 1d (126 MHz, $CDCl_3$):

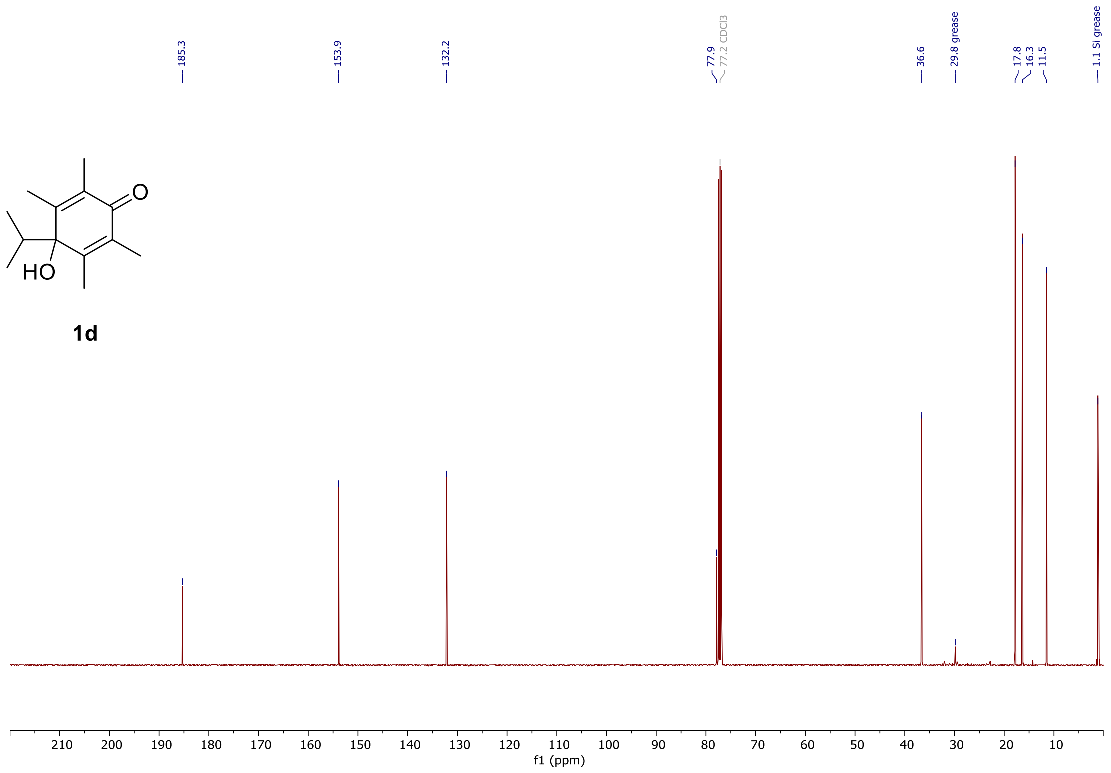

9.2.9. ^{1}H NMR Spectrum of Compound 1e (500 MHz, $CDCl_3$):

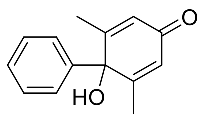

1e

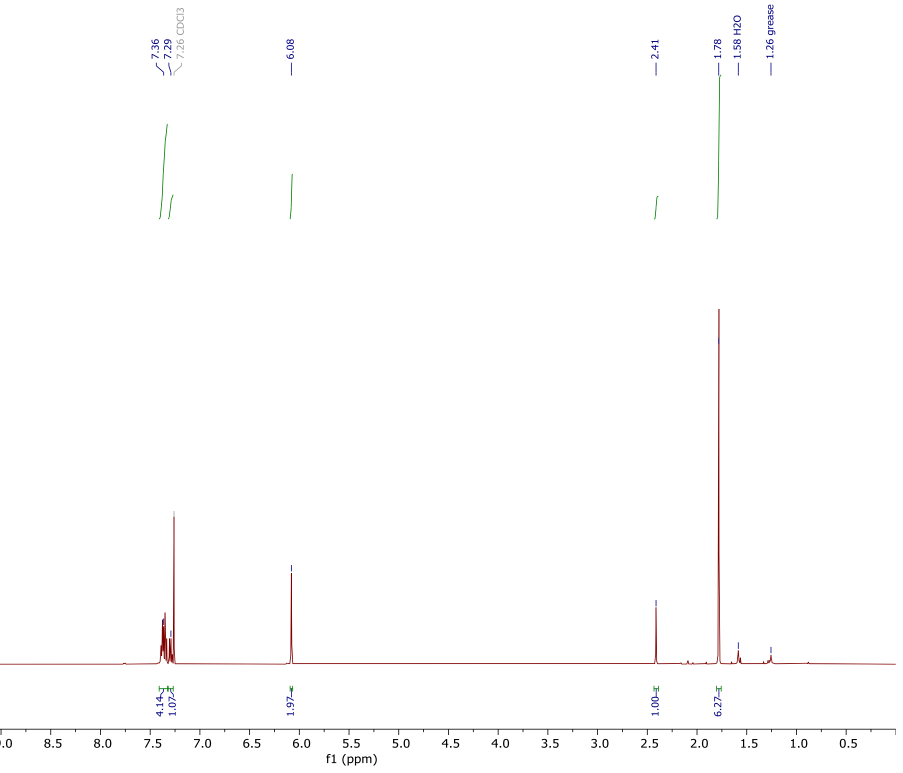

9.2.10. ^{13}C NMR Spectrum of Compound 1e (126 MHz, $CDCl_3$):

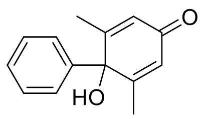

1e

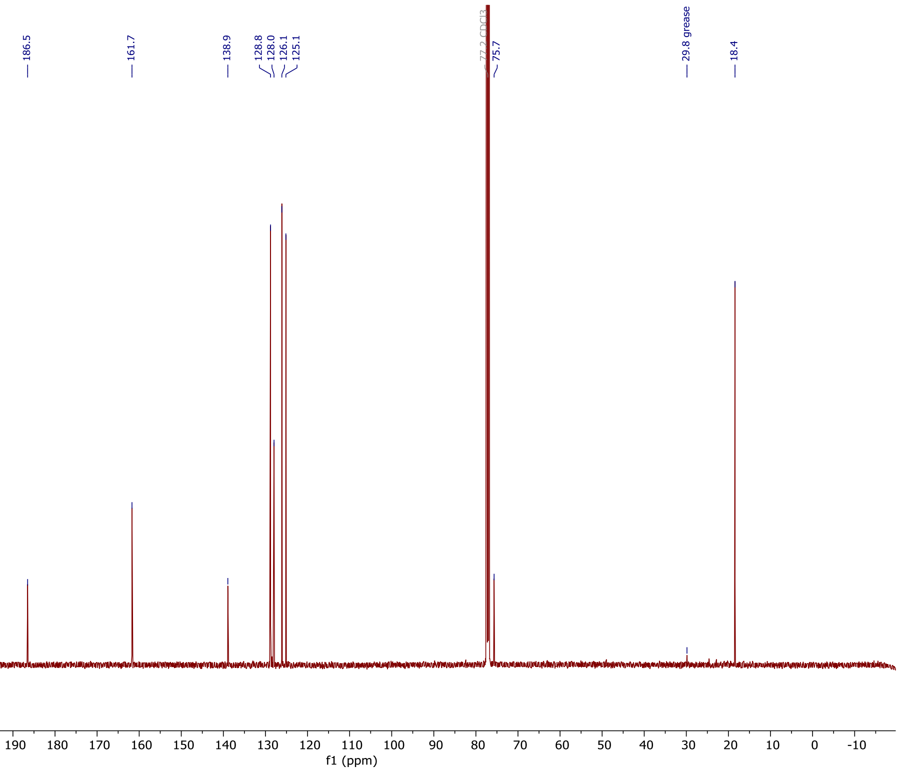

9.2.11. ^{1}H NMR Spectrum of Compound 1f (500 MHz, $CDCl_3$):

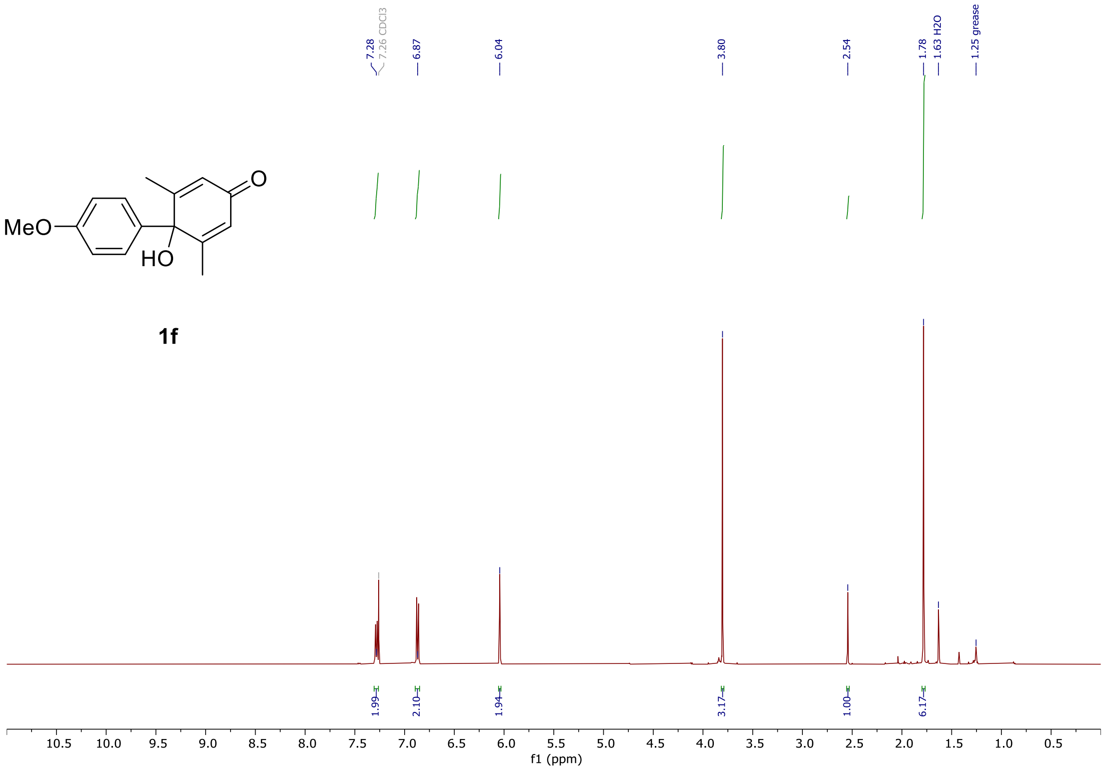

9.2.12. ^{13}C NMR Spectrum of Compound 1f (126 MHz, $CDCl_3$):

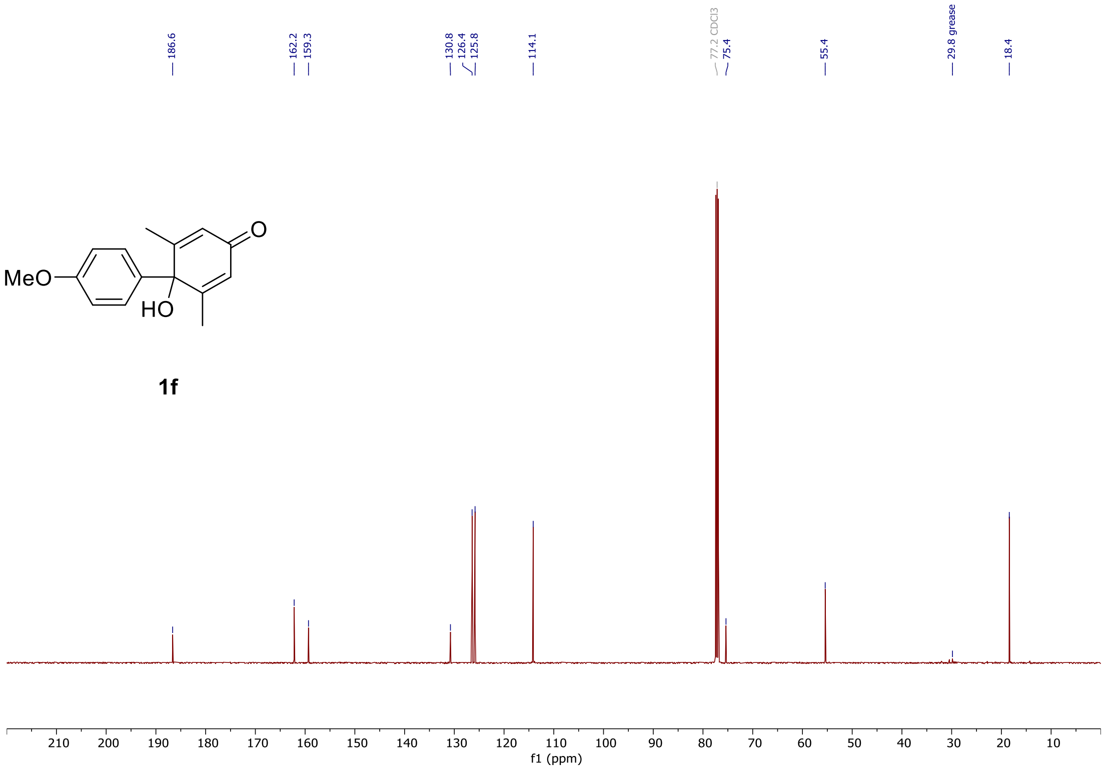

9.2.13. ^{1}H NMR Spectrum of Compound 1g (500 MHz, $CDCl_3$):

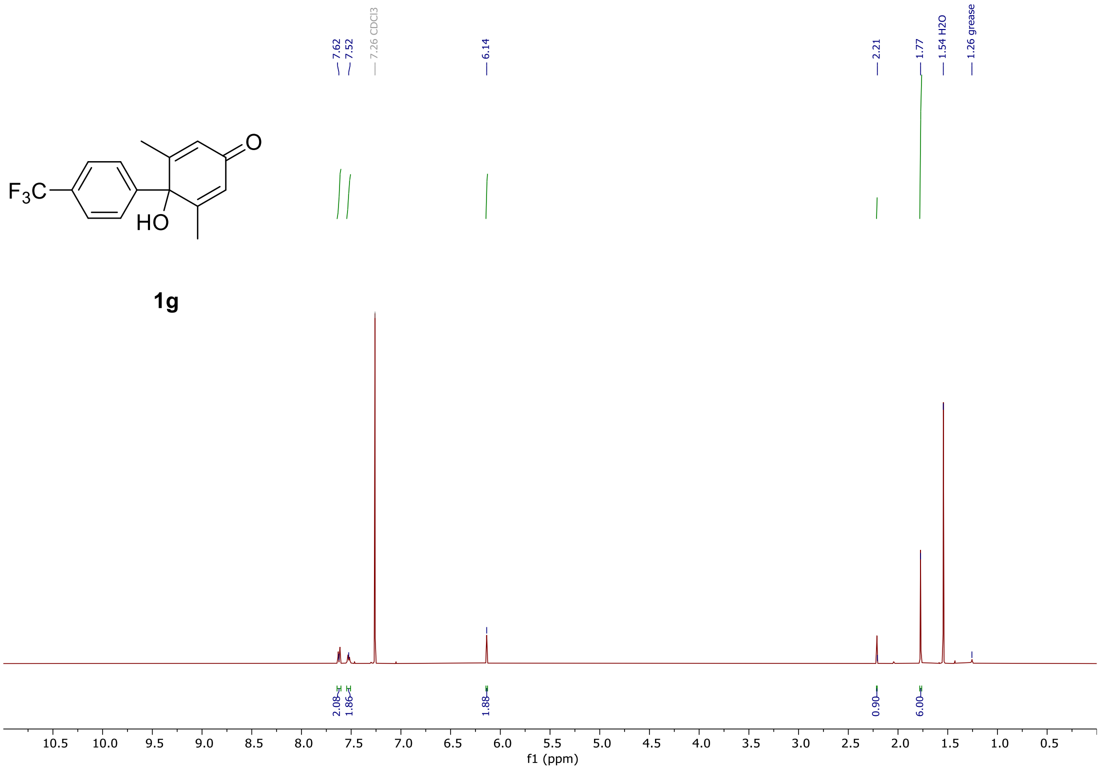

9.2.14. ^{13}C NMR Spectrum of Compound 1g (126 MHz, $CDCl_3$):

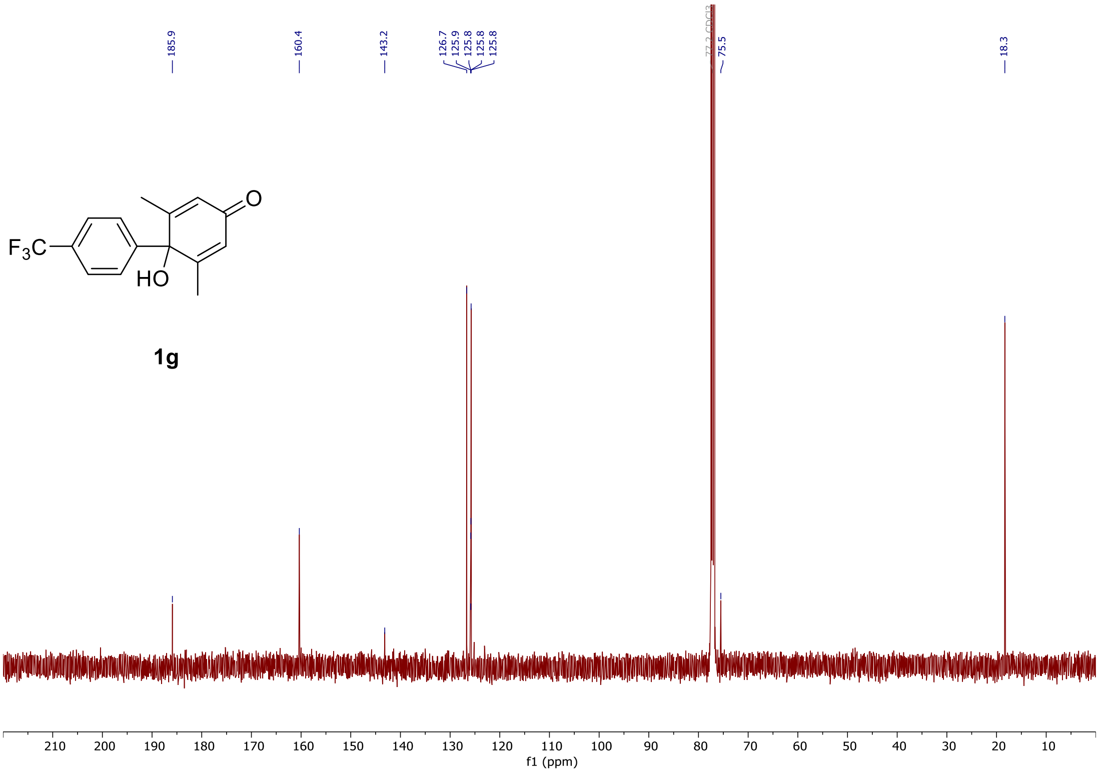

9.2.15. ^{19}F NMR Spectrum of Compound 1g (471 MHz, $CDCl_3$):

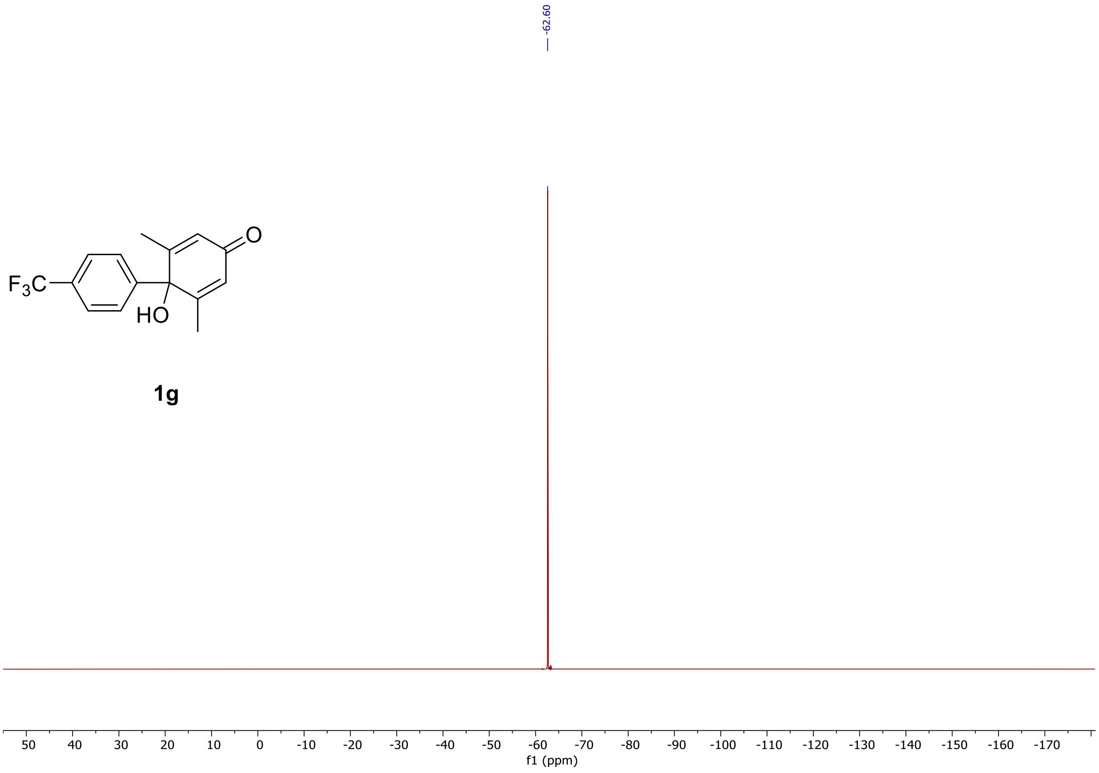

9.2.16. ^1H NMR Spectrum of Compound 1h (500 MHz, CD_3OD):

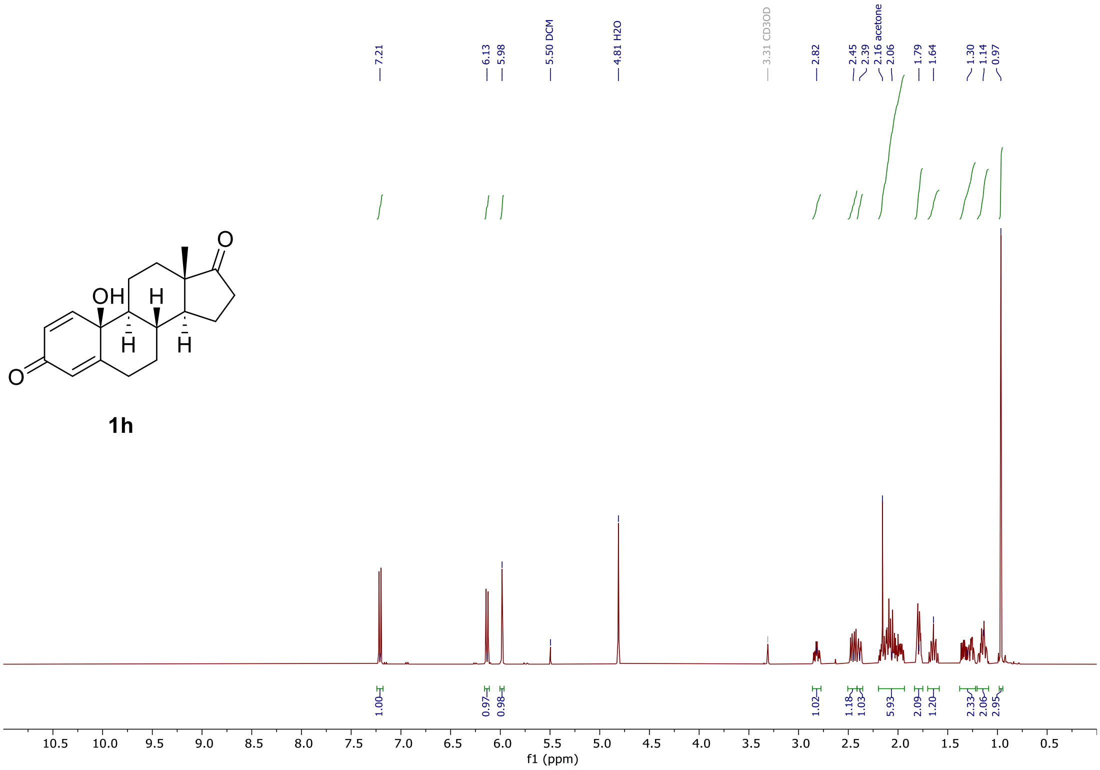

9.2.17. ^{13}C NMR Spectrum of Compound 1h (126 MHz, CD_3OD):

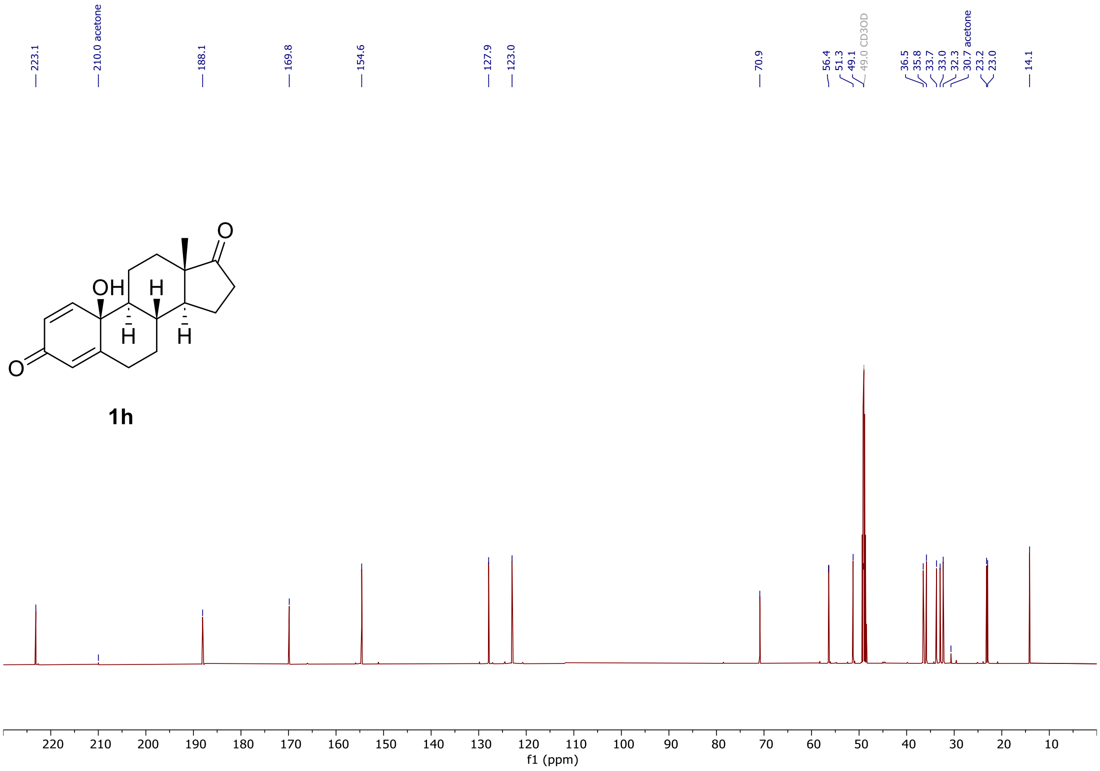

9.2.18. ^{1}H NMR Spectrum of Compound 1i (500 MHz, $CDCl_3$):

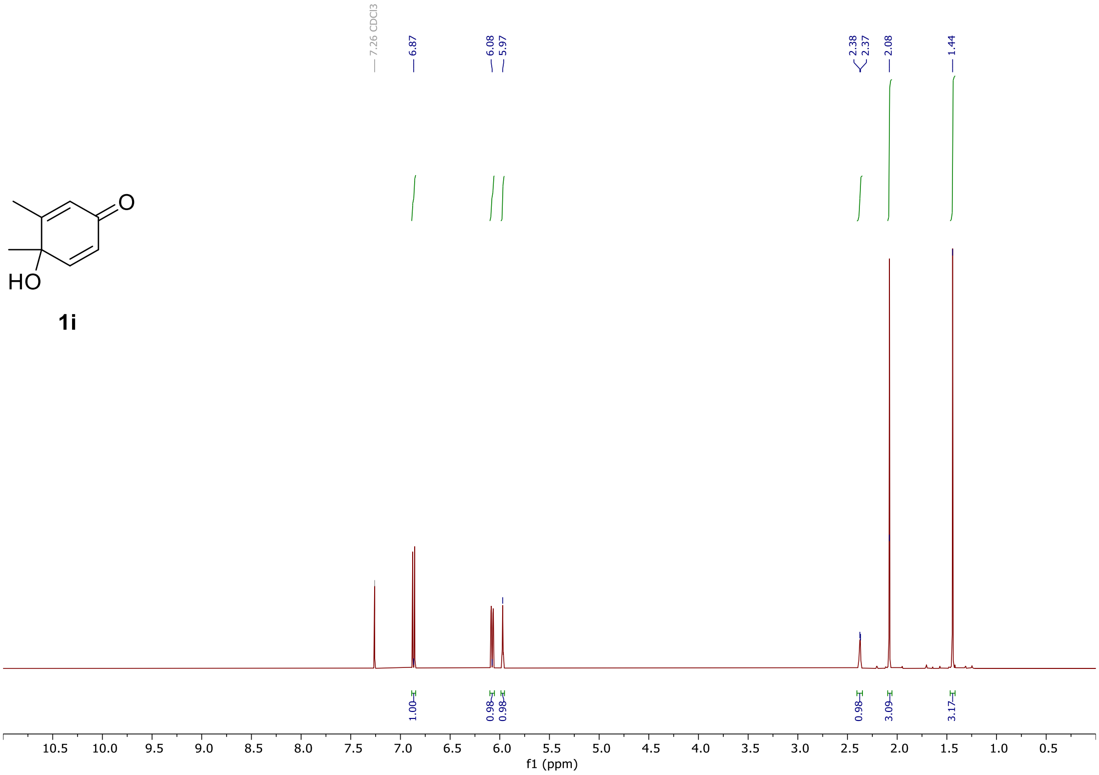

9.2.19. ^{13}C NMR Spectrum of Compound 1i (126 MHz, $CDCl_3$):

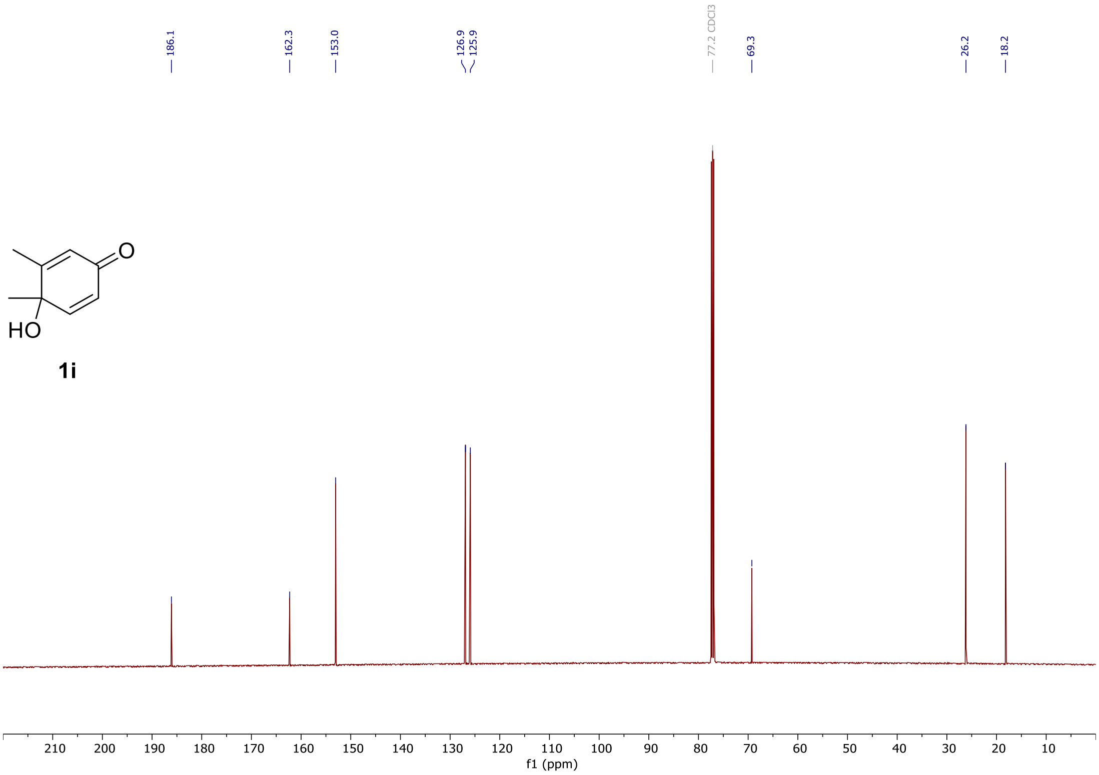

9.2.20. ^{1}H NMR Spectrum of Compound 1j (500 MHz, $CDCl_3$):

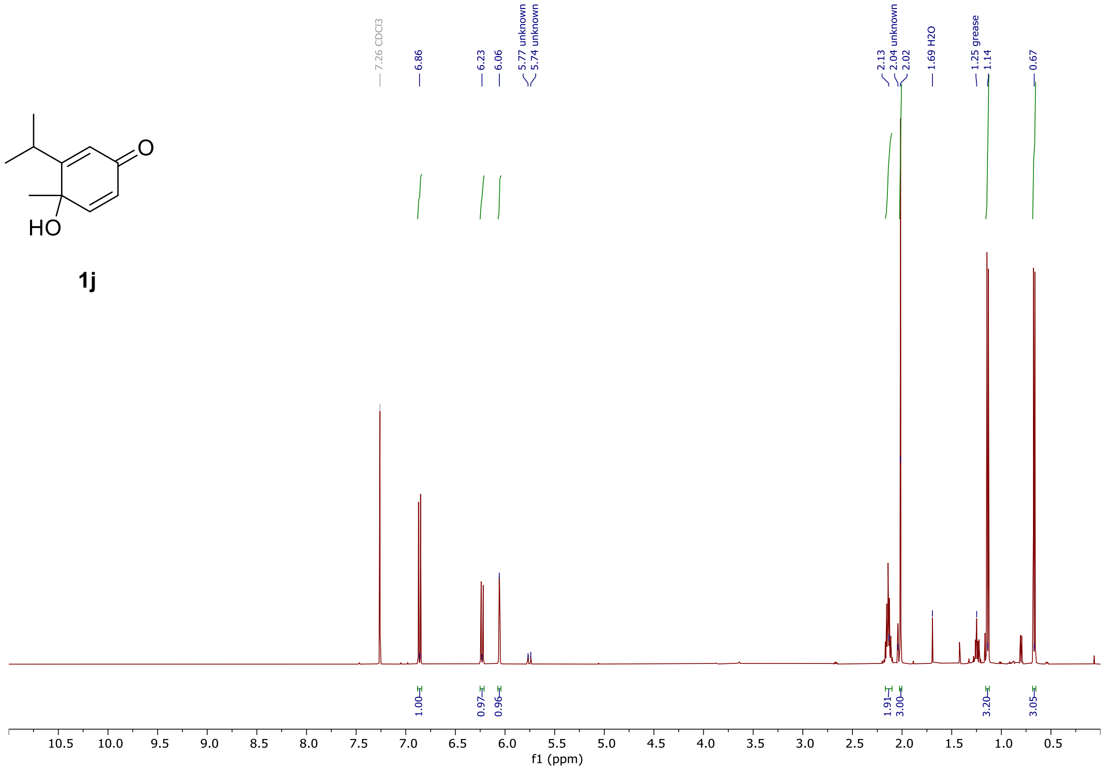

9.2.21. ^{13}C NMR Spectrum of Compound 1j (126 MHz, $CDCl_3$):

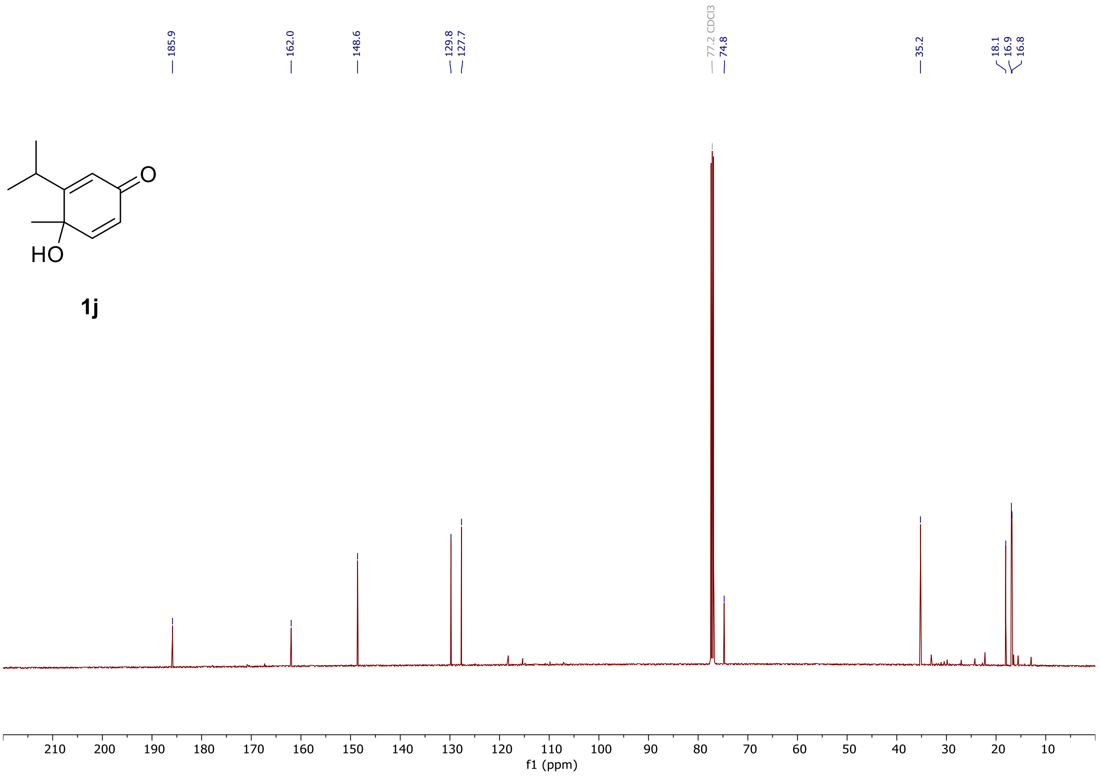

9.2.22. ^{1}H NMR Spectrum of Compound 1k (500 MHz, $CDCl_3$):

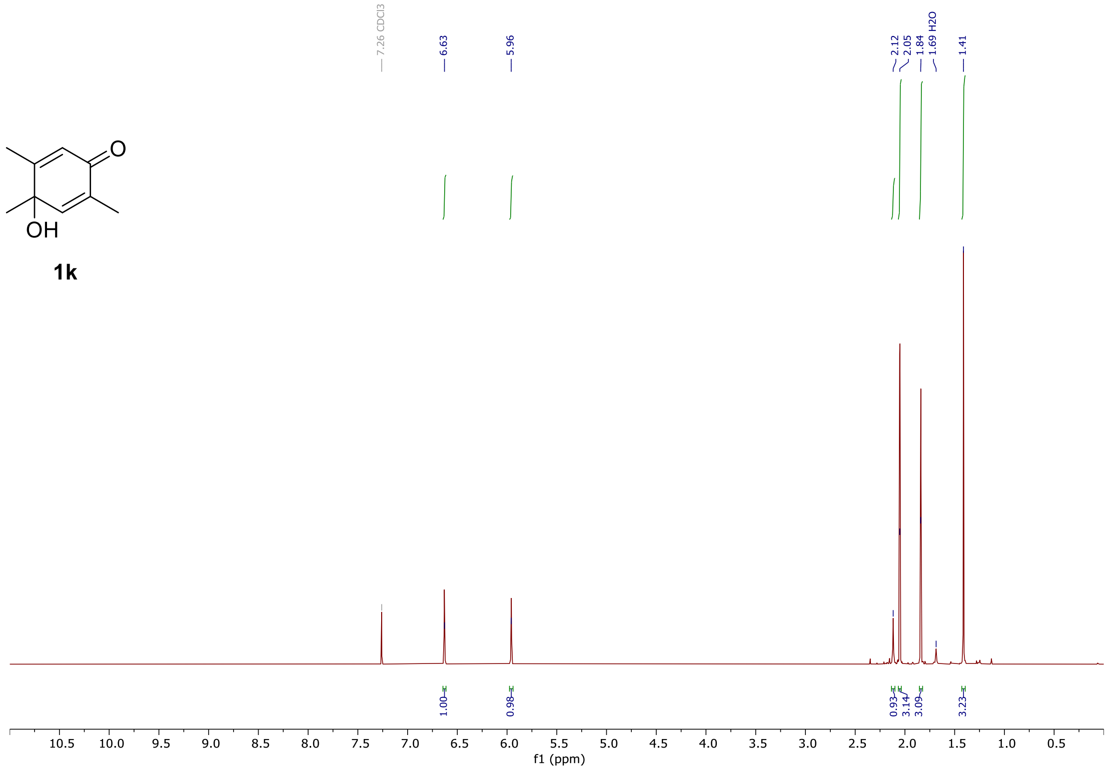

9.2.23. ^{13}C NMR Spectrum of Compound 1k (126 MHz, $CDCl_3$):

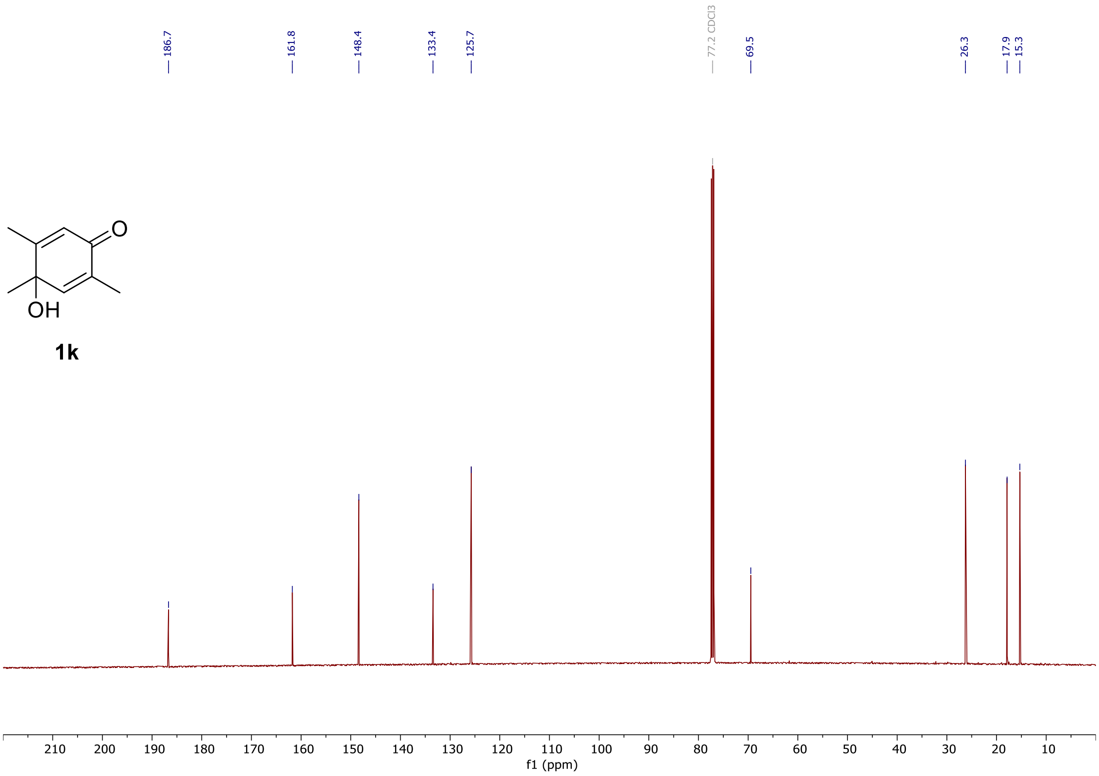

9.2.24. ^{1}H NMR Spectrum of Compound 1l (500 MHz, $CDCl_3$):

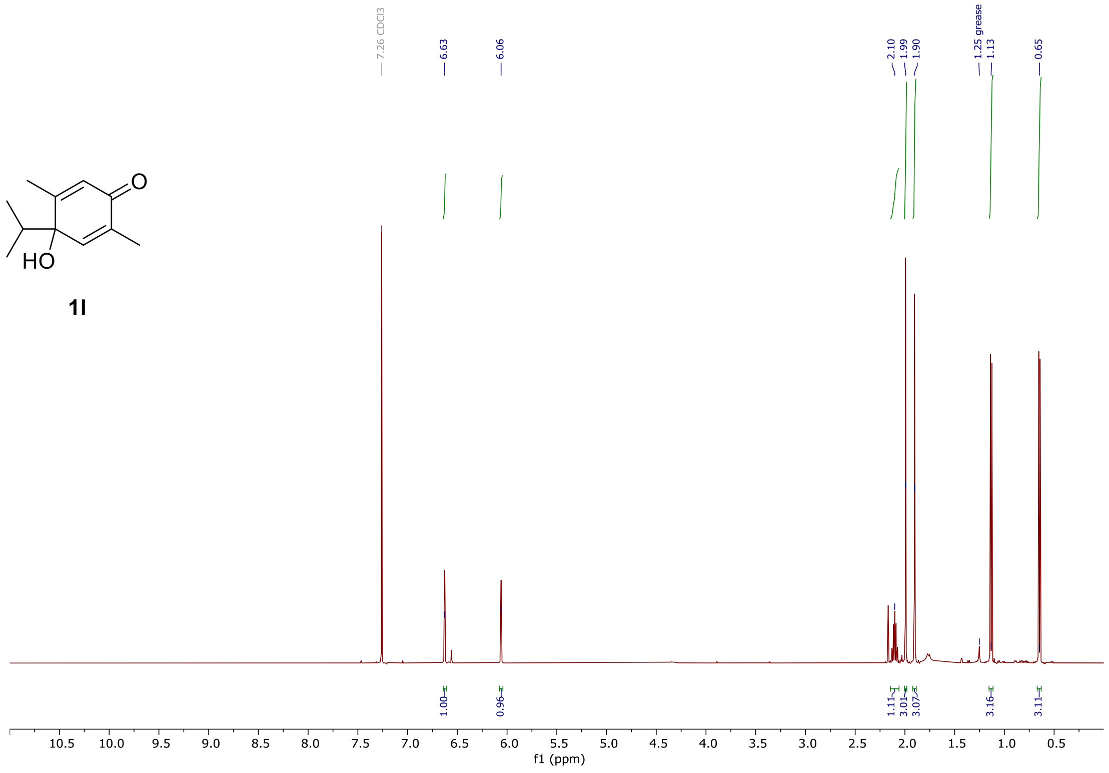

9.2.25. ^{13}C NMR Spectrum of Compound 1l (126 MHz, $CDCl_3$):

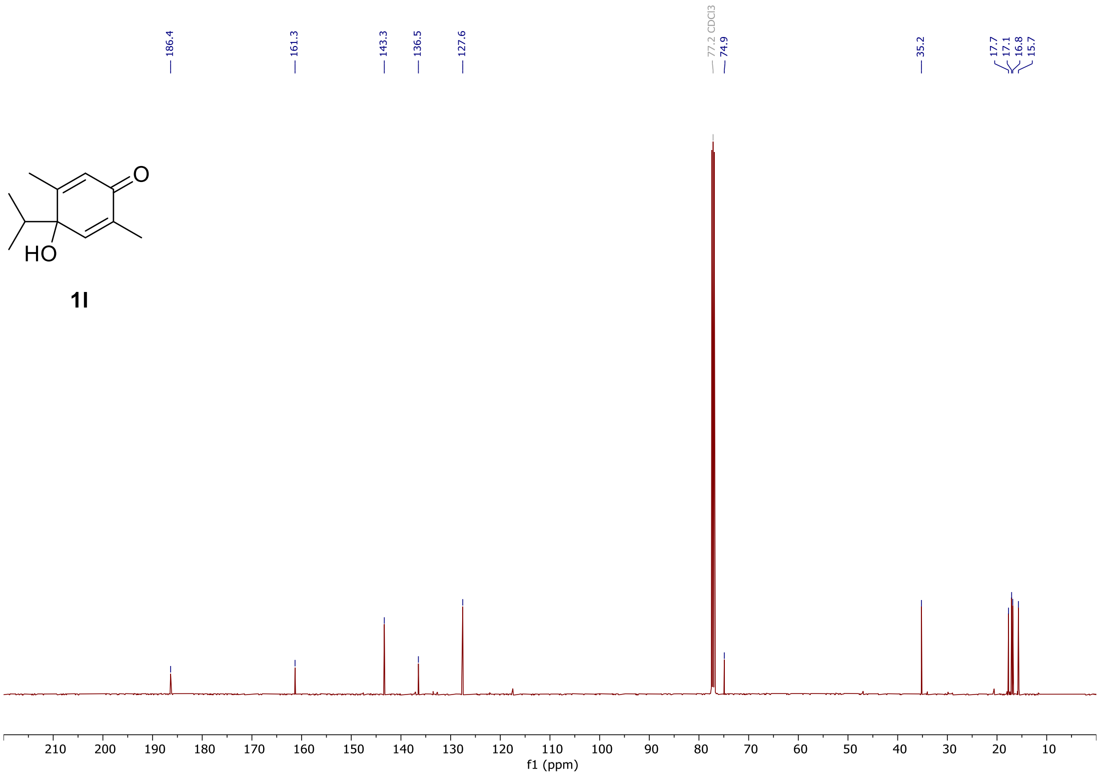

9.2.26. ^{1}H NMR Spectrum of Compound 1m (500 MHz, $CDCl_3$):

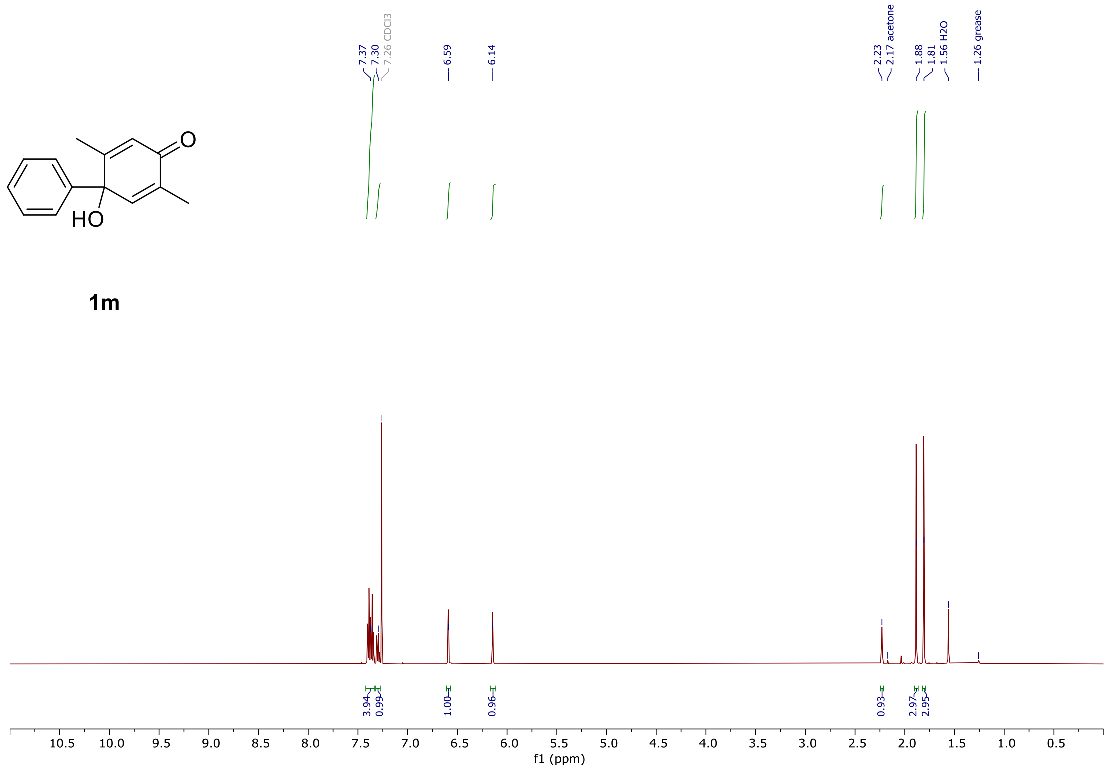

9.2.27. ^{13}C NMR Spectrum of Compound 1m (126 MHz, $CDCl_3$):

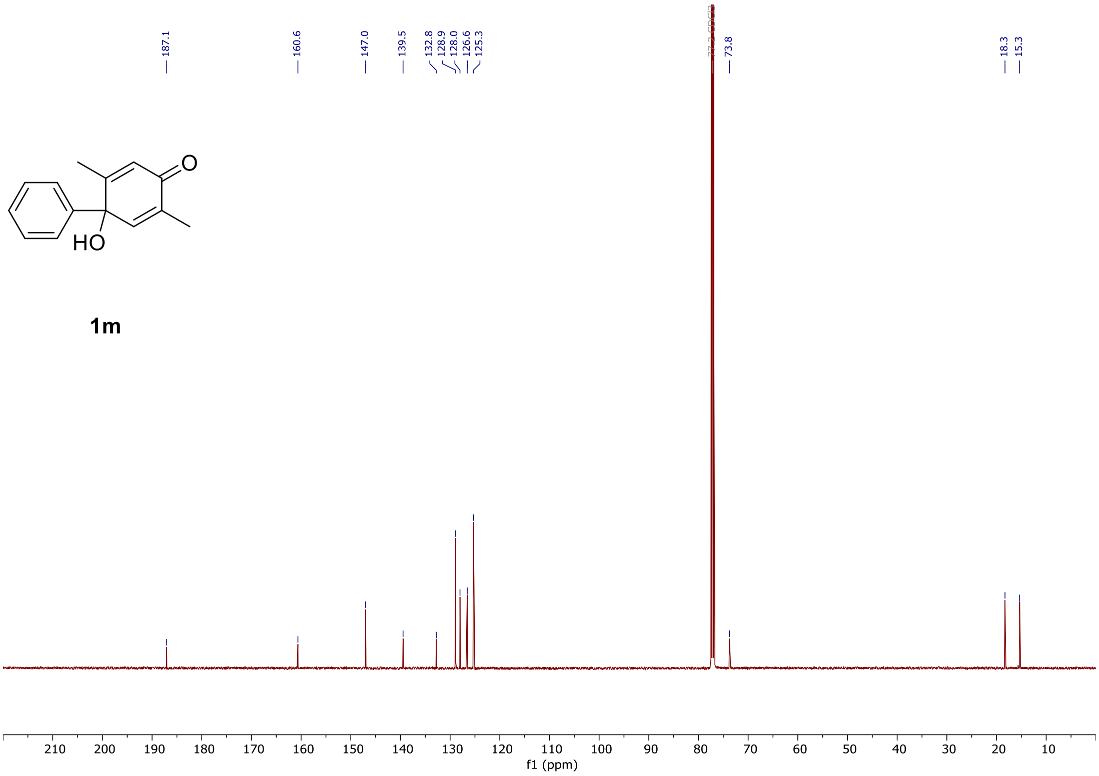

9.2.28. ^{1}H NMR Spectrum of Compound S24 (500 MHz, $CDCl_3$):

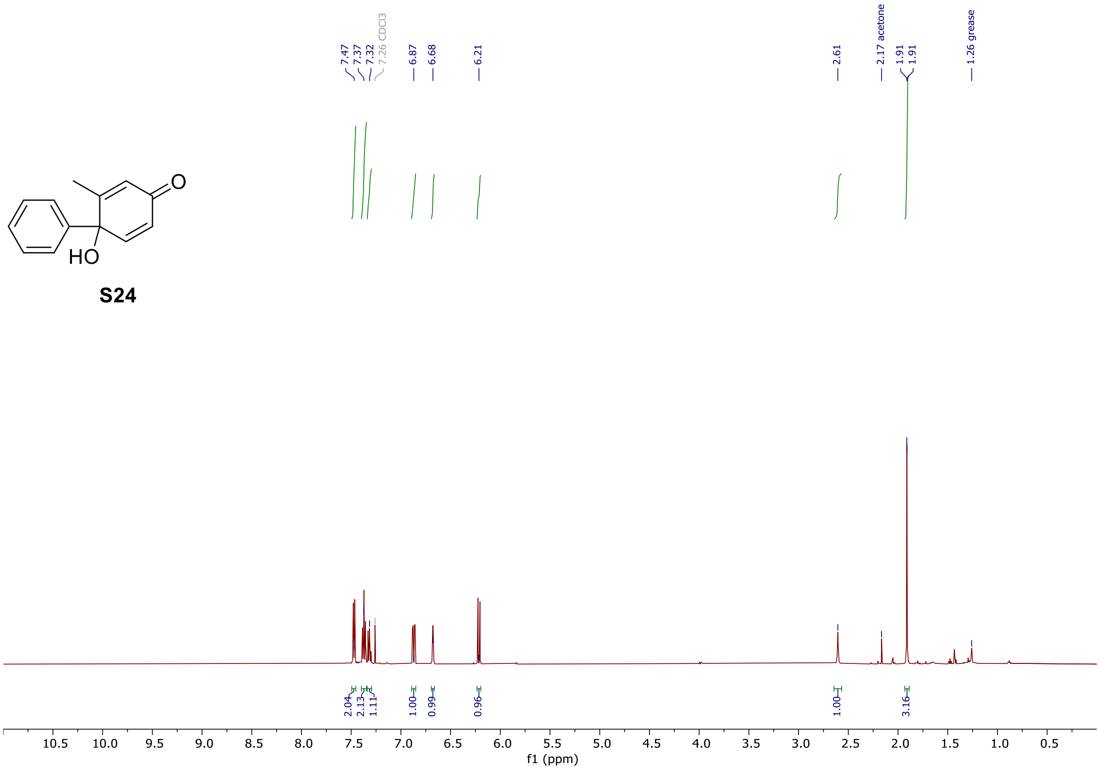

9.2.29. ^{13}C NMR Spectrum of Compound S24 (126 MHz, $CDCl_3$):

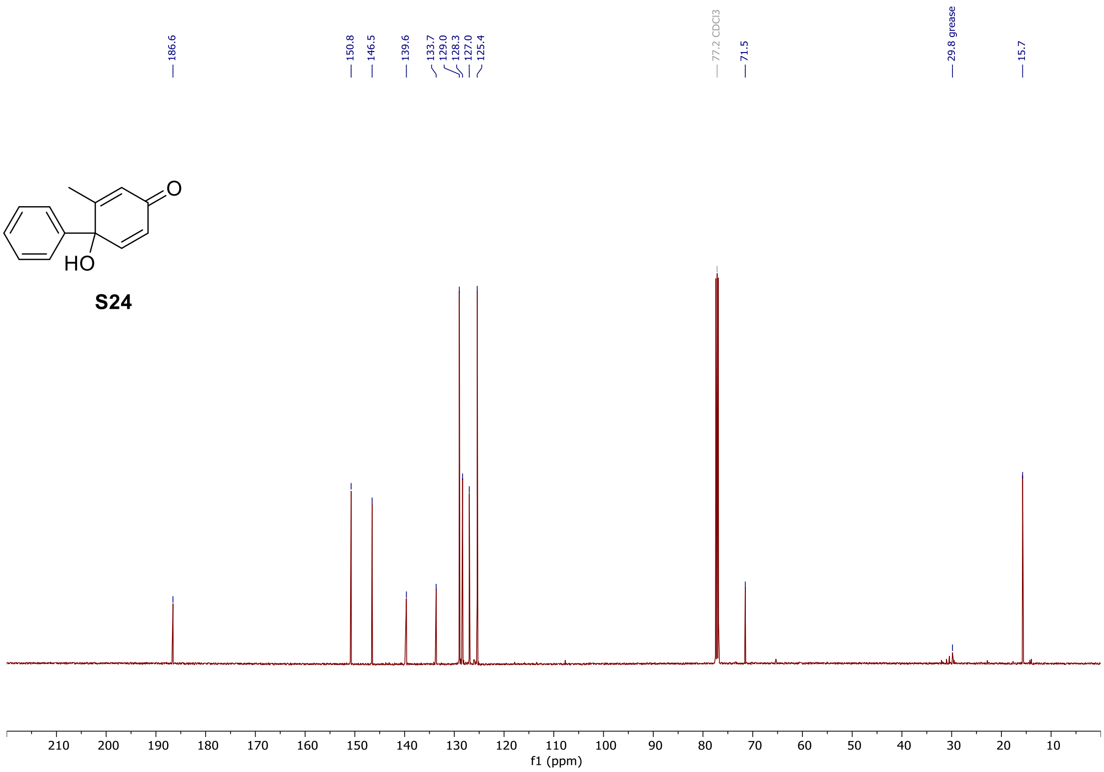

9.2.30. ^{1}H NMR Spectrum of Compound 1n (500 MHz, $CDCl_3$):

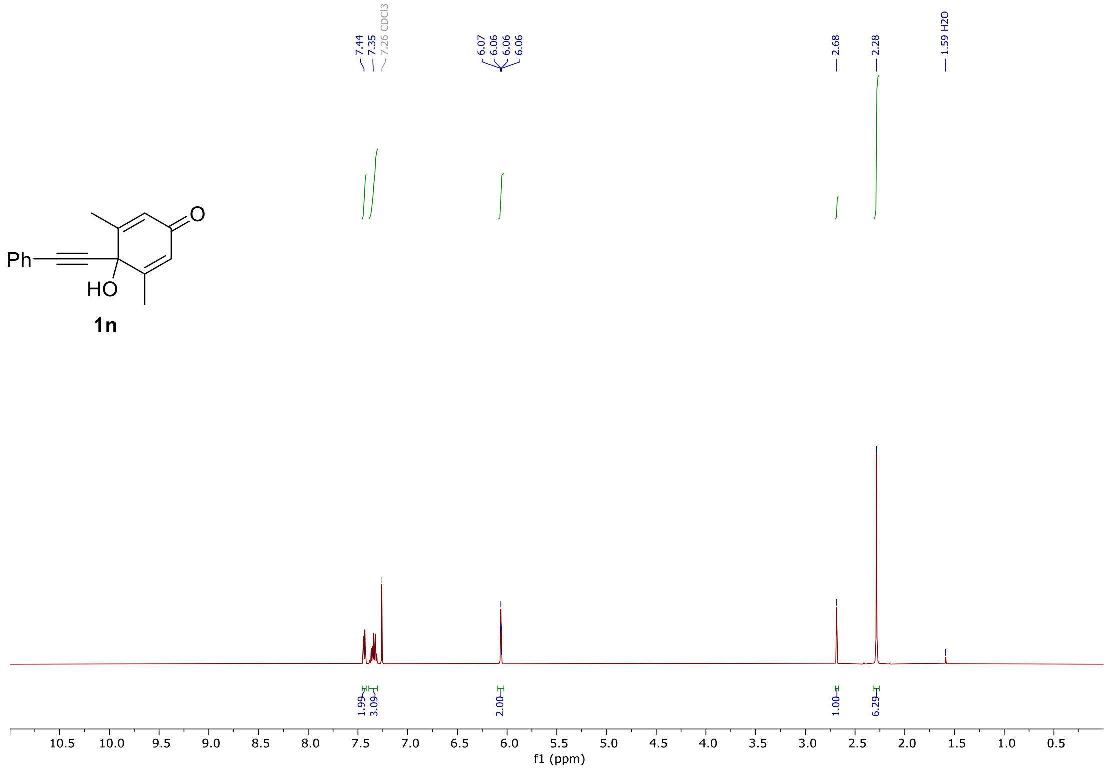

9.2.31. ^{13}C NMR Spectrum of Compound 1n (126 MHz, $CDCl_3$):

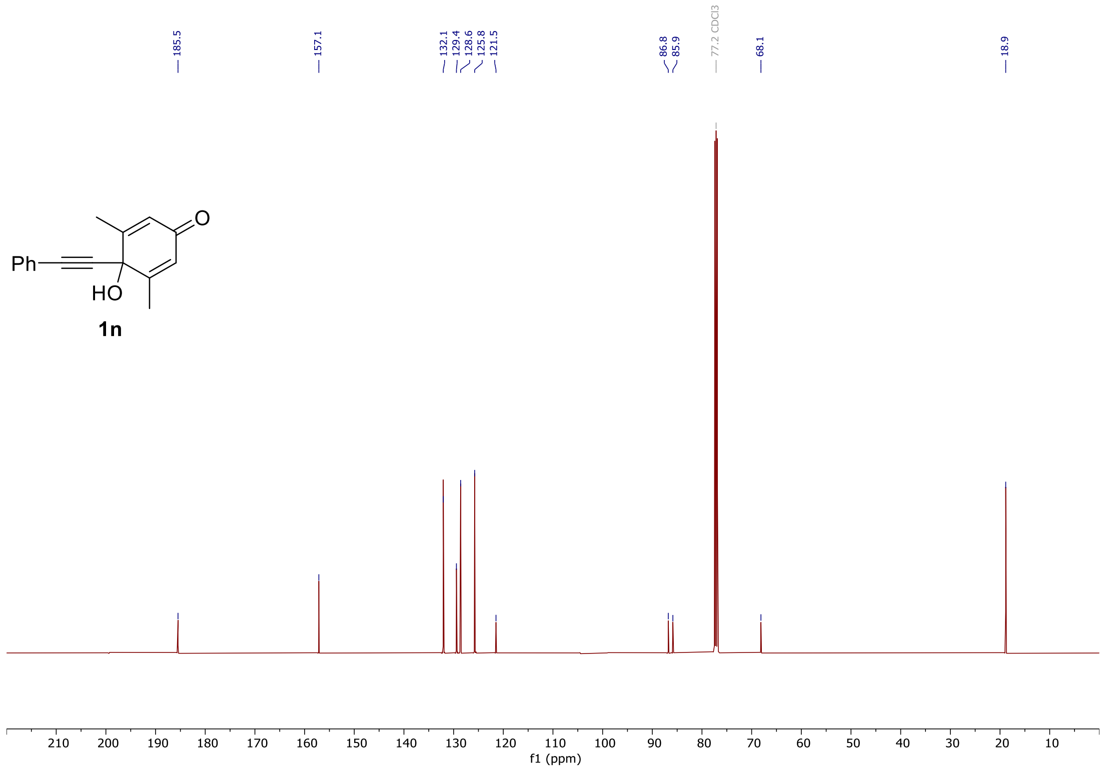

9.2.32. ^{1}H NMR Spectrum of Compound 1o (601 MHz, $CDCl_3$)

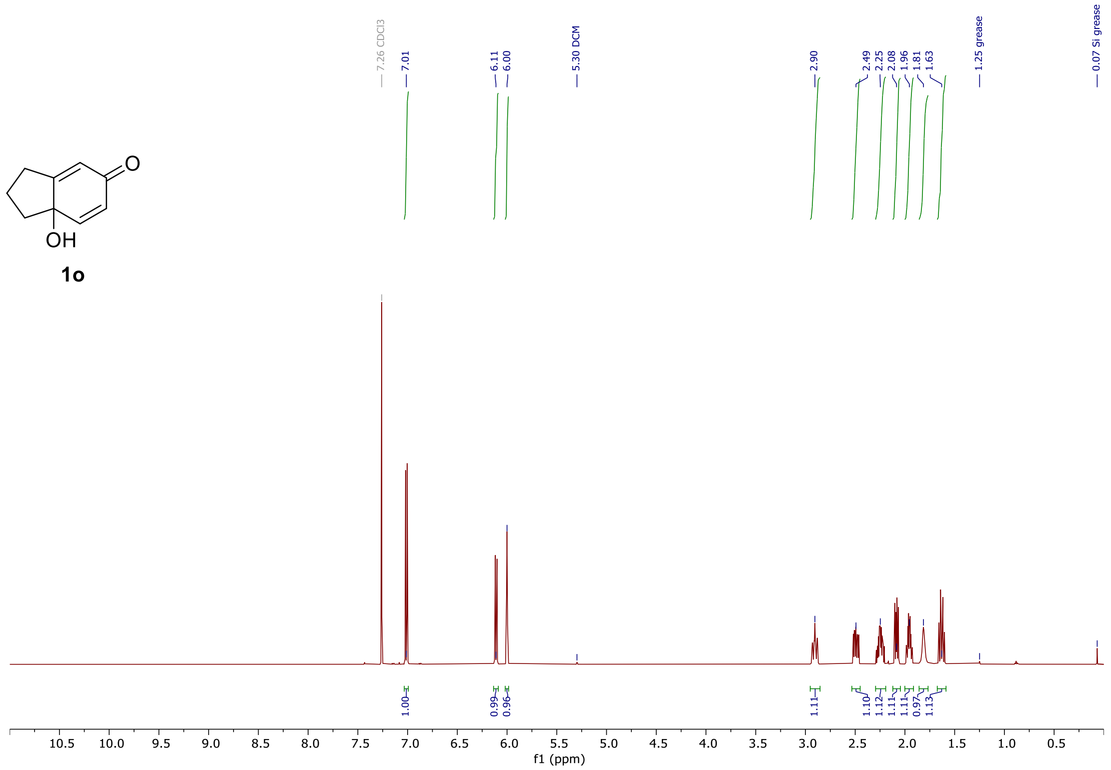

9.2.33. ^{13}C NMR Spectrum of Compound 1o (151 MHz, $CDCl_3$)

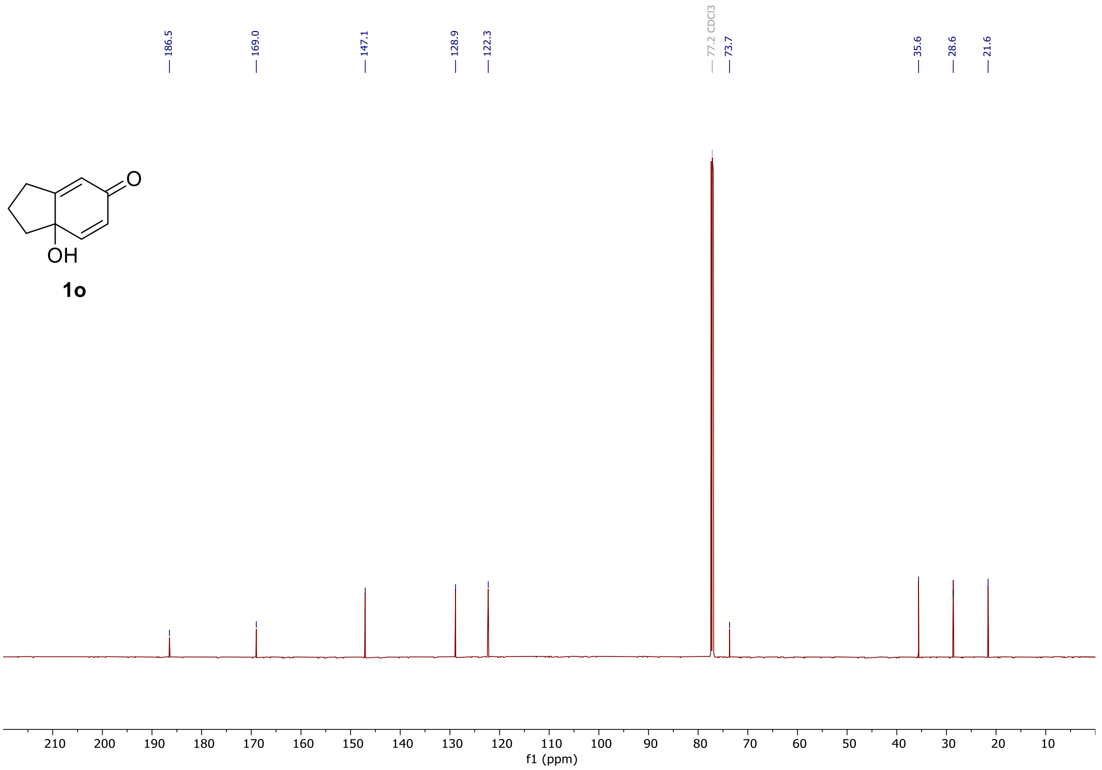

9.2.34. ^{1}H NMR Spectrum of Compound S19 (500 MHz, $CDCl_3$)

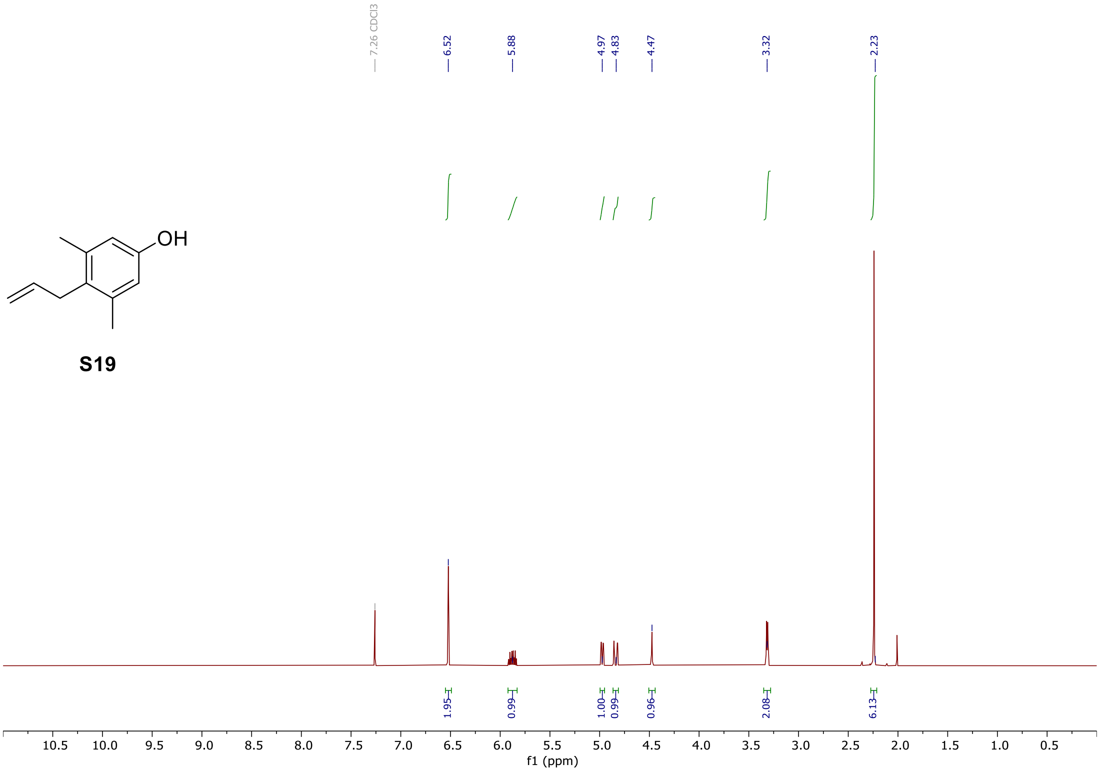

S19

9.2.35. ^{13}C NMR Spectrum of Compound S19 (126 MHz, $CDCl_3$)

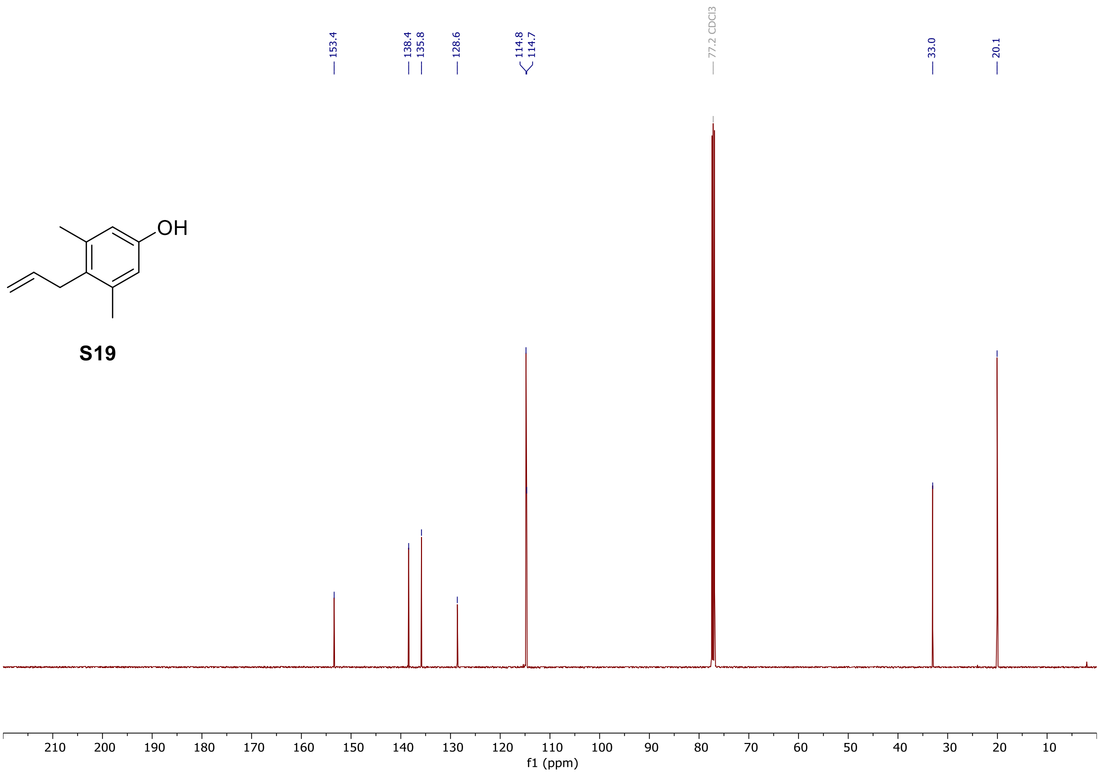

S19

9.2.36. ^{1}H NMR Spectrum of Compound 1p (601 MHz, $CDCl_3$)

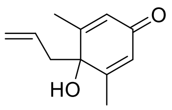

1p

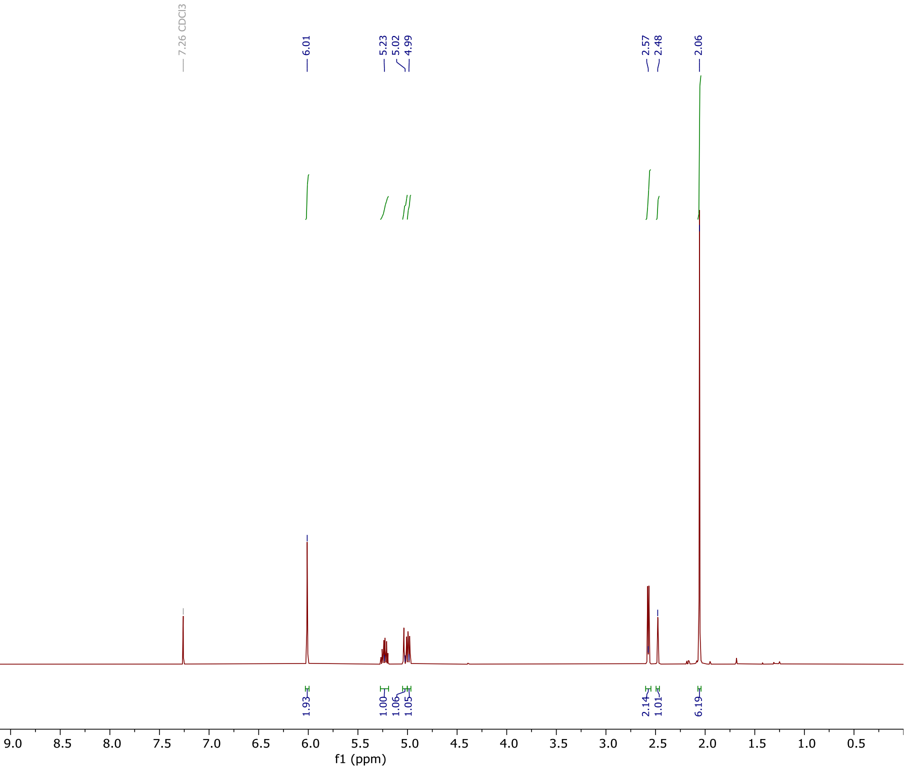

9.2.37. ^{13}C NMR Spectrum of Compound 1p (151 MHz, $CDCl_3$)

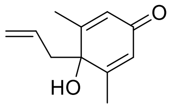

1p

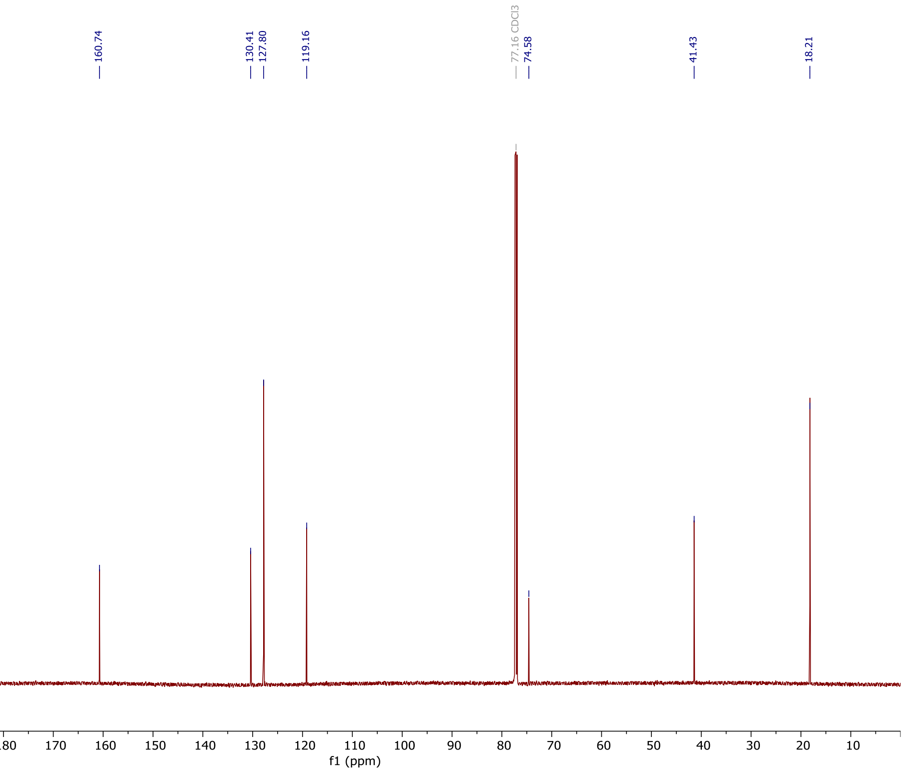

9.3. Enediones

9.3.1. ^{1}H NMR Spectrum of Compound 2a (500 MHz, $CDCl_3$):

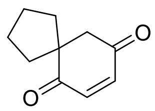

2a

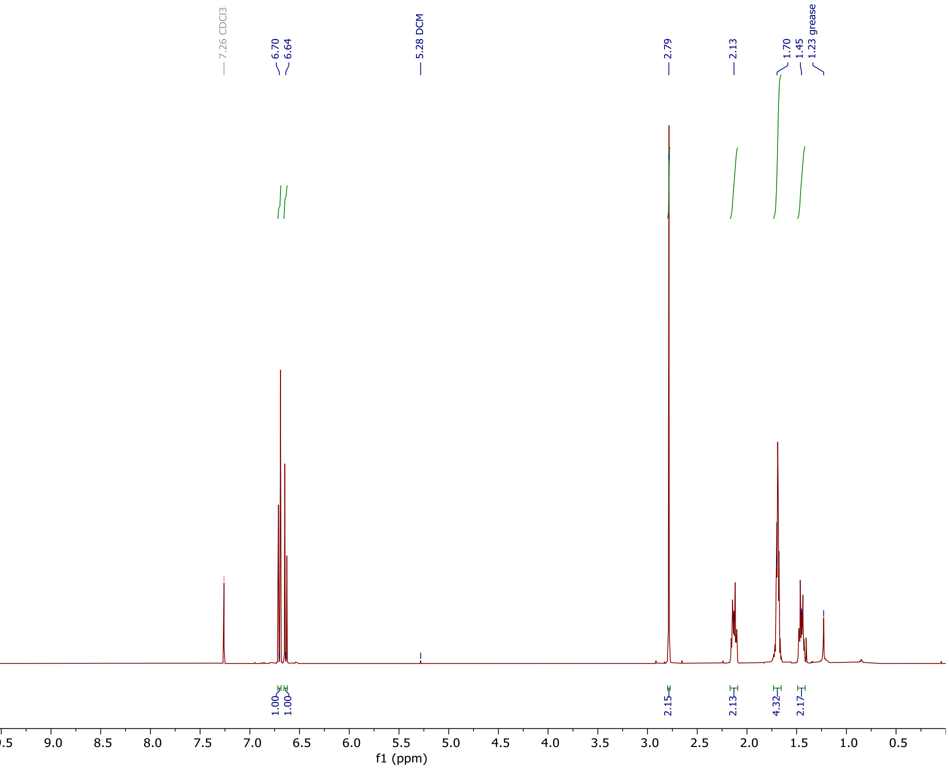

9.3.2. ^{13}C NMR Spectrum of Compound 2a (126 MHz, $CDCl_3$):

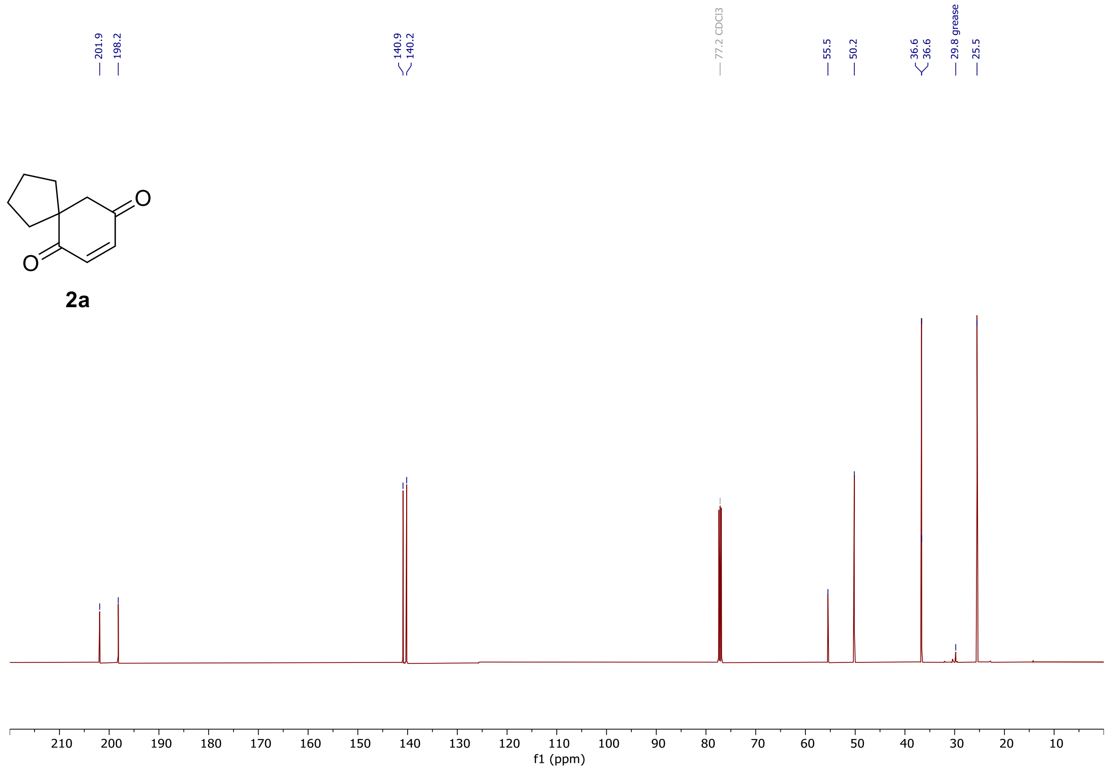

9.3.3. ^{1}H NMR Spectrum of Keto-Isophorone 2b (500 MHz, $CDCl_3$):

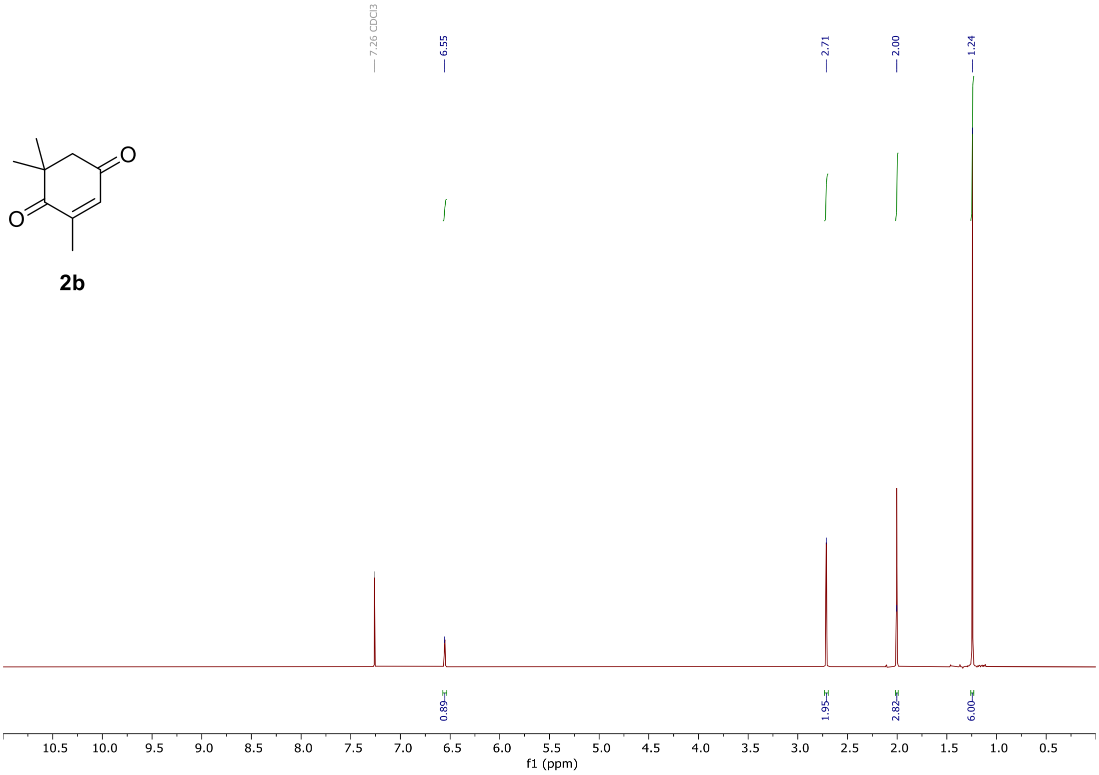

9.3.4. ^{13}C NMR Spectrum of Keto-Isophorone 2b (126 MHz, $CDCl_3$):

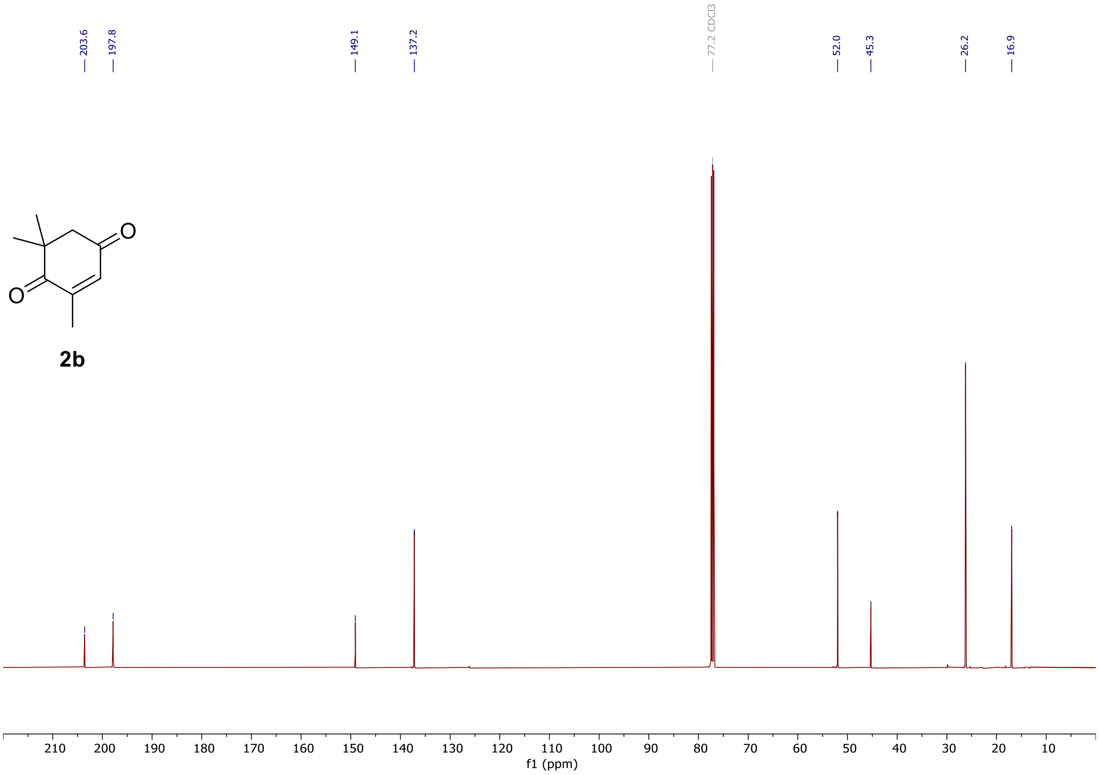

9.3.5. ^{1}H NMR Spectrum of Compound 2c (500 MHz, $CDCl_3$):

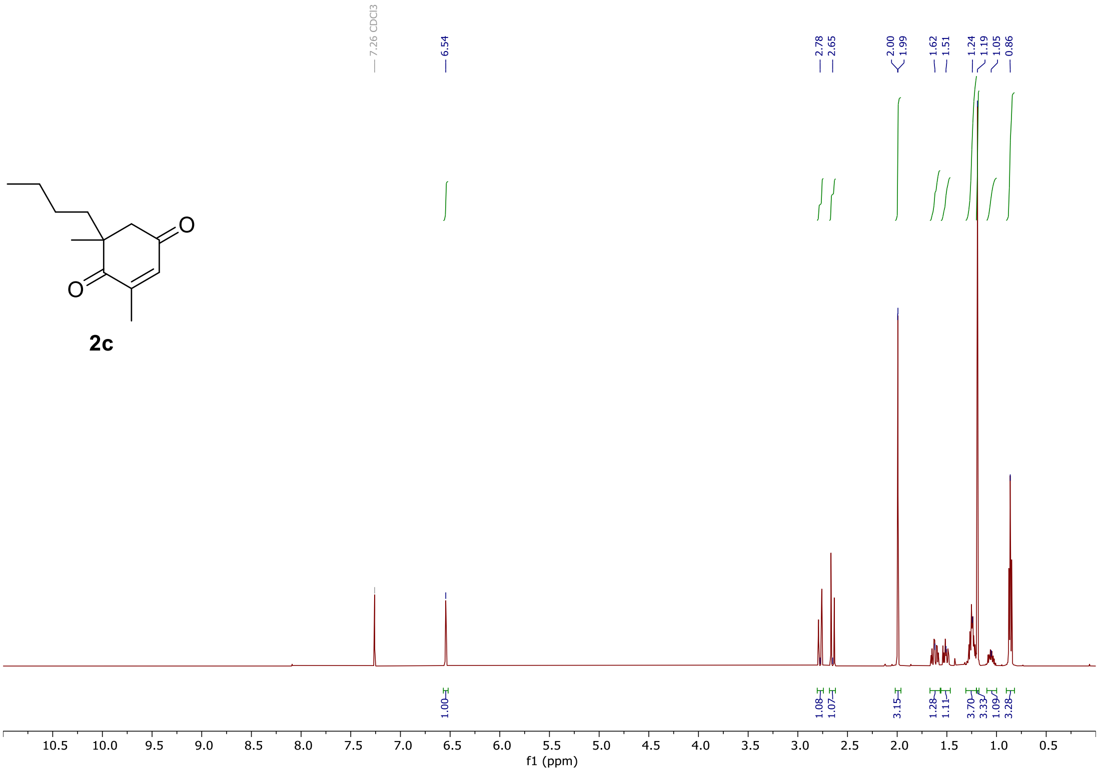

9.3.6. ^{13}C NMR Spectrum of Compound 2c (126 MHz, $CDCl_3$):

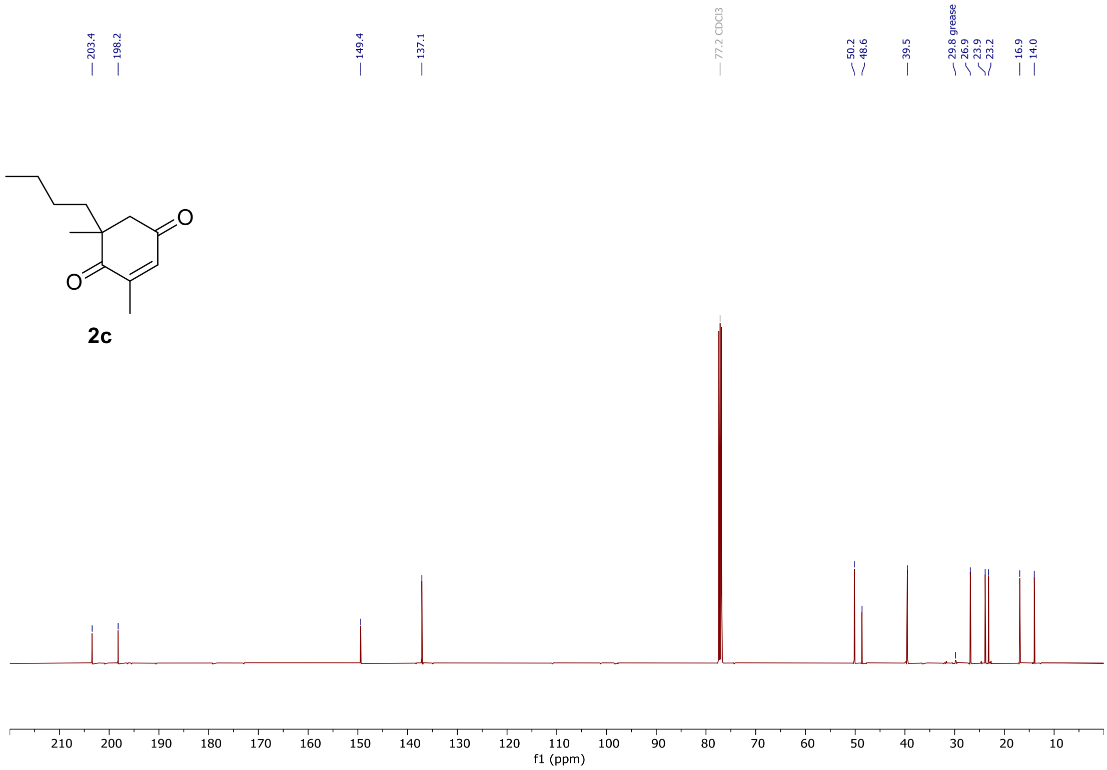

9.3.7. ^{1}H NMR Spectrum of Compound 2d (500 MHz, $CDCl_3$):

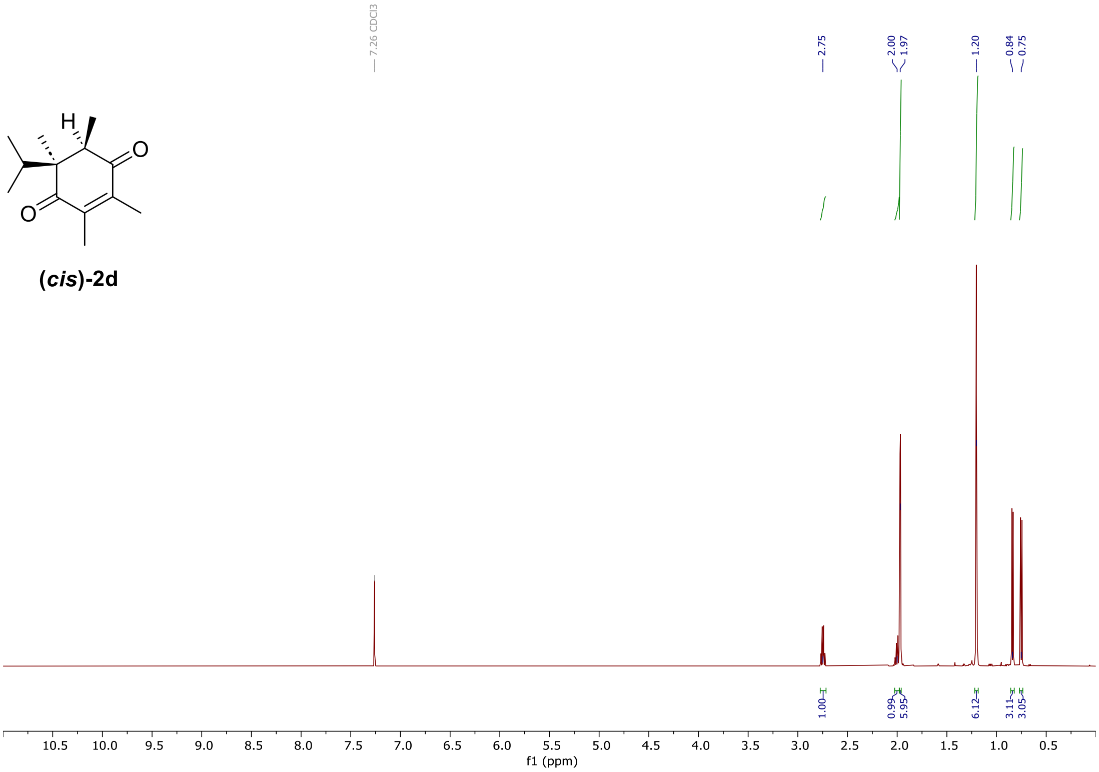

9.3.8. ^{13}C NMR Spectrum of Compound 2d (126 MHz, CDCl$_3$):

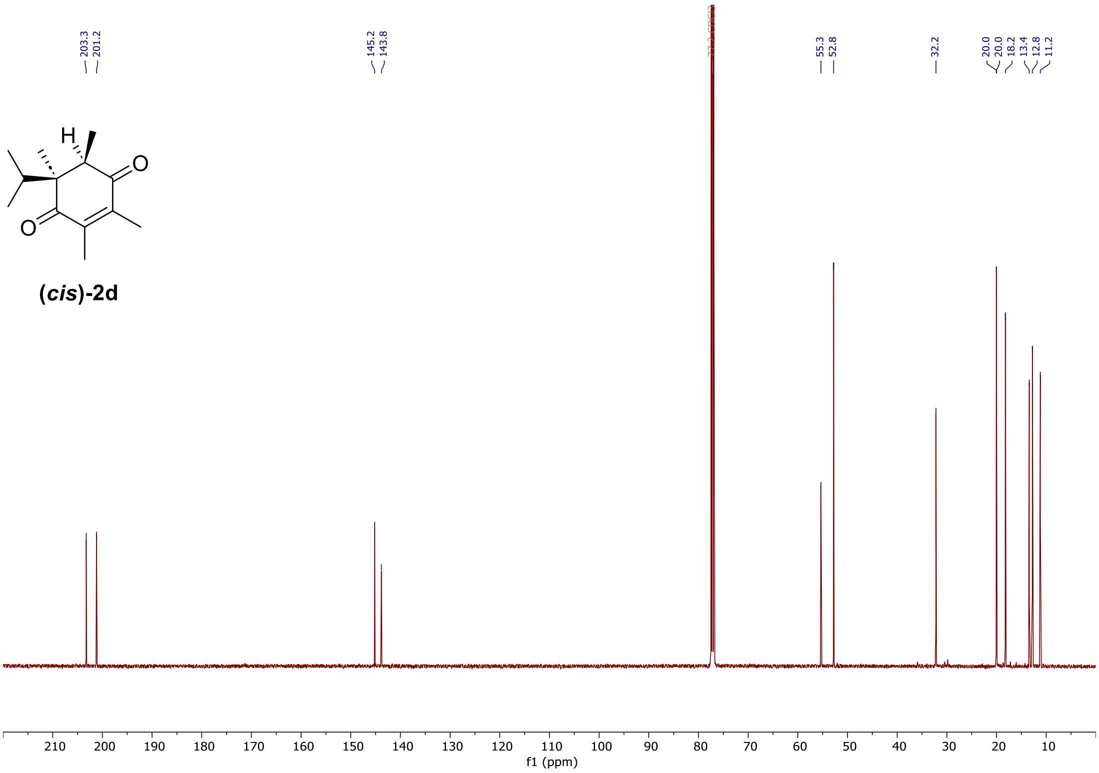

9.3.9. NOESY Spectrum of Compound 2d (500 MHz, MeOD, diagonal suppressed):

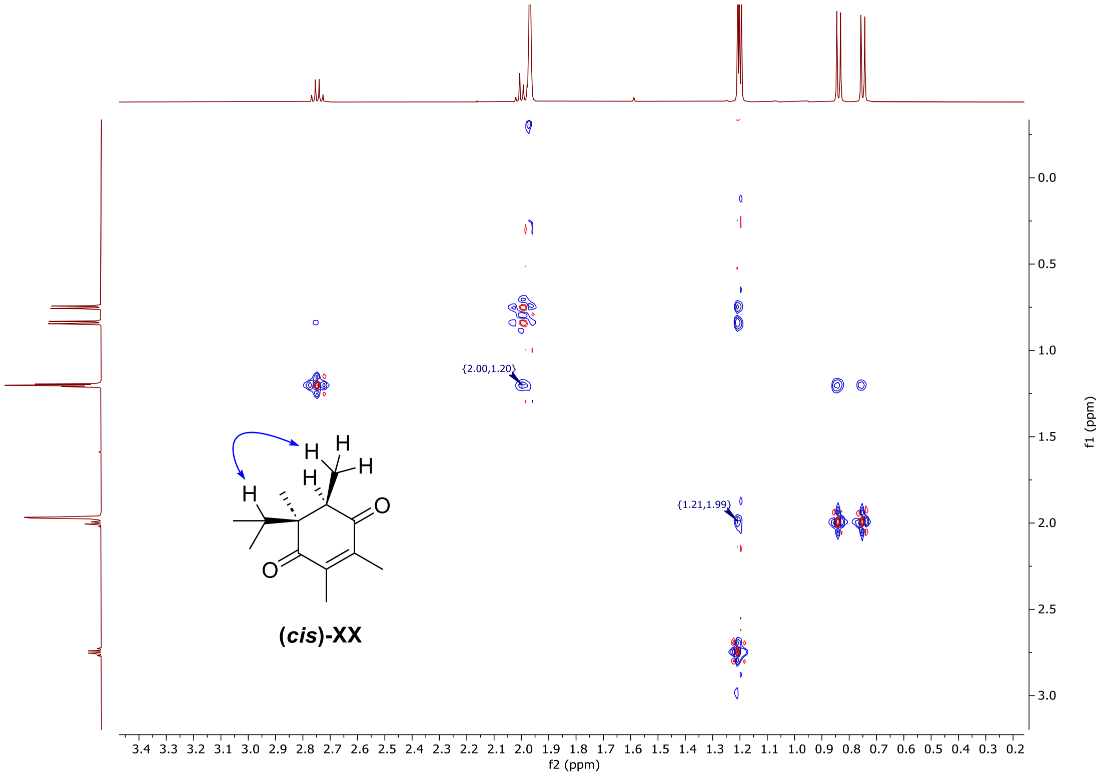

9.3.10. ^{1}H NMR Spectrum of Compound 2e (601 MHz, $CDCl_3$):

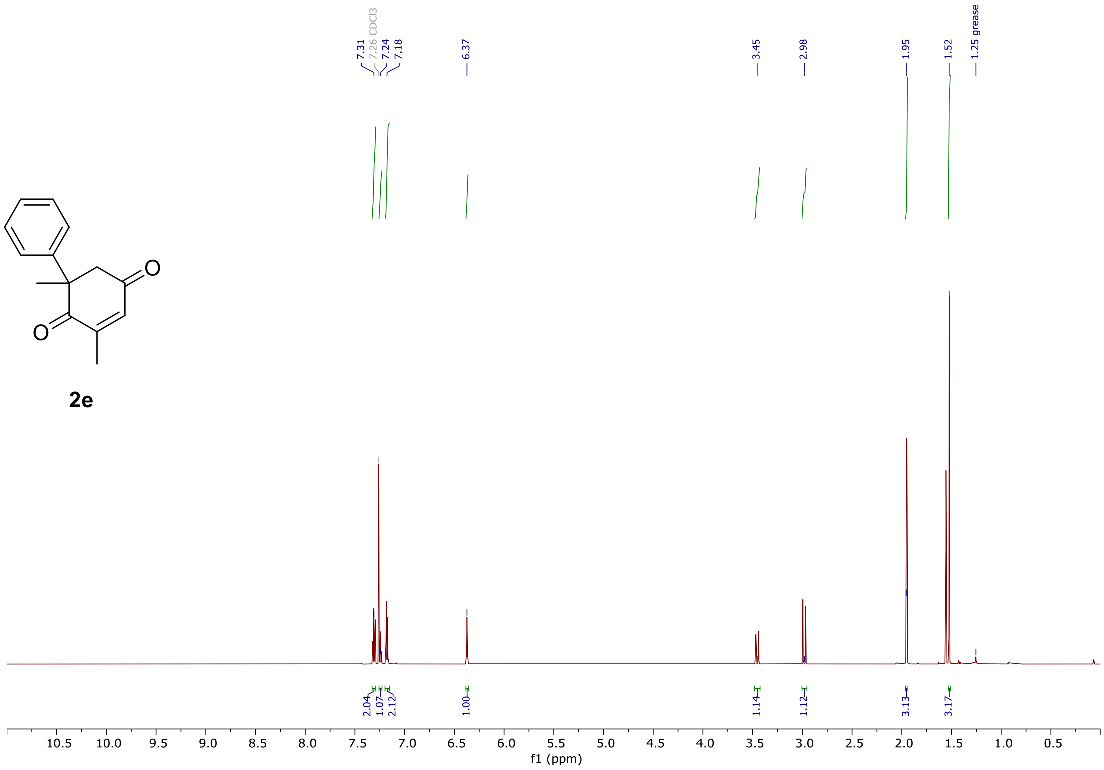

2e

9.3.11. ^{13}C NMR Spectrum of Compound 2e (126 MHz, $CDCl_3$):

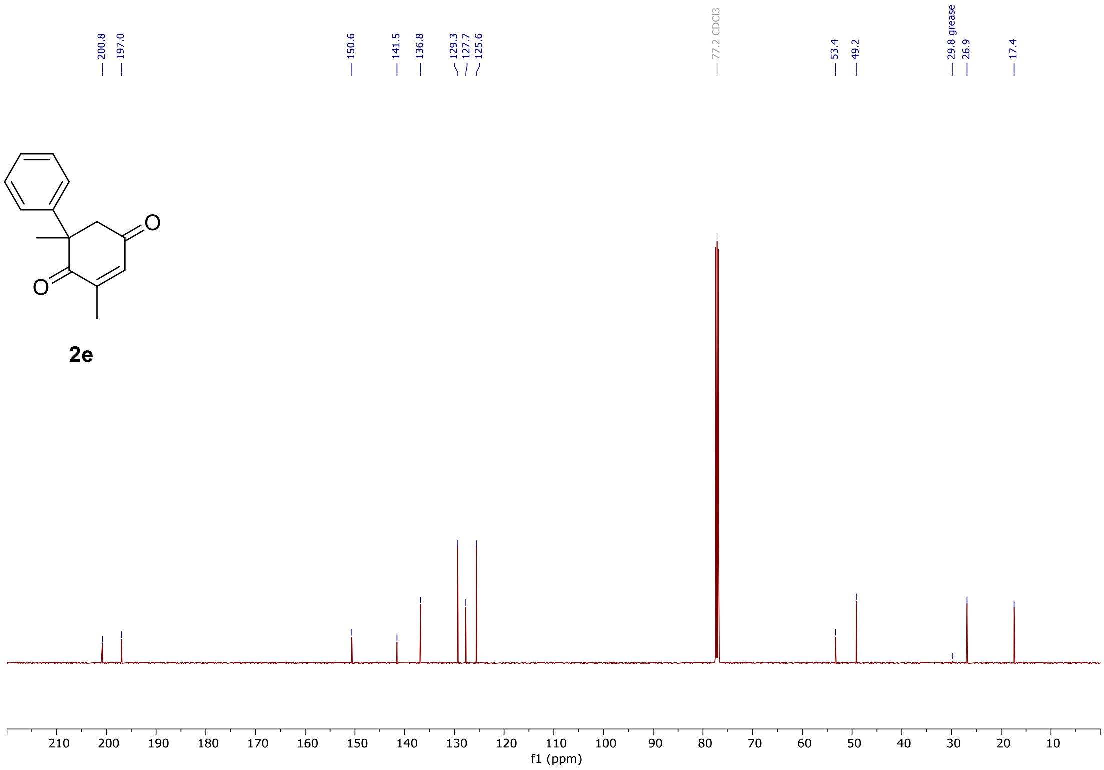

2e

9.3.12. ^{1}H NMR Spectrum of Compound 2f (500 MHz, $CDCl_3$):

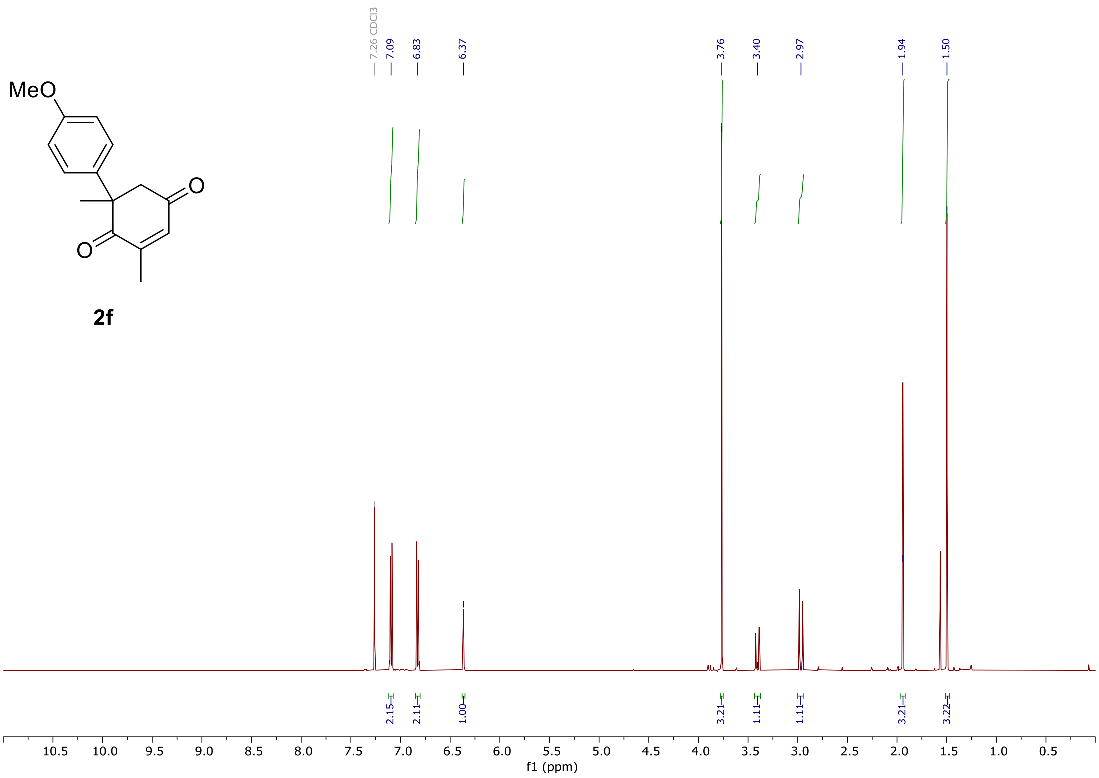

9.3.13. ^{13}C NMR Spectrum of Compound 2f (126 MHz, $CDCl_3$):

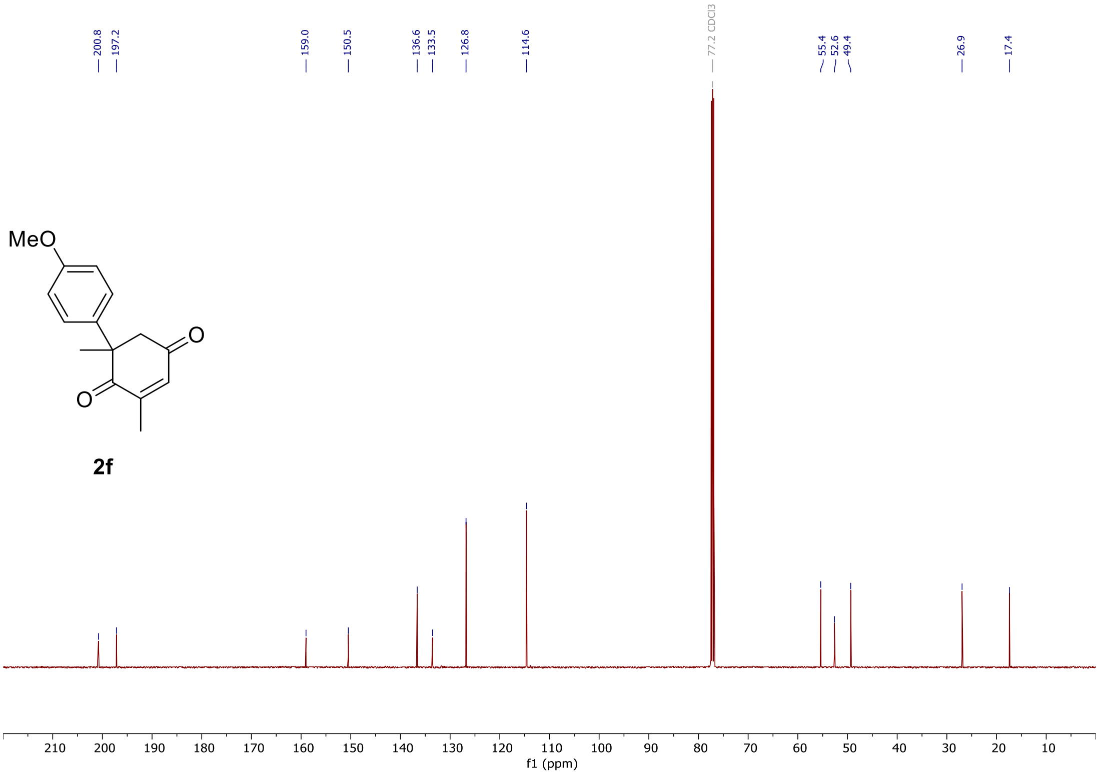

9.3.14. ^{1}H NMR Spectrum of Compound 2g (500 MHz, $CDCl_3$):

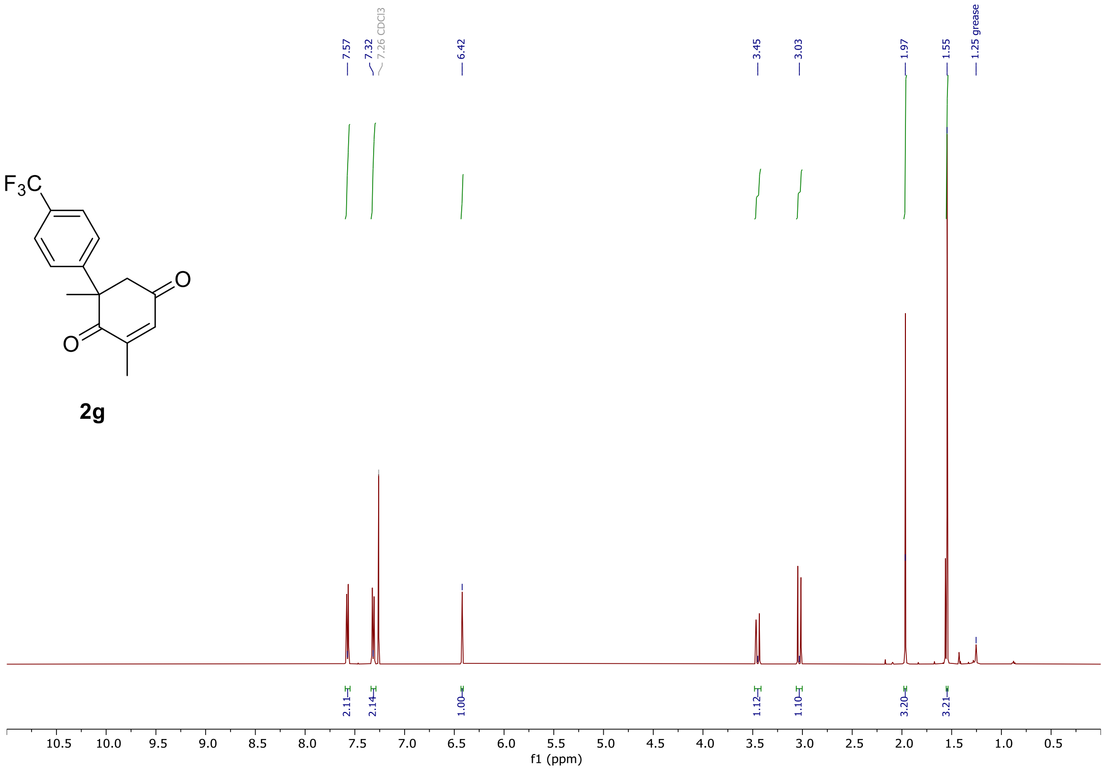

2g

9.3.15. ^{13}C NMR Spectrum of Compound 2g (126 MHz, $CDCl_3$):

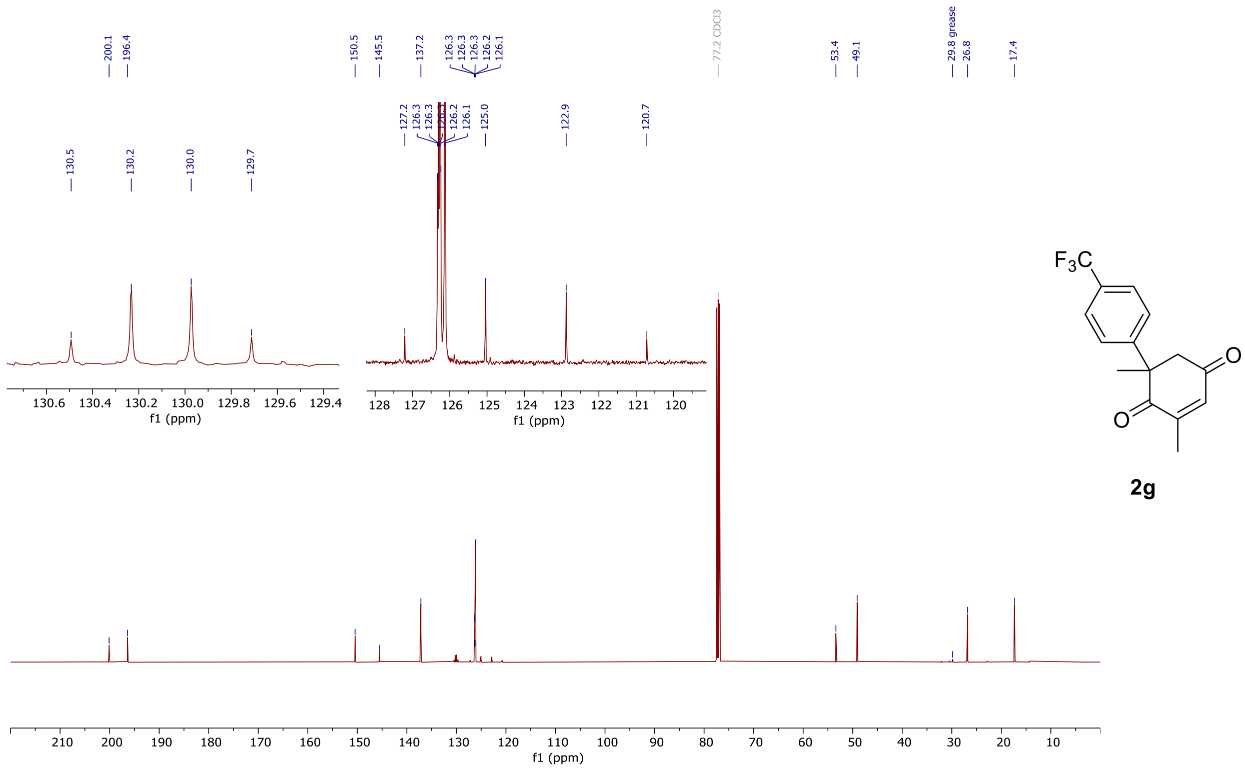

2g

9.3.16. ^{19}F NMR Spectrum of Compound 2g (471 MHz, $CDCl_3$):

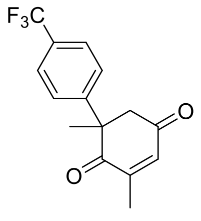

2g

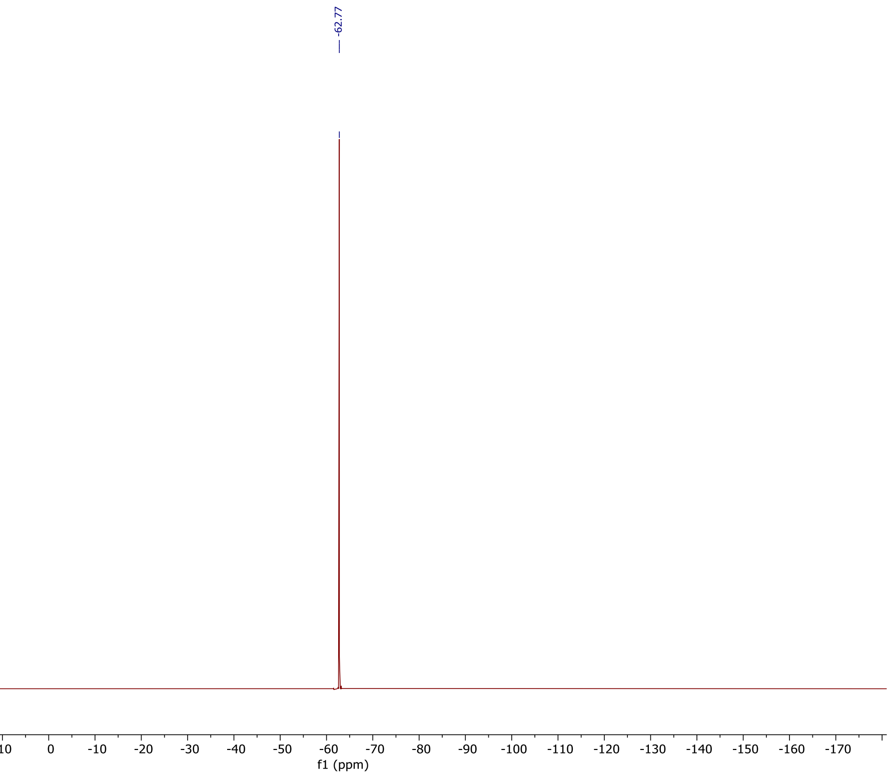

9.3.17. ^{1}H NMR Spectrum of Compound 2h (500 MHz, $CDCl_3$):

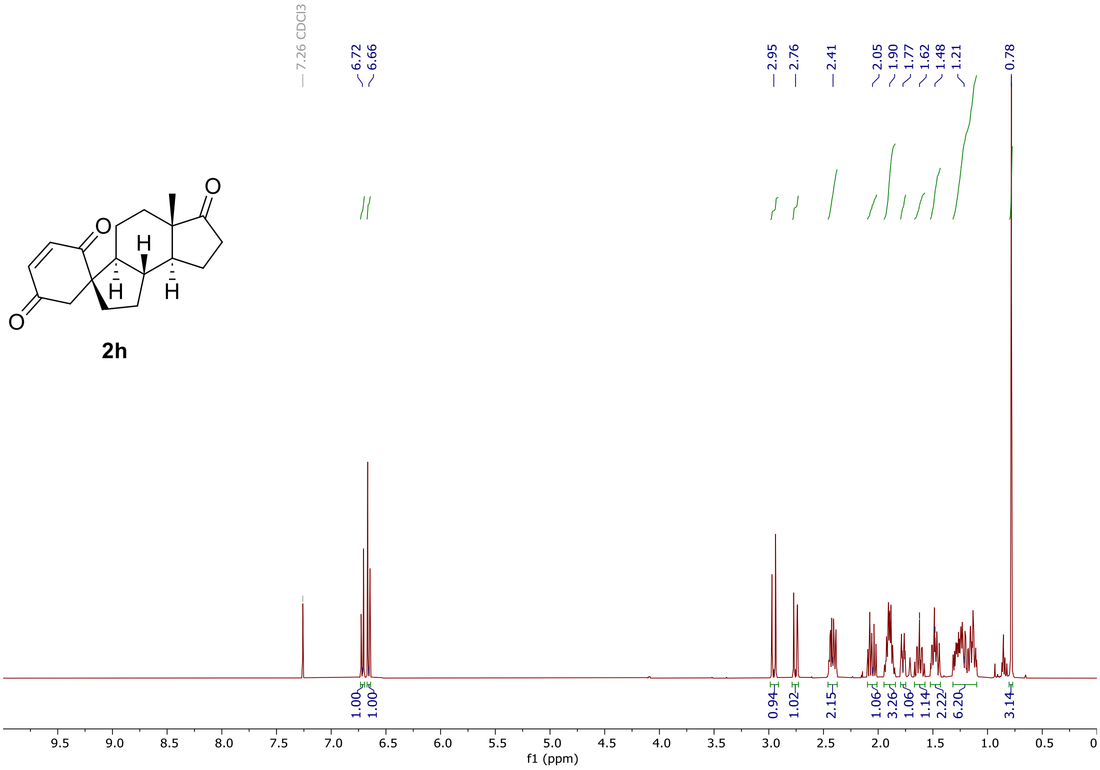

9.3.18. ^{13}C NMR Spectrum of Compound 2h (126 MHz, $CDCl_3$):

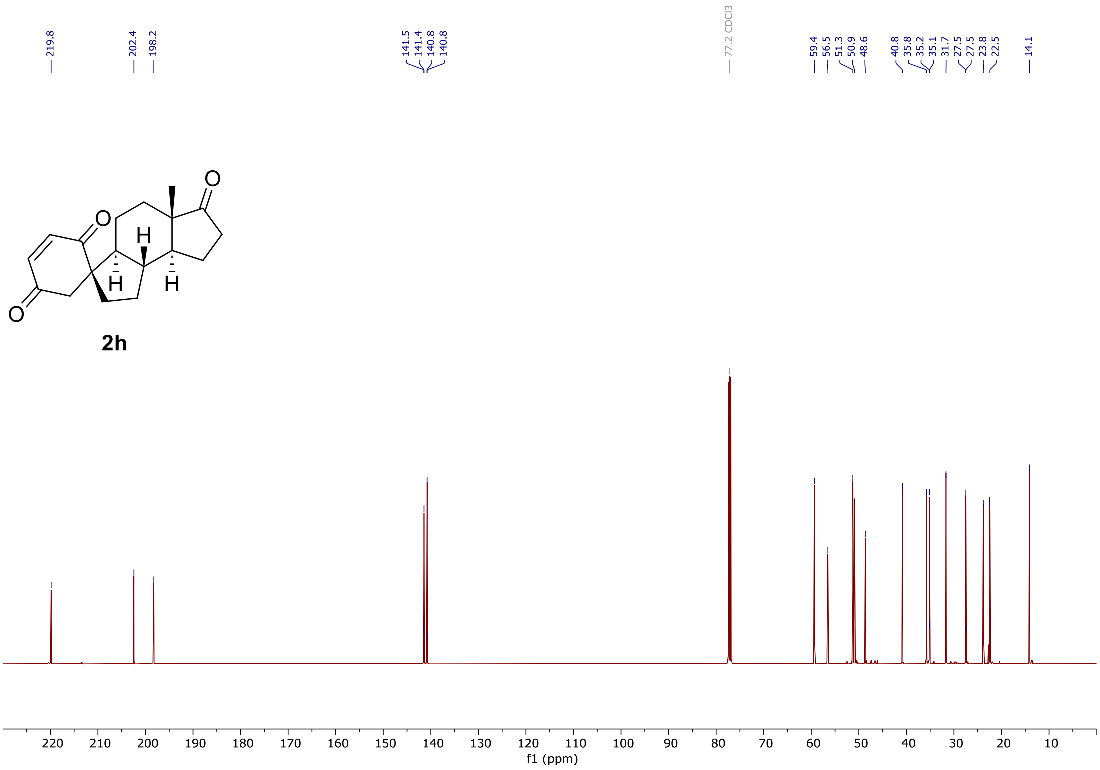

9.3.19. ^{1}H NMR Spectrum of Compound 2i (500 MHz, $CDCl_3$):

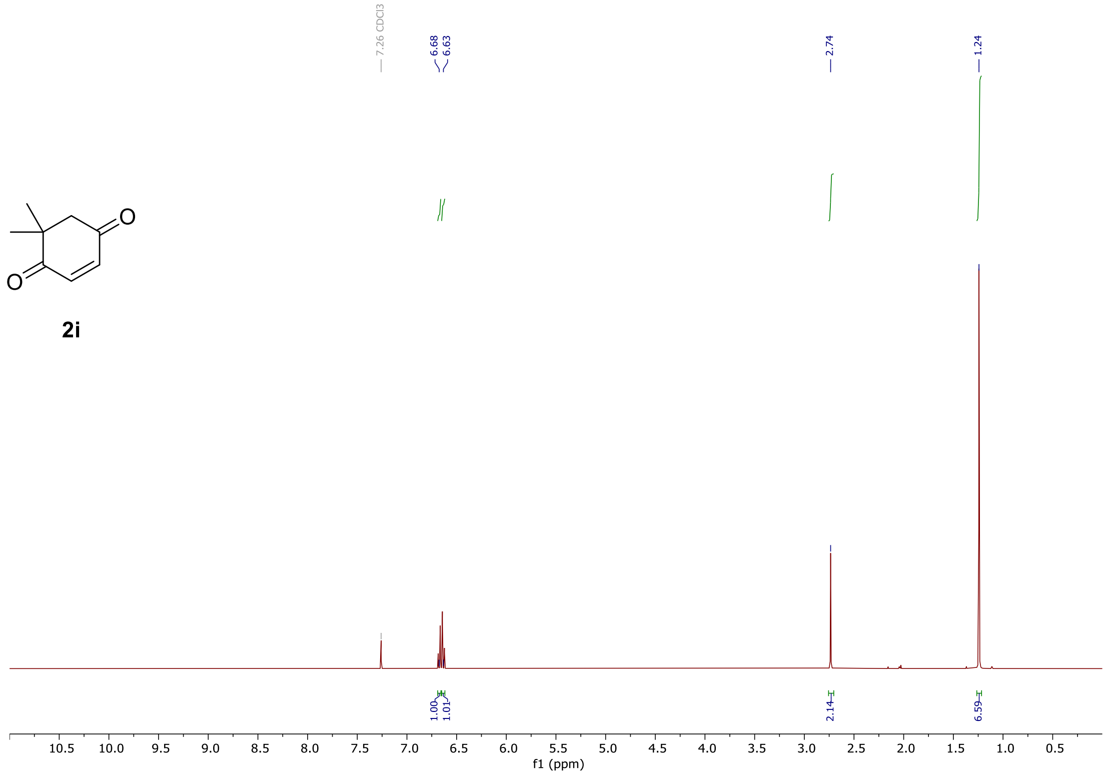

9.3.20. ^{13}C NMR Spectrum of Compound 2i (126 MHz, $CDCl_3$):

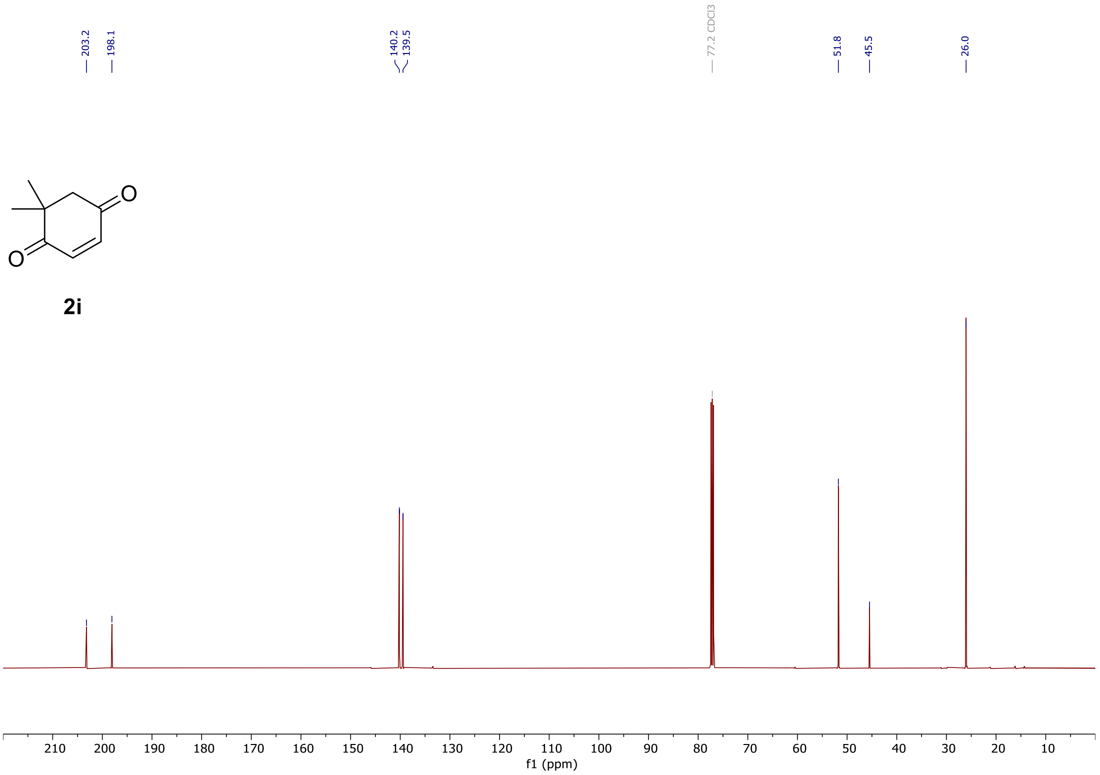

9.3.21. ^{1}H NMR Spectrum of Compound 2j (500 MHz, $CDCl_3$):

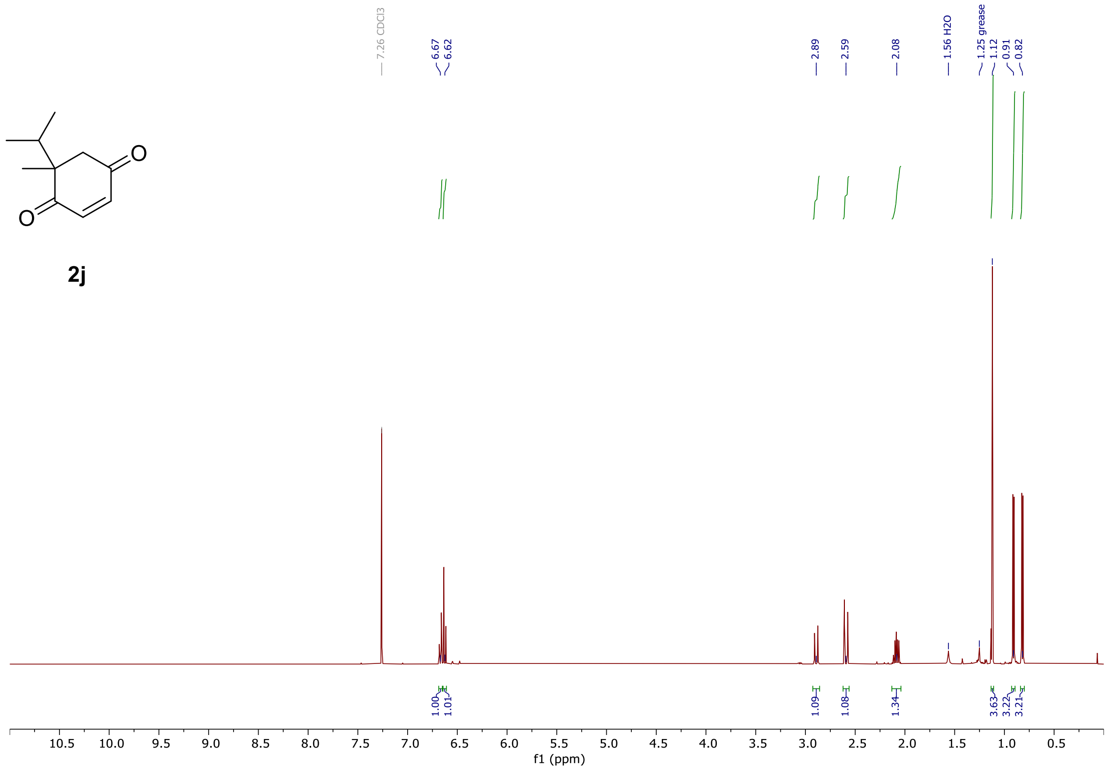

9.3.22. ^{13}C NMR Spectrum of Compound 2j (126 MHz, $CDCl_3$):

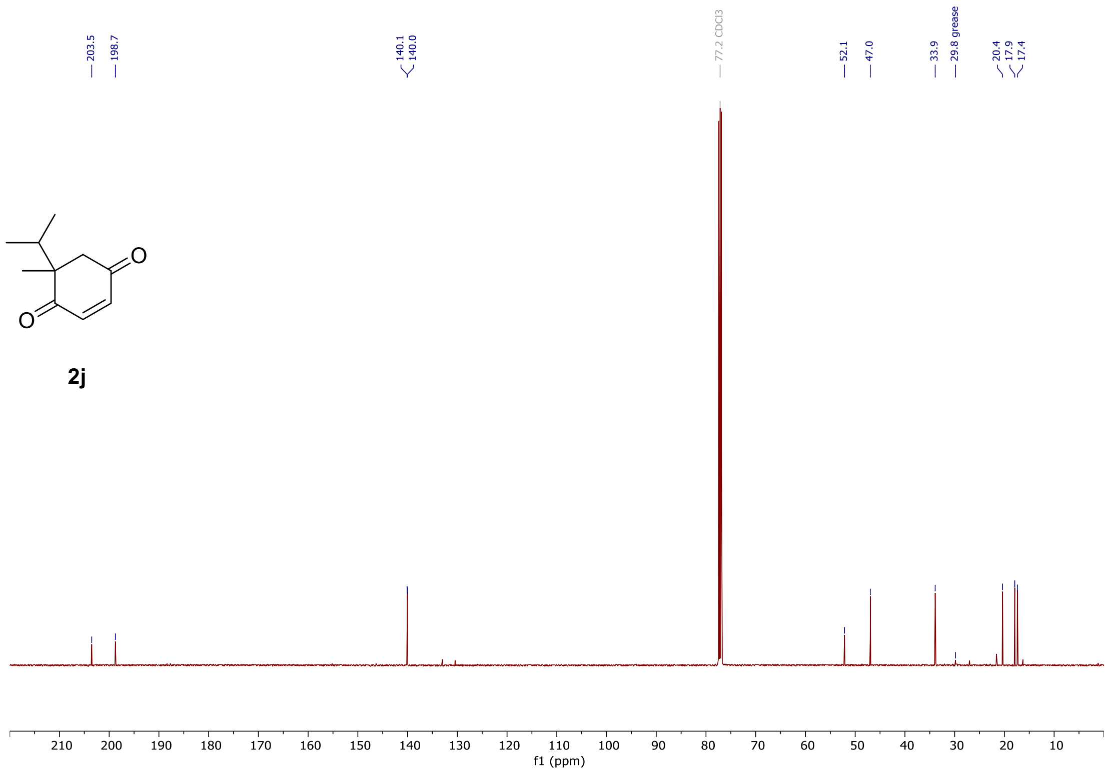

9.3.23. ^{1}H NMR Spectrum of Compound 4j (500 MHz, $CDCl_3$):

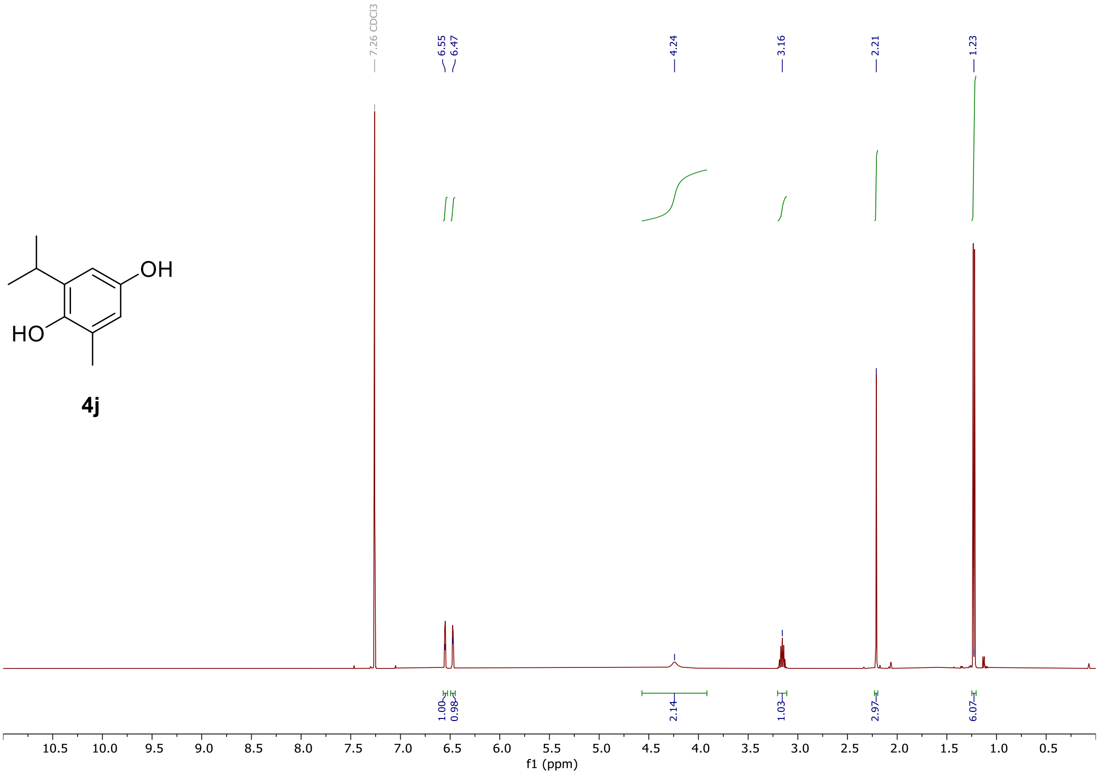

9.3.24. ^{13}C NMR Spectrum of Compound 4j (126 MHz, $CDCl_3$):

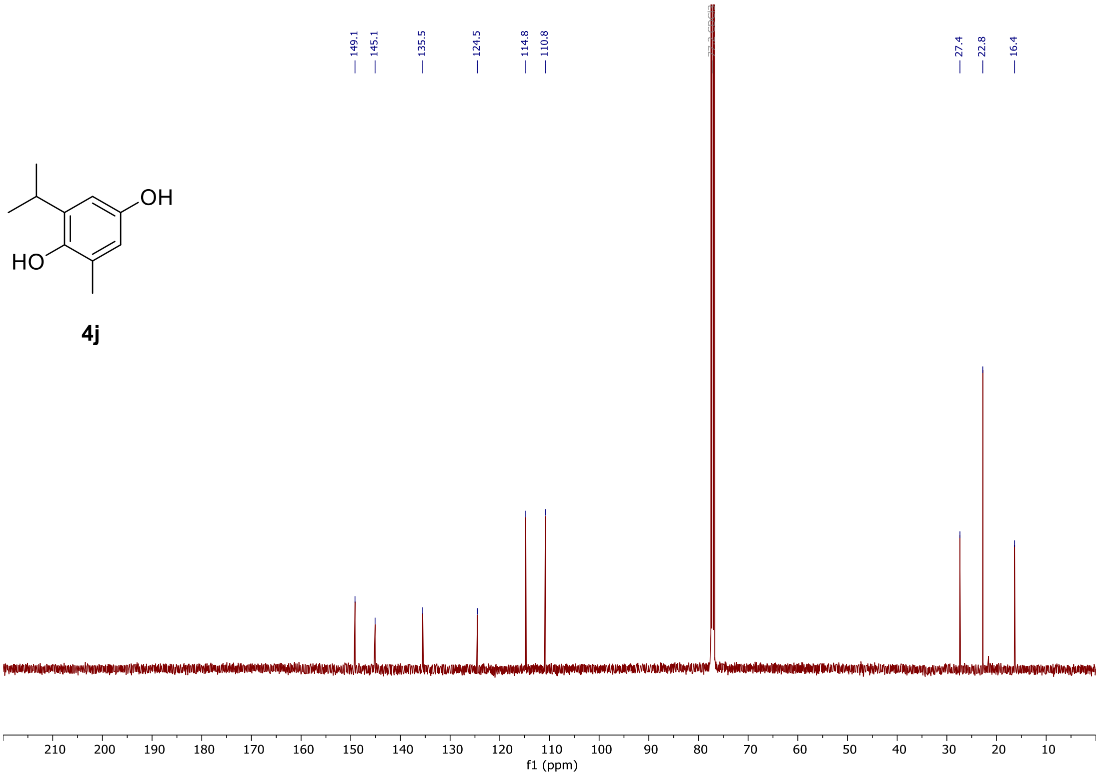

9.3.25. ^{1}H NMR Spectrum of Compound 2k (500 MHz, $CDCl_3$):

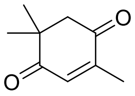

2k

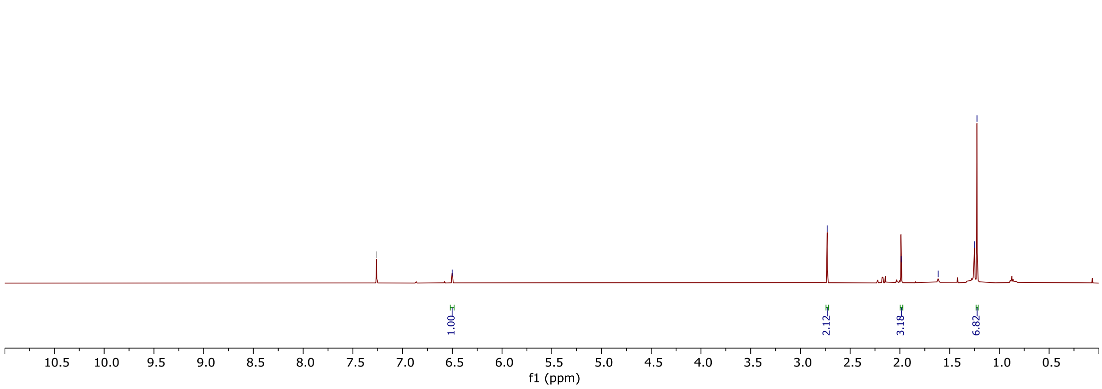

9.3.26. ^{13}C NMR Spectrum of Compound 2k (126 MHz, $CDCl_3$):

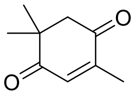

2k

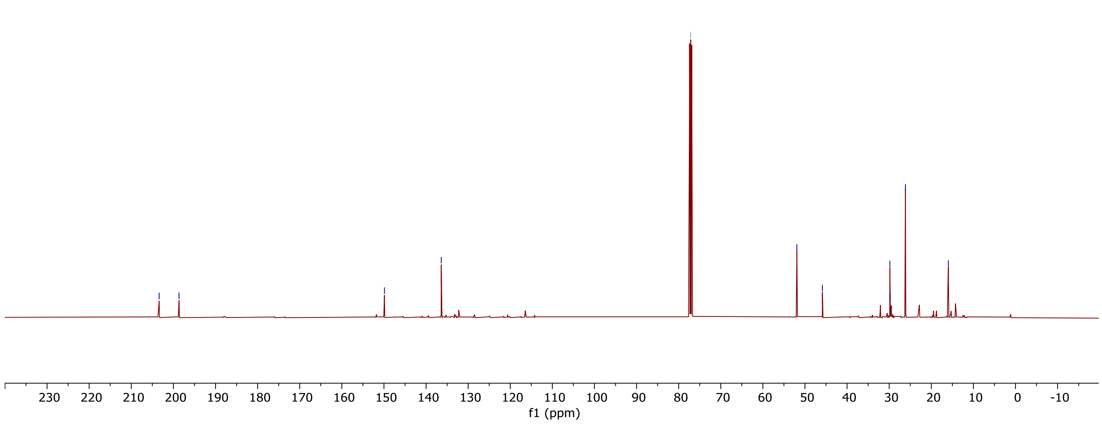

9.3.27. ^{1}H NMR Spectrum of Compound 2l (500 MHz, $CDCl_3$):

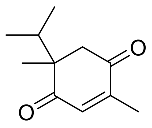

2l

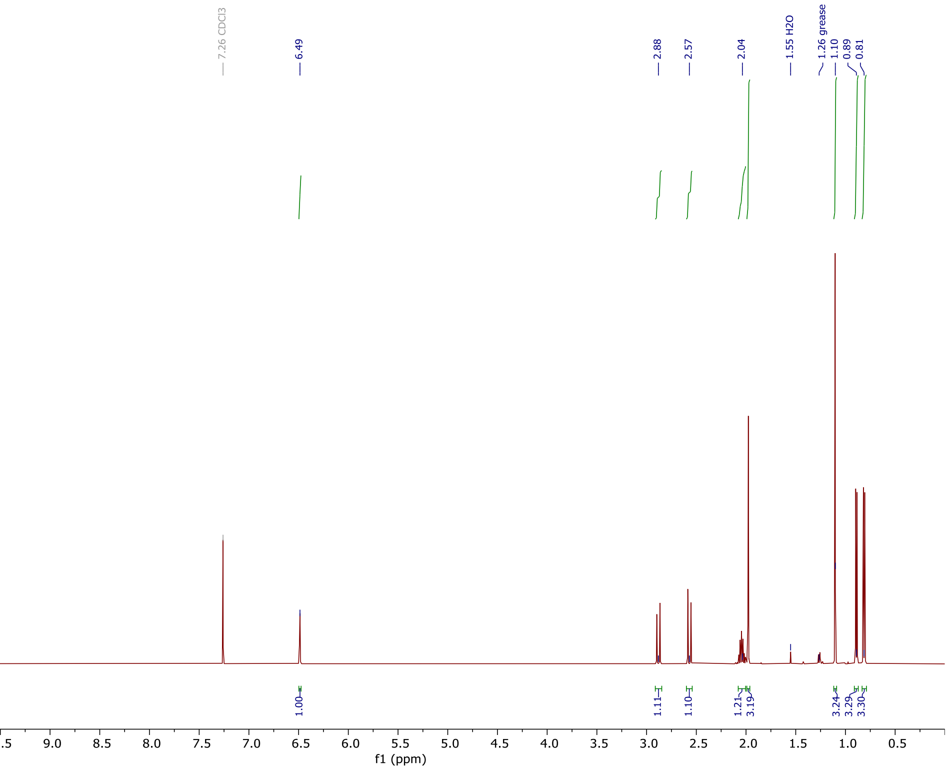

9.3.28. ^{13}C NMR Spectrum of Compound 2l (126 MHz, $CDCl_3$):

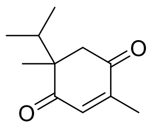

2l

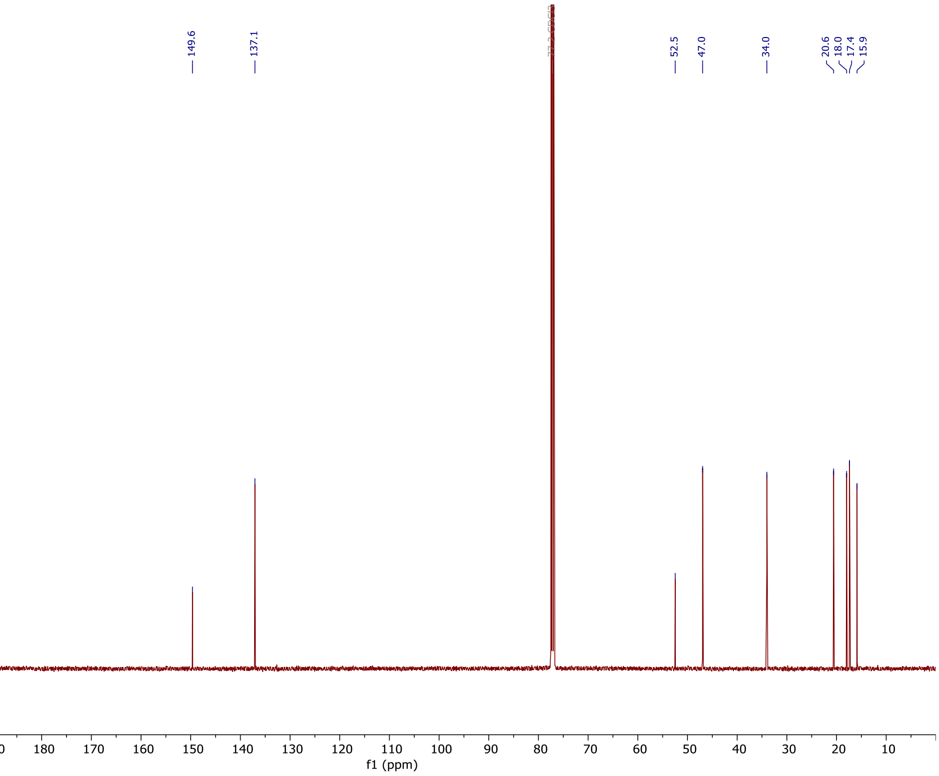

9.3.29. ^{1}H NMR Spectrum of Compound 4l (500 MHz, $CDCl_3$):

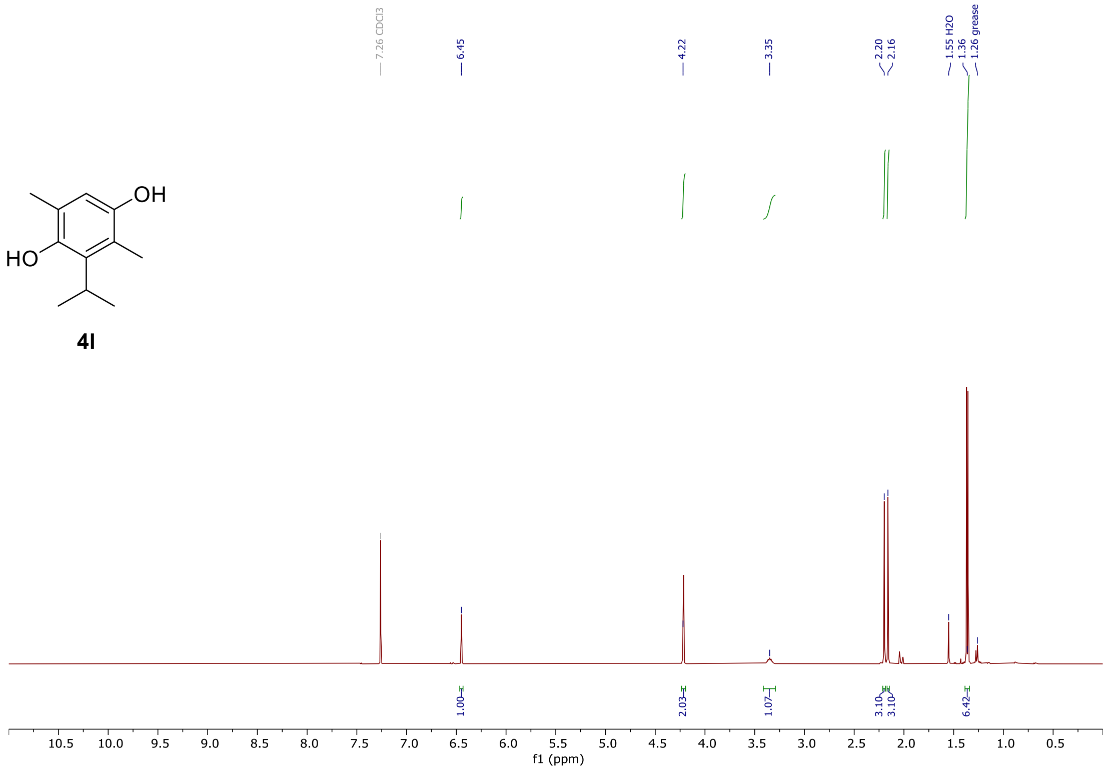

9.3.30. ^{13}C NMR Spectrum of Compound 4l (126 MHz, $CDCl_3$):

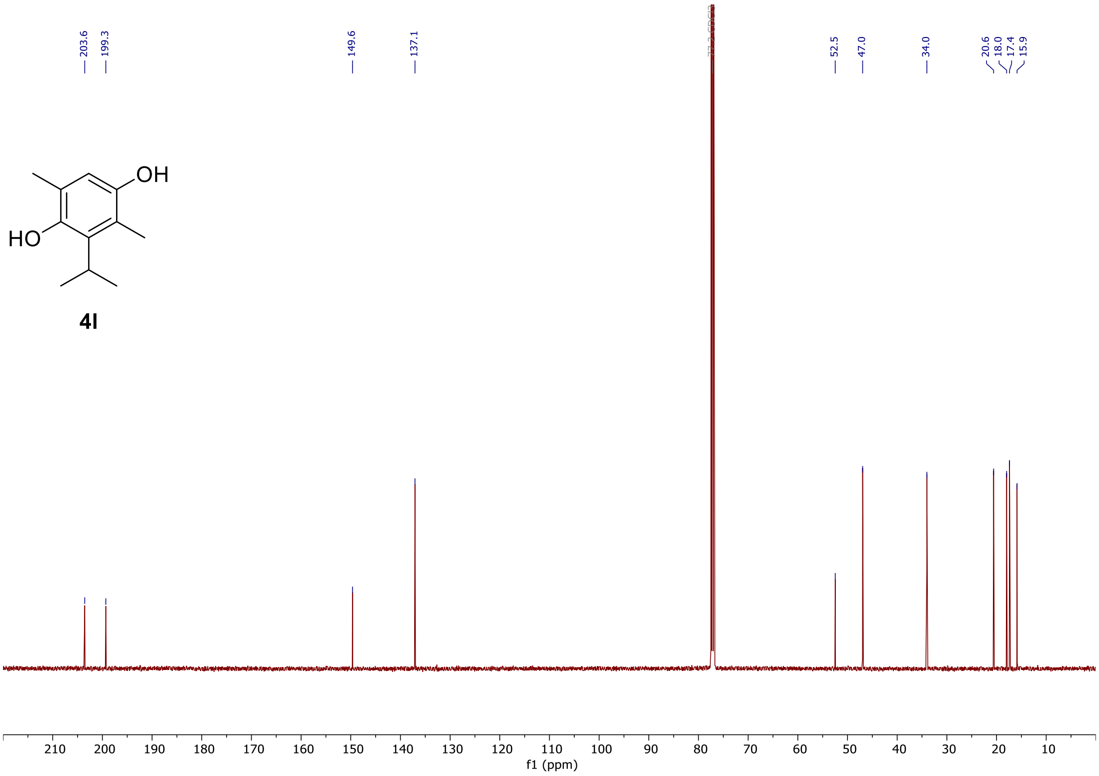

9.3.31. ^{1}H NMR Spectrum of Compound 2m (500 MHz, $CDCl_3$):

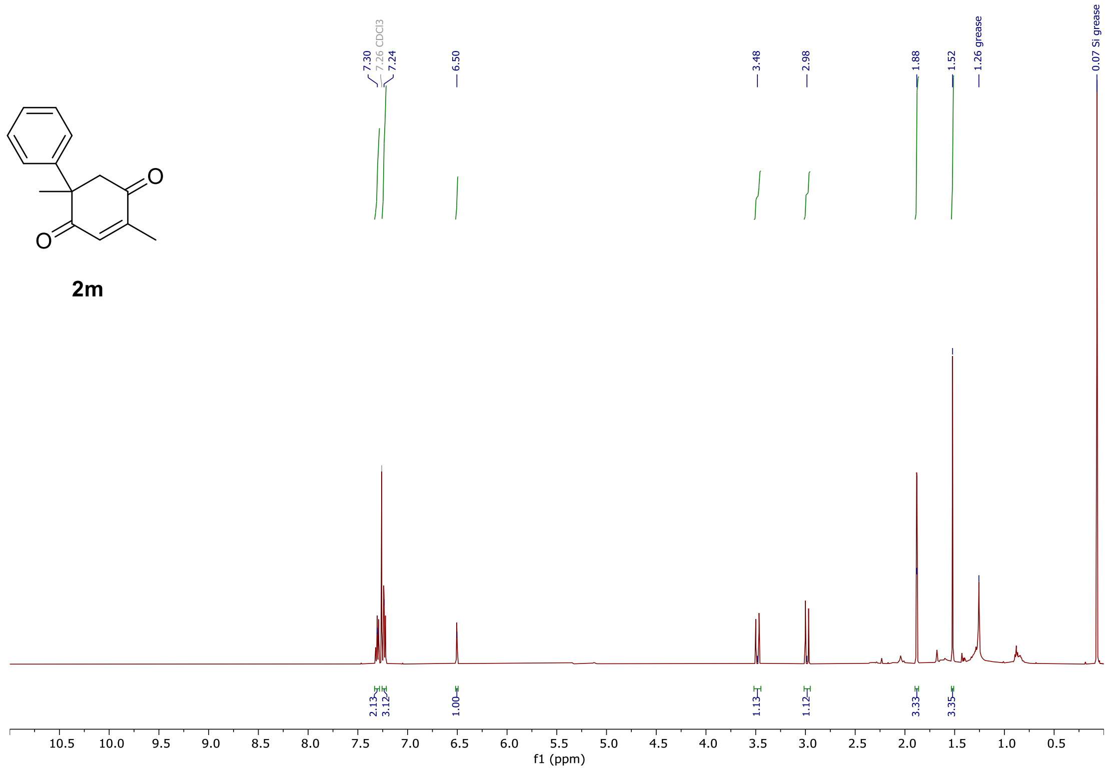

9.3.32. ^{13}C NMR Spectrum of Compound 2m (126 MHz, $CDCl_3$):

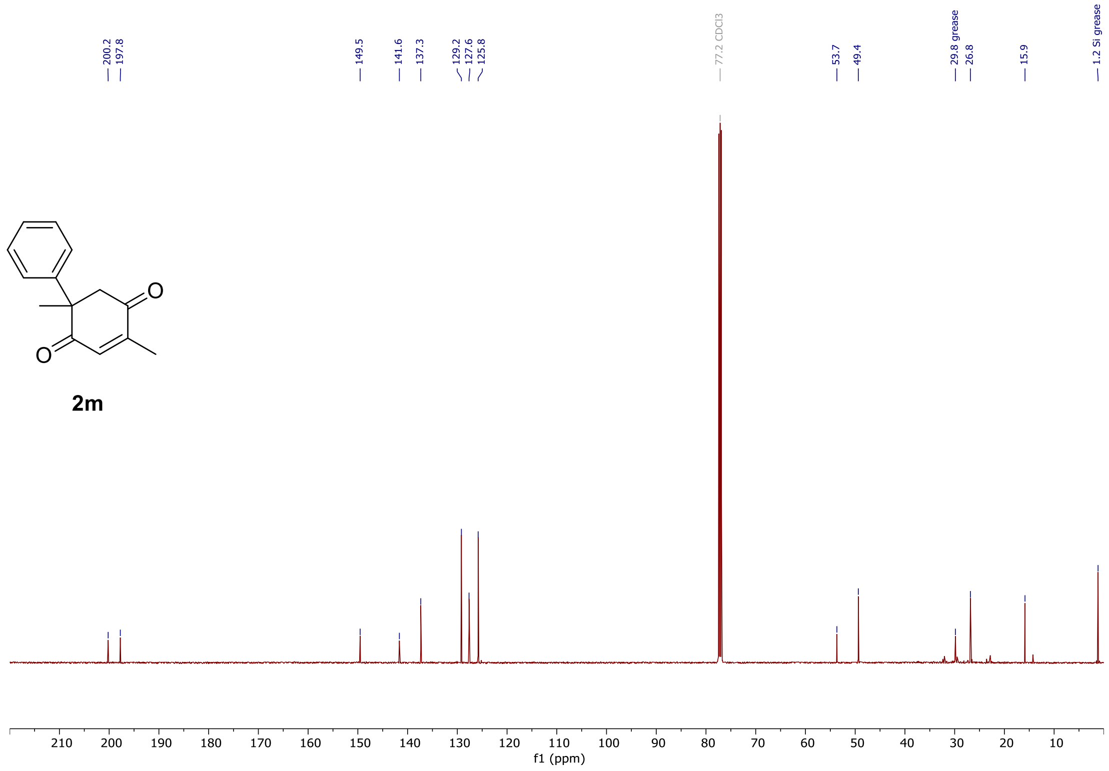

9.3.33. ^{1}H NMR Spectrum of Compound 4m (500 MHz, $CDCl_3$):

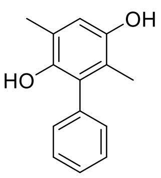

4m

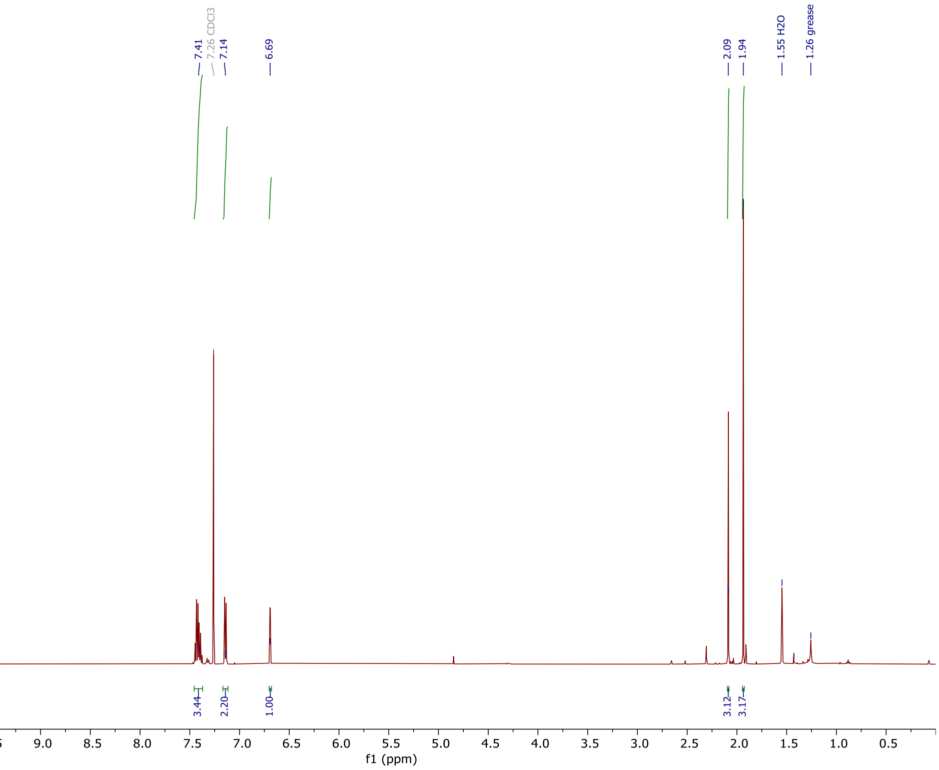

9.3.34. ^{13}C NMR Spectrum of Compound 4m (126 MHz, $CDCl_3$):

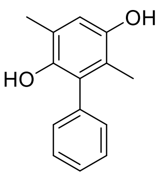

4m

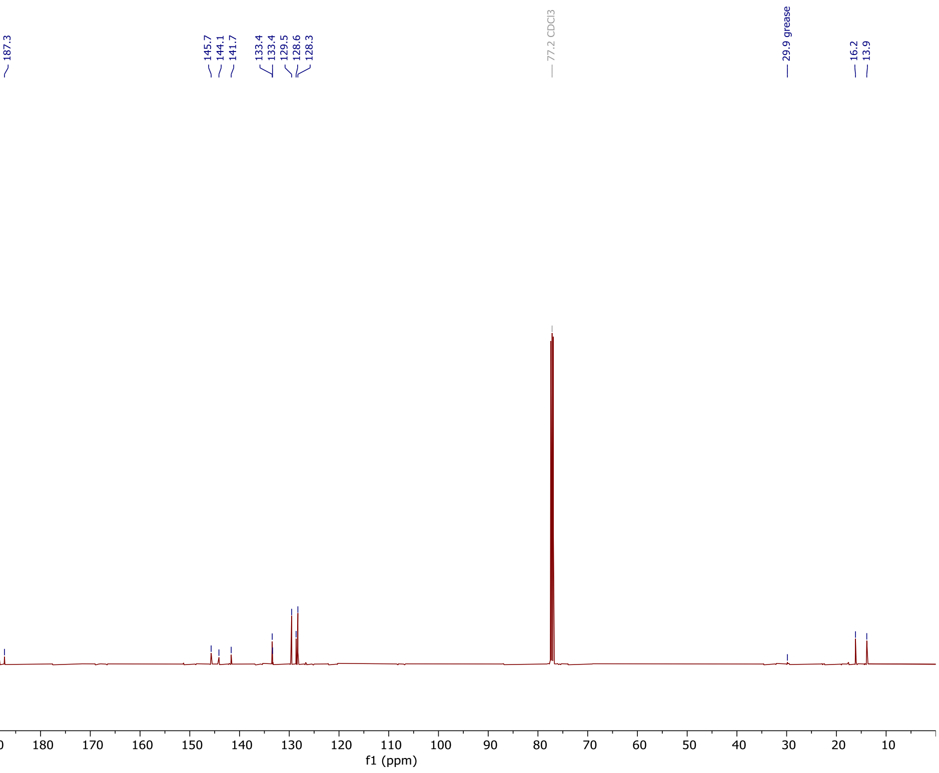

9.3.35. ^{1}H NMR Spectrum of Compounds 2p and 5 (500 MHz, $CDCl_3$):

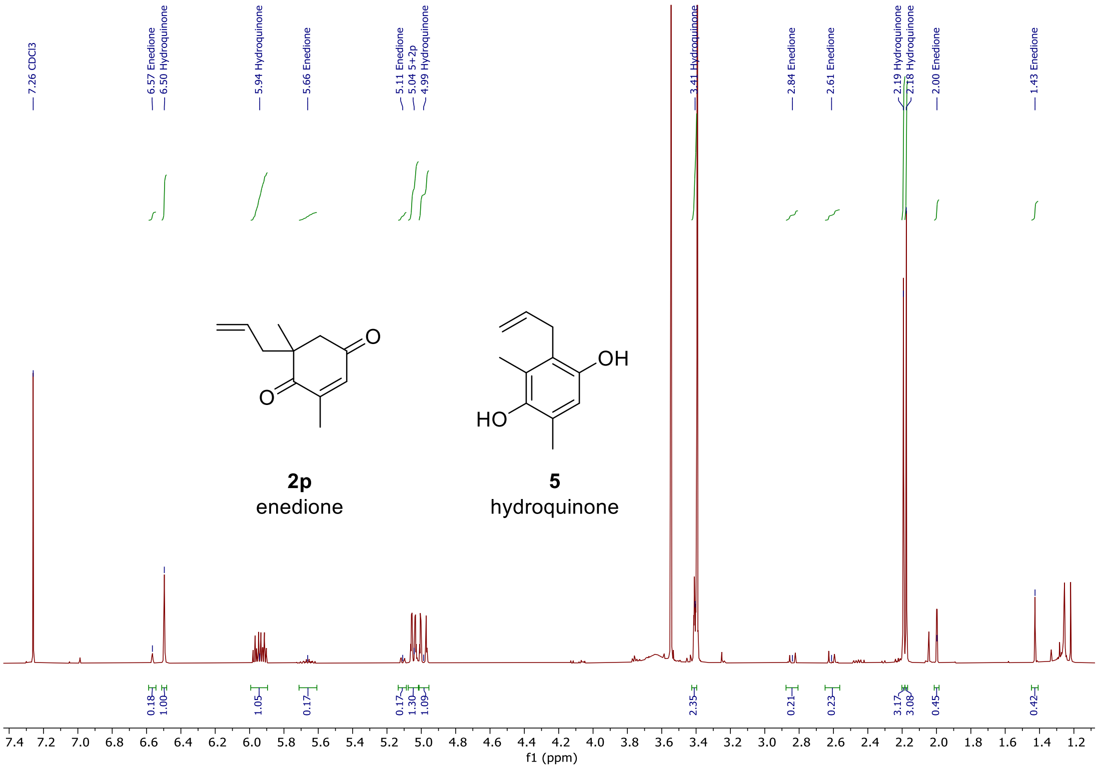

9.3.36. Stacked ^{1}H NMR Spectrum of Compound 1p and Compounds 2p and 5 (500 MHz, $CDCl_3$)

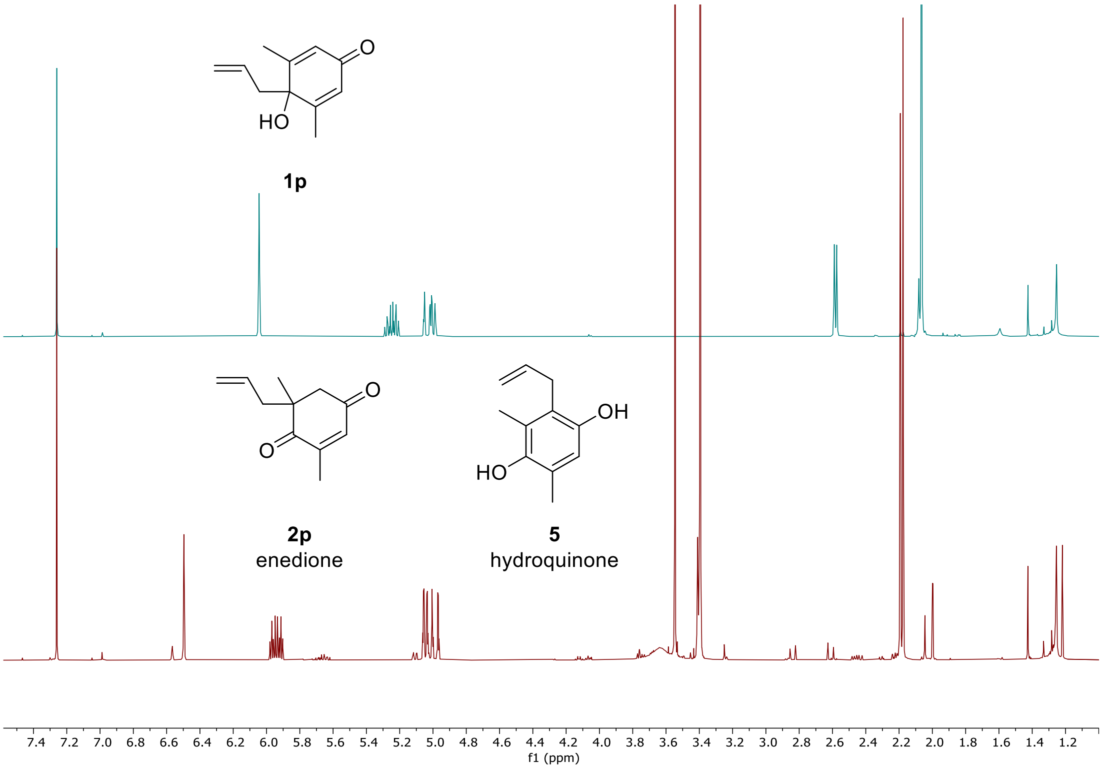

9.4. Enedione derivatives

9.4.1. ^{1}H NMR Spectrum of Compound 6 (500 MHz, $CDCl_3$)

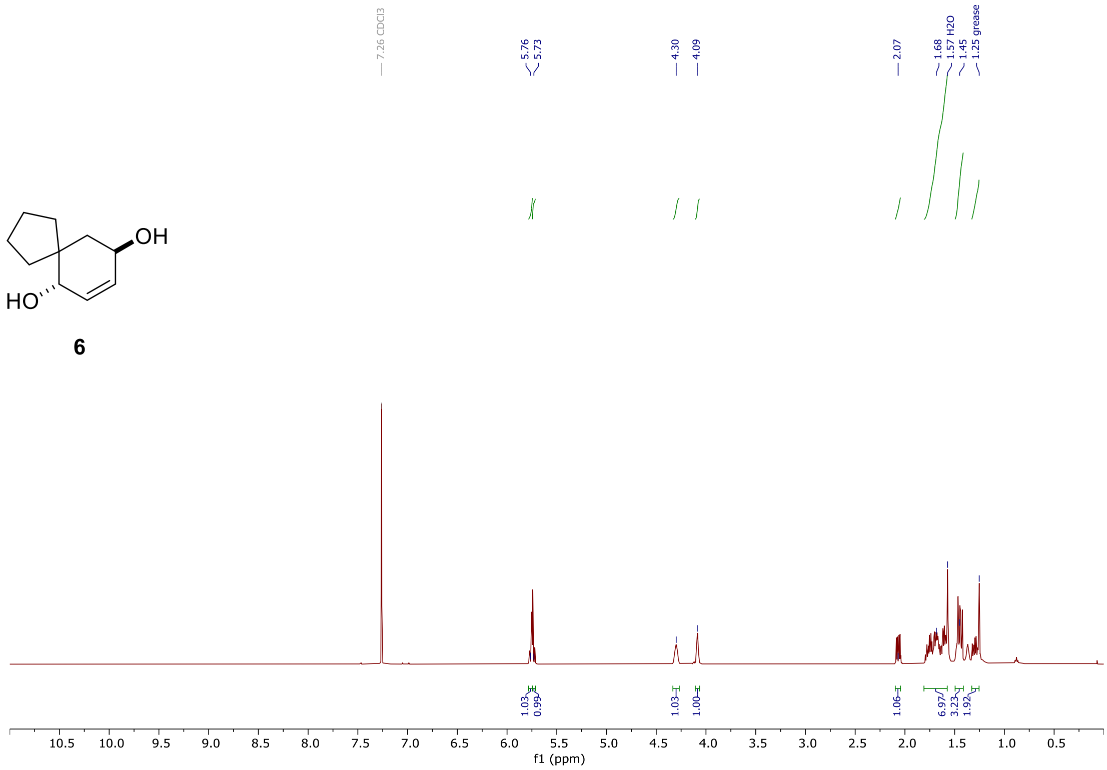

9.4.2. ^{13}C NMR Spectrum of Compound 6 (126 MHz, $CDCl_3$)

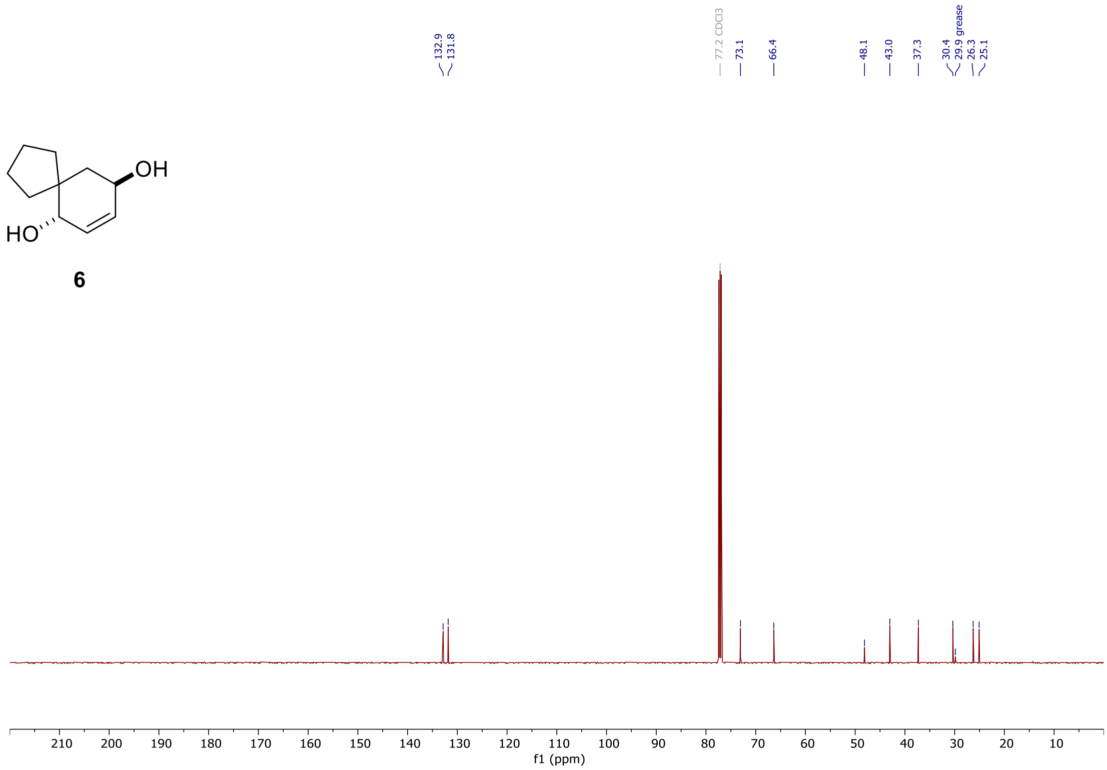

9.4.3. ^{1}H NMR Spectrum of Compound 7 (500 MHz, $CDCl_3$)

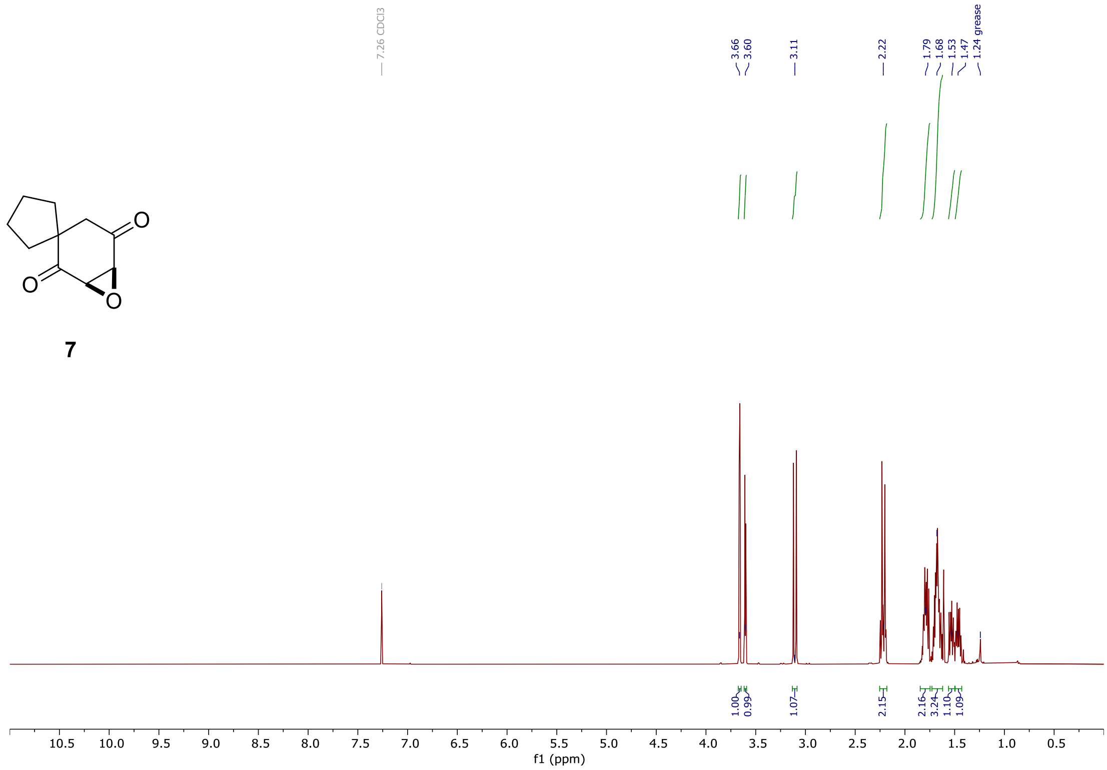

9.4.4. ^{13}C NMR Spectrum of Compound 7 (126 MHz, CDCl3)

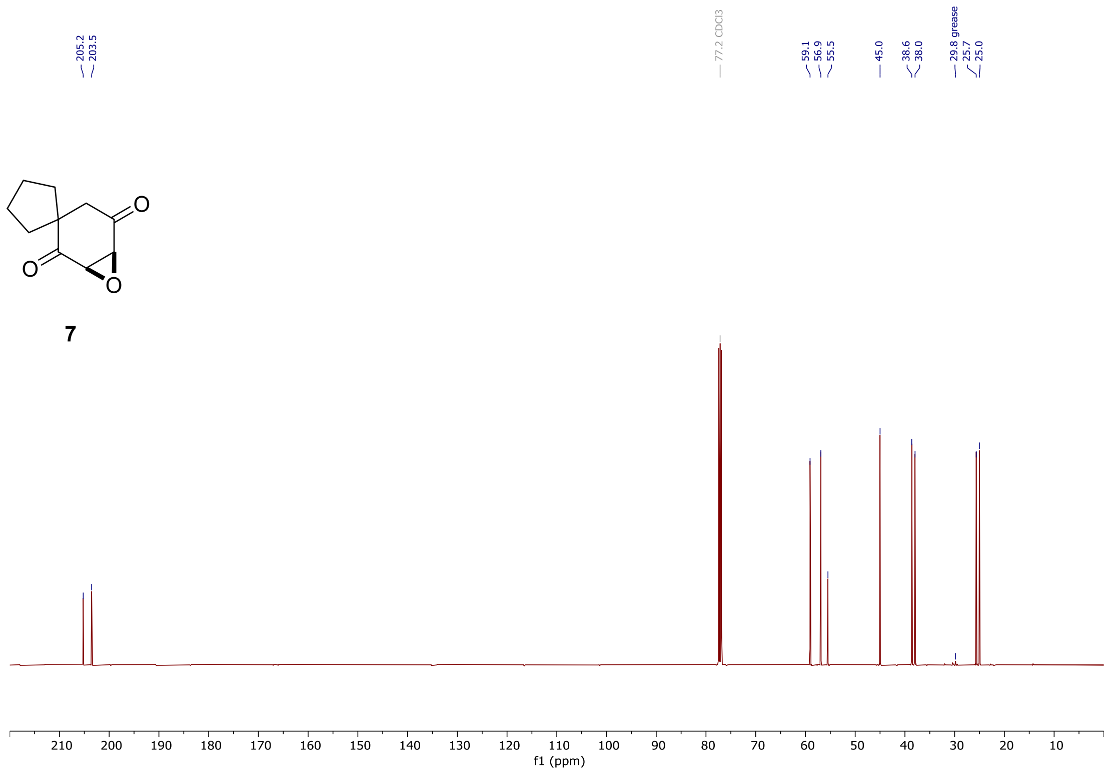

9.4.5. ^{1}H NMR Spectrum of Compounds 8 and S21 (500 MHz, $CDCl_3$)

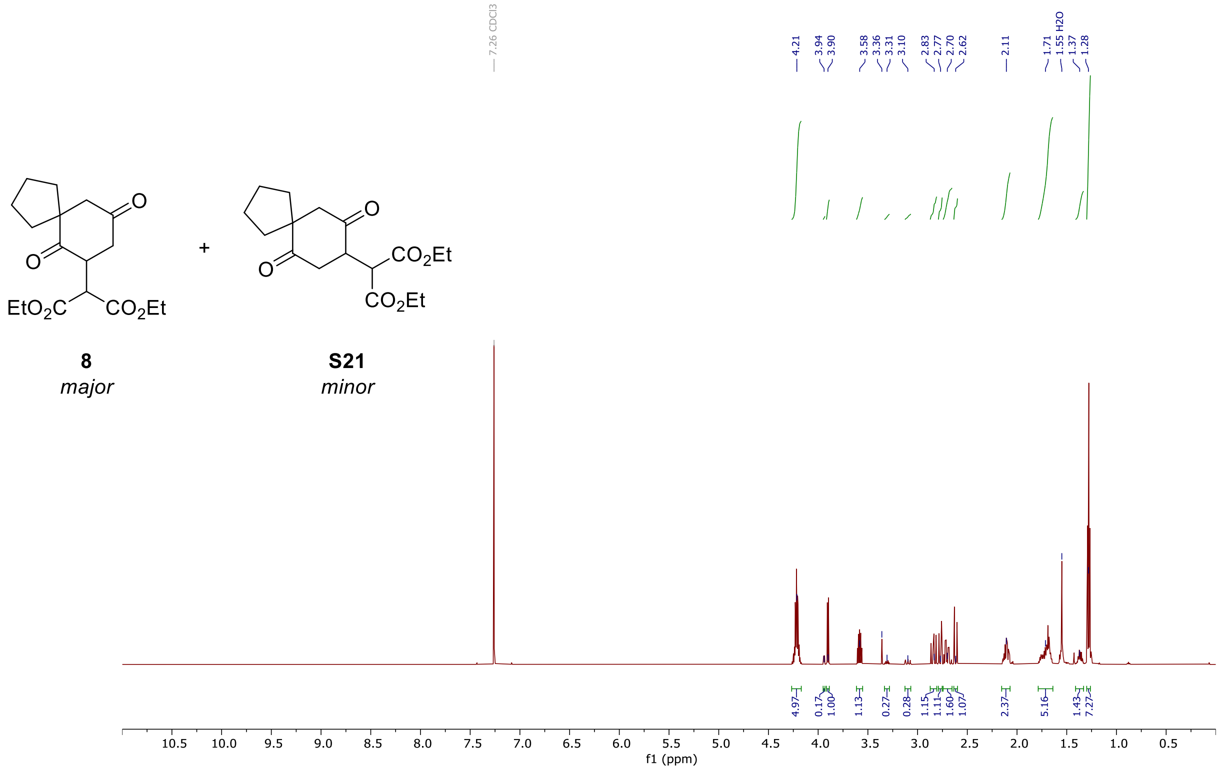

9.4.6. ^{13}C NMR Spectrum of Compounds 8 and S21 (126 MHz, $CDCl_3$)

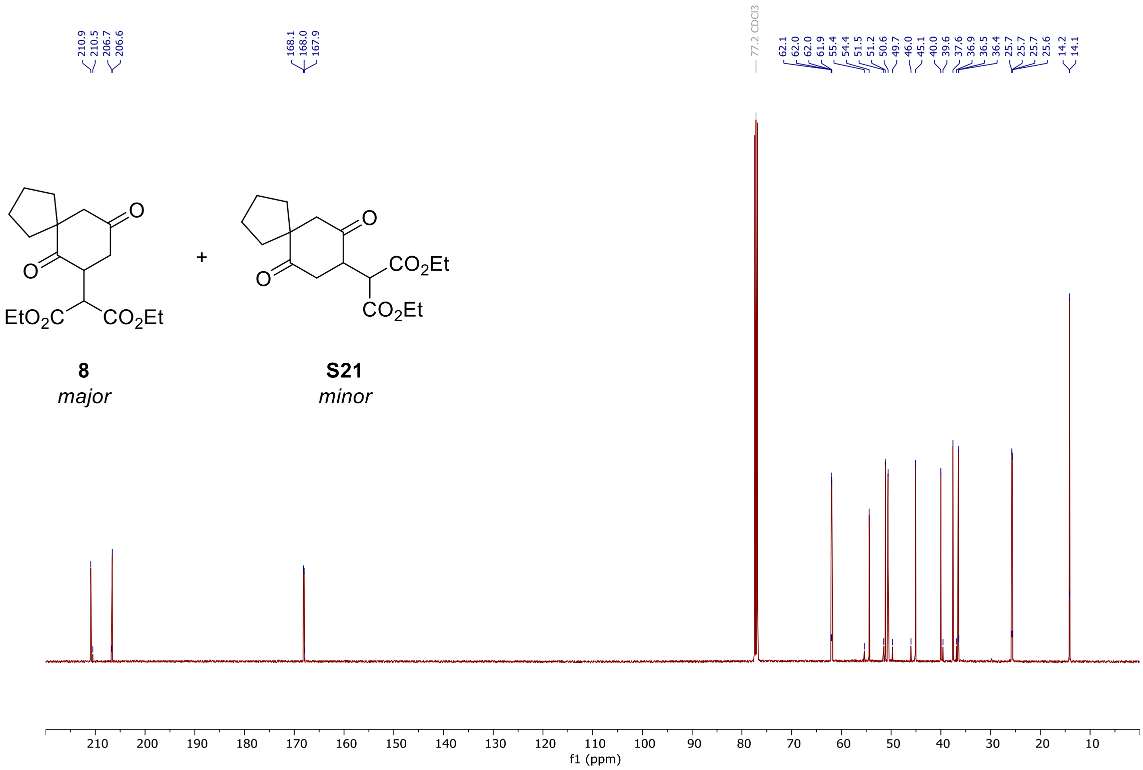

9.4.7. COSY Spectrum of Compounds 8 and S21 (500 MHz, $CDCl_3$)

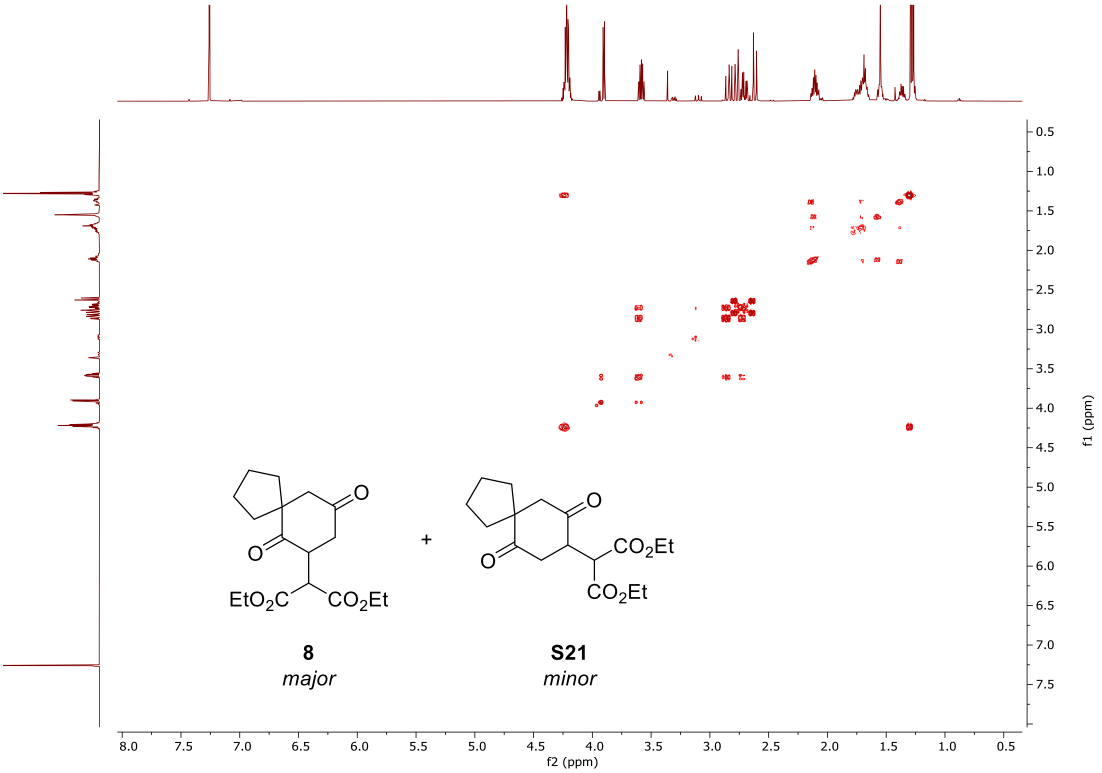

9.4.8. HSQC Spectrum of Compounds 8 and S21 (500 MHz, $CDCl_3$)

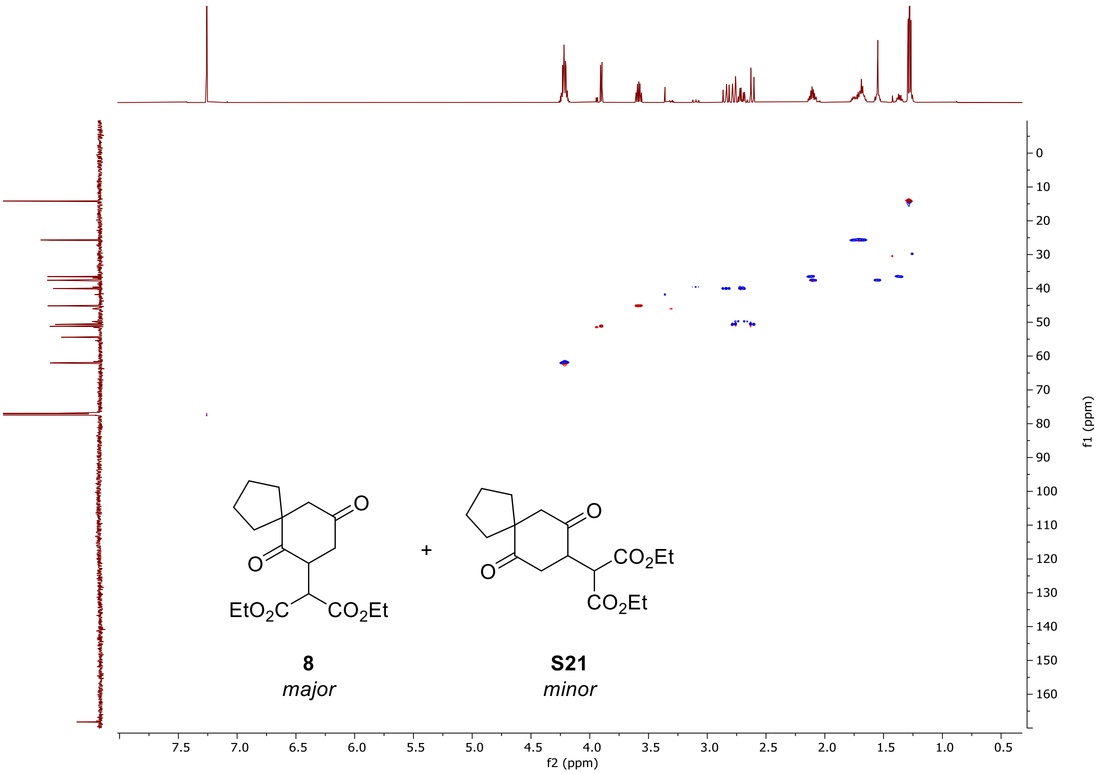

9.4.9. HMBC Spectrum of Compounds 8 and S21 (500 MHz, $CDCl_3$)

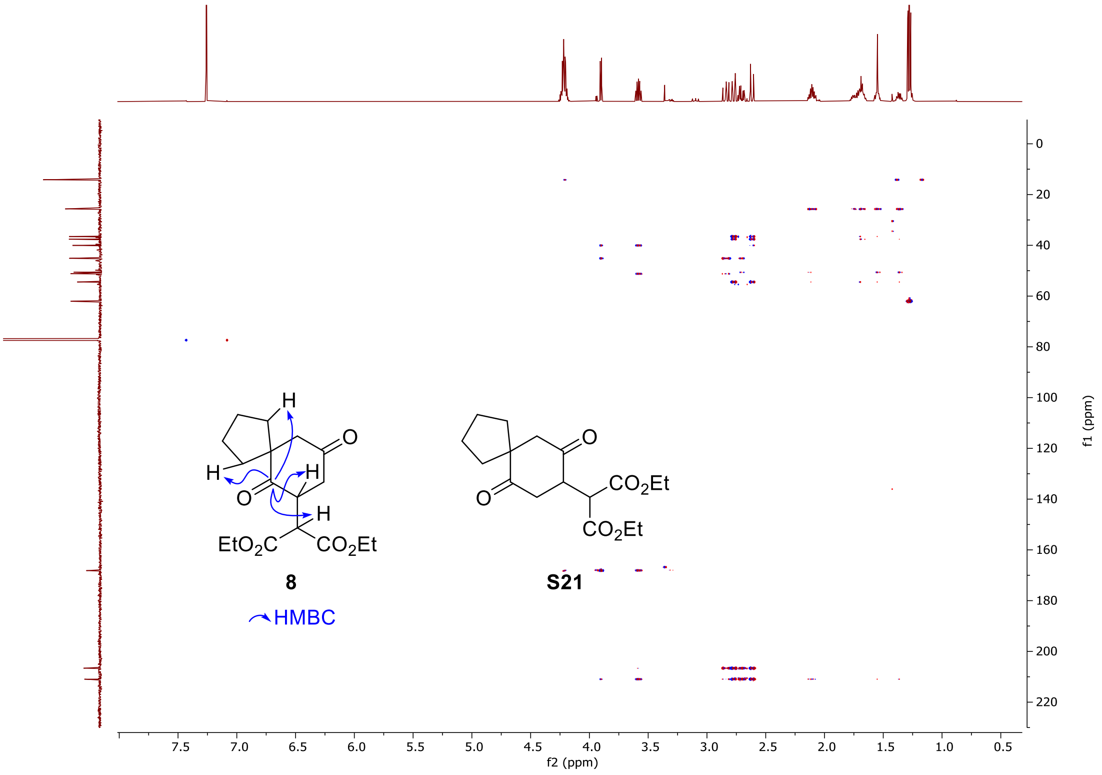

9.4.10. ^{1}H NMR Spectrum of Compound 9 (500 MHz, $CDCl_3$)

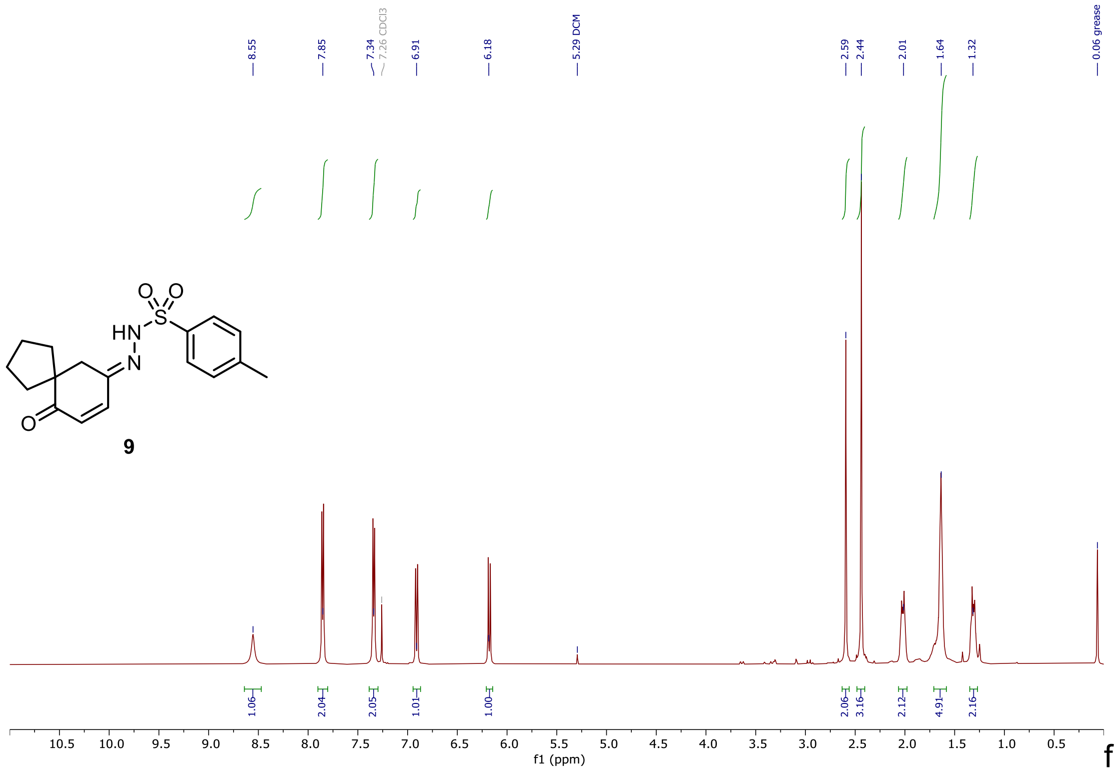

9.4.11. ^{13}C NMR Spectrum of Compound 9 (126 MHz, $CDCl_3$)

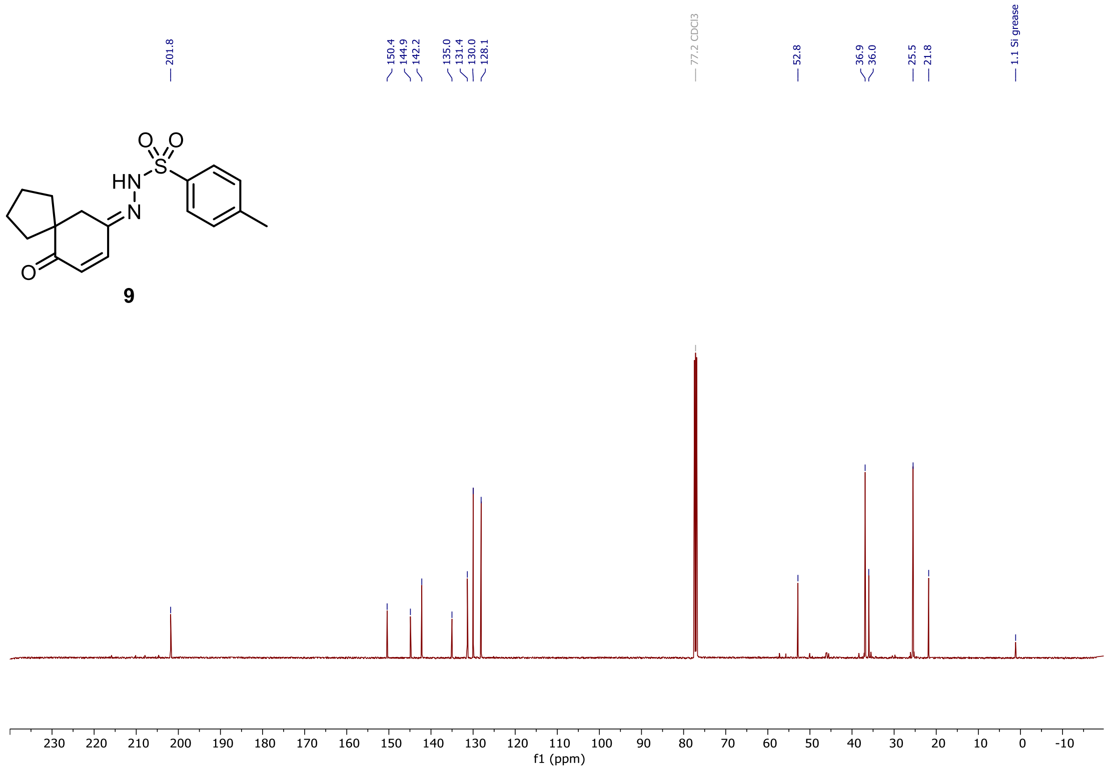

9.4.12. COSY Spectrum of Compound 9 (500 MHz, $CDCl_3$)

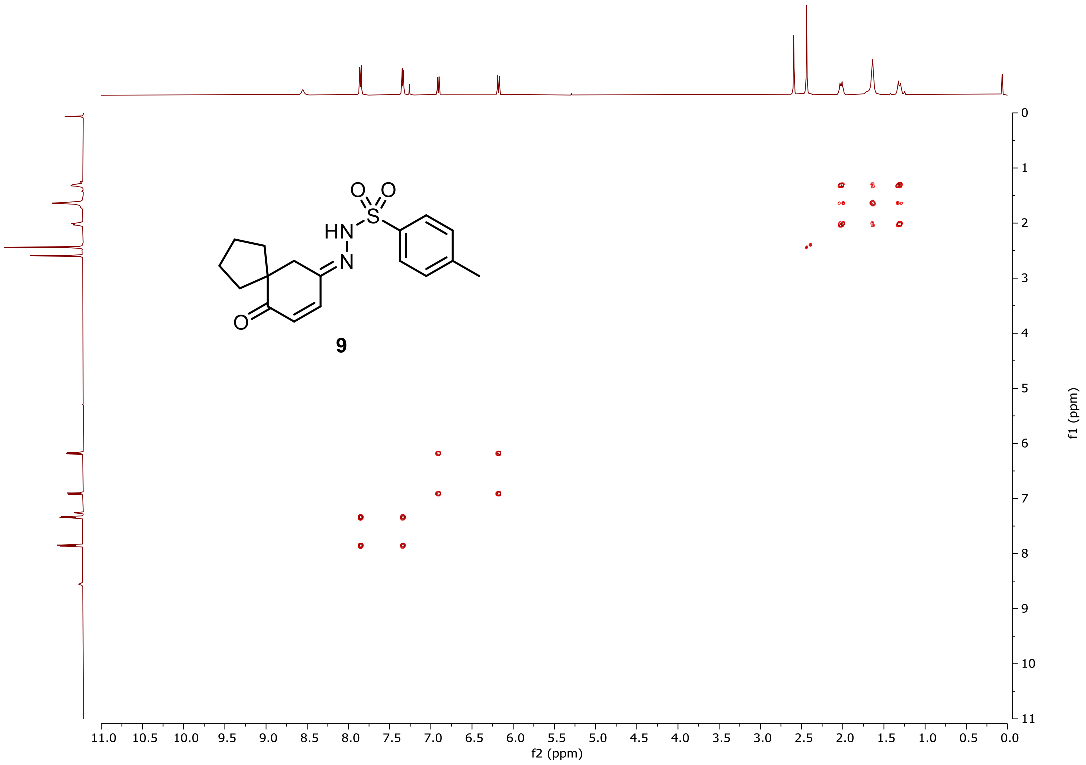

9.4.13. HSQC Spectrum of Compound 9 (500 MHz, $CDCl_3$)

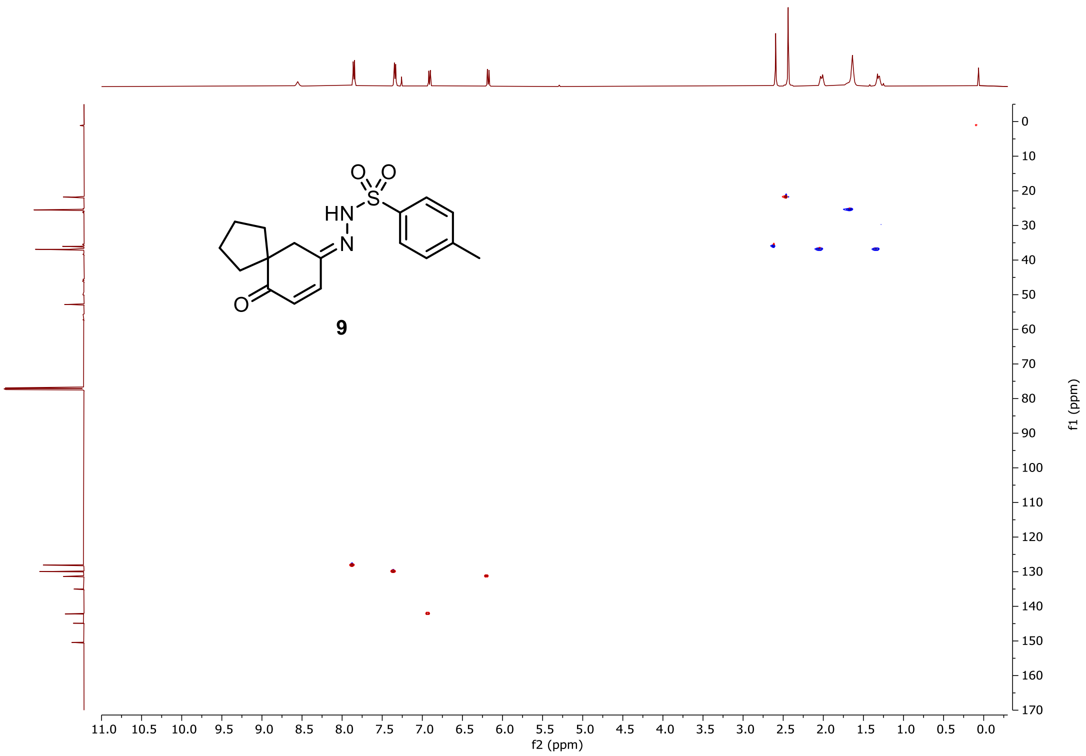

9.4.14. HMBC Spectrum of Compound 9 (500 MHz, $CDCl_3$)

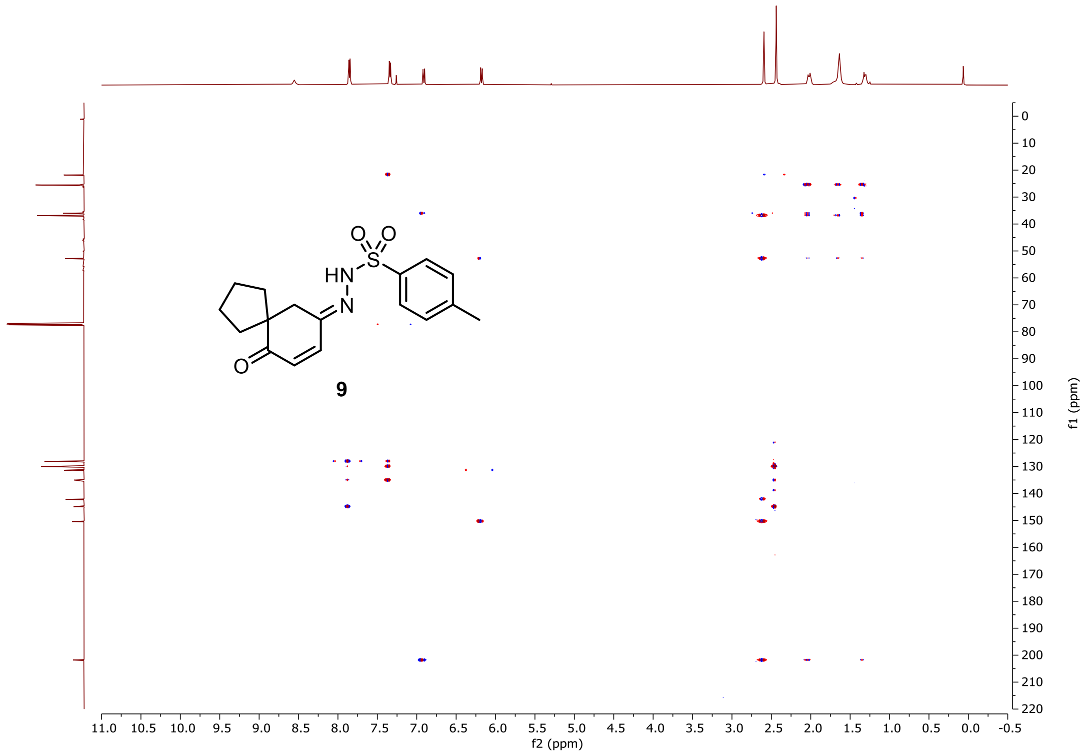

9.4.15. NOESY Spectrum of Compound 9 (500 MHz, $CDCl_3$, diagonal suppressed)

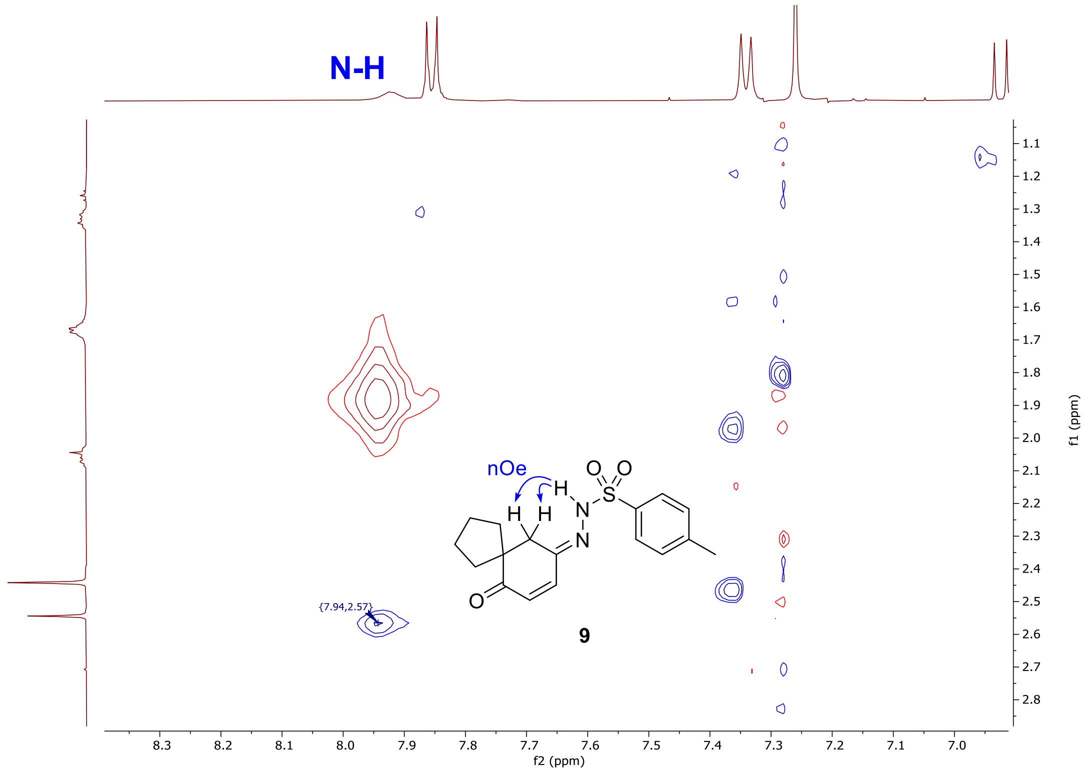

9.4.16. ^{1}H NMR Spectrum of Compound 10 (500 MHz, $CDCl_3$)

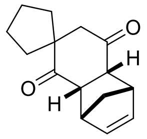

10

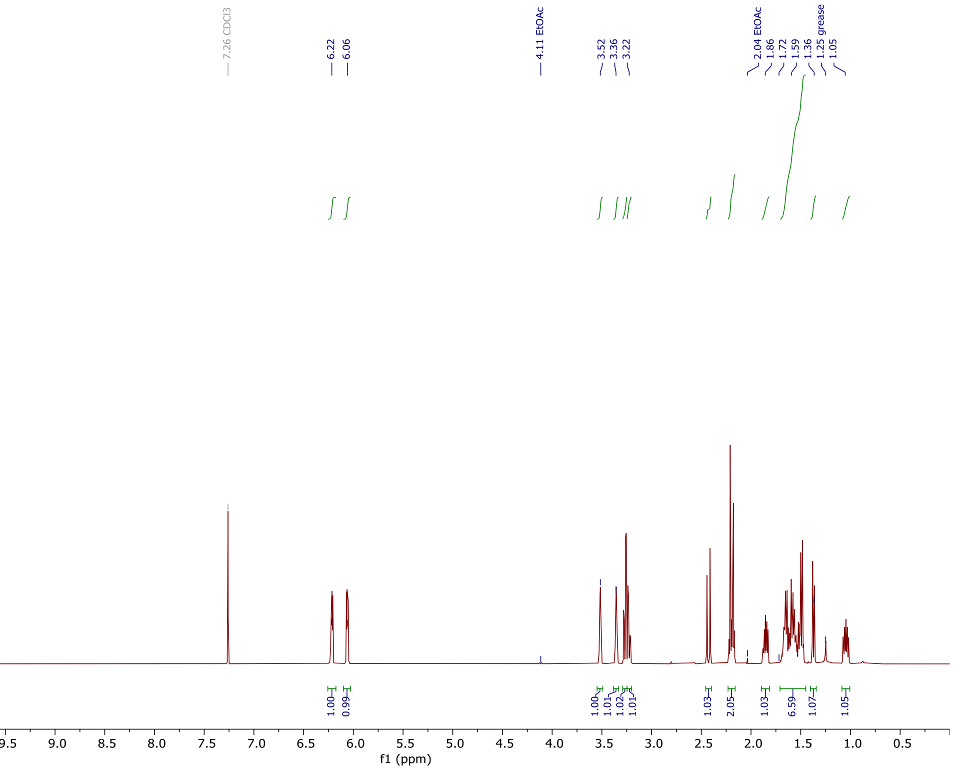

9.4.17. CP DA ^{13}C NMR Spectrum of Compound 10 (126 MHz, $CDCl_3$)

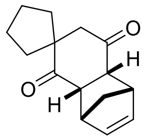

10

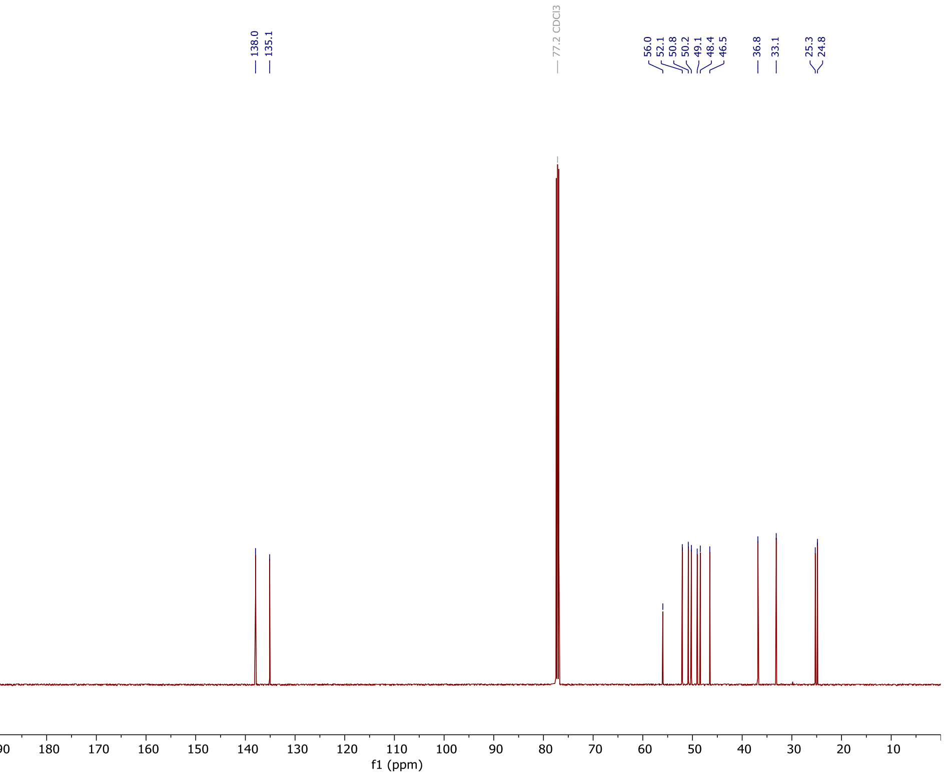

9.4.18. COSY Spectrum of Compound 10 (500 MHz, $CDCl_3$)

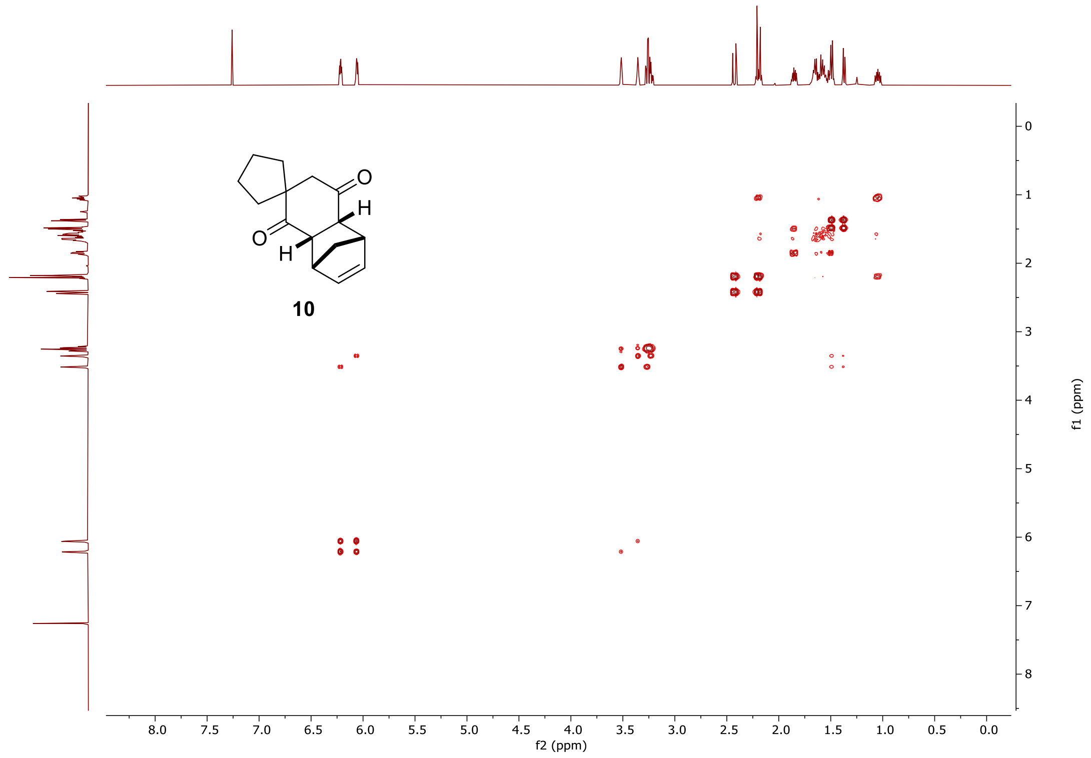

9.4.19. HSQC Spectrum of Compound 10 (500 MHz, $CDCl_3$)

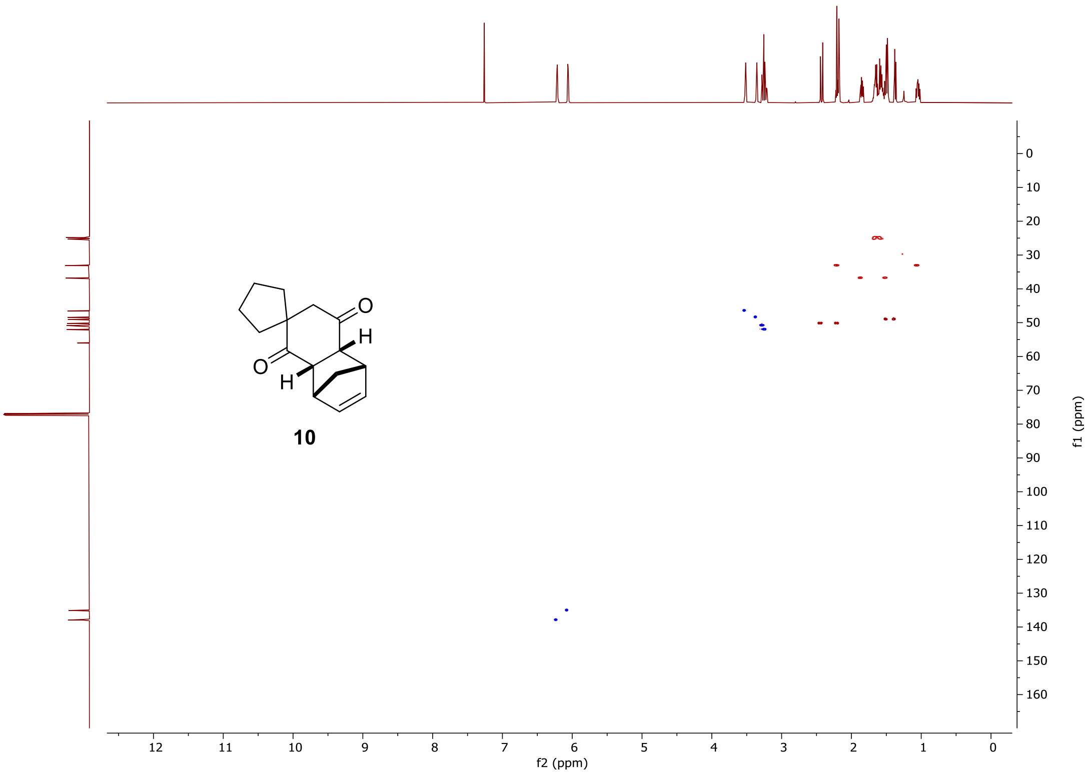

9.4.20. HMBC Spectrum of Compound 10 (500 MHz, CDCl$_3$)

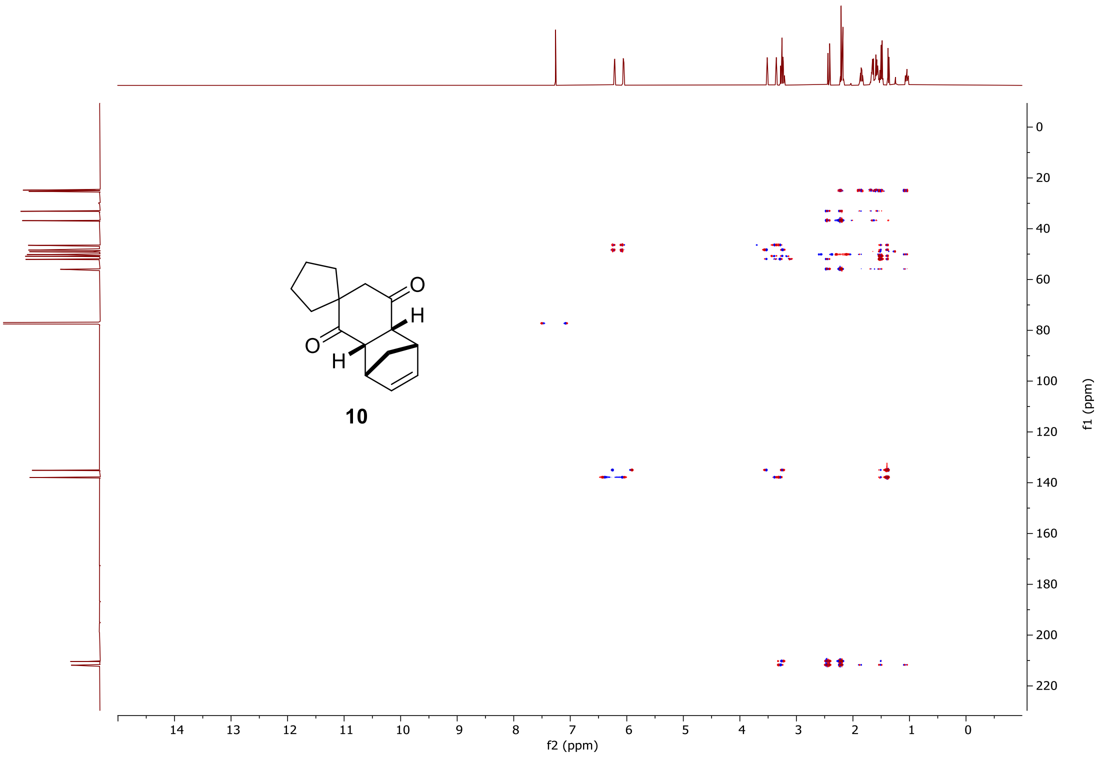

9.4.21. NOESY Spectrum of Compound 10 (500 MHz, CDCl$_3$, diagonal suppressed)

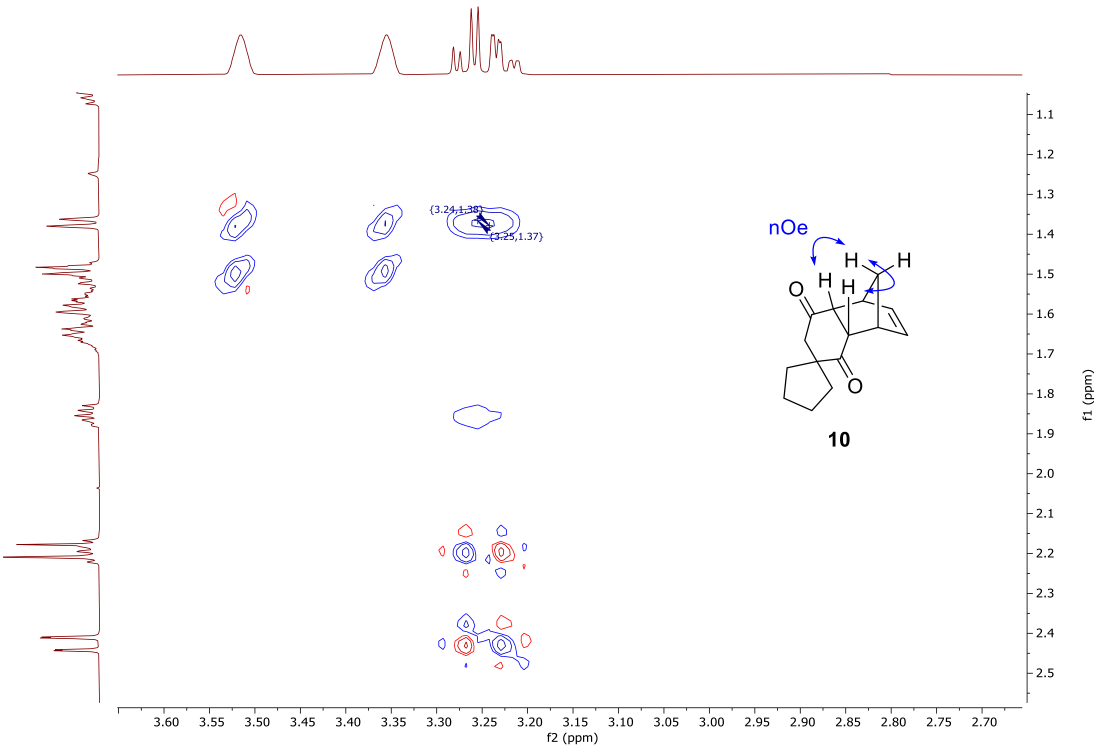

9.4.22. ^{1}H NMR Spectrum of Compounds 11 and S22 (500 MHz, $CDCl_3$)

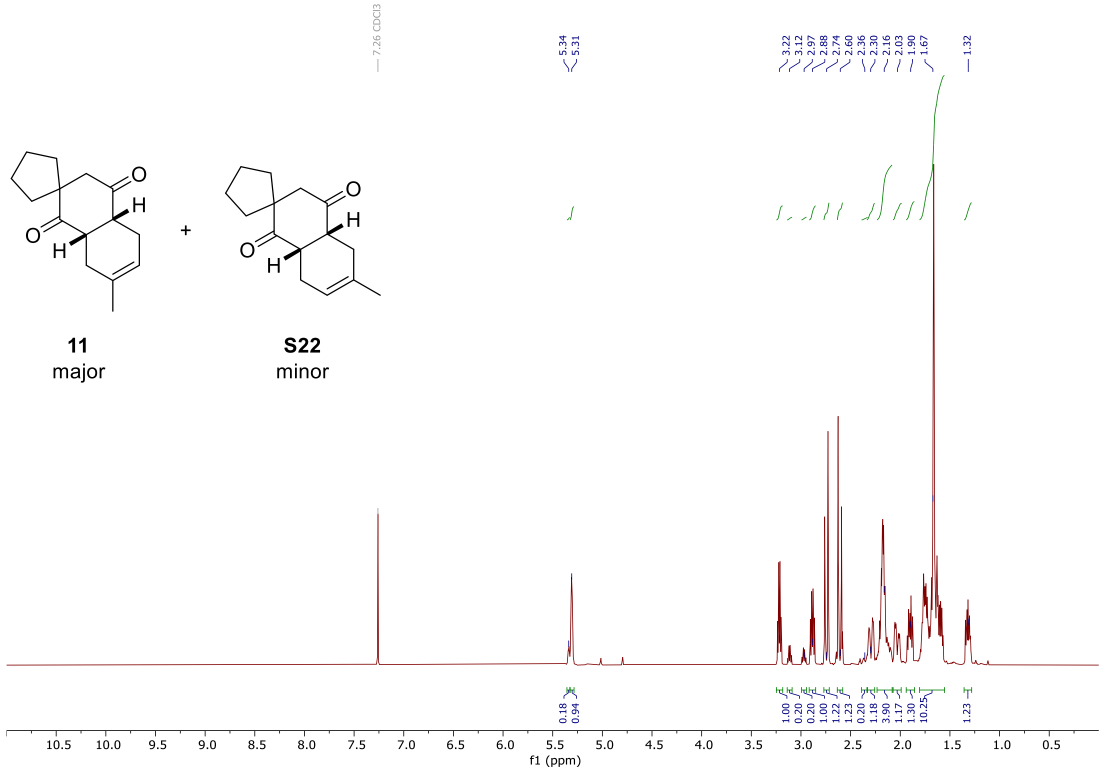

9.4.23. ^{13}C NMR Spectrum of Compounds 11 and S22 (126 MHz, $CDCl_3$)

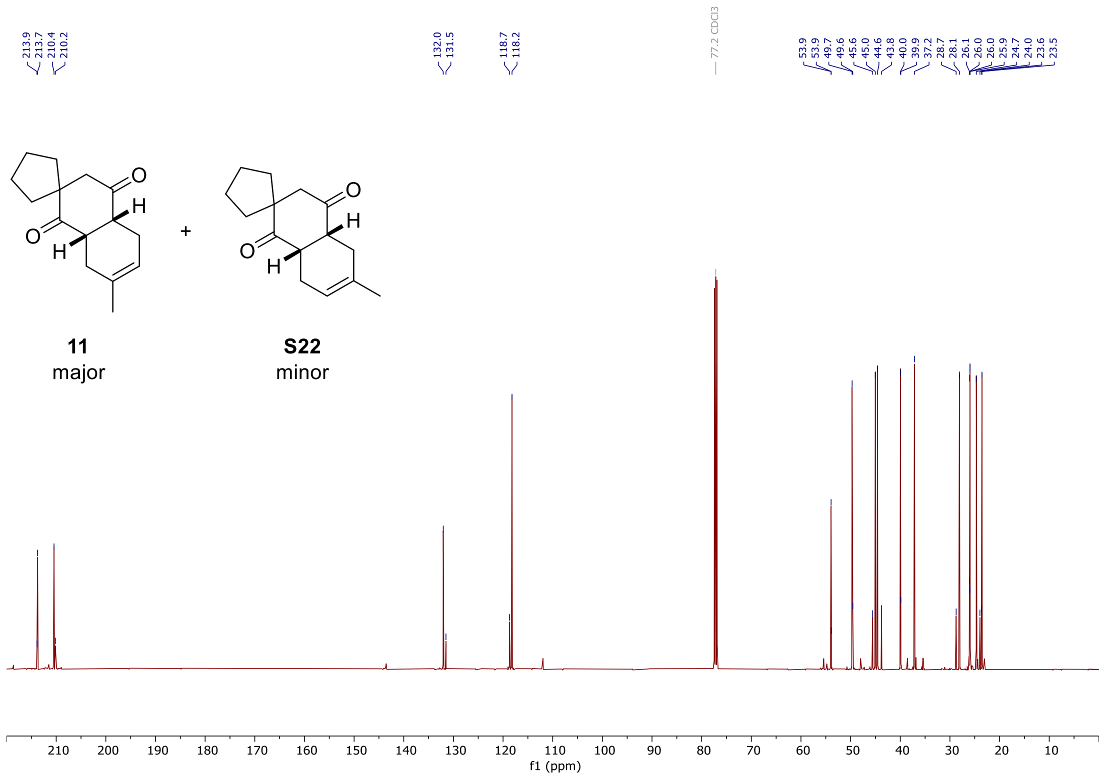

9.4.24. COSY Spectrum of Compounds 11 and S22 (500 MHz, CDCl3)

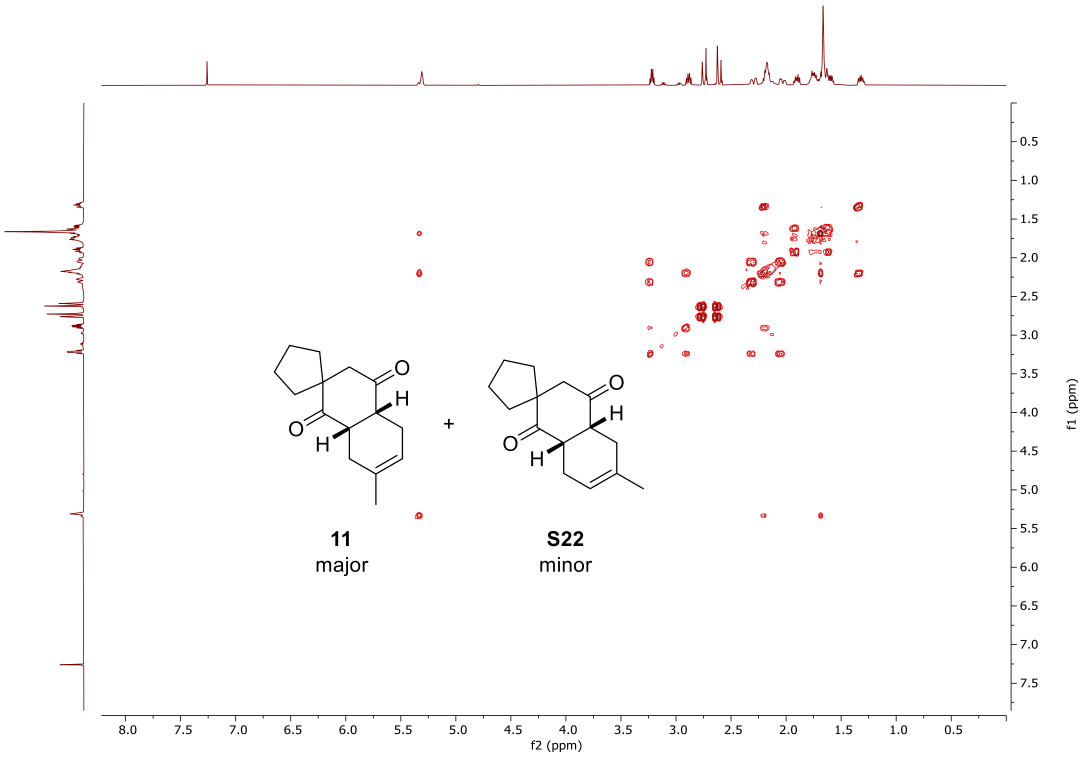

9.4.25. HSQC Spectrum of Compounds 11 and S22 (500 MHz, CDCl3)

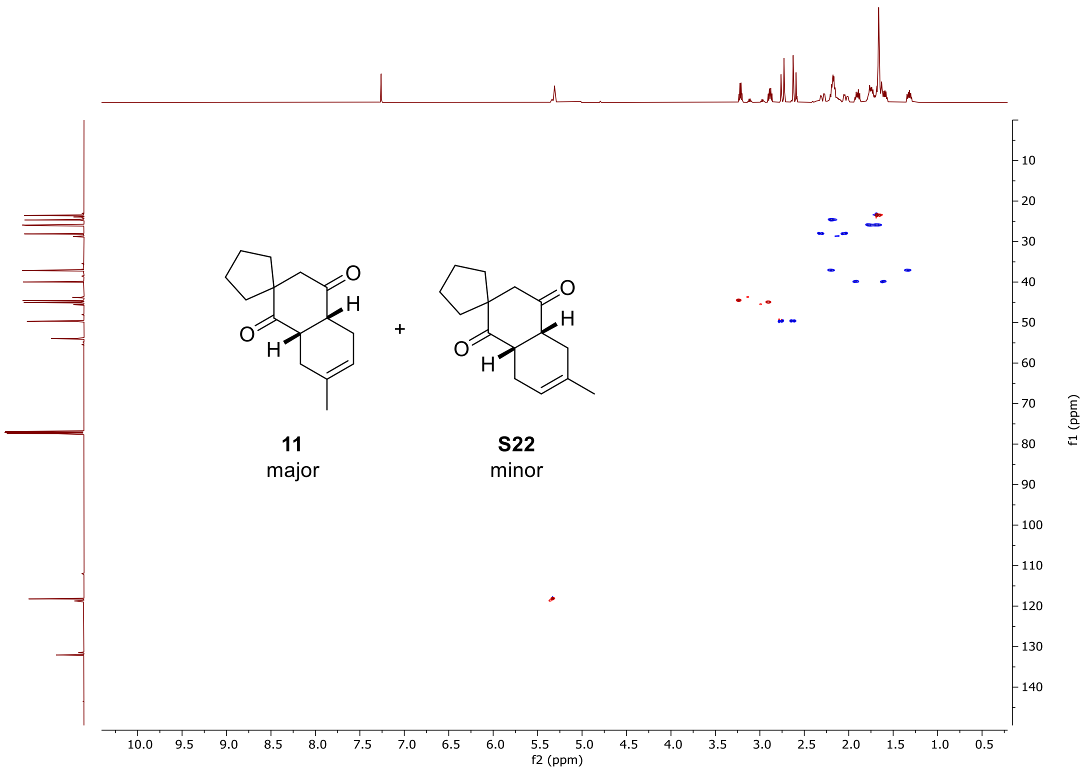

9.4.26. HMBC Spectrum of Compounds 11 and S22 (500 MHz, CDCl$_3$)

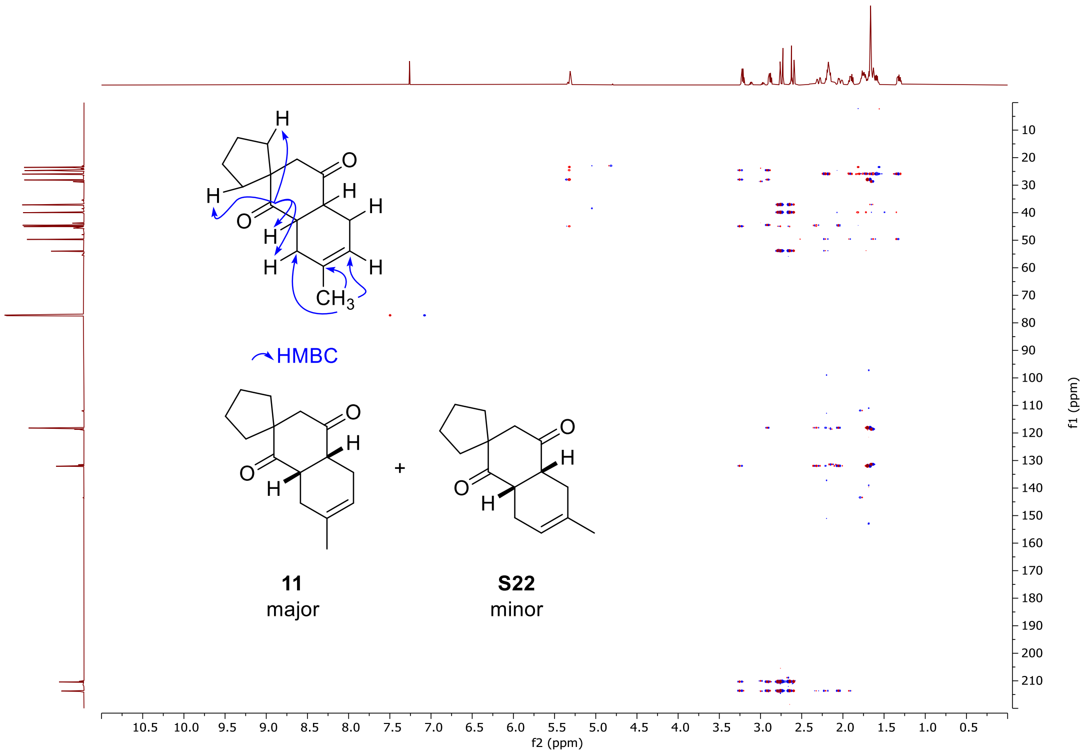

9.4.27. ^{1}H NMR Spectrum of Compound 12 (601 MHz, $CDCl_3$)

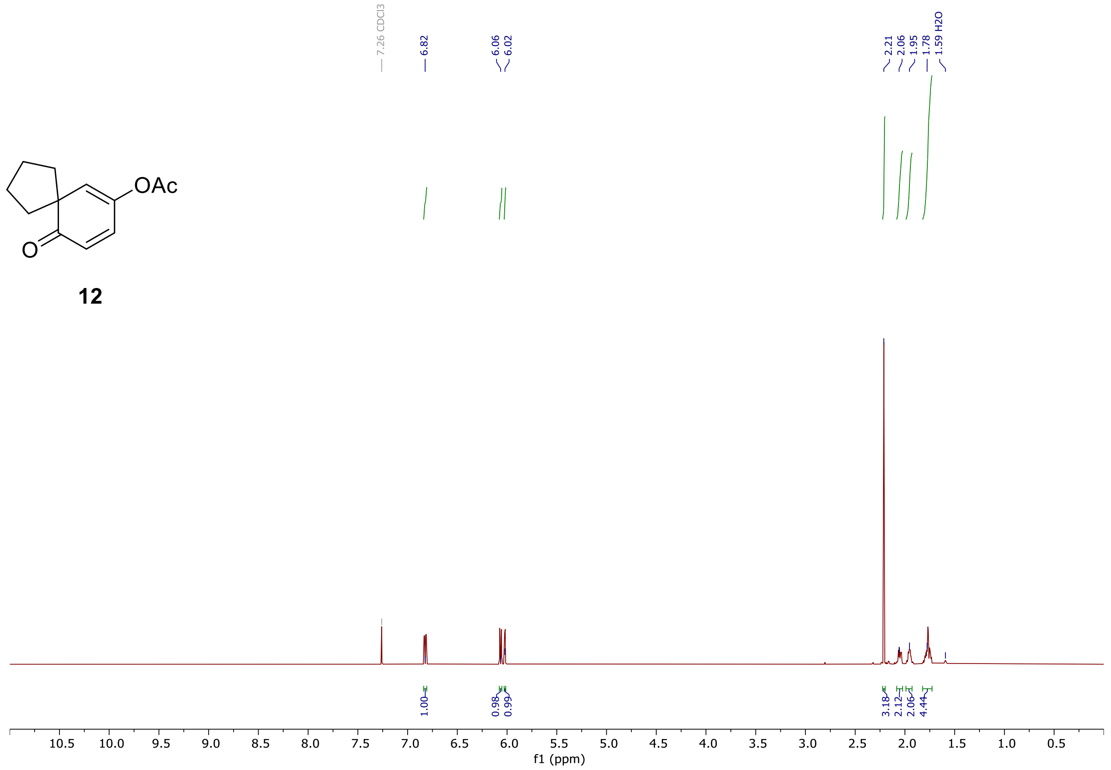

9.4.28. ^{13}C NMR Spectrum of Compound 12 (151 MHz, $CDCl_3$)

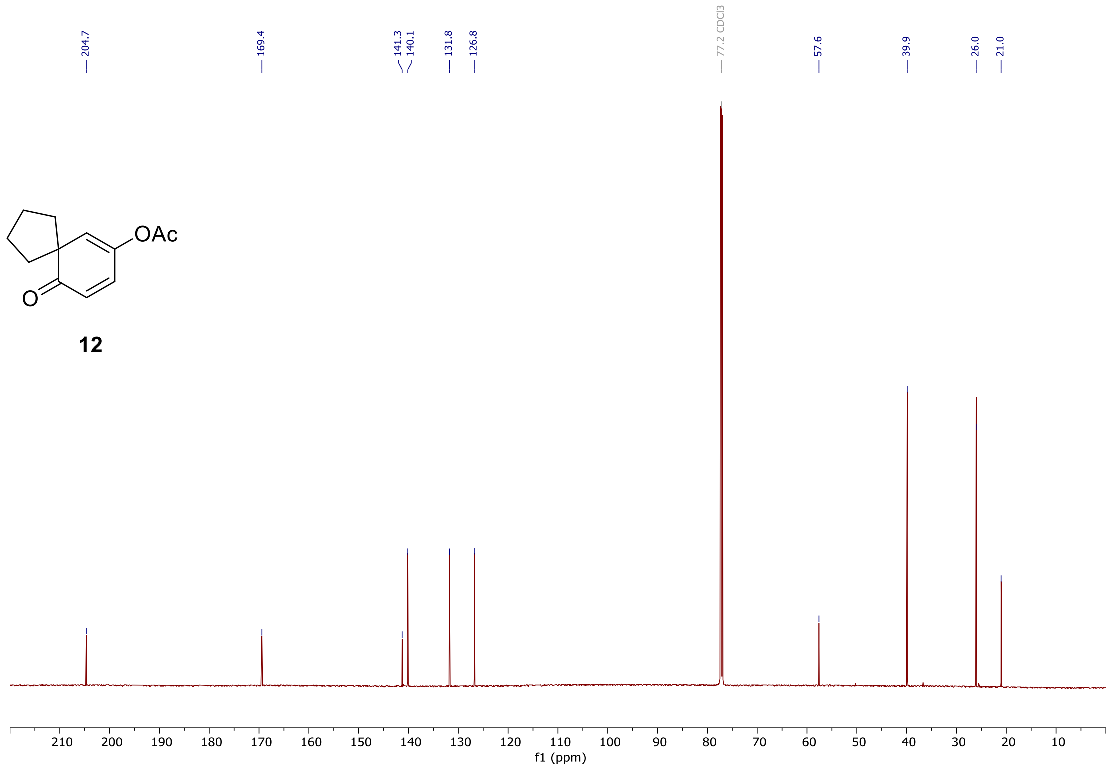

9.4.29. ^{1}H NMR Spectrum of Compound 13 (500 MHz, $CDCl_3$)

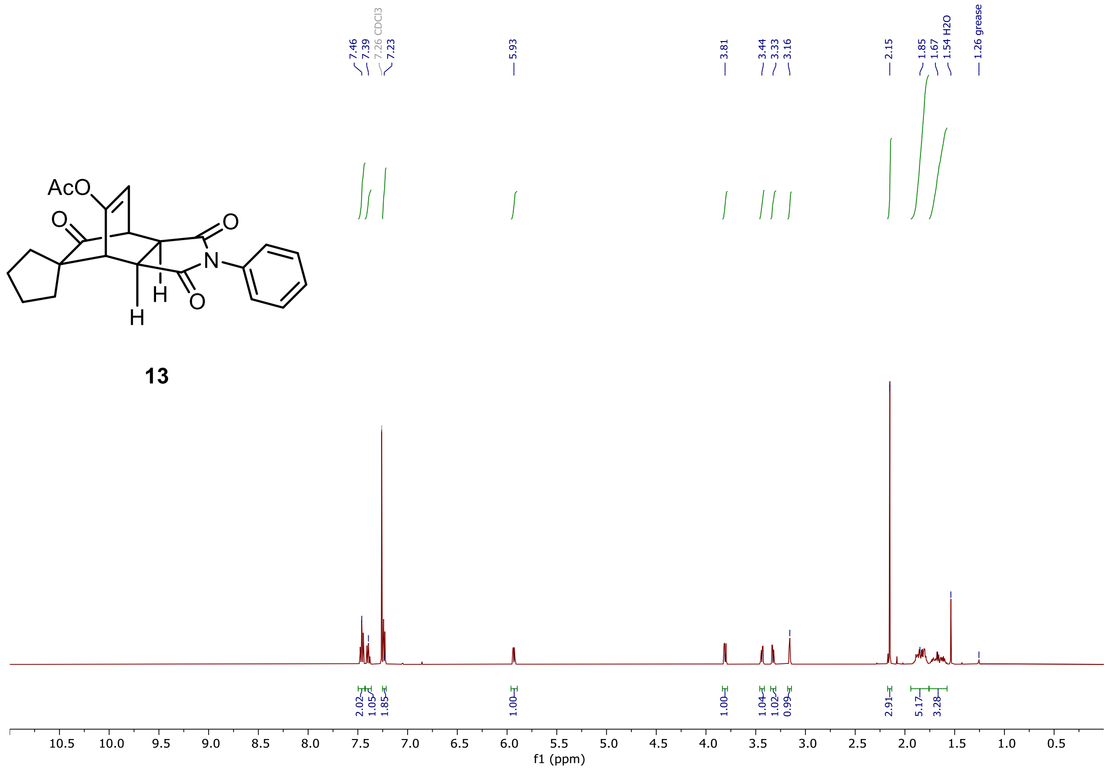

9.4.30. ^{13}C NMR Spectrum of Compound 13 (126 MHz, $CDCl_3$)

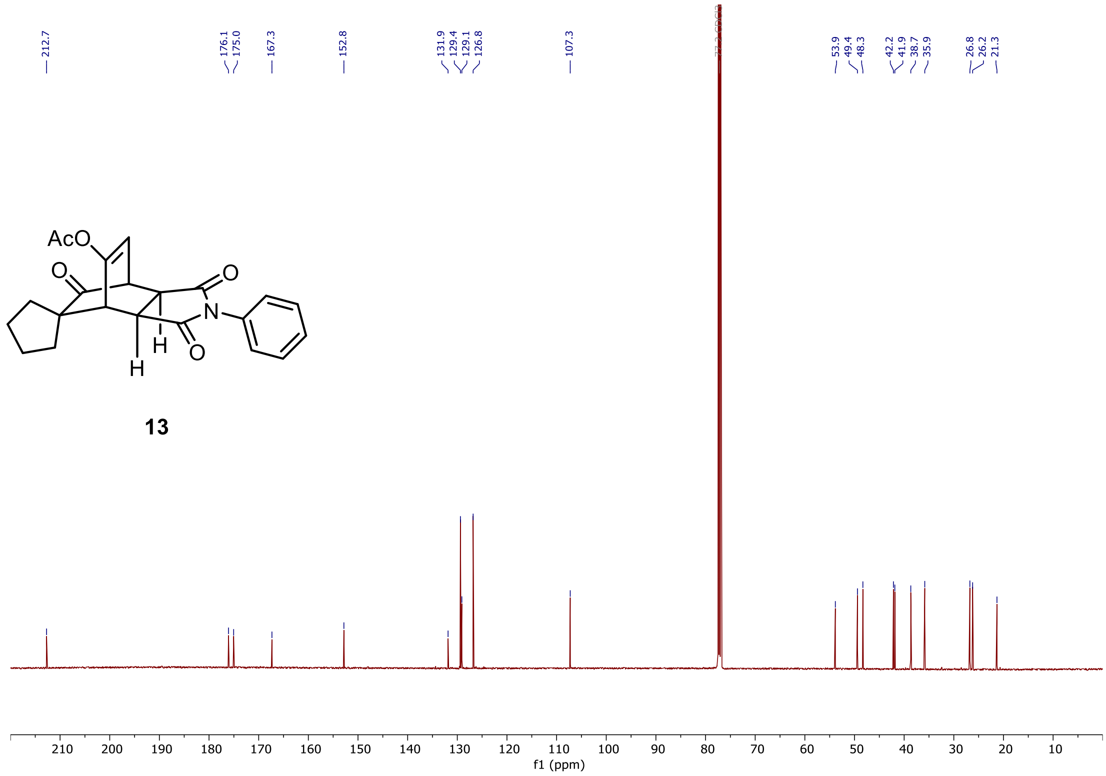

9.4.31. ^{1}H NMR Spectrum of Compound 14 (500 MHz, $CDCl_3$)

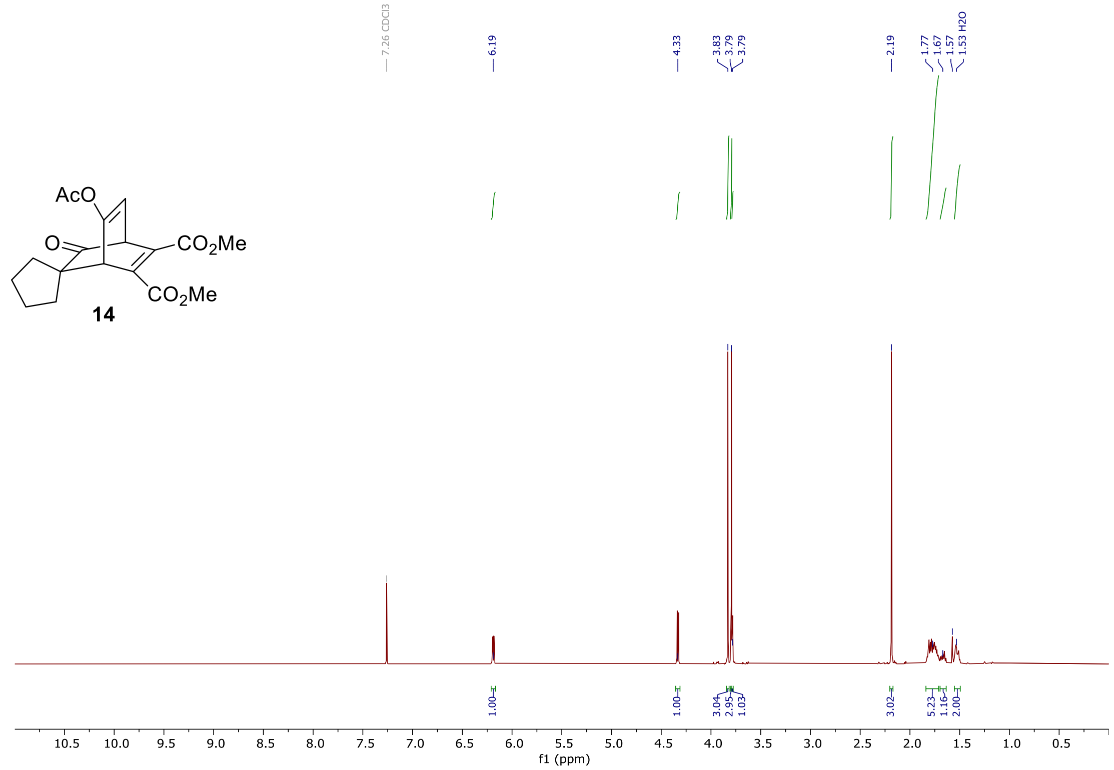

9.4.32. ^{13}C NMR Spectrum of Compound 14 (126 MHz, $CDCl_3$)

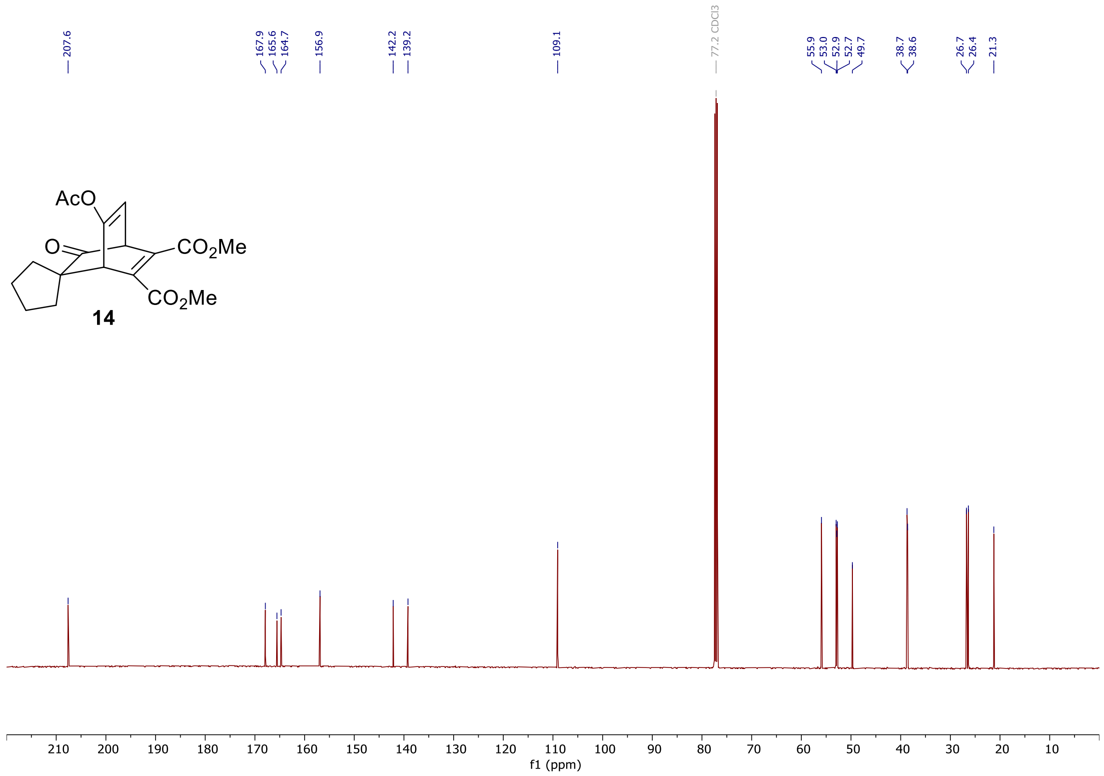

9.4.33. ^{1}H NMR Spectrum of Compound 15 (601 MHz, $CDCl_3$)

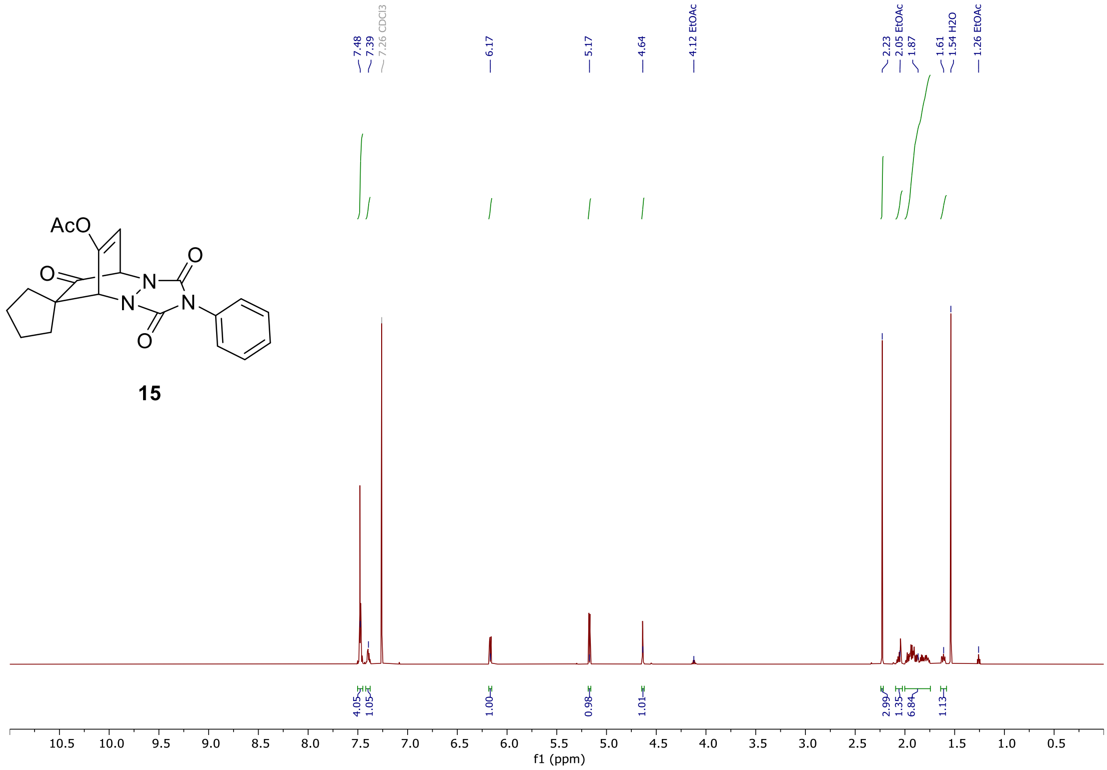

9.4.34. ^{13}C NMR Spectrum of Compound 15 (126 MHz, $CDCl_3$)

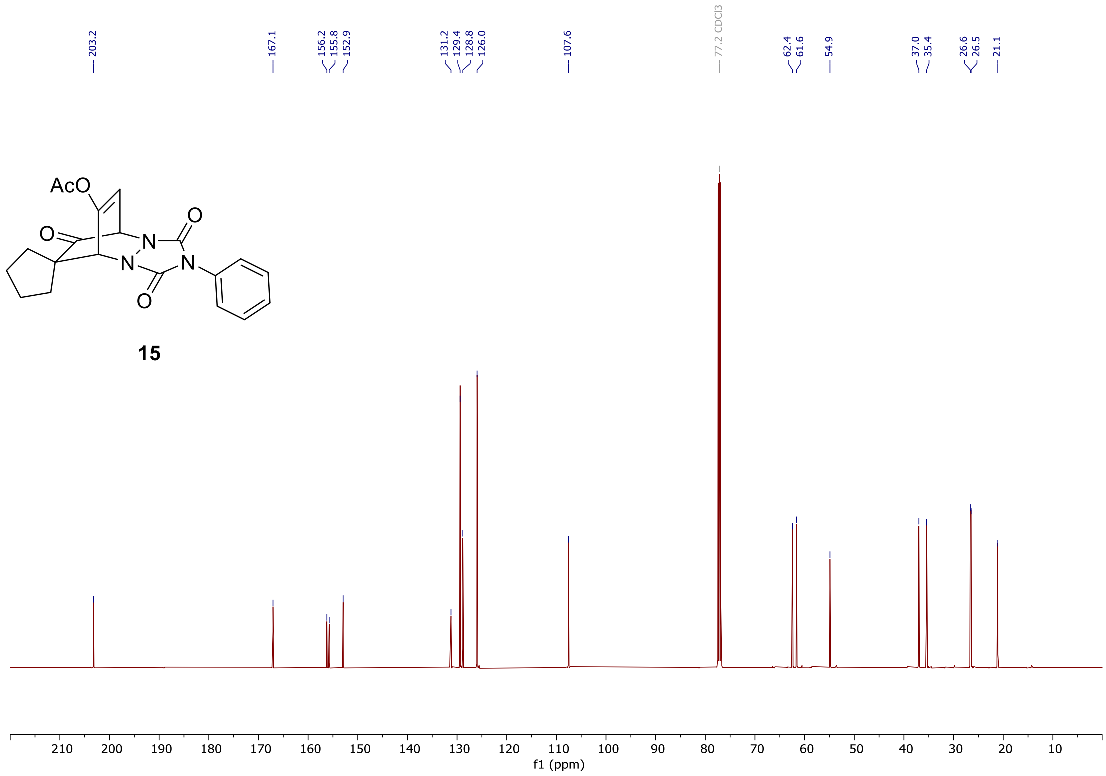

9.4.35. ^{1}H NMR Spectrum of Compound S23 (500 MHz, $CDCl_3$)

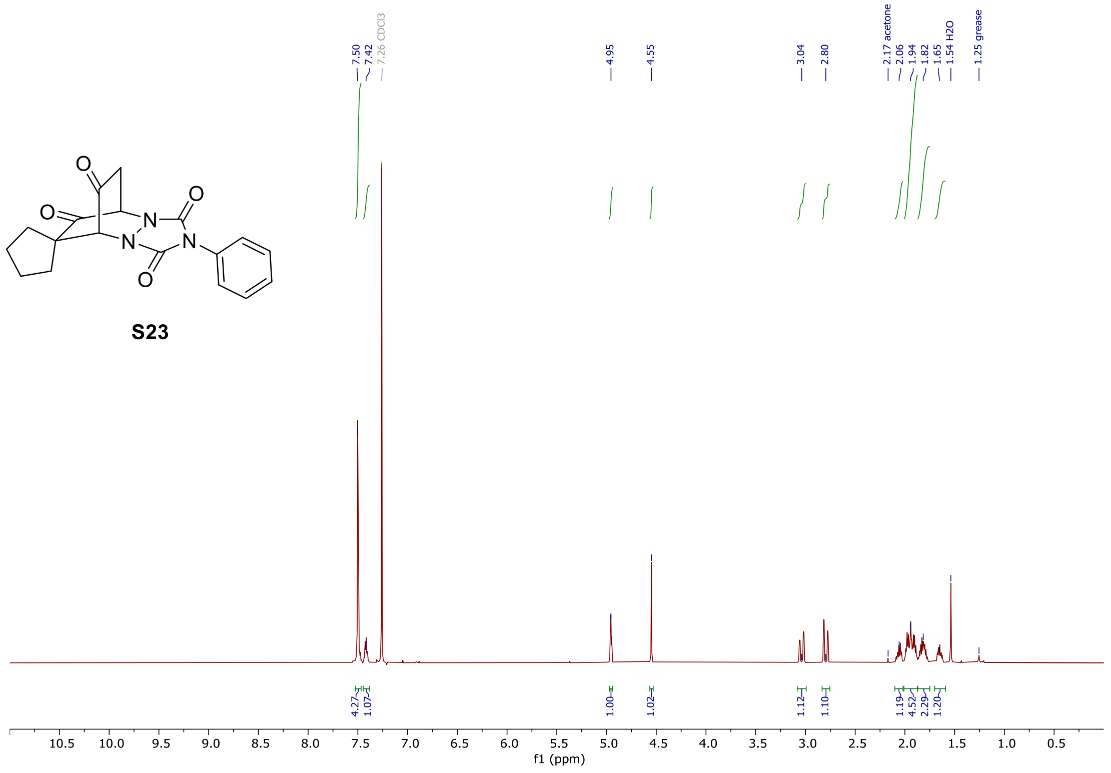

9.4.36. ^{13}C NMR Spectrum of Compound S23 (126 MHz, $CDCl_3$)

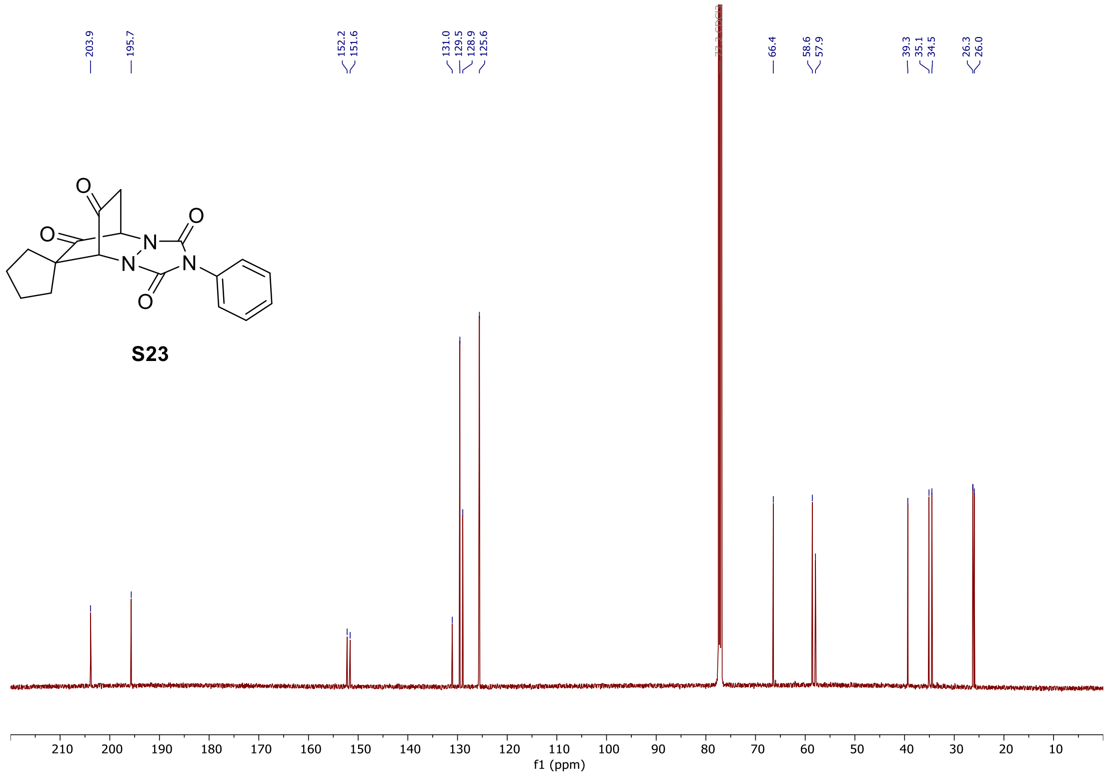

10. X-Ray Data

10.1. X-Ray Crystal Structure of 2a: CCDC Number 2425361:

Table S1: Crystal data and structure refinement for **2a**.

**Compound**	**AL22007**
Formula	$C_{10}H_{12}O_2$
$D_{calc.}$/ g cm^{-3}	1.335
μ/mm^{-1}	0.092
Formula Weight	164.206
Colour	colourless
Shape	block-shaped
Size/mm^3	0.51×0.34×0.11
T/K	100.1(8)
Crystal System	triclinic
Space Group	P-1
a/Å	5.6822(2)
b/Å	6.2486(3)
c/Å	12.6720(6)
α/°	76.774(4)
β/°	83.921(4)
γ/°	68.919(4)
V/Å^3	408.54(3)
Z	2
Z'	1
Wavelength/Å	0.71073
Radiation type	Mo K_{α}
Θ_{min}/°	3.57
Θ_{max}/°	37.72
Measured Refl's.	24230
Indep't Refl's	4180
Refl's I≥2 σ(I)	3722
$R_{\rm int}$	0.0335
Parameters	217
Restraints	0
Largest Peak	0.3270
Deepest Hole	-0.2081
GooF	1.1818
wR_2 (all data)	0.0573
wR_2	0.0551
R_1 (all data)	0.0368
R_1	0.0298

Experimental. Single colourless block-shaped crystals of **AL22007** recrystallised from TBME by slow evaporation. A suitable crystal with dimensions 0.51 × 0.34 × 0.11 mm^3 was selected and mounted on a MITIGEN holder in Paratone oil. on a Rigaku Oxford Diffraction XCalibur diffractometer. The crystal was kept at a steady T = 100.1(8) K during data collection. The structure was solved with the **ShelXS** (Sheldrick, 2008) solution program using direct methods and by using **Olex2** 1.5-beta (Dolomanov et al., 2009) as the graphical interface. The model was refined with **olex2.refine** 1.5-beta (Bourhis et al., 2015) using full matrix least squares minimisation on F^2.

Crystal Data. $C_{10}H_{12}O_2$, M_r = 164.206, triclinic, *P*-1 (No. 2), a = 5.6822(2) Å, b = 6.2486(3) Å, c = 12.6720(6) Å, α = 76.774(4)°, β = 83.921(4)°, γ = 68.919(4)°, *V* = 408.54(3) Å^3, *T* = 100.1(8) K, *Z* = 2, *Z'* = 1, μ(Mo K$_\alpha$) = 0.092, 24230 reflections measured, 4180 unique (R_{int} = 0.0335) which were used in all calculations. The final wR_2 was 0.0573 (all data) and R_1 was 0.0298 (I≥2 σ(I)).

Structure Quality Indicators

**Reflections:**	d min (Mo) 2Θ=75.4° 0.58	I/σ(I) 40.2	Rint 3.35%	Full 50.5° 96% to 75.4° 99.5
**Refinement:**	Shift 0.000	Max Peak 0.3	Min Peak -0.2	GooF 1.182

A colourless block-shaped-shaped crystal with dimensions 0.51 × 0.34 × 0.11 mm^3 was mounted on a MITIGEN holder in Paratone oil.. Data were collected using a Rigaku Oxford Diffraction XCalibur diffractometer equipped with an Oxford Cryosystems Cryostream 700+ low-temperature device operating at *T* = 100.1(8) K.

Data were measured using ω scans with Mo K$_\alpha$ radiation. The diffraction pattern was indexed and the total number of runs and images was based on the strategy calculation from the program CrysAlisPro 1.171.41.123a (Rigaku OD, 2022). The maximum resolution that was achieved was Θ = 37.72° (0.58 Å).

The unit cell was refined using CrysAlisPro 1.171.41.123a (Rigaku OD, 2022) on 10292 reflections, 42% of the observed reflections.

Data reduction, scaling and absorption corrections were performed using CrysAlisPro 1.171.41.123a (Rigaku OD, 2022). The final completeness is 99.53 % out to 37.72° in Θ. A multi-scan absorption correction was performed using CrysAlisPro 1.171.41.123a (Rigaku Oxford Diffraction, 2022) Spherical absorption correction using equivalent radius and absorption coefficient. Empirical absorption correction using spherical harmonics, implemented in SCALE3 ABSPACK scaling algorithm.. The absorption coefficient μ of this material is 0.092 mm^{-1} at this wavelength (λ = 0.71073Å) and the minimum and maximum transmissions are 0.983 and 0.983.

The structure was solved and the space group *P*-1 (# 2) determined by the ShelXS (Sheldrick, 2008) structure solution program using using direct methods and refined by full matrix least squares minimisation on $\boldsymbol{F}^2$ using version of **olex2.refine** 1.5-beta (Bourhis et al., 2015). All non-hydrogen atoms were refined anisotropically. Hydrogen atom positions were calculated geometrically and refined using the riding model.

_refine_special_details: Refinement using NoSpherA2, an implementation ofNOn-SPHERical Atom-form-factors in Olex2.Please cite:F. Kleemiss et al. Chem. Sci. DOI 10.1039/D0SC05526C - 2021NoSpherA2 implementation of HAR makes use oftailor-made aspherical atomic form factors calculatedon-the-fly from a Hirshfeld-partitioned electron density (ED) - not fromspherical-atom form factors.The ED is calculated from a gaussian basis set single determinant SCFwavefunction - either Hartree-Fock or DFT using selected funtionals - for a fragment of the crystal.This fragment can be embedded in an electrostatic crystal field by employing cluster chargesor modelled using implicit solvation models, depending on the software used.The following options were used: SOFTWARE: ORCA PARTITIONING: NoSpherA2 INT ACCURACY: Normal METHOD: PBE BASIS SET: cc-pVTZ CHARGE: 0 MULTIPLICITY: 1 DATE: 2022-09-01_11-24-20

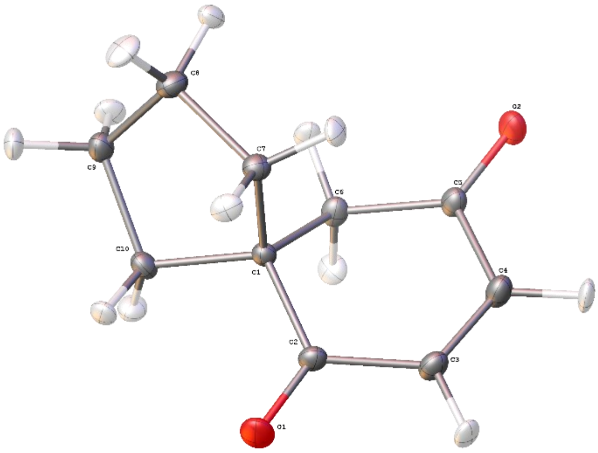

Figure S1: Illustration of structure **2a**. Displacement ellipsoids are at the 50% probability level.

Data Plots: Diffraction Data

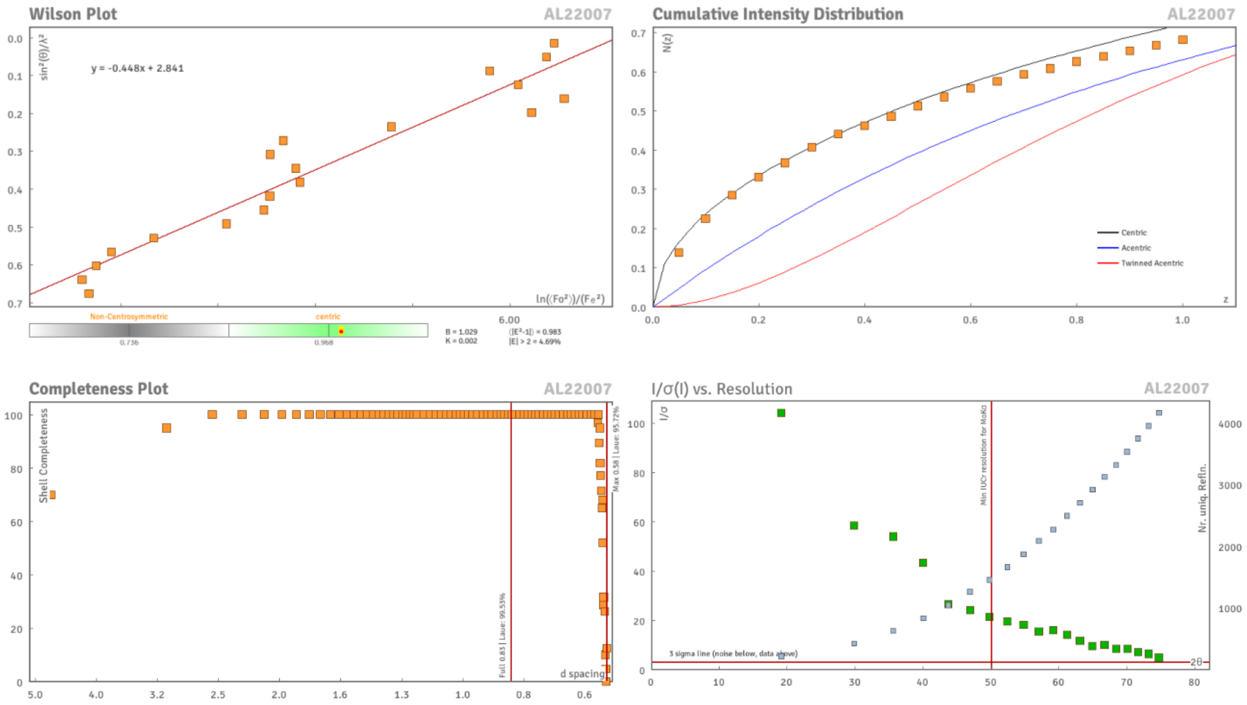

Data Plots: Refinement and Data

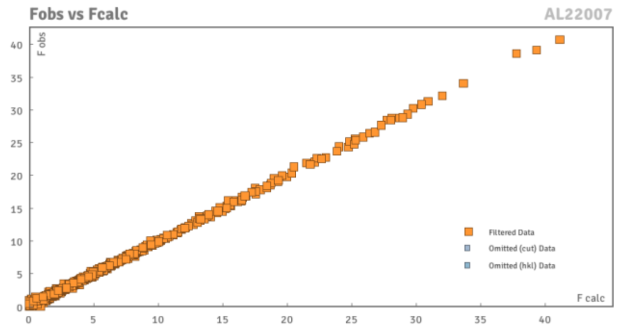

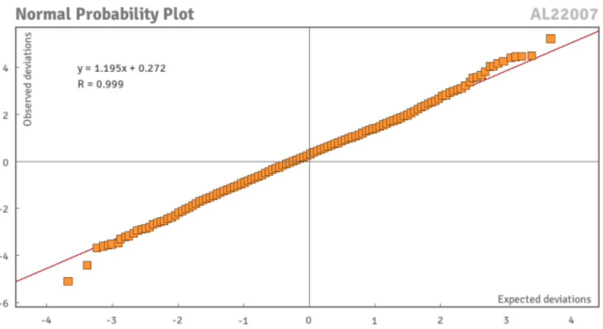

Reflection Statistics

Total reflections (after filtering)	24230	Unique reflections	4180
Completeness	0.957	Mean I/σ	24.84
hkl_{max} collected	(9, 10, 21)	hkl_{min} collected	(-9, -10, -21)
hkl_{max} used	(9, 10, 21)	hkl_{min} used	(-9, -10, 0)
Lim d_{max} collected	100.0	Lim d_{min} collected	0.36
d_{max} used	5.71	d_{min} used	0.58
Friedel pairs	3792	Friedel pairs merged	1
Inconsistent equivalents	0	R_{int}	0.0335
R_{sigma}	0.0249	Intensity transformed	0
Omitted reflections	0	Omitted by user (OMIT hkl)	0
Multiplicity	(1683, 2654, 1213, 526, 766, 604, 279, 171, 48, 21, 5, 2)	Maximum multiplicity	15
Removed systematic absences	0	Filtered off (Shel/OMIT)	0

Table 1: Fractional Atomic Coordinates (×10[4]) and Equivalent Isotropic Displacement Parameters (Å[2]×10[3]) for **AL22007**. U_{eq} is defined as 1/3 of the trace of the orthogonalised U_{ij}.

Atom	x	y	z	U_{eq}
O1	2117.0(6)	6749.4(6)	2354.5(3)	22.73(7)
O2	11083.7(6)	2041.8(5)	4174.0(3)	19.76(7)
C1	6060.6(6)	3557.3(6)	2316.4(3)	10.20(6)
C2	4231.0(7)	5688.7(6)	2708.9(3)	12.75(7)
C3	5157.1(8)	6567.5(7)	3513.4(3)	15.05(7)
C4	7379.2(8)	5374.9(7)	4000.3(3)	16.58(8)
C5	8944.4(7)	2998.7(7)	3829.3(3)	13.84(7)
C6	7703.2(7)	1833.8(6)	3256.7(3)	13.24(7)
C7	7744.5(7)	4347.1(7)	1373.8(3)	12.73(7)
C8	8789.4(8)	2267.2(7)	791.3(4)	15.71(7)
C9	6902.7(7)	933.0(7)	1068.9(4)	15.43(7)
C10	4781.0(7)	2353.6(7)	1757.2(3)	13.18(7)

Table 2: Anisotropic Displacement Parameters (×10[4]) for **AL22007.** The anisotropic displacement factor exponent takes the form: $-2\pi^2[h^2a^{*2} \times U_{11}+ ... +2hka^* \times b^* \times U_{12}]$

Atom	U_{11}	U_{22}	U_{33}	U_{23}	U_{13}	U_{12}
O1	13.34(13)	19.49(14)	31.38(18)	4.81(11)	-7.44(12)	-11.63(13)
O2	16.31(14)	19.90(14)	21.19(16)	0.55(11)	-8.41(11)	-8.19(12)

Atom	U_{11}	U_{22}	U_{33}	U_{23}	U_{13}	U_{12}
C1	8.90(14)	9.37(13)	11.67(16)	-1.83(11)	-1.35(11)	-2.56(11)
C2	10.85(15)	10.91(14)	14.37(17)	-0.53(12)	-0.87(12)	-3.54(12)
C3	16.06(17)	12.17(15)	15.20(17)	-0.90(13)	-1.09(13)	-5.73(13)
H3	35(4)	23(3)	40(4)	1(3)	-6(3)	-20(3)
C4	18.25(18)	15.07(16)	16.02(18)	-1.95(14)	-3.61(14)	-7.29(14)
H4	44(4)	37(4)	34(4)	-8(3)	-15(3)	-22(3)
C5	14.17(16)	13.31(15)	12.72(16)	-1.36(12)	-3.61(13)	-4.21(12)
C6	14.76(16)	9.82(14)	13.48(17)	-1.51(12)	-3.68(13)	-2.16(12)
H6a	27(3)	23(3)	32(4)	5(3)	-8(3)	-11(3)
H6b	36(4)	30(3)	20(3)	-11(3)	-5(3)	5(3)
C7	12.40(15)	11.98(14)	13.90(17)	-4.49(12)	0.68(13)	-2.88(12)
H7a	33(3)	32(4)	36(4)	-21(3)	-1(3)	-12(3)
H7b	34(4)	17(3)	26(3)	-3(3)	-1(3)	4(3)
C8	14.73(17)	16.09(16)	15.86(19)	-3.87(13)	2.44(14)	-6.16(14)
H8a	55(4)	41(4)	21(4)	-21(4)	15(3)	-7(3)
H8b	20(3)	30(4)	47(4)	-6(3)	-1(3)	-17(3)
C9	13.72(16)	14.52(16)	19.36(19)	-3.22(13)	-2.93(14)	-7.65(14)
H9a	27(3)	20(3)	48(4)	0(3)	-3(3)	-11(3)
H9b	37(4)	49(4)	40(4)	-16(3)	-8(3)	-24(3)
C10	10.22(15)	13.13(15)	17.15(18)	-4.24(12)	-2.06(13)	-3.85(13)
H10a	31(4)	37(4)	33(4)	-18(3)	8(3)	-11(3)
H10b	18(3)	27(3)	34(4)	1(3)	-8(3)	-8(3)

Table 3: Bond Lengths in Å for **AL22007**.

Atom	Atom	Length/Å
O1	C2	1.2203(5)
O2	C5	1.2231(5)
C1	C2	1.5164(5)
C1	C6	1.5414(5)
C1	C7	1.5536(5)
C1	C10	1.5307(5)
C2	C3	1.4817(6)
C3	C4	1.3446(6)
C4	C5	1.4829(5)
C5	C6	1.5077(5)
C7	C8	1.5419(6)
C8	C9	1.5482(6)
C9	C10	1.5374(6)

Table 4: Bond Angles in ° for **AL22007**.

Atom	Atom	Atom	Angle/°
C6	C1	C2	110.12(3)
C7	C1	C2	109.43(3)
C7	C1	C6	110.41(3)
C10	C1	C2	113.54(3)
C10	C1	C6	111.79(3)
C10	C1	C7	101.21(3)
C1	C2	O1	123.07(3)
C3	C2	O1	119.64(3)
C3	C2	C1	117.19(3)

Atom	Atom	Atom	Angle/˚
C4	C3	C2	122.38(3)
C5	C4	C3	121.42(4)
C4	C5	O2	120.79(3)
C6	C5	O2	123.02(3)
C6	C5	C4	116.14(3)
C5	C6	C1	112.53(3)
C8	C7	C1	104.22(3)
C9	C8	C7	105.86(3)
C10	C9	C8	105.59(3)
C9	C10	C1	104.41(3)

Table 5: Torsion Angles in ˚ for **AL22007**.

Atom	Atom	Atom	Atom	Angle/˚
O1	C2	C1	C6	-146.47(4)
O1	C2	C1	C7	92.00(4)
O1	C2	C1	C10	-20.25(4)
O1	C2	C3	C4	174.33(4)
O2	C5	C4	C3	171.56(4)
O2	C5	C6	C1	-142.58(4)
C1	C2	C3	C4	-9.20(4)
C1	C6	C5	C4	39.93(4)
C1	C7	C8	C9	23.47(3)
C1	C10	C9	C8	-28.01(3)
C2	C3	C4	C5	-5.32(5)
C3	C4	C5	C6	-10.88(5)
C7	C8	C9	C10	2.50(4)

Table 6: Hydrogen Fractional Atomic Coordinates (×10[4]) and Equivalent Isotropic Displacement Parameters (Å^2×10^3) for **AL22007**. U_{eq} is defined as 1/3 of the trace of the orthogonalised U_{ij}.

Atom	x	y	z	U_{eq}
H3	3968(11)	8253(10)	3677(5)	33.0(16)
H4	8066(12)	6087(11)	4550(5)	36.4(17)
H6a	9132(10)	344(10)	3006(5)	29.9(15)
H6b	6530(11)	1162(11)	3868(5)	30.2(14)
H7a	9197(11)	4832(11)	1650(5)	29.6(15)
H7b	6532(11)	5904(10)	836(5)	28.9(14)
H8a	8982(12)	2881(11)	-86(5)	38.5(17)
H8b	10626(10)	1101(11)	1085(5)	31.4(15)
H9a	7822(11)	-834(10)	1531(5)	33.3(15)
H9b	6167(11)	780(12)	329(5)	38.3(17)
H10a	3845(11)	1318(11)	2316(5)	32.2(15)
H10b	3335(10)	3730(10)	1241(5)	28.2(14)

Citations

CrysAlisPro (Rigaku, V1.171.41.123a, 2022)

CrysAlisPro (ROD), Rigaku Oxford Diffraction, Poland (?).

L.J. Bourhis and O.V. Dolomanov and R.J. Gildea and J.A.K. Howard and H. Puschmann, The Anatomy of a Comprehensive Constrained, Restrained, Refinement Program for the Modern Computing Environment - Olex2 Disected, *Acta Cryst. A*, (2015), **A71**, 59-71.

O.V. Dolomanov and L.J. Bourhis and R.J. Gildea and J.A.K. Howard and H. Puschmann, Olex2: A complete structure solution, refinement and analysis program, *J. Appl. Cryst.*, (2009), **42**, 339-341.

Sheldrick, G.M., A short history of ShelX, *Acta Cryst.*, (2008), **A64**, 339-341.

```
#==============================================================================
# PLATON/CHECK-(181221) versus check.def version 211218, Entry: AL22007
# Data: AL22007.cif - Type: CIF                 Bond Precision   C-C = 0.0006 A
# Refl: AL22007.fcf - Type: LIST4                                  Temp = 100 K
# Audit:OLEX2 1.5-BETA (COMPILED 2022.04.07 SVN.RCA3783A0 FOR OLEXSYS, GUI SVN.R
# Refin:OLEX2.REFINE 1.5-BETA (BOURHIS ET AL., 2015)
# X-ray MoKa              R(int) = 0.034,  wR2/R(int) =  1.7,  Nref/Npar =  19.3
# Cell   5.6822(2)   6.2486(3)  12.6720(6)    76.774(4)    83.921(4)   68.919(4)
# Wavelength 0.71073  Volume Reported       408.54(3) Calculated       408.54(3)
# SpaceGroup from Symmetry P -1         Hall: -P 1                     triclinic
#                 Reported P -1               -P 1                     triclinic
# MoietyFormula C10 H12 O2
#      Reported C10 H12 O2
#    SumFormula C10 H12 O2
#      Reported C10 H12 O2
# Mr        =    164.20[Calc],    164.21[Rep]       Volume/NonHatoms = 17 Ang**3
# Dx,gcm-3  =     1.335[Calc],     1.335[Rep]
# Z         =         2[Calc],         2[Rep]
# Mu (mm-1) =     0.092[Calc],     0.092[Rep]  Xtal Size =  0.110x0.340x0.510 mm
# F000      =     176.0[Calc],     176.1[Rep]  or F000' =    176.09[Calc]
# Reported   T Limits: Tmin=0.983              Tmax=0.983  AbsCorr = MULTI-SCAN
# Calculated T Limits: Tmin=0.963 Tmin'=0.954  Tmax=0.990
# Measured   HKL: Reported  24230, CIF-loop  24230, <Mult>  5.8
# Reported   Hmax=  9, Kmax= 10, Lmax= 21, Nref=   4180        , Th(max)= 37.720
# Obs in FCF Hmax=  9, Kmax= 10, Lmax= 21, Nref=   4180[  4180], Th(max)= 37.718
# Calculated Hmax=  9, Kmax= 10, Lmax= 21, Nref=   4369        , Ratio  =  0.957
# Reported   Rho(min) = -0.21, Rho(max) =  0.33 e/Ang**3 (From CIF)
# Calculated Rho(min) = -0.27, Rho(max) =  0.66 e/Ang**3 (From CIF+FCF data)
#                  w=1/[<sup>2</sup>(Fo<sup>2</sup>)+(0.0196P)<sup>2</sup>+0.0072P],
P=(Fo<sup>2</sup>+2Fc<sup>2</sup>)/3
# R= 0.0510(  3721), wR2= 0.1237(  4180), S = 2.550      (From CIF+FCF data)
# R= 0.0298(  3721), wR2= 0.0573(  4180), S = 1.182      (From FCF data only)
# R= 0.0298(  3722), wR2= 0.0573(  4180), S = 1.182, Npar=  217
#==============================================================================
# For Documentation:http://www.platonsoft.nl/CIF-VALIDATION.pdf
#==============================================================================
*
#==============================================================================
#>>> The Following Improvement and Query ALERTS were generated - (Acta-Mode) <<<
#==============================================================================
Format: alert-number_ALERT_alert-type_alert-level text

906_ALERT_3_C Large K Value in the Analysis of Variance ......      3.719 Check
911_ALERT_3_C Missing FCF Refl Between Thmin & STh/L=    0.600          5 Report
#==============================================================================
154_ALERT_1_G The s.u.'s on the Cell Angles are Equal ..(Note)      0.004 Degree
802_ALERT_4_G CIF Input Record(s) with more than 80 Characters          1 Info
910_ALERT_3_G Missing # of FCF Reflection(s) Below Theta(Min).          2 Note
912_ALERT_4_G Missing # of FCF Reflections Above STh/L=  0.600        182 Note
913_ALERT_3_G Missing # of Very Strong Reflections in FCF ....          3 Note
978_ALERT_2_G Number C-C Bonds with Positive Residual Density.         10 Info
979_ALERT_1_G NoSpherA2 Scattering Factors Used .............     Please Note
#==============================================================================

ALERT_Level and ALERT_Type Summary
==================================
2 ALERT_Level_C = Check. Ensure it is Not caused by an Omission or Oversight
7 ALERT_Level_G = General Info/Check that it is not Something Unexpected

2 ALERT_Type_1 CIF Construction/Syntax Error, Inconsistent or Missing Data.
1 ALERT_Type_2 Indicator that the Structure Model may be Wrong or Deficient.
4 ALERT_Type_3 Indicator that the Structure Quality may be Low.
2 ALERT_Type_4 Improvement, Methodology, Query or Suggestion.
#==============================================================================
```

```
0 Missing  Experimental  Info Issue(s) (Out of  64 Tests) -  100 % Satisfied
0 Experimental  Data  Related Issue(s) (Out of  35 Tests) -  100 % Satisfied
2 Structural  Model   Related Issue(s) (Out of 136 Tests) -   99 % Satisfied
7 Unresolved or to be Checked Issue(s) (Out of 272 Tests) -   97 % Satisfied

*
```

10.2. X-Ray Crystal Structure of 6: CCDC Number 2425362:

Table S2: Crystal data and structure refinement for **6**.

**Compound**	**AL23007**
Formula	$C_{10}H_{16}O_2$
$D_{calc.}$/ g cm^{-3}	1.238
μ/mm^{-1}	0.673
Formula Weight	168.23
Colour	clear colourless
Shape	slab-shaped
Size/mm^3	0.48×0.24×0.06
T/K	120.00(10)
Crystal System	orthorhombic
Space Group	*Pbca*
a/Å	9.32601(18)
b/Å	24.9845(5)
c/Å	15.4931(5)
α/°	90
β/°	90
γ/°	90
V/Å^3	3609.98(15)
Z	16
Z'	2
Wavelength/Å	1.54184
Radiation type	Cu K_α
Θ_{min}/°	4.547
Θ_{max}/°	75.900
Measured Refl's.	43755
Indep't Refl's	3747
Refl's I≥2 σ(I)	3554
R_{int}	0.0495
Parameters	343
Restraints	444
Largest Peak	0.268
Deepest Hole	-0.186
GooF	1.143
wR_2 (all data)	0.1508
wR_2	0.1499
R_1 (all data)	0.0674
R_1	0.0656

Experimental. Single clear colourless slab-shaped crystals of **AL23007** recrystallised from a mixture of hexane and ethyl acetate by slow evaporation. A suitable crystal with dimensions 0.48 × 0.24 × 0.06 mm^3 was selected and mounted on a MITIGEN holder in Paratone oil. on a Rigaku Oxford Diffraction SuperNova diffractometer. The crystal was kept at a steady T = 120.00(10) K during data collection. The structure was solved with the ShelXS (Sheldrick, 2008) solution program using direct methods and by using Olex2 1.5-beta (Dolomanov et al., 2009) as the graphical interface. The model was refined with ShelXL 2018/3 (Sheldrick, 2015) using full matrix least squares minimisation on $\boldsymbol{F}^2$.

Crystal Data. $C_{10}H_{16}O_2$, M_r = 168.23, orthorhombic, *Pbca* (No. 61), a = 9.32601(18) Å, b =

24.9845(5) Å, c = 15.4931(5) Å, $\alpha = \beta = \gamma = 90^{\circ}$, V = 3609.98(15) Å^3, T = 120.00(10) K, Z = 16, Z' = 2, μ(Cu K$_\alpha$) = 0.673, 43755 reflections measured, 3747 unique (R_{int} = 0.0495) which were used in all calculations. The final wR_2 was 0.1508 (all data) and R_1 was 0.0656 (I≥2 σ(I)).

Structure Quality Indicators

**Reflections:**	d min (Cu\a) 2Θ=151.8° 0.79	I/σ(I) 55.6	Rint m=12.51 4.95%	Full 135.4° 99.9
**Refinement:**	Shift 0.000	Max Peak 0.3	Min Peak -0.2	GooF 1.143

A clear colourless slab-shaped-shaped crystal with dimensions 0.48 × 0.24 × 0.06 mm^3 was mounted on a MITIGEN holder in Paratone oil.. Data were collected using a Rigaku Oxford Diffraction SuperNova diffractometer equipped with an Oxford Cryosystems Cryostream 700+ low-temperature device operating at T = 120.00(10) K.

Data were measured using ω scans with Cu K$_\alpha$ radiation. The diffraction pattern was indexed and the total number of runs and images was based on the strategy calculation from the program CrysAlisPro 1.171.42.81a (Rigaku OD, 2023). The maximum resolution that was achieved was Θ= 75.900° (0.79 Å).

The unit cell was refined using CrysAlisPro 1.171.42.81a (Rigaku OD, 2023) on 25705 reflections, 59% of the observed reflections.

Data reduction, scaling and absorption corrections were performed using CrysAlisPro 1.171.42.81a (Rigaku OD, 2023). The final completeness is 99.90 % out to 75.900° in Θ. A gaussian absorption correction was performed using CrysAlisPro 1.171.42.81a (Rigaku Oxford Diffraction, 2023) Numerical absorption correction based on gaussian integration over a multifaceted crystal model Empirical absorption correction using spherical harmonics, implemented in SCALE3 ABSPACK scaling algorithm.. The absorption coefficient μ of this material is 0.673 mm^{-1} at this wavelength (λ = 1.54184Å) and the minimum and maximum transmissions are 0.552 and 1.000.

The structure was solved and the space group *Pbca* (# 61) determined by the ShelXS (Sheldrick, 2008) structure solution program using using direct methods and refined by full matrix least squares minimisation on $\boldsymbol{F}^2$ using version 2018/3 of ShelXL 2018/3 (Sheldrick, 2015). All non-hydrogen atoms were refined anisotropically. Hydrogen atom positions were calculated geometrically and refined using the riding model. Most hydrogen atom positions were calculated geometrically and refined using the riding model, but some hydrogen atoms were refined freely.

_refine_special_details: There are two crystallographically unique molecules in the asymmetric unit. One of these (atoms O21 to C30) was modelled as completely disordered, consistent with elongated ellipsoids and peaks corresponding to an alternative position of C28, C39 and C30. The geometry of both components was restrained to be similar to that of the the ordered molecule.

The value of Z' is 2. This means that there are two independent molecules in the asymmetric unit. The moiety formula is 1(C10 H16 O2).

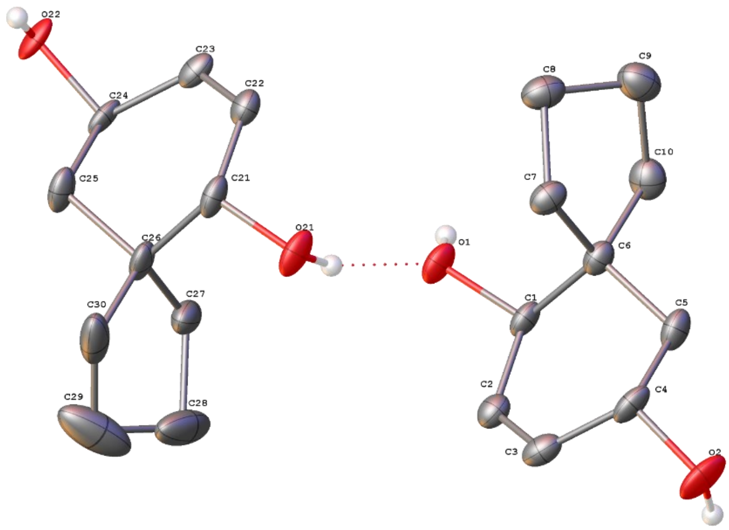

Figure S2. The asymmetric unit of **6**. Displacement ellipsoids are at the 50% probability level. Disorder and C-bound H atoms are not shown.

Data Plots: Diffraction Data

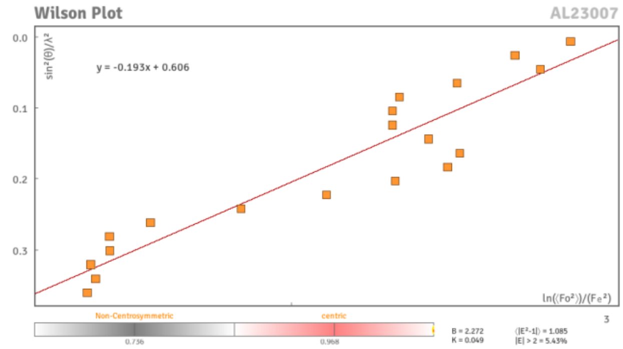

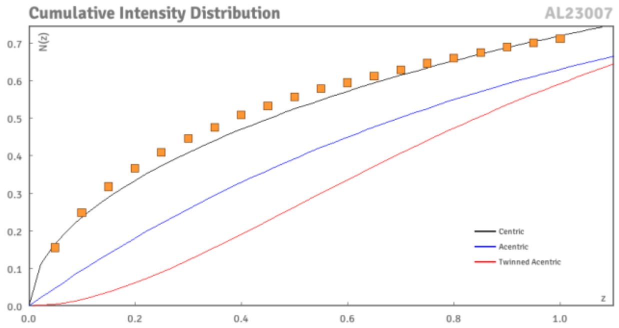

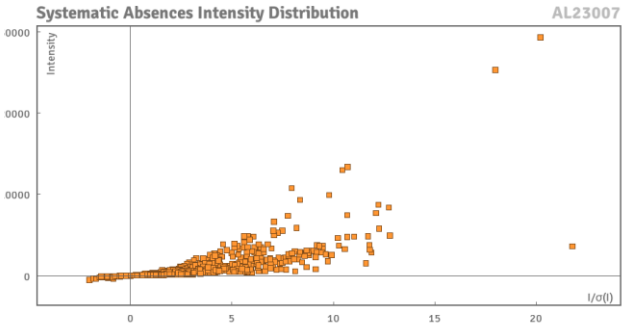

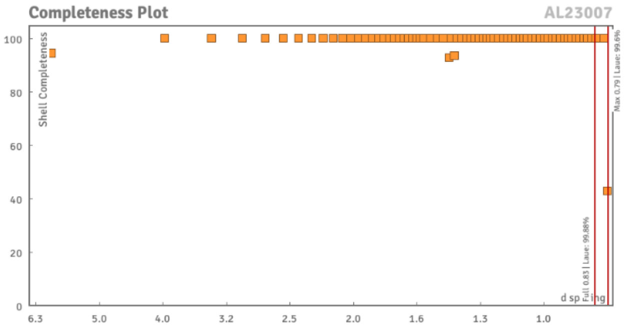

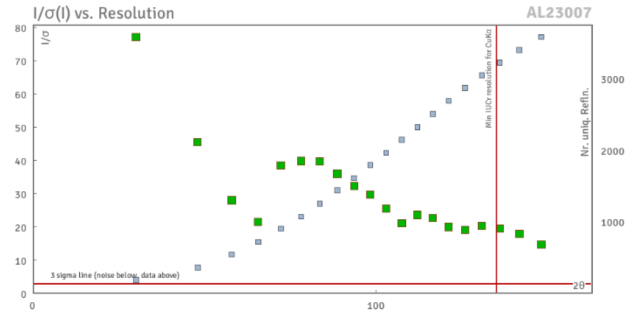

Data Plots: Refinement and Data

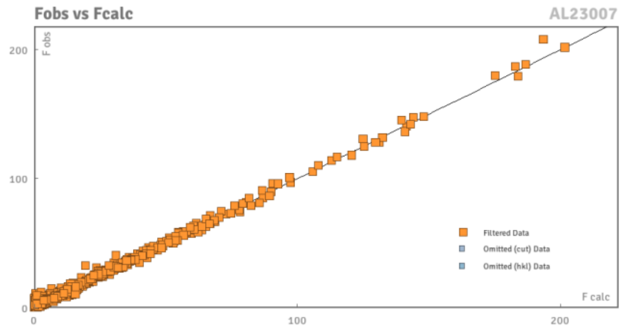

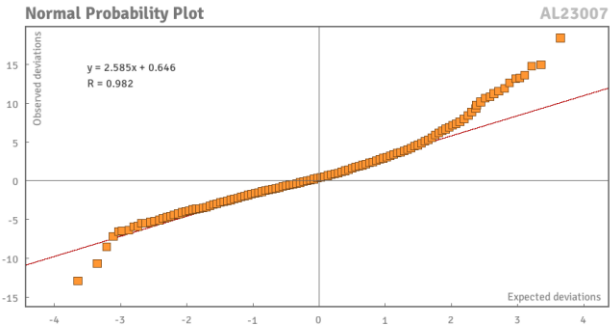

Reflection Statistics

Total reflections (after filtering)	46888	Unique reflections	3747
Completeness	0.996	Mean I/σ	29.63
hkl_{max} collected	(11, 31, 18)	hkl_{min} collected	(-8, -31, -19)
hkl_{max} used	(11, 31, 19)	hkl_{min} used	(0, 0, 0)
Lim d_{max} collected	100.0	Lim d_{min} collected	0.77
d_{max} used	9.72	d_{min} used	0.79
Friedel pairs	5372	Friedel pairs merged	1
Inconsistent equivalents	7	R_{int}	0.0495
R_{sigma}	0.018	Intensity transformed	0
Omitted reflections	0	Omitted by user (OMIT hkl)	0
Multiplicity	(8242, 5296, 2500, 1279, 715, 482, 321, 166, 111, 76, 60, 72, 48, 56, 33, 11, 2)	Maximum multiplicity	60
Removed systematic absences	3133	Filtered off (Shel/OMIT)	0

Table 7: Fractional Atomic Coordinates (×10⁴) and Equivalent Isotropic Displacement Parameters (Å²×10³) for **AL23007**. U_{eq} is defined as 1/3 of the trace of the orthogonalised U_{ij}.

Atom	x	y	z	U_{eq}
O1	7440.9(17)	5984.1(5)	7331.8(10)	25.8(4)
O2	9291.1(19)	3887.6(6)	7109.7(13)	36.6(4)
C1	7373(2)	5412.6(7)	7218.0(13)	20.8(4)
C2	8152(2)	5155.5(8)	7957.8(14)	22.9(4)
C3	8977(2)	4725.9(8)	7874.5(15)	25.1(5)
C4	9258(2)	4460.9(7)	7024.3(15)	24.7(5)
C5	8161(2)	4632.1(8)	6357.2(15)	25.4(5)

Atom	x	y	z	U_{eq}
C6	7941(2)	5243.9(8)	6332.1(13)	21.2(4)
C7	9353(2)	5541.2(8)	6091.9(14)	23.8(4)
C8	8917(3)	5972.6(9)	5441.4(15)	30.5(5)
C9	7745(3)	5697.9(10)	4920.8(15)	36.0(6)
C10	6875(2)	5405.3(9)	5604.4(15)	30.0(5)
O21	9867(11)	6419(3)	7874(5)	28.8(14)
O22	7911(15)	8511(4)	8083(9)	24.3(13)
C21	9765(12)	6989(4)	7971(5)	23.7(16)
C22	8940(11)	7240(4)	7255(7)	22.9(16)
C23	8119(13)	7668(5)	7359(8)	22.1(18)
C24	7870(20)	7937(5)	8223(9)	21(2)
C25	9030(20)	7760(6)	8862(11)	27(2)
C26	9193(16)	7140(5)	8867(7)	26.2(18)
C27	7748(16)	6886(7)	9122(7)	32(2)
C28	8026(12)	6491(2)	9870(4)	66(2)
C29	9412(12)	6738(5)	10277(5)	75(3)
C30	10260(9)	6975(3)	9577(6)	45.8(18)
O21A	9843(13)	6394(4)	8199(6)	30.2(18)
O22A	8090(20)	8508(5)	7960(13)	50(5)
C21A	9827(15)	6974(5)	8247(7)	23.3(18)
C22A	9294(14)	7195(6)	7409(8)	27(2)
C23A	8481(17)	7622(7)	7341(10)	29(3)
C24A	8010(30)	7941(7)	8091(10)	26(3)
C25A	8800(30)	7787(7)	8913(12)	24(3)
C26A	9019(18)	7183(6)	9026(8)	23(2)
C27A	7589(18)	6861(9)	9144(10)	31(3)
C28A	7650(7)	6726(4)	10100(5)	49(2)
C29A	9188(9)	6518(3)	10223(6)	30.3(17)
C30A	9828(11)	7047(4)	9878(6)	41(2)

Table 8: Anisotropic Displacement Parameters (×10[4]) for **AL23007**. The anisotropic displacement factor exponent takes the form: $-2\pi^2[h^2a^{*2} \times U_{11} + ... +2hka^* \times b^* \times U_{12}]$

Atom	U_{11}	U_{22}	U_{33}	U_{23}	U_{13}	U_{12}
O1	25.4(8)	11.3(6)	40.8(9)	-3.8(6)	1.6(7)	3.2(6)
O2	34.3(9)	10.4(7)	65.0(12)	4.2(7)	10.4(9)	5.2(6)
C1	20.5(10)	10.2(8)	31.7(11)	-1.9(7)	2.7(8)	0.5(7)
C2	22.9(10)	17.6(9)	28.2(11)	-0.1(8)	2.7(8)	-2.6(8)
C3	21.3(10)	19.1(10)	34.9(11)	7.2(8)	0.5(9)	-1.1(8)
C4	21.5(10)	10.2(9)	42.5(13)	1.8(8)	3.7(9)	1.6(7)
C5	24.9(10)	15.7(9)	35.8(12)	-7.3(8)	1.9(9)	0.0(8)
C6	20.4(10)	14.8(9)	28.3(11)	-1.0(8)	-0.6(8)	1.2(7)
C7	21.8(10)	18.6(9)	30.9(11)	2.2(8)	3.4(8)	2.5(8)
C8	32.1(12)	25.4(11)	33.9(12)	6.9(9)	5.4(10)	4.5(9)
C9	43.0(14)	36.0(13)	28.9(12)	1.6(10)	-1.9(10)	8.7(11)
C10	27.0(11)	30.3(11)	32.7(12)	-3.2(9)	-3.9(9)	2.2(9)
O21	23(2)	12.1(16)	51(4)	-8(2)	3(3)	2.9(13)
O22	14(3)	10(2)	49(3)	-0.9(18)	2(2)	-4.5(17)
C21	20(3)	12(2)	39(4)	-8(3)	-1(3)	0.8(17)
C22	23(4)	17(3)	30(3)	-3(2)	0(3)	-3(3)
C23	16(5)	14(3)	36(3)	3(2)	0(3)	-2(2)
C24	19(4)	6(3)	36(4)	-1(2)	8(3)	1(2)
C25	25(5)	15(3)	40(4)	-11(2)	-5(3)	4(3)
C26	34(4)	10(2)	34(4)	-6(2)	-8(3)	1(2)
C27	56(5)	18(5)	24(3)	-4(3)	5(3)	-6(4)

Atom	U_{11}	U_{22}	U_{33}	U_{23}	U_{13}	U_{12}
C28	132(6)	24(3)	40(3)	8(2)	27(4)	18(3)
C29	90(6)	101(8)	32(3)	9(4)	2(3)	58(5)
C30	68(4)	21(2)	48(4)	-12(3)	-29(3)	9(3)
O21A	20(2)	15(2)	56(5)	-9(3)	-4(4)	2.6(16)
O22A	36(7)	16(3)	97(10)	19(4)	5(5)	8(3)
C21A	19(3)	11(2)	40(4)	-4(4)	-7(4)	1(2)
C22A	26(6)	24(4)	32(4)	-6(3)	-1(3)	-6(3)
C23A	27(8)	27(5)	33(4)	4(3)	-10(4)	-9(4)
C24A	15(5)	18(4)	45(6)	10(4)	-1(5)	-3(3)
C25A	25(6)	11(3)	36(4)	-5(3)	1(3)	-2(3)
C26A	26(4)	13(3)	30(4)	-5(3)	-7(3)	1(3)
C27A	31(4)	17(5)	44(5)	2(4)	-2(3)	-13(4)
C28A	37(3)	60(5)	49(4)	18(4)	9(3)	16(3)
C29A	36(3)	15(3)	40(4)	9(2)	-1(3)	3(2)
C30A	59(5)	28(4)	35(4)	3(3)	-23(3)	-13(3)

Table 9: Bond Lengths in Å for **AL23007**.

Atom	Atom	Length/Å
O1	C1	1.440(2)
O2	C4	1.439(2)
C1	C2	1.502(3)
C1	C6	1.530(3)
C2	C3	1.327(3)
C3	C4	1.497(3)
C4	C5	1.515(3)
C5	C6	1.543(3)
C6	C7	1.558(3)
C6	C10	1.556(3)
C7	C8	1.531(3)
C8	C9	1.522(3)
C9	C10	1.521(3)
O21	C21	1.436(11)
O22	C24	1.452(12)
C21	C22	1.488(8)
C21	C26	1.535(9)
C22	C23	1.326(9)
C23	C24	1.515(12)
C24	C25	1.527(17)
C25	C26	1.557(13)
C26	C27	1.541(14)
C26	C30	1.539(14)
C27	C28	1.545(14)
C28	C29	1.565(14)
C29	C30	1.468(11)
O21A	C21A	1.451(13)
O22A	C24A	1.435(16)
C21A	C22A	1.496(9)
C21A	C26A	1.516(10)
C22A	C23A	1.313(11)
C23A	C24A	1.475(14)
C24A	C25A	1.522(17)
C25A	C26A	1.532(15)
C26A	C27A	1.569(15)
C26A	C30A	1.557(13)

Atom	Atom	Length/Å
C27A	C28A	1.520(15)
C28A	C29A	1.538(10)
C29A	C30A	1.545(9)

Table 10: Bond Angles in ° for **AL23007**.

Atom	Atom	Atom	Angle/°
O1	C1	C2	108.01(16)
O1	C1	C6	111.57(16)
C2	C1	C6	113.53(16)
C3	C2	C1	123.5(2)
C2	C3	C4	123.0(2)
O2	C4	C3	111.29(18)
O2	C4	C5	110.99(18)
C3	C4	C5	110.94(17)
C4	C5	C6	112.76(17)
C1	C6	C5	107.24(16)
C1	C6	C7	112.07(16)
C1	C6	C10	110.93(17)
C5	C6	C7	111.44(16)
C5	C6	C10	111.12(17)
C10	C6	C7	104.09(17)
C8	C7	C6	105.55(17)
C9	C8	C7	102.85(18)
C10	C9	C8	103.34(19)
C9	C10	C6	106.77(18)
O21	C21	C22	112.0(7)
O21	C21	C26	111.2(7)
C22	C21	C26	113.0(8)
C23	C22	C21	123.3(9)
C22	C23	C24	123.5(10)
O22	C24	C23	107.6(10)
O22	C24	C25	111.4(13)
C23	C24	C25	109.8(11)
C24	C25	C26	111.2(12)
C21	C26	C25	105.9(9)
C21	C26	C27	115.7(10)
C21	C26	C30	110.8(8)
C27	C26	C25	108.9(12)
C30	C26	C25	109.6(10)
C30	C26	C27	105.8(8)
C26	C27	C28	108.0(10)
C27	C28	C29	100.9(7)
C30	C29	C28	107.8(7)
C29	C30	C26	106.7(8)
O21A	C21A	C22A	109.1(8)
O21A	C21A	C26A	113.0(9)
C22A	C21A	C26A	113.5(9)
C23A	C22A	C21A	124.2(11)
C22A	C23A	C24A	123.1(13)
O22A	C24A	C23A	113.9(14)
O22A	C24A	C25A	109.9(15)
C23A	C24A	C25A	112.2(14)
C24A	C25A	C26A	114.1(14)
C21A	C26A	C25A	108.2(11)

Atom	Atom	Atom	Angle/˚
C21A	C26A	C27A	109.7(12)
C21A	C26A	C30A	111.0(10)
C25A	C26A	C27A	114.1(14)
C25A	C26A	C30A	112.1(11)
C30A	C26A	C27A	101.6(9)
C28A	C27A	C26A	101.3(10)
C27A	C28A	C29A	103.3(9)
C28A	C29A	C30A	91.6(7)
C29A	C30A	C26A	107.1(8)

Table 11: Torsion Angles in ˚ for **AL23007**.

Atom	Atom	Atom	Atom	Angle/˚
O1	C1	C2	C3	-141.2(2)
O1	C1	C6	C5	166.69(16)
O1	C1	C6	C7	44.1(2)
O1	C1	C6	C10	-71.8(2)
O2	C4	C5	C6	172.18(17)
C1	C2	C3	C4	2.0(3)
C1	C6	C7	C8	-103.2(2)
C1	C6	C10	C9	129.89(19)
C2	C1	C6	C5	44.4(2)
C2	C1	C6	C7	-78.3(2)
C2	C1	C6	C10	165.87(17)
C2	C3	C4	O2	-141.4(2)
C2	C3	C4	C5	-17.3(3)
C3	C4	C5	C6	47.9(2)
C4	C5	C6	C1	-61.9(2)
C4	C5	C6	C7	61.1(2)
C4	C5	C6	C10	176.72(18)
C5	C6	C7	C8	136.62(18)
C5	C6	C10	C9	-110.9(2)
C6	C1	C2	C3	-16.9(3)
C6	C7	C8	C9	-36.4(2)
C7	C6	C10	C9	9.2(2)
C7	C8	C9	C10	41.9(2)
C8	C9	C10	C6	-31.7(2)
C10	C6	C7	C8	16.8(2)
O21	C21	C22	C23	146.5(11)
O21	C21	C26	C25	-175.8(11)
O21	C21	C26	C27	-55.1(13)
O21	C21	C26	C30	65.4(12)
O22	C24	C25	C26	-168.2(12)
C21	C22	C23	C24	-3(2)
C21	C26	C27	C28	112.6(10)
C21	C26	C30	C29	-137.0(10)
C22	C21	C26	C25	-48.8(13)
C22	C21	C26	C27	71.9(12)
C22	C21	C26	C30	-167.6(9)
C22	C23	C24	O22	138.8(14)
C22	C23	C24	C25	17(2)
C23	C24	C25	C26	-49.2(18)
C24	C25	C26	C21	65.6(16)
C24	C25	C26	C27	-59.5(15)
C24	C25	C26	C30	-174.8(12)

Atom	Atom	Atom	Atom	Angle/˚
C25	C26	C27	C28	-128.2(11)
C25	C26	C30	C29	106.5(12)
C26	C21	C22	C23	20.0(15)
C26	C27	C28	C29	25.9(12)
C27	C26	C30	C29	-10.8(11)
C27	C28	C29	C30	-33.3(12)
C28	C29	C30	C26	28.0(11)
C30	C26	C27	C28	-10.5(12)
O21A	C21A	C22A	C23A	143.6(15)
O21A	C21A	C26A	C25A	-168.4(13)
O21A	C21A	C26A	C27A	-43.3(14)
O21A	C21A	C26A	C30A	68.1(13)
O22A	C24A	C25A	C26A	-169.3(16)
C21A	C22A	C23A	C24A	1(3)
C21A	C26A	C27A	C28A	131.8(12)
C21A	C26A	C30A	C29A	-94.3(12)
C22A	C21A	C26A	C25A	-43.5(16)
C22A	C21A	C26A	C27A	81.6(14)
C22A	C21A	C26A	C30A	-167.0(11)
C22A	C23A	C24A	O22A	137.1(19)
C22A	C23A	C24A	C25A	11(3)
C23A	C24A	C25A	C26A	-41(2)
C24A	C25A	C26A	C21A	58(2)
C24A	C25A	C26A	C27A	-65(2)
C24A	C25A	C26A	C30A	-179.7(15)
C25A	C26A	C27A	C28A	-106.6(14)
C25A	C26A	C30A	C29A	144.5(13)
C26A	C21A	C22A	C23A	16.6(19)
C26A	C27A	C28A	C29A	-47.1(15)
C27A	C26A	C30A	C29A	22.3(13)
C27A	C28A	C29A	C30A	57.8(12)
C28A	C29A	C30A	C26A	-48.0(10)
C30A	C26A	C27A	C28A	14.3(15)

Table 12: Hydrogen Fractional Atomic Coordinates (×10[4]) and Equivalent Isotropic Displacement Parameters (Å[2]×10[3]) for **AL23007**. U_{eq} is defined as 1/3 of the trace of the orthogonalised U_{ij}.

Atom	x	y	z	U_{eq}
H1	6342.6	5303.39	7253.23	25
H2	8048.36	5308.64	8515.58	27
H3	9409.34	4579.78	8377.26	30
H4	10222.66	4580.01	6818.77	30
H5A	7233.91	4457.01	6487.11	31
H5B	8477.81	4508.38	5780.81	31
H7A	9791.15	5705.25	6610.41	29
H7B	10052.17	5290.37	5831.36	29
H8A	8545.87	6295.31	5737.31	37
H8B	9734.52	6075.46	5069.08	37
H9A	7147.42	5962.9	4610.31	43
H9B	8157.86	5443.81	4498.22	43
H10A	6417.6	5083.1	5354.29	36
H10B	6115.49	5641.6	5836.73	36
H5	6610(40)	6128(12)	7174(19)	45(8)
H6	8440(40)	3795(12)	7380(20)	48(9)
H7	9060(40)	6296(13)	7820(20)	55(10)

Atom	x	y	z	U_{eq}
H8	8840(40)	8575(15)	7960(30)	61(14)
H21	10764.09	7132.42	7933.9	28
H22	9005.95	7085.49	6696.53	27
H23	7660.33	7814.6	6864.39	27
H24	6907.02	7832.78	8448.96	25
H25A	8765.85	7884.45	9448.15	32
H25B	9951.64	7926.55	8703.18	32
H27A	7330.57	6694.05	8622.31	39
H27B	7065.95	7166.78	9306.62	39
H28A	8197.01	6122.98	9655.84	79
H28B	7220.29	6486.9	10286.44	79
H29A	9151.74	7015.25	10705.75	90
H29B	9973.86	6456.21	10573.61	90
H30A	10956.68	6711.62	9349.6	55
H30B	10793.14	7291.44	9789.04	55
H21A	10845.67	7092.77	8310.75	28
H22A	9561.54	7015.22	6893.64	33
H23A	8181.17	7731.09	6782.1	35
H24A	6976.32	7853.8	8188.73	31
H25C	8264.55	7926.01	9415.13	29
H25D	9755.93	7963.19	8910.58	29
H27C	6740.19	7082.43	9004.45	37
H27D	7578.83	6533.47	8784.08	37
H28C	6936.41	6448.1	10250.79	58
H28D	7474.48	7047.88	10457.93	58
H29C	9414.15	6204.04	9856.8	36
H29D	9434.32	6445.51	10833.13	36
H30C	10865.97	7003.81	9764.31	49
H30D	9696.98	7336.7	10305.73	49

Table 13: Hydrogen Bond information for **AL23007**.

D	H	A	d(D-H)/Å	d(H-A)/Å	d(D-A)/Å	D-H-A/deg
O1	H5	O21[1]	0.89(3)	1.78(3)	2.653(9)	166(3)
O1	H5	O21A[1]	0.89(3)	1.87(4)	2.756(11)	177(3)
O2	H6	O22[2]	0.93(3)	1.81(4)	2.716(12)	165(3)
O2	H6	O22A[2]	0.93(3)	1.84(4)	2.754(18)	170(3)
O21	H7	O1	0.82(4)	1.86(4)	2.647(10)	161(4)
O21A	H7	O1	0.97(4)	1.86(4)	2.806(11)	165(3)
O22	H8	O2[3]	0.90(5)	1.91(4)	2.789(14)	164(4)
O22A	H8	O2[3]	0.72(4)	1.91(4)	2.62(2)	169(4)

----
[1]-1/2+x,+y,3/2-z; [2]3/2-x,-1/2+y,+z; [3]2-x,1/2+y,3/2-z

Table 14: Atomic Occupancies for all atoms that are not fully occupied in **AL23007**.

Atom	Occupancy
O21	0.550(11)
O22	0.550(11)

Atom	Occupancy
C21	0.550(11)
H21	0.550(11)
C22	0.550(11)
H22	0.550(11)
C23	0.550(11)
H23	0.550(11)
C24	0.550(11)
H24	0.550(11)
C25	0.550(11)
H25A	0.550(11)
H25B	0.550(11)
C26	0.550(11)
C27	0.550(11)
H27A	0.550(11)
H27B	0.550(11)
C28	0.550(11)
H28A	0.550(11)
H28B	0.550(11)
C29	0.550(11)
H29A	0.550(11)
H29B	0.550(11)
C30	0.550(11)
H30A	0.550(11)
H30B	0.550(11)
O21A	0.450(11)
O22A	0.450(11)
C21A	0.450(11)
H21A	0.450(11)
C22A	0.450(11)
H22A	0.450(11)
C23A	0.450(11)
H23A	0.450(11)
C24A	0.450(11)
H24A	0.450(11)
C25A	0.450(11)
H25C	0.450(11)
H25D	0.450(11)
C26A	0.450(11)
C27A	0.450(11)
H27C	0.450(11)
H27D	0.450(11)
C28A	0.450(11)
H28C	0.450(11)
H28D	0.450(11)
C29A	0.450(11)
H29C	0.450(11)
H29D	0.450(11)
C30A	0.450(11)
H30C	0.450(11)
H30D	0.450(11)

Citations

CrysAlisPro Software System, Rigaku Oxford Diffraction, (2023).

O.V. Dolomanov and L.J. Bourhis and R.J. Gildea and J.A.K. Howard and H. Puschmann, Olex2: A complete structure solution, refinement and analysis program, *J. Appl. Cryst.*, (2009), **42**, 339-341.

Sheldrick, G.M., A short history of ShelX, *Acta Cryst.*, (2008), **A64**, 339-341.

Sheldrick, G.M., Crystal structure refinement with ShelXL, *Acta Cryst.*, (2015), **C71**, 3-8.

```
#===============================================================================
# PLATON/CHECK-(110423) versus check.def version 221222, Entry: al23007
# Data: AL23007.cif - Type: CIF                  Bond Precision   C-C = 0.0031 A
# Refl: AL23007.fcf - Type: LIST4                                   Temp = 120 K
# Audit:OLEX2 1.5-BETA (COMPILED 2023.03.06 SVN.RBB2C1857 FOR OLEXSYS, GUI SVN.R
# Refin:SHELXL 2018/3 (SHELDRICK, 2015)
# X-ray CuKa             R(int) = 0.049,  wR2/R(int) =  3.0,  Nref/Npar =  10.9
# Cell 9.32601(18)  24.9845(5)  15.4931(5)           90          90          90
# Wavelength 1.54184  Volume Reported     3609.98(15) Calculated     3609.98(15)
# SpaceGroup from Symmetry P b c a      Hall: -P 2ac 2ab           orthorhombic
#                 Reported P b c a            -P 2ac 2ab           orthorhombic
# MoietyFormula C10 H16 O2
#      Reported 1(C10 H16 O2)
#    SumFormula C10 H16 O2
#      Reported C10 H16 O2
# Mr        =    168.23[Calc],    168.23[Rep]       Volume/NonHatoms = 19 Ang**3
# Dx,gcm-3  =     1.238[Calc],     1.238[Rep]
# Z         =        16[Calc],        16[Rep]
# Mu (mm-1) =     0.673[Calc],     0.673[Rep]  Xtal Size =  0.064x0.237x0.478 mm
# F000      =    1472.0[Calc],    1472.0[Rep]  or F000' =   1476.31[Calc]
# Reported   T Limits: Tmin=0.552              Tmax=1.000  AbsCorr = GAUSSIAN
# Calculated T Limits: Tmin=0.826 Tmin'=0.725  Tmax=0.958  Exti    =    0.00084
# Measured   HKL: Reported  43755, Embedded  46888, <Mult> 11.6
# Reported   Hmax= 11, Kmax= 31, Lmax= 19, Nref=   3747        , Th(max)= 75.900
# Obs in FCF Hmax= 11, Kmax= 31, Lmax= 19, Nref=   3747[  3747], Th(max)= 75.900
# Calculated Hmax= 11, Kmax= 31, Lmax= 19, Nref=   3762        , Ratio  =  0.996
# Reported   Rho(min) = -0.19, Rho(max) = 0.27 e/Ang**3 (From CIF)
# Calculated Rho(min) = -0.20, Rho(max) = 0.26 e/Ang**3 (From CIF+FCF data)
#                  w=1/[<sup>2</sup>(Fo<sup>2</sup>)+(0.0347P)<sup>2</sup>+5.5017P],
P=(Fo<sup>2</sup>+2Fc<sup>2</sup>)/3
# R= 0.0656(  3553), wR2= 0.1509(  3747), S = 1.144     (From CIF+FCF data)
# R= 0.0656(  3553), wR2= 0.1508(  3747), S = 1.143     (From FCF data only)
# R= 0.0656(  3554), wR2= 0.1508(  3747), S = 1.143, Npar=  343
#===============================================================================
# For Documentation:http://www.platonsoft.nl/CIF-VALIDATION.pdf
#===============================================================================
#                                                                              *
#===============================================================================
#>>> The Following Improvement and Query ALERTS were generated - (Acta-Mode) <<<
#===============================================================================
Format: alert-number_ALERT_alert-type_alert-level text

213_ALERT_2_C Atom O22A            has ADP max/min Ratio .....        3.2 prolat
220_ALERT_2_C NonSolvent   Resd 1  C   Ueq(max)/Ueq(min) Range        3.7 Ratio
250_ALERT_2_C Large U3/U1 Ratio for Average U(i,j) Tensor ....        2.1 Note
906_ALERT_3_C Large K Value in the Analysis of Variance ......      9.044 Check
911_ALERT_3_C Missing FCF Refl Between Thmin & STh/L=    0.600          3 Report
#===============================================================================
002_ALERT_2_G Number of Distance or Angle Restraints on AtSite         24 Note
003_ALERT_2_G Number of Uiso or Uij Restrained non-H Atoms ...         24 Report
083_ALERT_2_G SHELXL Second Parameter in WGHT  Unusually Large       5.50 Why ?
175_ALERT_4_G The CIF-Embedded .res File Contains SAME Records          1 Report
176_ALERT_4_G The CIF-Embedded .res File Contains SADI Records          2 Report
177_ALERT_4_G The CIF-Embedded .res File Contains DELU Records          2 Report
178_ALERT_4_G The CIF-Embedded .res File Contains SIMU Records          2 Report
187_ALERT_4_G The CIF-Embedded .res File Contains RIGU Records          4 Report
189_ALERT_3_G A Non-default SAME Restraint Value for SecondPar     0.0400 Report
301_ALERT_3_G Main Residue  Disorder .............(Resd  1  )       100% Note
414_ALERT_2_G Short Intra D-H..H-X       H7       ..H27D     .       2.12 Ang.
x,y,z  =      1_555 Check
793_ALERT_4_G Model has Chirality at C1          (Centro SPGR)          R Verify
793_ALERT_4_G Model has Chirality at C4          (Centro SPGR)          S Verify
793_ALERT_4_G Model has Chirality at C21         (Centro SPGR)          S Verify
793_ALERT_4_G Model has Chirality at C24         (Centro SPGR)          R Verify
793_ALERT_4_G Model has Chirality at C21A        (Centro SPGR)          S Verify
```

```
793_ALERT_4_G Model has Chirality at C24A        (Centro SPGR)          R Verify
811_ALERT_5_G No ADDSYM Analysis: Too Many Excluded Atoms ....          ! Info
860_ALERT_3_G Number of Least-Squares Restraints ............         444 Note
910_ALERT_3_G Missing # of FCF Reflection(s) Below Theta(Min).          1 Note
912_ALERT_4_G Missing # of FCF Reflections Above STh/L=  0.600         12 Note
978_ALERT_2_G Number C-C Bonds with Positive Residual Density.         13 Info
#==============================================================================

ALERT_Level and ALERT_Type Summary
==================================
5 ALERT_Level_C = Check. Ensure it is Not caused by an Omission or Oversight
22 ALERT_Level_G = General Info/Check that it is not Something Unexpected

8 ALERT_Type_2 Indicator that the Structure Model may be Wrong or Deficient.
6 ALERT_Type_3 Indicator that the Structure Quality may be Low.
12 ALERT_Type_4 Improvement, Methodology, Query or Suggestion.
1 ALERT_Type_5 Informative Message, Check.
#==============================================================================

0 Missing  Experimental  Info Issue(s) (Out of  65 Tests) -  100 % Satisfied
0 Experimental  Data  Related Issue(s) (Out of  35 Tests) -  100 % Satisfied
10 Structural  Model   Related Issue(s) (Out of 144 Tests) -   93 % Satisfied
17 Unresolved or to be Checked Issue(s) (Out of 277 Tests) -   94 % Satisfied

#                                                                             *
```

10.3. X-Ray Crystal Structure of 13: CCDC Number 2425364:

Table S3: Crystal data and structure refinement for **13**

**Compound**	**AL24014**
Formula	$C_{22}H_{21}NO_5$
$D_{calc.}$/ g cm^{-3}	1.411
μ/mm^{-1}	0.825
Formula Weight	379.416
Colour	clear colourless
Shape	block-shaped
Size/mm^3	0.50×0.24×0.20
T/K	120.00(10)
Crystal System	triclinic
Space Group	P-1
a/Å	9.3212(3)
b/Å	10.2824(3)
c/Å	10.6049(3)
α/°	72.894(3)
β/°	81.533(3)
γ/°	66.882(3)
V/Å^3	892.86(6)
Z	2
Z'	1
Wavelength/Å	1.54184
Radiation type	Cu K$_\alpha$
Θ_{min}/°	4.36
Θ_{max}/°	76.03
Measured Refl's.	18731
Indep't Refl's	3684
Refl's I≥2 σ(I)	3608
R_{int}	0.0184
Parameters	442
Restraints	0
Largest Peak	0.0879
Deepest Hole	-0.1118
GooF	1.1809
wR_2 (all data)	0.0291
wR_2	0.0288
R_1 (all data)	0.0129
R_1	0.0123

Experimental. Single clear colourless block-shaped crystals of **AL24014** recrystallised from diethyl ether by slow evaporation. A suitable crystal with dimensions 0.50 × 0.24 × 0.20 mm^3 was selected and mounted on a MITIGEN holder in Paratone oil. on a Rigaku Oxford Diffraction SuperNova diffractometer. The crystal was kept at a steady T = 120.00(10) K during data collection. The structure was solved with the ShelXS (Sheldrick, 2008) solution program using direct methods and by using Olex2 1.5-beta (Dolomanov et al., 2009) as the graphical interface. The model was refined with olex2.refine 1.5-beta (Bourhis et al., 2015) using full matrix least squares minimisation on F^2.

Crystal Data. $C_{22}H_{21}NO_5$, M_r = 379.416, triclinic, P-1 (No. 2), a = 9.3212(3) Å, b = 10.2824(3) Å, c = 10.6049(3) Å, α = 72.894(3)°, β = 81.533(3)°, γ = 66.882(3)°, V = 892.86(6) Å^3, T = 120.00(10) K, Z = 2,

Z' = 1, μ(Cu K$_\alpha$) = 0.825, 18731 reflections measured, 3684 unique (R_{int} = 0.0184) which were used in all calculations. The final wR_2 was 0.0291 (all data) and R_1 was 0.0123 (I≥2 σ(I)).

Structure Quality Indicators

**Reflections:**	d min (CuKα) 2Θ=152.1° 0.79	I/σ(I) 89.0	Rint m=5.08 1.84%	Full 135.4° 99.4
**Refinement:**	Shift -0.001	Max Peak 0.1	Min Peak -0.1	GooF 1.181

A clear colourless block-shaped-shaped crystal with dimensions 0.50 × 0.24 × 0.20 mm^3 was mounted on a MITIGEN holder in Paratone oil.. Data were collected using a Rigaku Oxford Diffraction SuperNova diffractometer equipped with an Oxford Cryosystems Cryostream 700+ low-temperature device operating at T = 120.00(10) K.

Data were measured using ω scans with Cu K$_\alpha$ radiation. The diffraction pattern was indexed and the total number of runs and images was based on the strategy calculation from the program CrysAlisPro 1.171.43.116a (Rigaku OD, 2024). The maximum resolution that was achieved was Θ = 76.03° (0.79 Å).

The unit cell was refined using CrysAlisPro 1.171.43.116a (Rigaku OD, 2024) on 15089 reflections, 81% of the observed reflections.

Data reduction, scaling and absorption corrections were performed using CrysAlisPro 1.171.43.116a (Rigaku OD, 2024). The final completeness is 99.38 % out to 76.03° in Θ. A gaussian absorption correction was performed using CrysAlisPro 1.171.43.116a (Rigaku Oxford Diffraction, 2024) Numerical absorption correction based on gaussian integration over a multifaceted crystal model Empirical absorption correction using spherical harmonics, implemented in SCALE3 ABSPACK scaling algorithm.. The absorption coefficient μ of this material is 0.825 mm^{-1} at this wavelength (λ = 1.54184Å) and the minimum and maximum transmissions are 0.585 and 1.000.

The structure was solved and the space group P-1 (# 2) determined by the ShelXS (Sheldrick, 2008) structure solution program using using direct methods and refined by full matrix least squares minimisation on $\boldsymbol{F}^2$ using version of olex2.refine 1.5-beta (Bourhis et al., 2015). All non-hydrogen atoms were refined anisotropically. Hydrogen atom positions were calculated geometrically and refined using the riding model.

_olex2_refine_details: Refinement using NoSpherA2, an implementation ofNOn-SPHERical Atom-form-factors in Olex2.Please cite:F. Kleemiss et al. Chem. Sci. DOI 10.1039/D0SC05526C - 2021NoSpherA2 implementation of HAR makes use oftailor-made aspherical atomic form factors calculatedon-the-fly from a Hirshfeld-partitioned electron density (ED) - not fromspherical-atom form factors.The ED is calculated from a gaussian basis set single determinant SCFwavefunction - either Hartree-Fock or DFT using selected funtionals - for a fragment of the crystal.This fragment can be embedded in an electrostatic crystal field by employing cluster chargesor modelled using implicit solvation models, depending on the software used.The following options were used: SOFTWARE: ORCA 5.0 PARTITIONING: NoSpherA2 INT ACCURACY: Normal METHOD: R2SCAN BASIS SET: cc-pVTZ CHARGE: 0 MULTIPLICITY: 1 DATE: 2024-09-23_15-02-06

_exptl_absorpt_process_details: CrysAlisPro 1.171.43.116a (Rigaku Oxford Diffraction, 2024)Numerical absorption correction based on gaussian integration overa multifaceted crystal modelEmpirical absorption correction using spherical harmonics,implemented in SCALE3 ABSPACK

scaling algorithm.

There is a single formula unit in the asymmetric unit, which is represented by the reported sum formula. In other words: Z is 2 and Z' is 1. The moiety formula is C22 H21 N O5.

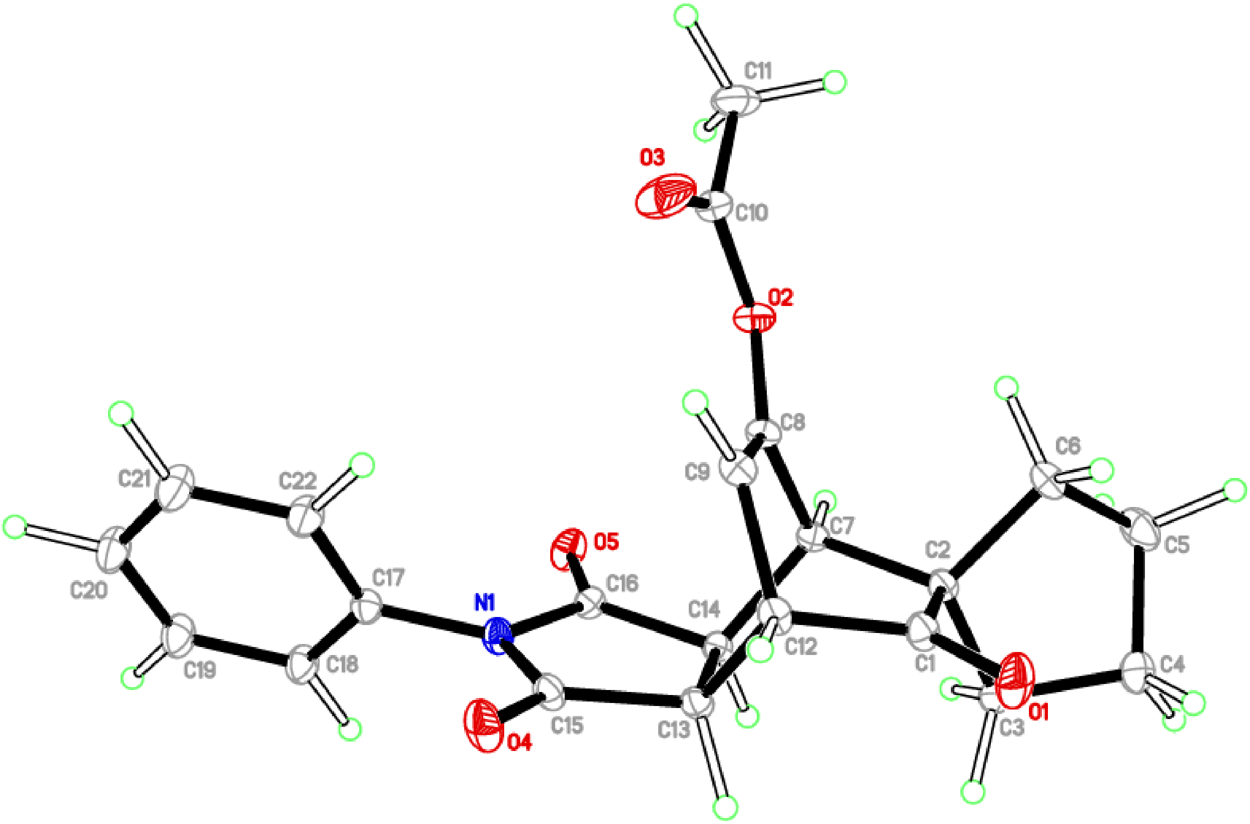

Figure 1 The molecular structure of AL24014. Displacement ellipsoids are at the 50% probability level.

_refine_special_details: NoSpherA2 refinement.

Data Plots: Diffraction Data

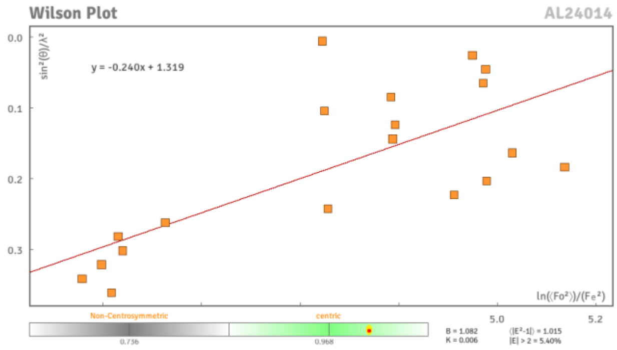

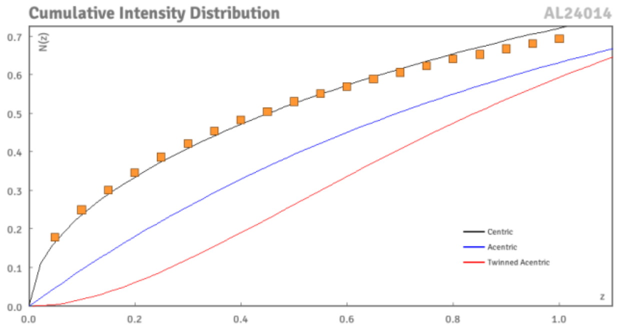

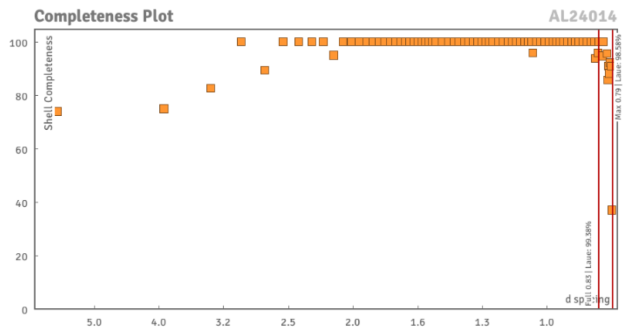

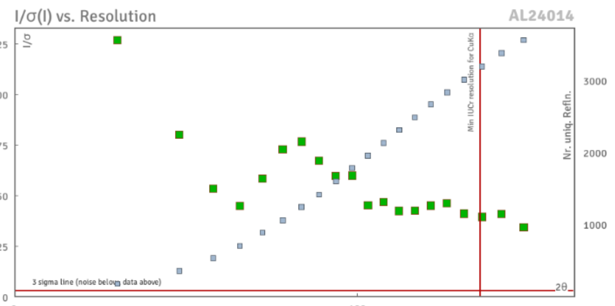

Data Plots: Refinement and Data

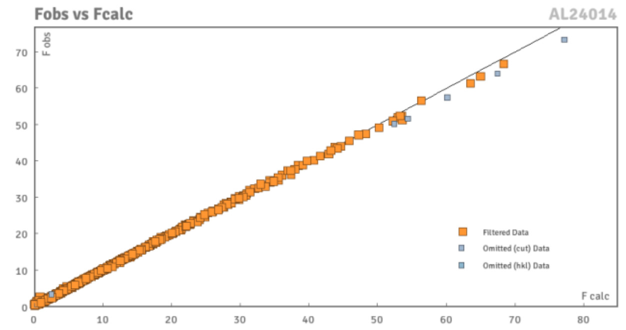

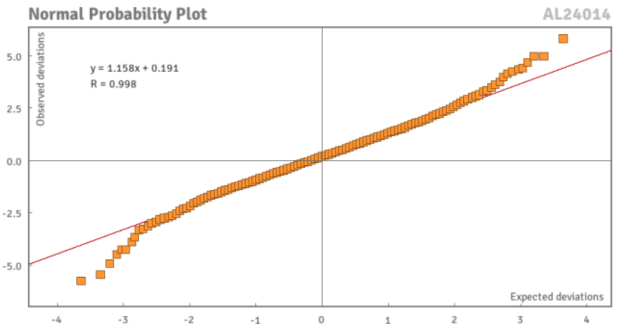

Reflection Statistics

Total reflections (after filtering)	18715	Unique reflections	3684
Completeness	0.986	Mean I/σ	55.94
hkl_{max} collected	(11, 12, 13)	hkl_{min} collected	(-11, -12, -10)
hkl_{max} used	(11, 12, 13)	hkl_{min} used	(-11, -12, 0)
Lim d_{max} collected	100.0	Lim d_{min} collected	0.77
d_{max} used	10.13	d_{min} used	0.79
Friedel pairs	2121	Friedel pairs merged	1
Inconsistent equivalents	10	R_{int}	0.0185
R_{sigma}	0.0112	Intensity transformed	0
Omitted reflections	16	Omitted by user (OMIT hkl)	0
Multiplicity	(1448, 1235, 1109, 755, 490, 284, 196, 97, 64, 43, 23, 22, 20, 9, 6, 7, 2, 0, 1)	Maximum multiplicity	20
Removed systematic absences	0	Filtered off (Shel/OMIT)	0

Table 15: Fractional Atomic Coordinates (×10^4) and Equivalent Isotropic Displacement Parameters (Å^2×10^3) for **AL24014.** U_{eq} is defined as 1/3 of the trace of the orthogonalised U_{ij}.

Atom	x	y	z	U_{eq}
O(1)	2814.1(3)	4933.9(3)	5833.1(2)	20.59(6)
O(2)	805.0(3)	4084.4(3)	2044.9(2)	14.76(5)
O(3)	276.0(4)	2063.1(3)	3135.4(3)	28.28(7)
O(4)	6519.1(3)	486.9(3)	4120.1(2)	19.68(6)
O(5)	4545.4(3)	3621.6(3)	193.5(2)	16.21(6)
N(1)	5788.6(3)	1812.9(3)	1991.2(3)	12.68(6)
C(1)	2915.1(4)	4655.9(4)	4780.0(3)	13.06(7)
C(2)	2325.5(4)	5826.2(4)	3496.9(3)	11.62(7)
C(3)	3119.6(4)	6967.8(4)	3211.3(3)	14.06(7)

Atom	x	y	z	U_{eq}
C(4)	1905.3(4)	8286.3(4)	3646.3(4)	19.96(8)
C(5)	381.2(4)	8340.6(4)	3224.8(4)	20.99(8)
C(6)	547.8(4)	6731.4(4)	3643.9(4)	16.21(7)
C(7)	2681.2(4)	5008.0(3)	2405.6(3)	10.57(6)
C(8)	1957.6(4)	3862.3(4)	2854.4(3)	11.54(6)
C(9)	2487.2(4)	2837.5(4)	3977.9(3)	12.86(7)
C(10)	-32.0(4)	3192.5(4)	2300.4(3)	15.33(7)
C(11)	-1354.1(5)	3855.1(5)	1384.4(4)	22.50(8)
C(12)	3676.1(4)	3104.6(4)	4603.6(3)	12.59(7)
C(13)	5080.6(4)	3126.3(4)	3618.8(3)	11.67(7)
C(14)	4470.1(4)	4196.9(3)	2287.3(3)	10.49(6)
C(15)	5898.2(4)	1637.9(4)	3338.2(3)	12.91(7)
C(16)	4902.0(4)	3242.8(4)	1332.5(3)	11.39(6)
C(17)	6372.6(4)	608.0(4)	1404.0(3)	12.72(7)
C(18)	7533.3(4)	549.9(4)	414.2(3)	15.99(7)
C(19)	8096.1(4)	-635.2(4)	-145.6(4)	19.12(7)
C(20)	7505.1(5)	-1747.9(4)	295.4(4)	20.26(8)
C(21)	6347.0(5)	-1680.0(4)	1291.3(4)	20.77(8)
C(22)	5769.6(4)	-494.9(4)	1845.7(3)	16.92(7)

Table 16: Anisotropic Displacement Parameters (×10[4]) for **AL24014**. The anisotropic displacement factor exponent takes the form: $-2\pi^2[h^2a^{*2} \times U_{11} + ... + 2hka^* \times b^* \times U_{12}]$

Atom	U_{11}	U_{22}	U_{33}	U_{23}	U_{13}	U_{12}
O(1)	27.86(14)	19.63(13)	11.15(11)	-3.36(11)	-1.83(10)	-6.47(9)
O(2)	13.23(11)	16.85(12)	15.33(11)	-7.76(9)	-3.11(9)	-1.50(9)
O(3)	32.41(16)	20.40(14)	35.79(15)	-16.80(12)	-10.66(13)	1.62(12)
O(4)	21.41(13)	15.08(12)	14.56(12)	2.1(1)	-5.2(1)	-2.41(9)
O(5)	24.11(13)	14.87(12)	9.63(11)	-7.01(10)	-2.43(9)	-2.55(9)
N(1)	13.66(13)	12.03(13)	11.10(13)	-2.84(11)	-0.44(10)	-3.83(10)
C(1)	13.52(15)	14.27(15)	9.53(14)	-2.80(12)	-0.42(11)	-3.66(12)
C(2)	10.59(15)	11.94(15)	11.02(14)	-2.65(12)	-0.53(11)	-3.13(11)
C(3)	12.97(17)	13.55(15)	16.01(17)	-4.29(13)	-1.83(13)	-4.56(12)
H(3a)	22(3)	27(3)	38(3)	-6(2)	-10(3)	-9(2)
H(3b)	39(3)	32(3)	24(3)	-16(3)	2(2)	-7(2)
C(4)	19.80(18)	14.20(17)	25.3(2)	-3.32(14)	-1.62(14)	-8.03(14)
H(4a)	49(4)	39(4)	31(3)	-12(3)	-2(3)	-18(3)
H(4b)	36(4)	25(3)	57(4)	-12(3)	-4(3)	-10(3)
C(5)	14.80(18)	14.71(17)	26.6(2)	0.44(14)	-2.47(15)	-2.86(15)
H(5a)	38(4)	39(4)	37(4)	-15(3)	-8(3)	7(3)
H(5b)	29(3)	22(3)	72(4)	0(3)	4(3)	-21(3)
C(6)	10.91(15)	16.58(16)	18.59(18)	-2.44(13)	0.85(13)	-5.22(13)
H(6a)	35(3)	34(3)	25(3)	-8(3)	17(3)	-5(2)
H(6b)	23(3)	43(4)	54(4)	-9(3)	-9(3)	-20(3)
C(7)	10.27(14)	11.41(14)	9.50(15)	-4.05(12)	-1.19(11)	-1.52(12)
H(7)	24(3)	19(3)	19(3)	-8(2)	-6(2)	2(2)
C(8)	10.54(14)	12.64(15)	11.09(14)	-4.84(12)	-0.68(11)	-1.64(12)
C(9)	12.42(15)	12.60(15)	11.65(14)	-4.41(12)	0.14(12)	-0.94(12)
H(9)	34(3)	28(3)	28(3)	-20(3)	-1(2)	1(2)
C(10)	13.71(16)	16.29(16)	18.85(16)	-7.40(13)	0.16(13)	-6.49(13)
C(11)	17.00(18)	29.6(2)	26.1(2)	-12.55(17)	-3.75(15)	-7.90(16)
H(11a)	54(4)	60(5)	65(5)	-41(4)	-17(4)	-11(4)
H(11b)	34(4)	58(5)	82(5)	5(4)	-21(4)	-33(4)
H(11c)	52(5)	112(7)	35(4)	-49(5)	-4(3)	-8(4)
C(12)	12.86(15)	12.95(15)	8.93(15)	-2.20(12)	-1.00(12)	-1.76(12)

Atom	U_{11}	U_{22}	U_{33}	U_{23}	U_{13}	U_{12}
H(12)	33(3)	24(3)	17(3)	-5(2)	-5(2)	3(2)
C(13)	10.41(15)	13.43(15)	10.09(14)	-2.43(12)	-1.80(12)	-3.56(12)
H(13)	26(3)	33(3)	25(3)	-13(2)	-1(2)	-11(2)
C(14)	10.46(14)	11.45(15)	9.59(14)	-3.99(12)	-0.10(11)	-2.97(11)
H(14)	25(3)	25(3)	25(3)	-13(2)	-3(2)	-4(2)
C(15)	11.39(14)	12.95(15)	11.54(15)	-1.00(12)	-1.83(11)	-3.25(12)
C(16)	12.85(14)	12.27(15)	9.37(14)	-5.06(12)	-0.06(11)	-2.86(12)
C(17)	12.90(15)	12.50(15)	12.70(14)	-4.14(12)	0.59(12)	-4.47(12)
C(18)	17.05(16)	15.98(16)	16.71(15)	-7.55(13)	4.32(13)	-7.17(13)
H(18)	44(4)	31(3)	41(3)	-26(3)	17(3)	-18(3)
C(19)	20.34(17)	18.94(17)	19.82(17)	-7.81(14)	6.56(14)	-10.31(14)
H(19)	43(4)	47(4)	44(4)	-24(3)	26(3)	-28(3)
C(20)	24.43(18)	16.95(17)	21.79(17)	-8.09(14)	4.25(14)	-10.31(14)
H(20)	49(4)	30(3)	47(4)	-15(3)	12(3)	-23(3)
C(21)	26.07(19)	17.46(17)	22.87(17)	-11.88(15)	5.28(15)	-8.81(14)
H(21)	59(4)	39(4)	53(4)	-34(3)	18(3)	-19(3)
C(22)	18.99(17)	16.36(16)	17.36(16)	-8.81(13)	4.21(14)	-6.37(13)
H(22)	35(3)	38(3)	35(3)	-23(3)	17(3)	-16(3)

Table 17: Bond Lengths in Å for **AL24014**.

Atom	Atom	Length/Å
O(1)	C(1)	1.2139(4)
O(2)	C(8)	1.3810(4)
O(2)	C(10)	1.3699(4)
O(3)	C(10)	1.1930(5)
O(4)	C(15)	1.2038(4)
O(5)	C(16)	1.2088(4)
N(1)	C(15)	1.4003(4)
N(1)	C(16)	1.3943(4)
N(1)	C(17)	1.4295(4)
C(1)	C(2)	1.5294(4)
C(1)	C(12)	1.5267(4)
C(2)	C(3)	1.5571(5)
C(2)	C(6)	1.5603(5)
C(2)	C(7)	1.5485(4)
C(3)	C(4)	1.5301(5)
C(4)	C(5)	1.5268(5)
C(5)	C(6)	1.5316(5)
C(7)	C(8)	1.5063(4)
C(7)	C(14)	1.5491(4)
C(8)	C(9)	1.3416(5)
C(9)	C(12)	1.5187(5)
C(10)	C(11)	1.4934(5)
C(12)	C(13)	1.5530(5)
C(13)	C(14)	1.5362(4)
C(13)	C(15)	1.5156(4)
C(14)	C(16)	1.5179(4)
C(17)	C(18)	1.3884(5)
C(17)	C(22)	1.3900(5)
C(18)	C(19)	1.3945(5)
C(19)	C(20)	1.3912(5)
C(20)	C(21)	1.3908(5)
C(21)	C(22)	1.3905(5)

Table 18: Bond Angles in ° for **AL24014**.

Atom	Atom	Atom	Angle/°
C(10)	O(2)	C(8)	122.58(3)
C(16)	N(1)	C(15)	112.88(3)
C(17)	N(1)	C(15)	122.71(3)
C(17)	N(1)	C(16)	124.02(3)
C(2)	C(1)	O(1)	123.35(3)
C(12)	C(1)	O(1)	123.16(3)
C(12)	C(1)	C(2)	113.48(3)
C(3)	C(2)	C(1)	109.97(3)
C(6)	C(2)	C(1)	110.53(3)
C(6)	C(2)	C(3)	104.61(3)
C(7)	C(2)	C(1)	106.50(3)
C(7)	C(2)	C(3)	113.17(3)
C(7)	C(2)	C(6)	112.12(3)
C(4)	C(3)	C(2)	105.22(3)
C(5)	C(4)	C(3)	102.45(3)
C(6)	C(5)	C(4)	103.26(3)
C(5)	C(6)	C(2)	105.88(3)
C(8)	C(7)	C(2)	107.66(3)
C(14)	C(7)	C(2)	108.60(2)
C(14)	C(7)	C(8)	107.36(3)
C(7)	C(8)	O(2)	113.30(3)
C(9)	C(8)	O(2)	130.77(3)
C(9)	C(8)	C(7)	115.88(3)
C(12)	C(9)	C(8)	112.39(3)
O(3)	C(10)	O(2)	123.90(3)
C(11)	C(10)	O(2)	109.63(3)
C(11)	C(10)	O(3)	126.47(3)
C(9)	C(12)	C(1)	107.09(3)
C(13)	C(12)	C(1)	105.12(3)
C(13)	C(12)	C(9)	109.63(3)
C(14)	C(13)	C(12)	109.16(3)
C(15)	C(13)	C(12)	109.55(3)
C(15)	C(13)	C(14)	105.47(3)
C(13)	C(14)	C(7)	109.98(3)
C(16)	C(14)	C(7)	111.74(3)
C(16)	C(14)	C(13)	104.77(3)
N(1)	C(15)	O(4)	124.77(3)
C(13)	C(15)	O(4)	127.15(3)
C(13)	C(15)	N(1)	108.08(3)
N(1)	C(16)	O(5)	124.38(3)
C(14)	C(16)	O(5)	127.12(3)
C(14)	C(16)	N(1)	108.50(3)
C(18)	C(17)	N(1)	119.85(3)
C(22)	C(17)	N(1)	119.04(3)
C(22)	C(17)	C(18)	121.11(3)
C(19)	C(18)	C(17)	119.20(3)
C(20)	C(19)	C(18)	120.07(3)
C(21)	C(20)	C(19)	120.18(3)
C(22)	C(21)	C(20)	120.08(3)
C(21)	C(22)	C(17)	119.35(3)

Table 19: Torsion Angles in ° for **AL24014.**

Atom	Atom	Atom	Atom	Angle/°
O(1)	C(1)	C(2)	C(3)	57.00(4)
O(1)	C(1)	C(2)	C(6)	-58.00(4)
O(1)	C(1)	C(2)	C(7)	179.97(4)
O(1)	C(1)	C(12)	C(9)	125.29(4)
O(1)	C(1)	C(12)	C(13)	-118.15(4)
O(2)	C(8)	C(7)	C(2)	116.98(3)
O(2)	C(8)	C(7)	C(14)	-126.26(3)
O(2)	C(8)	C(9)	C(12)	-174.61(4)
O(4)	C(15)	N(1)	C(16)	175.16(4)
O(4)	C(15)	N(1)	C(17)	2.11(4)
O(4)	C(15)	C(13)	C(12)	-61.23(4)
O(4)	C(15)	C(13)	C(14)	-178.60(4)
O(5)	C(16)	N(1)	C(15)	-173.75(4)
O(5)	C(16)	N(1)	C(17)	-0.81(4)
O(5)	C(16)	C(14)	C(7)	55.24(4)
O(5)	C(16)	C(14)	C(13)	174.29(4)
N(1)	C(15)	C(13)	C(12)	117.64(3)
N(1)	C(15)	C(13)	C(14)	0.27(3)
N(1)	C(16)	C(14)	C(7)	-124.17(3)
N(1)	C(16)	C(14)	C(13)	-5.12(3)
N(1)	C(17)	C(18)	C(19)	179.74(3)
N(1)	C(17)	C(22)	C(21)	-179.14(3)
C(1)	C(2)	C(3)	C(4)	-100.83(3)
C(1)	C(2)	C(6)	C(5)	127.12(3)
C(1)	C(2)	C(7)	C(8)	54.54(3)
C(1)	C(2)	C(7)	C(14)	-61.40(3)
C(1)	C(12)	C(9)	C(8)	54.75(3)
C(1)	C(12)	C(13)	C(14)	-63.23(3)
C(1)	C(12)	C(13)	C(15)	-178.27(2)
C(2)	C(3)	C(4)	C(5)	-37.86(3)
C(2)	C(6)	C(5)	C(4)	-32.21(3)
C(2)	C(7)	C(8)	C(9)	-60.90(3)
C(2)	C(7)	C(14)	C(13)	58.32(3)
C(2)	C(7)	C(14)	C(16)	174.24(3)
C(3)	C(4)	C(5)	C(6)	43.23(3)
C(7)	C(8)	C(9)	C(12)	2.82(3)
C(7)	C(14)	C(13)	C(12)	5.46(3)
C(7)	C(14)	C(13)	C(15)	123.10(3)
C(8)	C(9)	C(12)	C(13)	-58.78(3)
C(9)	C(12)	C(13)	C(14)	51.57(3)
C(9)	C(12)	C(13)	C(15)	-63.46(3)
C(12)	C(13)	C(14)	C(16)	-114.77(3)
C(17)	C(18)	C(19)	C(20)	-0.53(4)
C(17)	C(22)	C(21)	C(20)	-0.67(4)
C(18)	C(19)	C(20)	C(21)	0.28(4)
C(19)	C(20)	C(21)	C(22)	0.33(4)

Table 20: Hydrogen Fractional Atomic Coordinates (×10^4) and Equivalent Isotropic Displacement Parameters (Å^2×10^3) for **AL24014.** U_{eq} is defined as 1/3 of the trace of the orthogonalised U_{ij}.

Atom	x	y	z	U_{eq}
H(3a)	4239(6)	6519(6)	3687(5)	28.5(12)
H(3b)	3353(7)	7280(6)	2147(5)	31.2(13)

Atom	x	y	z	U_{eq}
H(4a)	1883(7)	8078(6)	4711(5)	38.4(14)
H(4b)	2145(7)	9293(6)	3194(6)	38.7(14)
H(5a)	301(7)	8759(6)	2148(6)	41.3(15)
H(5b)	-650(7)	9028(6)	3668(6)	42.7(16)
H(6a)	184(7)	6442(6)	4681(5)	35.6(14)
H(6b)	-150(6)	6485(7)	3066(6)	38.7(15)
H(7)	2214(6)	5784(5)	1455(5)	21.9(11)
H(9)	2133(7)	1918(6)	4430(5)	28.6(12)
H(11a)	-1856(8)	3058(8)	1428(7)	53.1(19)
H(11b)	-2219(8)	4780(8)	1673(8)	60(2)
H(11c)	-963(8)	4235(9)	391(7)	63(2)
H(12)	4048(6)	2305(6)	5550(5)	27.9(12)
H(13)	5921(6)	3376(6)	4025(5)	26.3(12)
H(14)	5038(6)	4985(5)	1923(5)	23.6(11)
H(18)	7990(7)	1427(6)	91(6)	34.7(14)
H(19)	8991(7)	-680(7)	-937(6)	41.5(16)
H(20)	7933(7)	-2674(6)	-145(6)	40.0(15)
H(21)	5877(8)	-2537(7)	1617(6)	45.6(16)
H(22)	4847(7)	-410(6)	2611(6)	33.6(13)

Citations

CrysAlisPro Software System, Rigaku Oxford Diffraction, (2024).

L.J. Bourhis and O.V. Dolomanov and R.J. Gildea and J.A.K. Howard and H. Puschmann, The Anatomy of a Comprehensive Constrained, Restrained, Refinement Program for the Modern Computing Environment - Olex2 Disected, *Acta Cryst. A*, (2015), **A71**, 59-71.

O.V. Dolomanov and L.J. Bourhis and R.J. Gildea and J.A.K. Howard and H. Puschmann, Olex2: A complete structure solution, refinement and analysis program, *J. Appl. Cryst.*, (2009), **42**, 339-341.

Sheldrick, G.M., A short history of ShelX, *Acta Cryst.*, (2008), **A64**, 339-341.

```
#==============================================================================
# PLATON/CHECK-(120923) versus check.def version 230825, Entry: AL24014
# Data: AL24014.cif - Type: CIF                 Bond Precision   C-C = 0.0005 A
# Refl: AL24014.fcf - Type: LIST4                                Temp = 120 K
# Audit:OLEX2 1.5-BETA (COMPILED 2024.02.16 SVN.R378C4104 FOR OLEXSYS, GUI SVN.R
# Refin:OLEX2.REFINE 1.5-BETA (BOURHIS ET AL., 2015)
# X-ray CuKa             R(int) = 0.018,  wR2/R(int) =  1.6,  Nref/Npar =   8.3
# Cell   9.3212(3)  10.2824(3)  10.6049(3)    72.894(3)    81.533(3)   66.882(3)
# Wavelength 1.54184  Volume Reported       892.86(6) Calculated       892.86(5)
# SpaceGroup from Symmetry P -1         Hall: -P 1                     triclinic
#                Reported P -1              -P 1                     triclinic
# MoietyFormula C22 H21 N O5
#      Reported C22 H21 N O5
#    SumFormula C22 H21 N O5
#      Reported C22 H21 N O5
# Mr        =    379.40[Calc],    379.42[Rep]       Volume/NonHatoms = 16 Ang**3
# Dx,gcm-3  =     1.411[Calc],     1.411[Rep]
# Z         =         2[Calc],         2[Rep]
# Mu (mm-1) =     0.825[Calc],     0.825[Rep]  Xtal Size =  0.200x0.240x0.500 mm
# F000      =     400.0[Calc],     401.4[Rep]  or F000' =    401.30[Calc]
# Reported   T Limits: Tmin=0.585              Tmax=1.000  AbsCorr = GAUSSIAN
# Calculated T Limits: Tmin=0.789 Tmin'=0.662  Tmax=0.848
# Measured   HKL: Reported  18731, CIF-loop  18731, <Mult>  5.1
# Reported   Hmax= 11, Kmax= 12, Lmax= 13, Nref=   3684        , Th(max)= 76.030
# Obs in FCF Hmax= 11, Kmax= 12, Lmax= 13, Nref=   3684[  3684], Th(max)= 76.030
# Expected   Hmax= 11, Kmax= 12, Lmax= 13, Nref=   3737        , Ratio  =  0.986
# Reported   Rho(min) = -0.11, Rho(max) = 0.09 e/Ang**3 (From CIF)
# Calculated Rho(min) = -0.25, Rho(max) = 0.55 e/Ang**3 (From CIF+FCF data)
#                  w=1/[<sup>2</sup>(Fo<sup>2</sup>)+(0.0142P)<sup>2</sup>+0.0067P],
P=(Fo<sup>2</sup>+2Fc<sup>2</sup>)/3
# R= 0.0476(  3608), wR2= 0.1230(  3684), S = 4.999     (From CIF+FCF data)
# R= 0.0123(  3608), wR2= 0.0291(  3684), S = 1.181     (From FCF data only)
# R= 0.0123(  3608), wR2= 0.0291(  3684), S = 1.181, Npar=  442
#==============================================================================
# For Documentation:http://www.platonsoft.nl/CIF-VALIDATION.pdf
#==============================================================================
#                                                                            *
#==============================================================================
#>>> The Following Improvement and Query ALERTS were generated - (Acta-Mode) <<<
#==============================================================================
Format: alert-number_ALERT_alert-type_alert-level text

088_ALERT_3_C Poor Data / Parameter Ratio ...................       8.33 Note
351_ALERT_3_C Long   C-H (X0.96,N1.08A)  C7       - H7       .       1.11 Ang.
911_ALERT_3_C Missing FCF Refl Between Thmin & STh/L=    0.600         20 Report
1  0  0,   2  2  0,  -1 -1  1,   0 -1  1,  -3  0  1,  -2  0  1,
1  1  1,   2  1  1,   0  2  1,   1 -1  2,  -2  0  2,  -1  0  2,
0  2  2,   1  0  3,   1  1  3,  -1  2  3,   0  4  3,   4  1  9,
1  0 12,   2  0 12,
913_ALERT_3_C Missing # of Very Strong Reflections in FCF ....          8 Note
#==============================================================================
068_ALERT_1_G Reported F000 Differs from Calcd (or Missing)...     Please Check
154_ALERT_1_G The s.u.'s on the Cell Angles are Equal ..(Note)      0.003 Degree
802_ALERT_4_G CIF Input Record(s) with more than 80 Characters          1 Info
912_ALERT_4_G Missing # of FCF Reflections Above STh/L=  0.600         33 Note
933_ALERT_2_G Number of HKL-OMIT Records in Embedded .res File          6 Note
-3  0  1,  -1  2  3,   0  2  1,   0  2  2,   0  4  3,   4  1  9,
978_ALERT_2_G Number C-C Bonds with Positive Residual Density.         13 Info
979_ALERT_1_G NoSpherA2 Scattering Factors Used .............     Please Note
#==============================================================================

ALERT_Level and ALERT_Type Summary
==================================
4 ALERT_Level_C = Check. Ensure it is Not caused by an Omission or Oversight
7 ALERT_Level_G = General Info/Check that it is not Something Unexpected
```

```
3 ALERT_Type_1 CIF Construction/Syntax Error, Inconsistent or Missing Data.
2 ALERT_Type_2 Indicator that the Structure Model may be Wrong or Deficient.
4 ALERT_Type_3 Indicator that the Structure Quality may be Low.
2 ALERT_Type_4 Improvement, Methodology, Query or Suggestion.
#==============================================================================

0 Missing  Experimental  Info Issue(s) (Out of  65 Tests) -  100 % Satisfied
0 Experimental  Data  Related Issue(s) (Out of  35 Tests) -  100 % Satisfied
4 Structural  Model   Related Issue(s) (Out of 144 Tests) -   97 % Satisfied
7 Unresolved or to be Checked Issue(s) (Out of 279 Tests) -   97 % Satisfied

#                                                                             *
```

10.4. X-Ray Crystal Structure of 15: CCDC Number 2425363:

Table S4: Crystal data and structure refinement for **15**

**Compound**	**AL24010**
Formula	$C_{20}H_{19}N_3O_5$
$D_{calc.}$/ g cm^{-3}	1.444
μ/mm^{-1}	0.106
Formula Weight	381.391
Colour	colourless
Shape	block-shaped
Size/mm^3	0.35×0.14×0.12
T/K	100.00
Crystal System	triclinic
Space Group	P-1
a/Å	9.1761(3)
b/Å	10.7208(3)
c/Å	18.9033(6)
α/°	79.021(1)
β/°	82.945(1)
γ/°	74.605(1)
V/Å^3	1754.94(9)
Z	4
Z'	2
Wavelength/Å	0.71073
Radiation type	Mo K$_\alpha$
Θ_{min}/°	2.20
Θ_{max}/°	33.16
Measured Refl's.	98252
Indep't Refl's	13366
Refl's I≥2 σ(I)	11052
R_{int}	0.0475
Parameters	847
Restraints	0
Largest Peak	0.2894
Deepest Hole	-0.2427
GooF	1.0982
wR_2 (all data)	0.0410
wR_2	0.0367
R_1 (all data)	0.0367
R_1	0.0242

Experimental. Single colourless block-shaped crystals of **AL24010** recrystallised from acetone by slow cooling. A suitable crystal with dimensions 0.35 × 0.14 × 0.12 mm^3 was selected and mounted on a mitegen tip in Paratone oil. on a Bruker D8 VENTURE diffractometer. The crystal was kept at a steady T = 100.00 K during data collection. The structure was solved with the ShelXT 2018/2 (Sheldrick, 2018) solution program using dual methods and by using Olex2 1.5-beta (Dolomanov et al., 2009) as the graphical interface. The model was refined with olex2.refine 1.5-beta (Bourhis et al., 2015) using full matrix least squares minimisation on $\boldsymbol{F}^2$.

Crystal Data. $C_{20}H_{19}N_3O_5$, M_r = 381.391, triclinic, P-1 (No. 2), a = 9.1761(3) Å, b = 10.7208(3) Å, c = 18.9033(6) Å, α = 79.021(1)°, β = 82.945(1)°, γ = 74.605(1)°, V = 1754.94(9) Å^3, T = 100.00 K, Z = 4, Z' =

2, μ(Mo K_α) = 0.106, 98252 reflections measured, 13366 unique (R_{int} = 0.0475) which were used in all calculations. The final wR_2 was 0.0410 (all data) and R_1 was 0.0242 (I≥2 σ(I)).

Structure Quality Indicators

**Reflections:**	d min (MoKα) 2Θ=66.3° 0.65	I/σ(I) 33.6	Rint m=7.35 4.75%	Full 50.5° 100
**Refinement:**	Shift -0.001	Max Peak 0.3	Min Peak -0.2	GooF 1.098

A colourless block-shaped-shaped crystal with dimensions 0.35 × 0.14 × 0.12 mm^3 was mounted on a mitegen tip in Paratone oil.. Data were collected using a Bruker D8 VENTURE diffractometer equipped with an Oxford Cryosystems Cryostream 700+ low-temperature device operating at T = 100.00 K.

Data were measured using ϕ and ω scans with Mo K_α radiation. The diffraction pattern was indexed and the total number of runs and images was based on the strategy calculation from the program APEX5. The maximum resolution that was achieved was Θ = 33.16° (0.65 Å).

The unit cell was refined using SAINT V8.40B (Bruker, 2016) on 9436 reflections, 10% of the observed reflections.

Data reduction, scaling and absorption corrections were performed using SAINT V8.40B (Bruker, 2016). The final completeness is 99.95 % out to 33.16° in Θ. SADABS-2016/2 (Bruker, 2016/2) was used for absorption correction. wR_2(int) was 0.0917 before and 0.0554 after correction. The Ratio of minimum to maximum transmission is 0.9479. The λ/2 correction factor is Not present. The absorption coefficient μ of this material is 0.106 mm^{-1} at this wavelength (λ = 0.71073Å) and the minimum and maximum transmissions are 0.708 and 0.747.

The structure was solved and the space group P-1 (# 2) determined by the ShelXT 2018/2 (Sheldrick, 2018) structure solution program using using dual methods and refined by full matrix least squares minimisation on $\boldsymbol{F}^2$ using version of olex2.refine 1.5-beta (Bourhis et al., 2015). All non-hydrogen atoms were refined anisotropically. Hydrogen atom positions were calculated geometrically and refined using the riding model.

_refine_special_details: NoSpherA2 refinement.

_olex2_refine_details: Refinement using NoSpherA2, an implementation ofNOn-SPHERical Atom-form-factors in Olex2.Please cite:F. Kleemiss et al. Chem. Sci. DOI 10.1039/D0SC05526C - 2021NoSpherA2 implementation of HAR makes use oftailor-made aspherical atomic form factors calculatedon-the-fly from a Hirshfeld-partitioned electron density (ED) - not fromspherical-atom form factors.The ED is calculated from a gaussian basis set single determinant SCFwavefunction - either Hartree-Fock or DFT using selected funtionals - for a fragment of the crystal.This fragment can be embedded in an electrostatic crystal field by employing cluster chargesor modelled using implicit solvation models, depending on the software used.The following options were used: SOFTWARE: ORCA 5.0 PARTITIONING: NoSpherA2 INT ACCURACY: Normal METHOD: R2SCAN BASIS SET: cc-pVTZ CHARGE: 0 MULTIPLICITY: 1 DATE: 2024-07-05_17-04-13

_exptl_absorpt_process_details: SADABS-2016/2 (Bruker,2016/2) was used for absorption correction.wR_2(int) was 0.0917 before and 0.0554 after correction.The Ratio of minimum to maximum transmission is 0.9479.The λ/2 correction factor is Not present.

The value of Z' is 2. This means that there are two independent molecules in the asymmetric unit. The moiety formula is C20 H19 N3 O5.

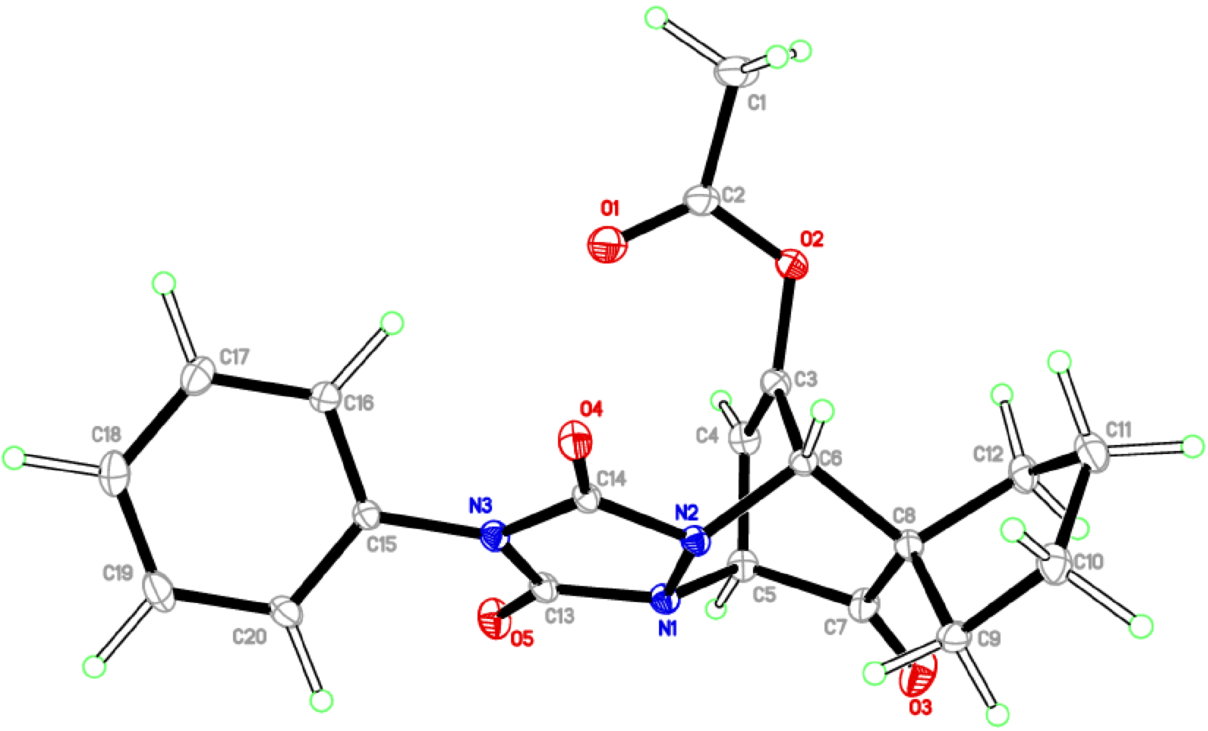

Figure S4 One of the two unique molecules in the asymmetric unit of **15**. Displacement ellipsoids are at the 50% probability level.

Data Plots: Diffraction Data

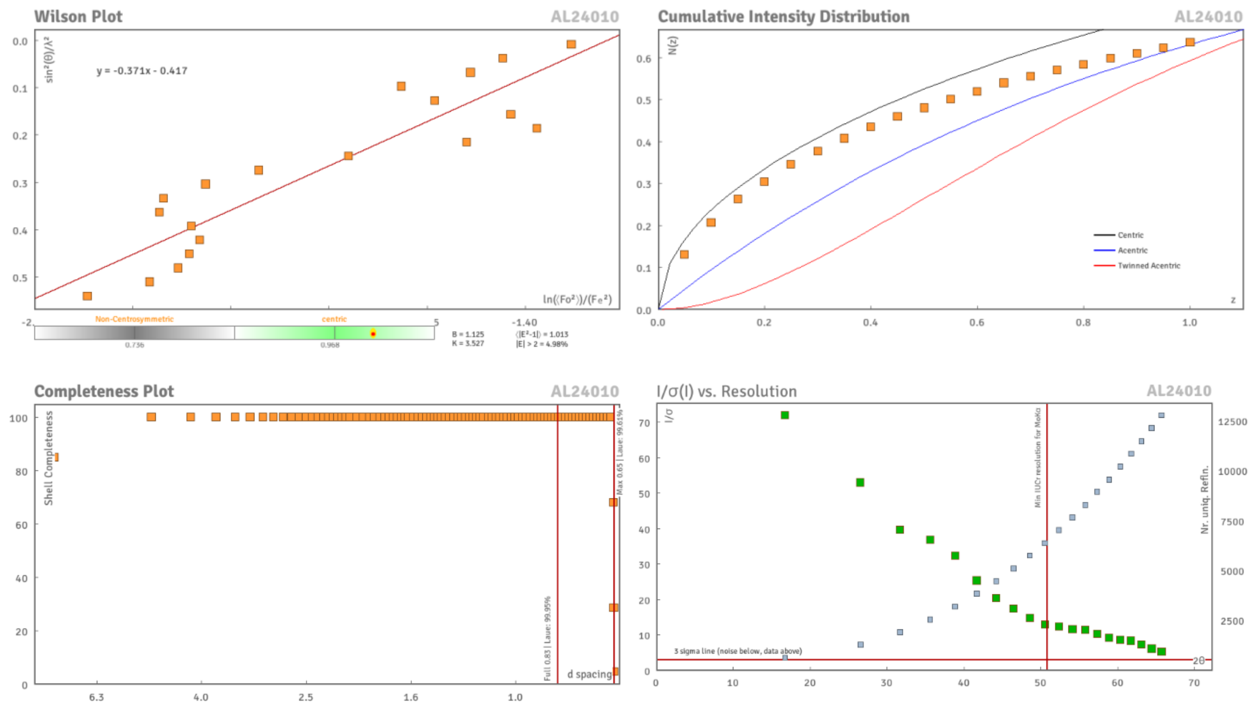

Data Plots: Refinement and Data

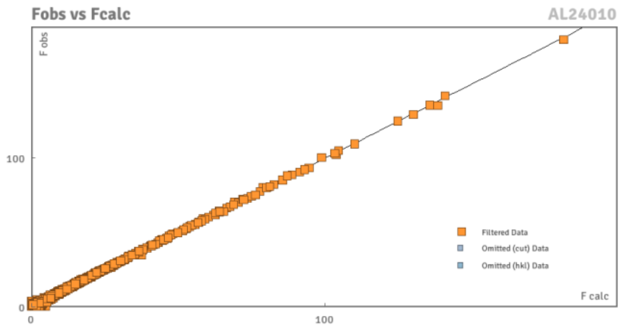

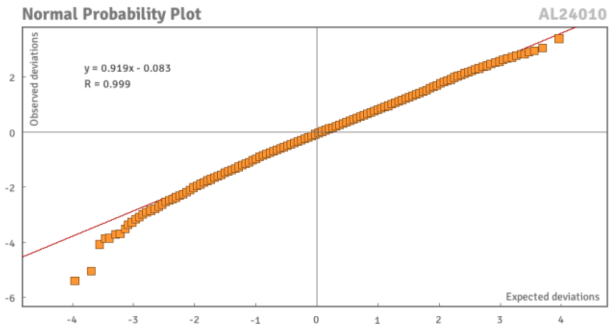

Reflection Statistics

Total reflections (after filtering)	98252	Unique reflections	13366
Completeness	0.996	Mean I/σ	20.8
hkl_{max} collected	(14, 16, 29)	hkl_{min} collected	(-14, -16, -29)
hkl_{max} used	(14, 16, 29)	hkl_{min} used	(-13, -16, 0)
Lim d_{max} collected	100.0	Lim d_{min} collected	0.36
d_{max} used	9.25	d_{min} used	0.65
Friedel pairs	13069	Friedel pairs merged	1
Inconsistent equivalents	0	R_{int}	0.0475
R_{sigma}	0.0297	Intensity transformed	0
Omitted reflections	0	Omitted by user (OMIT hkl)	0
Multiplicity	(923, 3973, 9083, 6307, 2887, 1484, 957, 564, 214, 43)	Maximum multiplicity	17
Removed systematic absences	0	Filtered off (Shel/OMIT)	0

Table 21: Fractional Atomic Coordinates (×10^4) and Equivalent Isotropic Displacement Parameters (Å^2×10^3) for **AL24010**. U_{eq} is defined as 1/3 of the trace of the orthogonalised U_{ij}.

Atom	x	y	z	U_{eq}
O1	2191.4(5)	9232.3(4)	5400.4(2)	28.94(10)
O2	3566.6(4)	7539.6(3)	4881.90(18)	14.87(7)
O3	7705.9(4)	4350.6(4)	6386.0(2)	23.45(9)
O4	4449.5(4)	10259.1(3)	6027.92(18)	13.97(7)
O5	3471.4(4)	6987.1(3)	7834.41(18)	17.29(7)
N1	5263.9(5)	7013.9(4)	6852.9(2)	11.41(8)
N2	5522.8(4)	8022.4(4)	6273.4(2)	10.30(7)
N3	3699.5(4)	8908.3(4)	7055.0(2)	11.09(7)
C1	1908.1(7)	9229.3(7)	4151.0(3)	20.26(11)
C2	2521.0(6)	8715.5(5)	4874.9(3)	16.13(10)
C3	4370.7(5)	7081.2(5)	5491.4(3)	12.19(9)
C4	4096.0(6)	6144.6(5)	6025.8(3)	13.79(9)
C5	5238.0(6)	5802.8(5)	6587.1(3)	12.72(9)
C6	5721.0(5)	7595.0(5)	5558.2(2)	10.10(8)
C7	6827.0(6)	5389.1(5)	6205.1(3)	13.84(9)
C8	7149.8(5)	6429.9(4)	5575.0(2)	11.37(9)
C9	8611.7(6)	6841.9(5)	5627.4(3)	14.02(9)
C10	9119.1(6)	7356.0(5)	4842.2(3)	16.2(1)
C11	8300.4(7)	6812.3(6)	4350.5(3)	17.74(10)
C12	7466.0(6)	5891.9(5)	4857.1(3)	15.25(10)
C13	4049.1(5)	7568.0(5)	7310.3(2)	11.76(9)
C14	4521.5(5)	9195.9(4)	6398.4(2)	10.25(8)

Atom	x	y	z	U_{eq}
C15	2575.5(5)	9848.3(4)	7397.7(2)	10.83(8)
C16	1401.1(6)	10673.4(5)	7006.5(3)	13.36(9)
C17	336.1(6)	11627.0(5)	7333.2(3)	16.31(10)
C18	435.0(6)	11725.8(5)	8048.4(3)	17.97(10)
C19	1595.8(6)	10871.0(5)	8440.0(3)	17.96(10)
C20	2681.4(6)	9932.1(5)	8114.3(3)	14.82(9)
O51	-785.0(4)	3310.5(4)	9558.9(2)	20.34(8)
O52	-2965.0(4)	4838.0(3)	9354.06(18)	15.06(7)
O53	-3028.7(5)	8862.7(4)	7586.0(2)	25.25(9)
O54	1274.1(4)	5192.7(4)	6888.79(18)	16.89(7)
O55	1915.3(4)	4542.2(3)	9322.65(17)	13.93(7)
N51	50.9(4)	6276.6(4)	7824.8(2)	12.06(8)
N52	229.0(4)	6052.5(4)	8575.1(2)	11.11(7)
N53	2045.7(4)	4612.5(4)	8070.5(2)	10.90(7)
C00I	1436.3(5)	4998.1(4)	8733.7(2)	10.38(8)
C51	-3057.4(8)	3075.2(7)	10304.6(3)	23.21(12)
C52	-2108.8(6)	3712.7(5)	9722.5(3)	15.39(9)
C53	-2201.3(5)	5551.2(5)	8820.7(2)	12.04(9)
C54	-2361.1(5)	5651.5(5)	8118.0(3)	12.23(9)
C55	-1553.4(5)	6619.8(5)	7662.2(3)	11.82(9)
C56	-1220.3(5)	6364.0(5)	9007.4(3)	11.23(9)
C57	-2272.6(6)	7914.6(5)	7943.8(3)	14.59(9)
C58	-1964.5(5)	7833.5(5)	8732.4(3)	14.06(9)
C59	-3413.0(6)	8333.8(7)	9204.8(4)	22.63(12)
C60	-2843.4(7)	8792.9(6)	9811.9(3)	21.43(11)
C61	-1653.9(9)	9486.4(6)	9414.2(3)	27.90(13)
C62	-927.2(6)	8739.9(6)	8792.4(3)	19.07(11)
C63	1137.7(5)	5325.7(5)	7516.7(2)	11.85(9)
C64	3464.8(5)	3702.2(4)	7964.9(2)	11.26(8)
C65	3664.9(6)	2905.0(5)	7440.5(3)	14.80(9)
C66	5067.3(6)	2042.8(5)	7326.4(3)	18.79(10)
C67	6255.0(6)	1961.3(5)	7738.0(3)	18.66(10)
C68	6040.1(6)	2759.9(5)	8260.9(3)	16.82(10)
C69	4651.7(6)	3642.5(5)	8376.3(3)	13.67(9)

Table 22: Anisotropic Displacement Parameters (×10[4]) for **AL24010**. The anisotropic displacement factor exponent takes the form: $-2\pi^2[h^2a^{*2} \times U_{11} + ... +2hka^* \times b^* \times U_{12}]$

Atom	U_{11}	U_{22}	U_{33}	U_{23}	U_{13}	U_{12}
O1	32.8(2)	25.6(2)	24.4(2)	10.63(18)	-12.57(18)	-13.18(17)
O2	14.11(17)	17.96(17)	13.36(16)	-2.40(14)	-2.98(13)	-5.60(13)
O3	20.9(2)	14.04(18)	25.6(2)	3.18(15)	4.22(16)	6.32(15)
O4	18.07(17)	9.61(15)	12.41(16)	-2.68(13)	2.30(13)	-0.65(12)
O5	22.71(19)	14.09(17)	12.75(16)	-5.09(15)	4.24(14)	0.66(13)
N1	13.33(19)	10.35(18)	9.83(18)	-2.81(15)	-0.77(14)	-0.19(14)
N2	11.57(19)	9.77(17)	9.30(17)	-2.82(14)	-0.12(14)	-1.15(14)
N3	12.51(19)	10.70(18)	9.18(17)	-2.40(15)	0.20(14)	-0.82(14)
C1	16.8(3)	26.2(3)	17.2(3)	-5.8(2)	-5.7(2)	1.5(2)
H1a	47(5)	56(6)	39(5)	-30(5)	-10(4)	17(4)
H1b	53(5)	49(5)	29(5)	-11(4)	-13(4)	-5(4)
H1c	23(4)	55(5)	37(5)	6(4)	-7(4)	-1(4)
C2	15.5(2)	17.6(2)	15.6(2)	-2.22(19)	-4.93(19)	-3.73(19)
C3	12.6(2)	12.4(2)	12.1(2)	-2.98(17)	-1.43(17)	-3.36(17)
C4	14.7(2)	13.1(2)	15.1(2)	-5.85(18)	0.26(18)	-3.65(18)
H4	30(4)	30(4)	30(4)	-17(4)	-1(3)	-2(3)

Atom	U_{11}	U_{22}	U_{33}	U_{23}	U_{13}	U_{12}
C5	15.0(2)	9.7(2)	12.5(2)	-3.33(17)	0.83(18)	-0.13(17)
H5	31(4)	25(4)	21(4)	-13(3)	1(3)	1(3)
C6	11.3(2)	9.2(2)	9.4(2)	-2.13(16)	-0.76(16)	-1.18(16)
H6	24(4)	19(4)	18(4)	-7(3)	-2(3)	-2(3)
C7	14.9(2)	9.9(2)	14.0(2)	-1.03(17)	0.74(18)	0.59(17)
C8	12.3(2)	10.0(2)	10.5(2)	-1.49(16)	-0.06(17)	-0.80(16)
C9	12.2(2)	16.5(2)	12.1(2)	-2.92(19)	-1.45(18)	0.10(19)
H9a	32(4)	49(5)	24(4)	-17(4)	5(3)	-20(4)
H9b	23(4)	37(4)	31(4)	2(3)	-10(3)	10(3)
C10	17.3(3)	15.6(2)	14.3(2)	-4.5(2)	2.22(19)	-0.74(19)
H10a	71(6)	25(4)	36(5)	-10(4)	0(4)	-6(4)
H10b	25(4)	62(6)	32(4)	-10(4)	8(3)	-9(4)
C11	22.1(3)	17.8(2)	12.0(2)	-3.9(2)	2.3(2)	-3.02(19)
H11a	40(5)	37(5)	48(5)	-13(4)	18(4)	-24(4)
H11b	48(5)	30(4)	35(5)	-4(4)	-19(4)	14(4)
C12	18.2(3)	12.4(2)	14.7(2)	-2.25(19)	1.48(19)	-5.06(18)
H12a	50(5)	15(4)	36(5)	6(4)	2(4)	-4(3)
H12b	32(4)	52(5)	33(4)	-15(4)	7(4)	-27(4)
C13	14.0(2)	11.5(2)	9.2(2)	-3.56(17)	-0.03(17)	-0.37(16)
C14	11.6(2)	9.9(2)	9.4(2)	-3.11(16)	-0.38(16)	-1.44(16)
C15	10.9(2)	12.3(2)	9.2(2)	-2.82(17)	-0.11(16)	-2.09(16)
C16	12.0(2)	15.1(2)	11.7(2)	-1.38(18)	-0.73(18)	-2.05(18)
H16	30(4)	34(4)	22(4)	3(3)	-2(3)	-12(3)
C17	12.5(2)	16.1(2)	18.2(2)	-1.19(19)	1.29(19)	-2.53(19)
H17	29(4)	37(5)	42(5)	-2(4)	0(4)	1(4)
C18	16.1(2)	18.5(2)	19.6(3)	-4.6(2)	5.6(2)	-8.0(2)
H18	36(5)	38(5)	40(5)	-3(4)	11(4)	-20(4)
C19	19.4(3)	23.3(3)	13.3(2)	-6.9(2)	3.1(2)	-8.7(2)
H19	41(5)	55(5)	28(4)	-8(4)	-4(4)	-21(4)
C20	15.6(2)	19.1(2)	10.2(2)	-4.11(19)	-1.18(18)	-3.65(18)
H20	35(4)	37(5)	26(4)	0(4)	-9(3)	-4(3)
O51	17.38(19)	18.42(19)	22.14(19)	-3.12(15)	0.86(15)	0.79(15)
O52	12.47(17)	20.58(18)	12.41(16)	-6.93(14)	-1.07(13)	0.67(13)
O53	32.4(2)	13.34(18)	29.0(2)	2.60(16)	-17.90(18)	-2.86(16)
O54	16.64(18)	24.34(19)	8.53(16)	-2.70(15)	-0.70(13)	-3.42(14)
O55	12.98(16)	18.36(17)	8.64(15)	-1.17(13)	-1.93(13)	-0.95(13)
N51	11.43(18)	14.75(19)	8.87(17)	-2.49(15)	-1.09(14)	-0.09(14)
N52	10.08(18)	13.36(19)	9.24(17)	-1.98(15)	-1.04(14)	-1.41(14)
N53	10.72(18)	12.63(18)	8.93(17)	-2.25(14)	-0.30(14)	-1.94(14)
C00I	9.8(2)	12.8(2)	8.5(2)	-2.90(16)	-0.93(16)	-1.39(16)
C51	23.9(3)	27.8(3)	18.6(3)	-14.1(3)	-0.9(2)	5.0(2)
H51a	119(10)	189(13)	30(6)	-97(9)	-9(6)	10(7)
H51b	34(6)	157(12)	153(11)	-19(7)	18(7)	117(10)
H51c	226(15)	87(8)	108(9)	-109(10)	120(9)	-67(7)
C52	16.3(2)	18.2(2)	12.6(2)	-7.85(19)	-1.08(18)	0.12(18)
C53	11.7(2)	15.0(2)	10.1(2)	-4.74(17)	-1.49(16)	-1.37(17)
C54	13.5(2)	13.6(2)	10.5(2)	-4.57(17)	-3.05(17)	-1.11(17)
H54	32(4)	32(4)	25(4)	-16(4)	-8(3)	-5(3)
C55	12.3(2)	13.0(2)	9.6(2)	-2.49(17)	-2.87(17)	-0.23(17)
H55	29(4)	27(4)	20(4)	-7(3)	-2(3)	-1(3)
C56	10.0(2)	14.3(2)	9.9(2)	-2.95(17)	-0.98(17)	-3.12(17)
H56	22(4)	29(4)	18(4)	-3(3)	-3(3)	-3(3)
C57	14.8(2)	11.6(2)	17.7(2)	-1.92(18)	-6.46(18)	-1.87(18)
C58	11.4(2)	14.3(2)	17.4(2)	-1.09(17)	-3.63(18)	-6.17(18)
C59	12.4(3)	28.4(3)	29.1(3)	0.6(2)	-3.4(2)	-16.7(3)
H59a	28(4)	59(6)	51(5)	27(4)	-23(4)	-24(4)
H59b	29(5)	54(5)	59(5)	-12(4)	20(4)	-29(4)
C60	20.8(3)	22.0(3)	21.7(3)	0.0(2)	-4.3(2)	-10.1(2)

Atom	U_{11}	U_{22}	U_{33}	U_{23}	U_{13}	U_{12}
H60a	35(5)	37(5)	66(6)	10(4)	-8(4)	-36(4)
H60b	58(5)	29(4)	25(4)	0(4)	-13(4)	3(3)
C61	46.4(4)	23.4(3)	20.6(3)	-16.3(3)	-3.4(3)	-8.5(2)
H61a	87(7)	157(10)	27(5)	-81(7)	6(5)	-37(6)
H61b	147(10)	21(5)	61(6)	-2(6)	21(6)	-8(5)
C62	17.7(3)	16.1(2)	26.3(3)	-5.0(2)	-6.1(2)	-6.6(2)
H62a	20(4)	34(5)	127(8)	0(4)	-31(5)	-26(5)
H62b	69(6)	46(5)	32(5)	-40(5)	-2(4)	-5(4)
C63	11.5(2)	15.3(2)	8.5(2)	-3.42(17)	-0.62(16)	-1.15(16)
C64	11.1(2)	11.1(2)	11.7(2)	-3.15(16)	0.01(17)	-2.10(16)
C65	13.1(2)	15.8(2)	17.7(2)	-4.95(19)	1.21(19)	-7.67(19)
H65	31(4)	33(4)	38(4)	-8(4)	-7(4)	-17(4)
C66	15.3(2)	17.1(2)	26.3(3)	-4.4(2)	3.0(2)	-11.5(2)
H66	31(4)	44(5)	51(5)	-12(4)	6(4)	-29(4)
C67	13.2(2)	14.0(2)	28.5(3)	-1.56(19)	1.5(2)	-7.5(2)
H67	29(4)	31(4)	56(5)	5(4)	2(4)	-17(4)
C68	12.1(2)	15.4(2)	22.1(3)	-0.68(19)	-3.2(2)	-3.64(19)
H68	22(4)	35(5)	43(5)	5(3)	-13(4)	-11(4)
C69	12.0(2)	13.7(2)	15.0(2)	-1.42(18)	-2.33(18)	-3.23(18)
H69	24(4)	37(4)	28(4)	1(3)	-13(3)	-13(3)

Table 23: Bond Lengths in Å for **AL24010**.

Atom	Atom	Length/Å
O1	C2	1.1967(6)
O2	C2	1.3655(6)
O2	C3	1.3835(6)
O3	C7	1.2053(6)
O4	C14	1.2089(5)
O5	C13	1.2090(5)
N1	N2	1.4306(5)
N1	C5	1.4854(6)
N1	C13	1.3966(6)
N2	C6	1.4840(6)
N2	C14	1.3888(6)
N3	C13	1.3891(6)
N3	C14	1.3973(6)
N3	C15	1.4254(6)
C1	C2	1.4933(7)
C3	C4	1.3333(7)
C3	C6	1.5105(7)
C4	C5	1.5098(7)
C5	C7	1.5375(7)
C6	C8	1.5511(6)
C7	C8	1.5277(7)
C8	C9	1.5389(7)
C8	C12	1.5398(7)
C9	C10	1.5422(7)
C10	C11	1.5423(8)
C11	C12	1.5292(7)
C15	C16	1.3898(7)
C15	C20	1.3913(6)
C16	C17	1.3941(7)
C17	C18	1.3919(7)
C18	C19	1.3925(8)

Atom	Atom	Length/Å
C19	C20	1.3919(7)
O51	C52	1.1990(6)
O52	C52	1.3671(6)
O52	C53	1.3818(6)
O53	C57	1.2045(6)
O54	C63	1.2098(5)
O55	C00I	1.2126(5)
N51	N52	1.4152(5)
N51	C55	1.4756(6)
N51	C63	1.3843(6)
N52	C00I	1.3721(6)
N52	C56	1.4659(6)
N53	C00I	1.3992(6)
N53	C63	1.3950(6)
N53	C64	1.4225(6)
C51	C52	1.4932(7)
C53	C54	1.3351(6)
C53	C56	1.5186(7)
C54	C55	1.5101(7)
C55	C57	1.5344(7)
C56	C58	1.5564(7)
C57	C58	1.5333(7)
C58	C59	1.5375(7)
C58	C62	1.5566(7)
C59	C60	1.5269(8)
C60	C61	1.5250(9)
C61	C62	1.5326(8)
C64	C65	1.3933(7)
C64	C69	1.3954(7)
C65	C66	1.3904(7)
C66	C67	1.3901(8)
C67	C68	1.3906(7)
C68	C69	1.3915(7)

Table 24: Bond Angles in ° for **AL24010**.

Atom	Atom	Atom	Angle/°
C3	O2	C2	116.05(4)
C5	N1	N2	111.88(3)
C13	N1	N2	108.16(4)
C13	N1	C5	118.92(4)
C6	N2	N1	112.29(3)
C14	N2	N1	108.05(3)
C14	N2	C6	121.51(4)
C14	N3	C13	111.07(4)
C15	N3	C13	124.00(4)
C15	N3	C14	124.81(4)
O2	C2	O1	121.50(5)
C1	C2	O1	126.74(5)
C1	C2	O2	111.76(4)
C4	C3	O2	124.64(4)
C6	C3	O2	119.56(4)
C6	C3	C4	115.70(4)
C5	C4	C3	111.70(4)
C4	C5	N1	109.16(4)

Atom	Atom	Atom	Angle/°
C7	C5	N1	103.61(4)
C7	C5	C4	107.99(4)
C3	C6	N2	107.60(4)
C8	C6	N2	106.40(4)
C8	C6	C3	107.89(4)
C5	C7	O3	123.06(4)
C8	C7	O3	124.37(4)
C8	C7	C5	112.57(4)
C7	C8	C6	106.01(4)
C9	C8	C6	112.66(4)
C9	C8	C7	113.37(4)
C12	C8	C6	111.17(4)
C12	C8	C7	111.18(4)
C12	C8	C9	102.59(4)
C10	C9	C8	105.11(4)
C11	C10	C9	106.55(4)
C12	C11	C10	105.82(4)
C11	C12	C8	104.76(4)
N1	C13	O5	126.14(4)
N3	C13	O5	127.89(4)
N3	C13	N1	105.92(4)
N2	C14	O4	126.13(4)
N3	C14	O4	127.50(4)
N3	C14	N2	106.26(4)
C16	C15	N3	119.26(4)
C20	C15	N3	119.50(4)
C20	C15	C16	121.23(4)
C17	C16	C15	119.22(5)
C18	C17	C16	120.06(5)
C19	C18	C17	120.11(5)
C20	C19	C18	120.25(5)
C19	C20	C15	119.09(5)
C53	O52	C52	116.51(4)
C55	N51	N52	112.65(4)
C63	N51	N52	108.22(4)
C63	N51	C55	121.98(4)
C00I	N52	N51	109.18(4)
C56	N52	N51	112.63(4)
C56	N52	C00I	126.74(4)
C63	N53	C00I	110.75(4)
C64	N53	C00I	124.55(4)
C64	N53	C63	124.48(4)
N52	C00I	O55	126.85(4)
N53	C00I	O55	127.41(4)
N53	C00I	N52	105.65(4)
O52	C52	O51	121.98(5)
C51	C52	O51	126.97(5)
C51	C52	O52	111.00(5)
C54	C53	O52	122.77(4)
C56	C53	O52	121.27(4)
C56	C53	C54	115.78(4)
C55	C54	C53	111.61(4)
C54	C55	N51	110.24(4)
C57	C55	N51	105.28(4)
C57	C55	C54	104.50(4)
C53	C56	N52	107.11(4)
C58	C56	N52	105.80(4)
C58	C56	C53	107.88(4)

Atom	Atom	Atom	Angle/°
C55	C57	O53	123.08(5)
C58	C57	O53	124.69(5)
C58	C57	C55	112.19(4)
C57	C58	C56	106.01(4)
C59	C58	C56	111.07(5)
C59	C58	C57	112.57(4)
C62	C58	C56	112.10(4)
C62	C58	C57	111.04(4)
C62	C58	C59	104.21(4)
C60	C59	C58	103.94(4)
C61	C60	C59	102.83(5)
C62	C61	C60	106.03(5)
C61	C62	C58	106.29(5)
N51	C63	O54	125.89(4)
N53	C63	O54	128.36(4)
N53	C63	N51	105.72(4)
C65	C64	N53	119.69(4)
C69	C64	N53	119.48(4)
C69	C64	C65	120.81(4)
C66	C65	C64	119.40(5)
C67	C66	C65	120.43(5)
C68	C67	C66	119.62(5)
C69	C68	C67	120.83(5)
C68	C69	C64	118.89(5)

Table 25: Torsion Angles in ° for **AL24010**.

Atom	Atom	Atom	Atom	Angle/°
O1	C2	O2	C3	-8.96(6)
O2	C3	C4	C5	176.08(5)
O2	C3	C6	N2	129.22(4)
O2	C3	C6	C8	-116.35(4)
O3	C7	C5	N1	119.81(5)
O3	C7	C5	C4	-124.48(5)
O3	C7	C8	C6	-178.84(5)
O3	C7	C8	C9	-54.74(6)
O3	C7	C8	C12	60.22(6)
O4	C14	N2	N1	177.94(5)
O4	C14	N2	C6	-50.18(6)
O4	C14	N3	C13	177.90(5)
O4	C14	N3	C15	1.77(6)
O5	C13	N1	N2	175.94(5)
O5	C13	N1	C5	46.94(6)
O5	C13	N3	C14	-174.69(5)
O5	C13	N3	C15	1.48(6)
N1	N2	C6	C3	53.34(4)
N1	N2	C6	C8	-62.08(4)
N1	N2	C14	N3	1.69(4)
N1	C5	C4	C3	55.09(4)
N1	C5	C7	C8	-60.65(4)
N1	C13	N3	C14	7.73(4)

Atom	Atom	Atom	Atom	Angle/˚
N1	C13	N3	C15	-176.10(3)
N2	C6	C3	C4	-54.21(4)
N2	C6	C8	C7	58.63(4)
N2	C6	C8	C9	-65.91(4)
N2	C6	C8	C12	179.58(4)
N2	C14	N3	C13	-5.91(4)
N2	C14	N3	C15	177.95(3)
N3	C15	C16	C17	-177.48(4)
N3	C15	C20	C19	178.70(4)
C3	C4	C5	C7	-56.92(4)
C3	C6	C8	C7	-56.59(4)
C3	C6	C8	C9	178.87(4)
C3	C6	C8	C12	64.36(4)
C4	C5	C7	C8	55.06(4)
C5	C7	C8	C6	1.62(4)
C5	C7	C8	C9	125.72(4)
C5	C7	C8	C12	-119.31(4)
C6	C8	C9	C10	-84.38(4)
C6	C8	C12	C11	81.80(4)
C7	C8	C9	C10	155.23(4)
C7	C8	C12	C11	-160.34(4)
C8	C9	C10	C11	-18.69(4)
C8	C12	C11	C10	27.51(4)
C9	C10	C11	C12	-5.42(5)
C15	C16	C17	C18	-1.47(6)
C15	C20	C19	C18	-0.97(6)
C16	C17	C18	C19	-0.13(6)
C17	C18	C19	C20	1.37(6)
O51	C52	O52	C53	-5.32(6)
O52	C53	C54	C55	172.88(5)
O52	C53	C56	N52	132.08(4)
O52	C53	C56	C58	-114.42(5)
O53	C57	C55	N51	130.04(5)
O53	C57	C55	C54	-113.80(5)
O53	C57	C58	C56	169.19(5)
O53	C57	C58	C59	47.57(6)
O53	C57	C58	C62	-68.83(6)
O54	C63	N51	N52	176.94(5)
O54	C63	N51	C55	43.81(6)
O54	C63	N53	C00I	-175.09(5)
O54	C63	N53	C64	10.08(7)
O55	C00I	N52	N51	179.40(5)
O55	C00I	N52	C56	-40.41(6)
O55	C00I	N53	C63	177.15(5)
O55	C00I	N53	C64	-8.02(6)
N51	N52	C00I	N53	2.74(4)
N51	N52	C56	C53	56.54(4)
N51	N52	C56	C58	-58.35(4)
N51	C55	C54	C53	54.23(4)
N51	C55	C57	C58	-52.13(4)
N51	C63	N53	C00I	7.19(4)
N51	C63	N53	C64	-167.64(3)
N52	C00I	N53	C63	-6.21(4)
N52	C00I	N53	C64	168.62(3)
N52	C56	C53	C54	-52.67(4)
N52	C56	C58	C57	64.23(4)
N52	C56	C58	C59	-173.20(4)
N52	C56	C58	C62	-57.08(4)

Atom	Atom	Atom	Atom	Angle/°
N53	C64	C65	C66	178.25(5)
N53	C64	C69	C68	-179.16(4)
C53	C54	C55	C57	-58.44(4)
C53	C56	C58	C57	-50.14(4)
C53	C56	C58	C59	72.44(4)
C53	C56	C58	C62	-171.44(4)
C54	C55	C57	C58	64.04(4)
C55	C57	C58	C56	-8.60(4)
C55	C57	C58	C59	-130.21(5)
C55	C57	C58	C62	113.39(4)
C56	C58	C59	C60	87.64(5)
C56	C58	C62	C61	-107.75(5)
C57	C58	C59	C60	-153.66(5)
C57	C58	C62	C61	133.88(5)
C58	C59	C60	C61	41.43(5)
C58	C62	C61	C60	13.00(5)
C59	C60	C61	C62	-33.53(5)
C64	C65	C66	C67	0.91(6)
C64	C69	C68	C67	0.91(6)
C65	C66	C67	C68	-0.76(6)
C66	C67	C68	C69	-0.17(6)

Table 26: Hydrogen Fractional Atomic Coordinates (×10[4]) and Equivalent Isotropic Displacement Parameters (Å^2×10^3) for **AL24010**. U_{eq} is defined as 1/3 of the trace of the orthogonalised U_{ij}.

Atom	x	y	z	U_{eq}
H1a	2623(8)	9819(7)	3839(4)	47(2)
H1b	1937(8)	8438(7)	3876(4)	43(2)
H1c	769(8)	9844(7)	4219(4)	42(2)
H4	3156(7)	5696(6)	6081(3)	28.7(17)
H5	5047(7)	5069(6)	7049(3)	25.3(16)
H6	5835(7)	8426(6)	5147(3)	20.4(15)
H9a	8421(7)	7576(7)	5980(3)	32.1(18)
H9b	9454(7)	5976(6)	5859(3)	33.3(18)
H10a	8806(8)	8414(7)	4741(4)	44(2)
H10b	10357(8)	7030(7)	4745(3)	41(2)
H11a	9072(8)	6316(7)	3960(4)	40.6(19)
H11b	7468(8)	7618(6)	4053(4)	40(2)
H12a	8191(8)	4885(6)	4961(3)	37.3(19)
H12b	6438(8)	5830(7)	4656(3)	36.3(19)
H16	1345(7)	10569(6)	6449(3)	29.6(17)
H17	-553(7)	12295(7)	7027(4)	38.4(19)
H18	-381(8)	12467(6)	8300(4)	38.3(19)
H19	1671(8)	10956(7)	8995(4)	40(2)
H20	3610(8)	9282(6)	8400(3)	34.2(18)
H51a	-2686(13)	2978(13)	10802(5)	105(4)
H51b	-4163(11)	3607(13)	10346(7)	137(6)
H51c	-2969(17)	2167(11)	10231(6)	131(6)
H54	-3017(7)	5140(6)	7895(3)	27.4(17)
H55	-1609(7)	6716(6)	7081(3)	25.8(16)
H56	-1003(7)	6166(6)	9569(3)	23.5(15)
H59a	-4170(8)	9159(8)	8878(4)	50(2)
H59b	-4009(8)	7597(7)	9399(4)	46(2)
H60a	-3766(8)	9442(7)	10097(4)	45(2)
H60b	-2319(8)	7943(6)	10204(3)	40(2)

**Atom**	**x**	**y**	**z**	U_{eq}
H61a	-837(10)	9540(10)	9751(4)	78(3)
H61b	-2215(12)	10506(8)	9189(5)	83(3)
H62a	212(8)	8156(7)	8909(5)	58(3)
H62b	-841(9)	9432(7)	8290(4)	44(2)
H65	2741(7)	2966(6)	7129(3)	32.3(18)
H66	5209(7)	1427(7)	6919(4)	39.2(19)
H67	7334(7)	1277(6)	7653(4)	40.3(19)
H68	6963(7)	2711(6)	8584(4)	34.1(18)
H69	4490(7)	4278(6)	8773(3)	29.3(17)

Citations

APEX5

L.J. Bourhis and O.V. Dolomanov and R.J. Gildea and J.A.K. Howard and H. Puschmann, The Anatomy of a Comprehensive Constrained, Restrained, Refinement Program for the Modern Computing Environment - Olex2 Disected, *Acta Cryst. A*, (2015), **A71**, 59-71.

O.V. Dolomanov and L.J. Bourhis and R.J. Gildea and J.A.K. Howard and H. Puschmann, Olex2: A complete structure solution, refinement and analysis program, *J. Appl. Cryst.*, (2009), **42**, 339-341.

Sheldrick, G.M., ShelXT-Integrated space-group and crystal-structure determination, *Acta Cryst.*, (2015), **A71**, 3-8.

Software for the Integration of CCD Detector System Bruker Analytical X-ray Systems, Bruker axs, Madison, WI (after 2013).

```
#==============================================================================
# PLATON/CHECK-(120923) versus check.def version 230825, Entry: AL24010
# Data: AL24010.cif - Type: CIF                  Bond Precision   C-C = 0.0008 A
# Refl: AL24010.fcf - Type: LIST4                                  Temp = 100 K
# Audit:OLEX2 1.5-BETA (COMPILED 2024.02.16 SVN.R378C4104 FOR OLEXSYS, GUI SVN.R
# Refin:OLEX2.REFINE 1.5-BETA (BOURHIS ET AL., 2015)
# X-ray MoKa              R(int) = 0.047,  wR2/R(int) =  0.9,  Nref/Npar =  15.8
# Cell   9.1761(3)  10.7208(3)  18.9033(6)    79.021(1)    82.945(1)   74.605(1)
# Wavelength 0.71073  Volume Reported      1754.94(9) Calculated      1754.94(9)
# SpaceGroup from Symmetry P -1         Hall: -P 1                     triclinic
#                 Reported P -1               -P 1                     triclinic
# MoietyFormula C20 H19 N3 O5
#      Reported C20 H19 N3 O5
#    SumFormula C20 H19 N3 O5
#      Reported C20 H19 N3 O5
# Mr        =    381.38[Calc],    381.39[Rep]       Volume/NonHatoms = 16 Ang**3
# Dx,gcm-3  =     1.444[Calc],     1.444[Rep]
# Z         =         4[Calc],         4[Rep]
# Mu (mm-1) =     0.106[Calc],     0.106[Rep]  Xtal Size =  0.119x0.135x0.352 mm
# F000      =     800.0[Calc],     800.6[Rep]  or F000' =    800.40[Calc]
# Reported   T Limits: Tmin=0.708              Tmax=0.747  AbsCorr = MULTI-SCAN
# Calculated T Limits: Tmin=0.983 Tmin'=0.963  Tmax=0.987
# Measured   HKL: Reported  98252, Embedded      0, <Mult>  0.0
# Reported   Hmax= 14, Kmax= 16, Lmax= 29, Nref=  13366        , Th(max)= 33.160
# Obs in FCF Hmax= 14, Kmax= 16, Lmax= 29, Nref=  13366[ 13366], Th(max)= 33.164
# Expected   Hmax= 14, Kmax= 16, Lmax= 29, Nref=  13417        , Ratio  =  0.996
# Reported   Rho(min) = -0.24, Rho(max) = 0.29 e/Ang**3 (From CIF)
# Calculated Rho(min) = -0.26, Rho(max) = 0.59 e/Ang**3 (From CIF+FCF data)
#                  w=1/[<sup>2</sup>(Fo<sup>2</sup>)+(0.0028P)<sup>2</sup>+0.0754P],
P=(Fo<sup>2</sup>+2Fc<sup>2</sup>)/3
# R= 0.0468( 11052), wR2= 0.1043( 13366), S = 2.789     (From CIF+FCF data)
# R= 0.0242( 11052), wR2= 0.0411( 13366), S = 1.098     (From FCF data only)
# R= 0.0242( 11052), wR2= 0.0410( 13366), S = 1.098, Npar=  847
#==============================================================================
# For Documentation:http://www.platonsoft.nl/CIF-VALIDATION.pdf
#==============================================================================
#                                                                             *
#==============================================================================
#>>> The Following Improvement and Query ALERTS were generated - (Acta-Mode) <<<
#==============================================================================
Format: alert-number_ALERT_alert-type_alert-level text

222_ALERT_3_C NonSolvent Resd 2  H   Uiso(max)/Uiso(min) Range        5.7 Ratio
411_ALERT_2_C Short Inter H...H Contact  H9A      ..H55      .       2.11 Ang.
1+x,y,z  =      1_655 Check
411_ALERT_2_C Short Inter H...H Contact  H10B     ..H12A     .       2.14 Ang.
2-x,1-y,1-z  =      2_766 Check
#==============================================================================
068_ALERT_1_G Reported F000 Differs from Calcd (or Missing)...     Please Check
154_ALERT_1_G The s.u.'s on the Cell Angles are Equal ..(Note)      0.001 Degree
432_ALERT_2_G Short Inter X...Y Contact  O55      ..C52      .       2.89 Ang.
-x,1-y,2-z  =      2_567 Check
720_ALERT_4_G Number of Unusual/Non-Standard Labels .........          1 Note
C00I
802_ALERT_4_G CIF Input Record(s) with more than 80 Characters         1 Info
910_ALERT_3_G Missing # of FCF Reflection(s) Below Theta(Min).         3 Note
0  1  0,   0  0  1,   0  1  1,
912_ALERT_4_G Missing # of FCF Reflections Above STh/L=  0.600        52 Note
960_ALERT_3_G Number of Intensities with I  <   - 2*sig(I) ...        38 Check
978_ALERT_2_G Number C-C Bonds with Positive Residual Density.        19 Info
979_ALERT_1_G NoSpherA2 Scattering Factors Used .............     Please Note
#==============================================================================

ALERT_Level and ALERT_Type Summary
==================================
```

```
3 ALERT_Level_C = Check. Ensure it is Not caused by an Omission or Oversight
10 ALERT_Level_G = General Info/Check that it is not Something Unexpected

3 ALERT_Type_1 CIF Construction/Syntax Error, Inconsistent or Missing Data.
4 ALERT_Type_2 Indicator that the Structure Model may be Wrong or Deficient.
3 ALERT_Type_3 Indicator that the Structure Quality may be Low.
3 ALERT_Type_4 Improvement, Methodology, Query or Suggestion.
#==============================================================================

0 Missing  Experimental  Info Issue(s) (Out of  65 Tests) -  100 % Satisfied
0 Experimental  Data  Related Issue(s) (Out of  35 Tests) -  100 % Satisfied
2 Structural  Model   Related Issue(s) (Out of 144 Tests) -   99 % Satisfied
11 Unresolved or to be Checked Issue(s) (Out of 279 Tests) -   96 % Satisfied

#                                                                             *
```

11. Computational Methods

The density functional theory (DFT) calculations were performed using the meta-GGA, range-separated hybrid ωB97M-V functional[1] which includes the VV10 nonlocal correlation and is currently the most recommended approximation for estimating barrier heights (RMSD < 2.0 kcal/mol) and also offers accurate estimates of thermochemical data[1]. This density functional approximation (DFA) was used with the def2-TZVP basis set and all the calculations were performed in the gas phase considering the low polarity of the organic solvents used in the experiments. Calculations of two-electron integrals were accelerated using the chain of sphere (RIJCOSX) approximation[2] as implemented in the Orca 5.0.4 program[3], which was used for all the DFT calculations presented in this work. The integration grid in the DFT calculations was set to DEFGRID3, which is one of the tightest grids available in Orca 5.0.4. To identify the minimum energy paths (MEPs) we used the nudged elastic band approach (NEB)[4], and the highest energy geometries along each optimized MEP were next used for the optimization of the transition state (TS) geometries. The character of each stationary point was confirmed by numerical calculations of the Hessian and the associated vibrational frequencies, ensuring that all of the Hessian eigenvalues were positive. TS geometries were confirmed based on the identification of a single imaginary frequency associated with the reaction coordinate. Additionally, the prior MEP calculations confirmed that the initial TS guess structures connected the protonated substrates and products in each case of the considered quinol–enedione rearrangement reactions. The NEB calculations demonstrated that each such rearrangement was a single stage reaction, going through a single saddle point (TS). Thermochemistry and Gibbs free energy differences were estimated through statistical thermodynamics assuming the harmonic oscillator and rigid rotor approximations and the temperature of 298.15 K. The minimum-energy paths as well as the resulting geometries of the substrates, products, transition states and protonated species are compiled in a compressed directory and attached as additional supporting material to this article.

(1) Mardirossian, N.; Head-Gordon, M. ωB97M-V: A Combinatorially Optimized, Range-Separated Hybrid, Meta-GGA Density Functional with VV10 Nonlocal Correlation. *J. Chem. Phys.* **2016**, *144* (21), 214110. https://doi.org/10.1063/1.4952647.

(2) Neese, F.; Wennmohs, F.; Hansen, A.; Becker, U. Efficient, Approximate and Parallel Hartree–Fock and Hybrid DFT Calculations. A ‘Chain-of-Spheres’ Algorithm for the Hartree–Fock Exchange. *Chem. Phys.* **2009**, *356* (1), 98–109. https://doi.org/10.1016/j.chemphys.2008.10.036.
(3) Neese, F. Software Update: The ORCA Program System—Version 5.0. *WIREs Comput. Mol. Sci.* **2022**, *12* (5), e1606. https://doi.org/10.1002/wcms.1606.
(4) Henkelman, G.; Jónsson, H. Improved Tangent Estimate in the Nudged Elastic Band Method for Finding Minimum Energy Paths and Saddle Points. *J. Chem. Phys.* **2000**, *113* (22), 9978–9985. https://doi.org/10.1063/1.1323224.
